# Electrochemical oxidation induced intermolecular aromatic C-H imidation

**DOI:** 10.1038/s41467-019-13524-4

**Published:** 2019-11-29

**Authors:** Xia Hu, Guoting Zhang, Lei Nie, Taige Kong, Aiwen Lei

**Affiliations:** The Institute for Advanced Studies (IAS), College of Chemistry and Molecular Sciences, Wuhan University, Wuhan, 430072 Hubei People’s Republic of China

**Keywords:** Synthetic chemistry methodology, Electrocatalysis

## Abstract

The dehydrogenative aryl C-H/N-H cross-coupling is a powerful synthetic methodology to install nitrogen functionalities into aromatic compounds. Herein, we report an electrochemical oxidation induced intermolecular cross-coupling between aromatics and sulfonimides with high regioselectivity through *N*-radical addition pathway under external-oxidant-free and catalyst-free conditions. A wide variety of arenes, heteroarenes, alkenes and sulfonimides are applicable scaffolds in this transformation. In addition, aryl sulfonamides or amines (aniline derivatives) can be obtained through different deprotection process. The cyclic voltammetry mechanistic study indicates that the *N*-centered imidyl radicals are generated via proton-coupled electron transfer event jointly mediated by tetrabutylammonium acetate and anode oxidation process.

## Abstract (web summary)

The dehydrogenative C-H/N-H cross-coupling serves to install nitrogen functionalities into arenes with the highest atom economy. Here, the authors report an electrochemical cross-coupling between aromatics and sulfonimides through an N-radical addition pathway under oxidant- and catalyst-free conditions.

## Introduction

The ubiquity of nitrogen-containing aromatics in natural products, pharmaceuticals, agrochemicals, and functional materials makes the exploration of efficient methodologies to construct aryl C–N bonds of great importance^1^. Compared with the well-established Buchwald−Hartwig and Ullmann amination^2–8^, the direct amination of aryl C–H bonds, especially the dehydrogenative aryl C–H/N–H cross-coupling, represents a more straightforward and atom-economical strategy to access these essential molecules, which can circumvent the pre-functionalization of the aromatic substrates^9–24^.

**Fig. 1:**
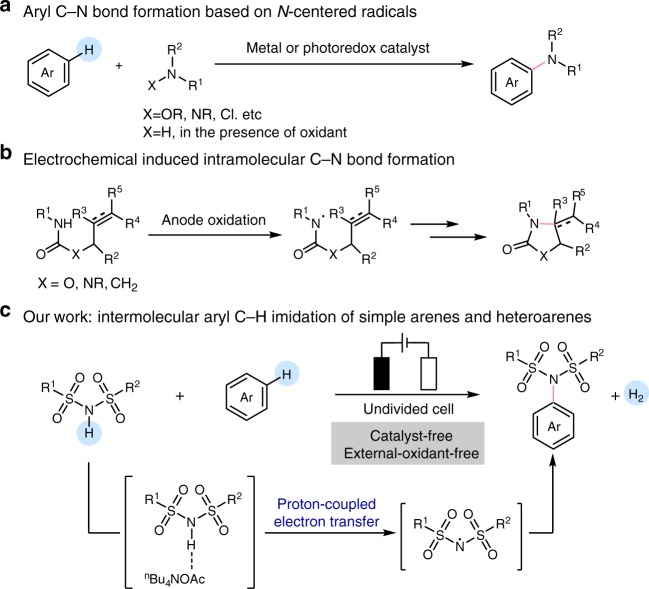
C–N bond formation via radical addition of *N*-centered radical to unsaturated bonds. **a** Aryl C–N bond formation based on *N*-centered radicals. **b** Electrochemical-induced intramolecular C–N bond formation. **c** Our work: intermolecular aryl C–H imidation of simple arenes and heteroarenes.

Although significant progress has been made in nucleophilic or electrophilic amination reactions of aromatic compounds, the formation of aryl C–N bonds based on *N*-centered radicals remains more challenging^25–29^. The lack of convenient methods to produce *N*-centered radical species is one of the major challenges in this field. Mostly, the *N*-centered radical intermediates were generated via the cleavage of reactive N–X (X = oxygen, nitrogen or halogen atom) bond^30–35^. Recent advances in this class have been propelled by the direct oxidative cleavage of the N–H bond (Fig. 1a)^36–45^. Itami^46^, Yu^47^, and Lee^48^ reported the dehydrogenative aromatic C–H imidation under visible-light irradiation conditions, respectively. More recently, Leonori’s group disclosed a regioselective amination of arenes using alkyl amino radicals generated by photo-redox catalysis^49^. However, the stoichiometric oxidants were required in these transformations. In this context, developing an efficient method to generate *N*-centered radicals from N–H reagent under external oxidant-free and catalyst-free conditions to realize dehydrogenative aryl C–H/N–H cross-coupling would be significantly appealing.

Electrochemical synthesis offers a powerful alternative to conventional chemical approaches in oxidative C–H functionalization reactions^50–65^. Particularly, anode oxidation has been proved as a convenient approach to generate *N*-centered radicals by cleavage of the strong N–H bonds from N–H precursors^66^. Although the electrochemical-oxidation-induced amidyl and sulfamidyl radicals have been successfully applied in the intramolecular C–N bond formation (Fig. 1b)^42–45^, the *N*-centered radical-mediated intermolecular aminations of simple arenes and heteroarenes are still limited. Based on our previous research, we found that the intermolecular H-bonding between sulfonamide and acetate would assist the single electron oxidation and deprotonation of the sulfonamide to generate the sulfamidyl radicals^67^. Furthermore, the sulfonimidyl radicals possess higher electrophilicity than sulfamidyl radicals. We hypothesized that the electrochemical oxidation concert deprotonation would be an efficient method to generate an electrophilic *N*-centered radical from sulfonimides, which tend to react with electron-rich aromatics through radical addition pathway. Followed by the sequent electrochemically oxidative aromatization process, the direct electrochemical intermolecular oxidative aryl C(sp^2^)-H amination can be achieved, accompanying with the cathodic hydrogen evolution process (Fig. 1c). Here, we report an electrochemically oxidative intermolecular cross-coupling of simple arenes and heteroarenes with sulfonimides under external-oxidant-free and catalyst-free conditions.

## Results

### Investigation of reaction conditions

Initially, naphthalene (**1aa**) was chosen as a model substrate for the electro-oxidative C–H imidation with dibenzenesulfonimide (**2aa**) as the nitrogen source. To our delight, the cross-coupling product **3aa** can be afforded in acetonitrile with a 43% isolated yield by using a carbon rod anode and a platinum plate cathode in an undivided cell under 10 mA constant current for 4 h at ambient temperature (Table 1, Entry 2). Tetrabutylammonium acetate was used as the electrolyte, simultaneously as a base. An obviously increasing yield can be obtained when fluorine-containing hexafluoroisopropanol (HFIP) was added as co-solvent (Table 1, Entry 3). Further investigation of the solvent revealed that a mix-solvent system, DCM/MeCN/HFIP = 20:4:1, gave the best result (76% isolated yield, Table 1, Entry 1). A significant decline of yield was observed when a platinum plate anode and carbon rod cathode were used, or a nickel plate cathode was used instead of the platinum plate cathode (Table 1, Entries 4–5). However, when the electrolyte was changed to ^*n*^Bu_4_NBF_4_, ^*n*^Bu_4_NPF_6_, or ^*n*^Bu_4_NClO_4_, no reaction can be observed (Table 1, Entry 6). The imidation product **3aa** can be formed with a 46% yield by using NaOAc instead of ^*n*^Bu_4_NOAc (Table 1, Entry 7). These results demonstrated that acetate plays an important role in this transformation. Decreasing the loading of ^*n*^Bu_4_NOAc to 0.5 equivalent or **2aa** to 1.2 equivalent provided reduced yields (Table 1, Entries 8–9). The reaction occured smoothly when decreasing the operating current to 5 mA, while increasing the current to 15 mA led to a slightly lower yield (Table 1, Entries 10–11). It should be noted that the reaction proceeded even when it was exposed to the air (Table 1, Entry 12). As expected, no product **3aa** can be observed without electric current (Table 1, Entry 13).

**Table 1:** Investigation of the reaction conditions.


Entry	Variation from standard condition	Yield (%)^b^
1	None	79(76)^c^
2	CH_3_CN as solvent	45(43)^c^
3	CH_3_CN/HFIP = 12 mL/0.5 mL as solvent	68(65)^c^
4	Pt(+) | C(−) instead of C(+) | Pt(−)	59
5	C(+) | Ni(−) instead of C(+) | Pt(−)	58
6	^*n*^Bu_4_NBF_4_ or ^*n*^Bu_4_NPF_6_ or ^*n*^Bu_4_NClO_4_ instead of ^*n*^Bu_4_NOAc	N.D.
7^a^	NaOAc instead of ^*n*^Bu_4_NOAc	46
8	0.5 equiv. ^*n*^Bu_4_NOAc	67
9	1.2 equiv. **2aa** instead of 1.5 equiv. **2aa**	63
10	5 mA, 8 h instead of 10 mA, 4h	76
11	15 mA, 2.67h instead of 10 mA, 4h	59
12	Reaction in the air	70
13	Without current	N.D.

Reaction conditions: carbon rod anode(Φ 6 mm), platinum plate cathode (15 mm × 15 mm × 0.3 mm), constant current = 10 mA, **1aa** (0.40 mmol), **2aa** (0.60 mmol), ^*n*^Bu_4_NOAc (0.40 mmol), solvent (DCM/CH_3_CN/HFIP = 10 mL/2 mL/0.5 mL), undivided cell, N_2_, 4 h.

*HFIP* hexafluoroisopropanol, *N. D.* not determined.

^a^1.0 equiv. ^*n*^Bu_4_NBF_4_ was used as an electrolyte.

^b^Yield of **3aa** was determined by HPLC analysis with 1,3,5-trimethoxybenzene as the internal standard.

^c^Isolated yields are shown in parentheses.

### Substrate scope

**Fig. 2:**
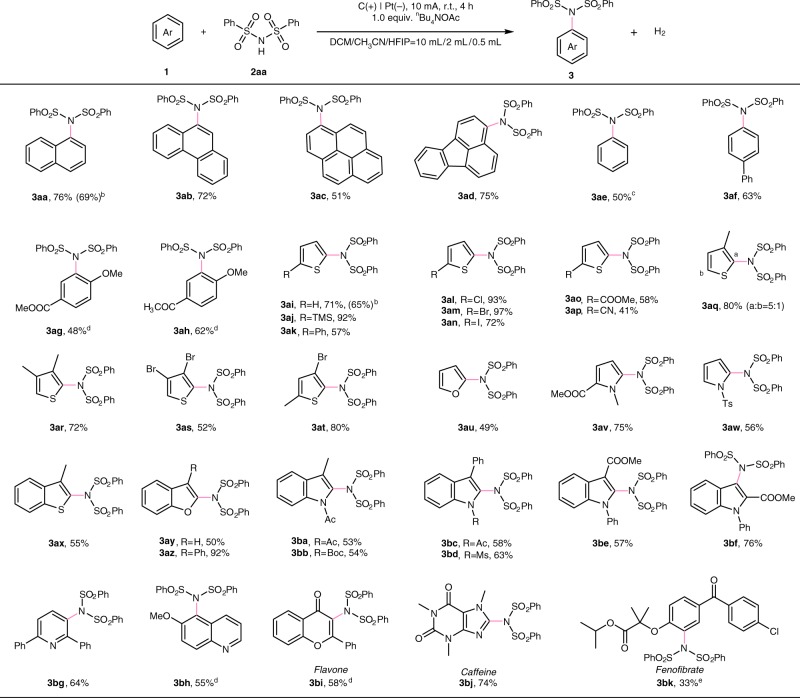
Substrate scope of aromatic compounds. Reaction conditions: carbon rod anode, platinum plate cathode, constant current = 10 mA, **1** (0.40 mmol), **2aa** (0.60 mmol), ^*n*^Bu_4_NOAc (0.40 mmol), solvent (DCM/ CH_3_CN/ HFIP = 10 mL/2 mL/0.5 mL), undivided cell, N_2_, 4 h. Isolated yields. ^b^5 mmol scale. ^c^1 mL benzene and 0.40 mmol **2aa** was used. ^d^5 mA, 8 h. ^e^Solvent (DCE/TFE = 8 mL/4 mL).

With the optimized reaction conditions in hand, the substrate scope of this electro-oxidative aryl C(sp^2^)-H imidation was explored (Fig. 2). A range of polycyclic aromatic hydrocarbons, such as naphthalene (**3aa**, 76%), phenanthrene (**3ab**, 72%), pyrene (**3ac**, 51%), and fluoranthene (**3ad**, 75%), could couple with dibenzenesulfonimide (**2aa**), giving the corresponding imidation product in moderate to good yields with excellent regioselectivity by constant current electrolysis. To our delight, benzene, a simple electron-neutral aromatic compound, can also be adapted to this electro-oxidative system albeit an excess amount of benzene was used (**3ae**). The imidation of biphenyl proceeded at the *para*-position to give **3af** in 63% yield. When ester or carbonyl functional group was introduced at the *para*-position of anisole, the imidation would occur at the *ortho-*position to the methoxy substituent (**3ag-3ah**). This strategy was not only applicable to aromatic hydrocarbons but also heteroaromatics. Thiophene (**3ai**), furan (**3au**), *N*-methyl pyrrole derivatives (**3av**), as well as tosyl-protected pyrrole (**3aw)** underwent regioselective C–H imidation at the α-position. The thiophene deriveatives bearing various functional groups such as silyl, phenyl, halide, ester, cyano groups in the C2 position were also well tolerated in moderate to good yields (**3aj-3ap**). Notably, **3al**, **3am**, and **3an** could be obtained without decomposition of the halide group, which can be applied in the subsequent transformation. 3-Methylthiophene gave the corresponding product as a mixture of regioisomers with ratio of 5:1 (**3aq**, 80%). Besides *mono*-substituted thiophenes, *di*-substituted thiophenes also provided the desired products with good yields (**3ar-3at**). Other 5-membered heteroaromatics, including 3-methylbenzothiophene, benzofuran, *N*-acetyl indole, *N*-methanesulfonyl indole, and *N*-butoxycarbonyl indole derivatives readily underwent C(sp^2^)-H imidation with **2aa**, thus giving the corresponding adducts (**3ax-3az**, **3ba-3bd**). Methyl 1-phenyl-1H-indole-3-carboxylate and methyl 1-phenyl-1H-indole-2-carboxylate would be selectively imidated at the C2 or C3 position (**3be**-**3bf**). Six-membered heterocycles bearing electron-donating substitution are competent substrates. 2,6-Diphenylpyridine and 6-methoxyquinoline gave the imidation products **3bg** and **3bh** as the single regioisomer, respectively. Importantly, the modification of natural products flavone and caffeine could be conducted smoothly with this strategy, yielding the imidated products **3bi** and **3bj**, respectively. Small-molecule drug such as fenofibrate could also give the corresponding imidation product **3bk**. Using the above conditions, this electrochemical oxidation-induced C(sp^2^)-H imidation can be performed in gram-scale. The constant current electrolysis of 5 mmol of **1aa** and **1ai** can be conducted to produce the desired imidation product (**3aa, 3ai**) in 69% and 65% isolated yield, respectively.

**Fig. 3:**
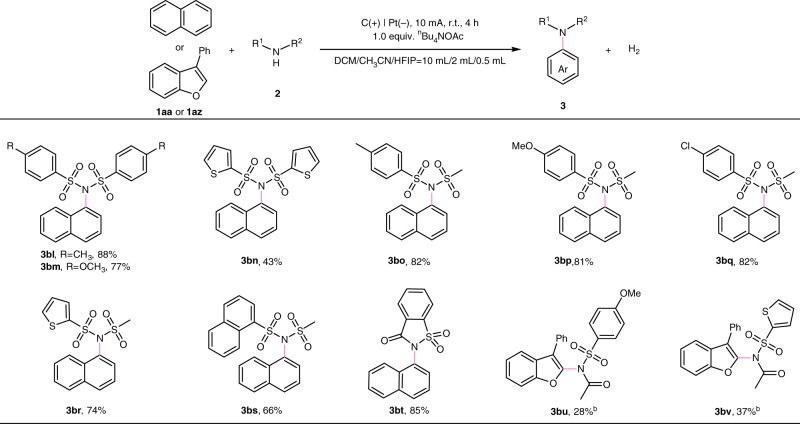
Substrate scope of sulfonimides. Reaction conditions: carbon rod anode, platinum plate cathode, constant current = 10 mA, **1aa** (0.40 mmol), **2** (0.60 mmol), ^*n*^Bu_4_NOAc (0.40 mmol), solvent ((DCM/CH_3_CN/HFIP = 10 mL/2 mL/0.5 mL), undivided cell, N_2_, 4 h. Isolated yields. ^b^ Reaction conditions: carbon cloth anode, platinum plate cathode, constant current = 3 mA, **1az** (0.40 mmol), **2** (0.60 mmol), ^*n*^Bu_4_NBF_4_ (0.40 mmol), NaOAc (0.8 mmol), solvent ((DCM/CH_3_CN/HFIP = 10 mL/2 mL/1 mL), undivided cell, N_2_, 8h.

**Fig. 4:**
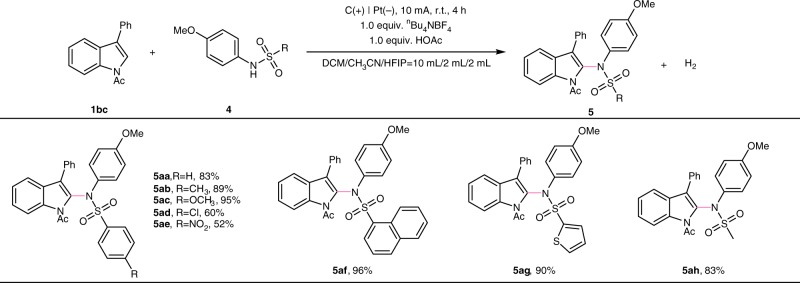
Substrate scope of sulfonamides. Reaction conditions: carbon rod anode, platinum plate cathode, constant current = 10 mA, **1aa** (0.40 mmol), **4** (0.60 mmol), ^*n*^Bu_4_NBF_4_ (0.40 mmol), HOAc (0.4 mmol), solvent (DCM/ CH_3_CN/ HFIP = 10 mL/2 mL/2 mL), undivided cell, N_2_, 4 h. Isolated yields.

Next, the scope of the reaction with respect to sulfonimides was also investigated by using naphthalene as the coupling-partner (Fig. 3). Various functional groups on the phenyl rings of symmetrical diaryl sulfonimides were tolerated well (**3bl**-**3bm**). The sulfonimide with two thienyl substituents could be used as a viable imination reagent to generate the desired product **3bn** in an acceptable yield (43%). Furthermore, *N*-(methylsulfonyl)benzenesulfonamide derivatives could react with naphthalene in good yields, and several substituents in the *para*-position of the phenyl rings were compatible in the reaction system (**3bo**-**3bq**). Besides, *N*-(methylsulfonyl)thiophene-2-sulfonamide and *N*-(methylsulfonyl)naphthalene-1-sulfonamide produced the aryimines **3br** in 74% yield and **3bs** in 66% yield, respectively. It is worth noting that the coupling of saccharin and naphthalene can be achieved successfully by using this method (**3bt**). To our delight, When 3-phenylbenzofuran was used as a coupling-partner, *N*-((4-methoxyphenyl)sulfonyl)acetamide and *N*-(thiophen-2-ylsulfonyl)acetamide were found to be available imidation reagents through the optimization of the reaction conditions (**3bu**-**3bv**). In addition, we have tried to expand the types of amine source. We found that *N*-(4-methoxyphenyl)sulfonamide derivatives could couple with *N*-acetyl indoles (Fig. 4), thus giving the corresponding amination products in moderate to good yields (Fig. 4, **5aa**-**5ah**).

**Fig. 5:**
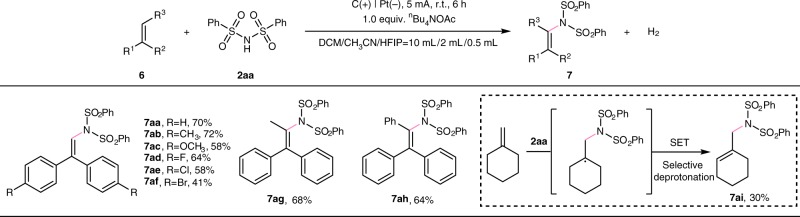
Electrochemical oxidation-induced dehydrogenative imidation of alkenes. Reaction conditions: carbon rod anode, platinum plate cathode, constant current = 5 mA, **6** (0.40 mmol), **2aa** (0.60 mmol), ^*n*^Bu_4_NOAc (0.40 mmol), solvent (DCM/CH_3_CN/HFIP = 10 mL/2 mL/0.5 mL), undivided cell, N_2_, 6 h. Isolated yields.

The dehydrogenative C–H/N–H cross-coupling between alkenes with amines to construct enamines, especially under the external oxidant-free conditions, is of great significance. And the further studies revealed that this electrochemical oxidation-induced system is also applicable for the imidation of alkenes (Fig. 5). And the desired product **7aa** was produced in 70% yield under 5 mA constant current for 6 h at ambient temperature when 1,1-diphenylethylene was employed as the radical acceptor. The 1,1-diphenylethylene derivatives bearing electron-donating and electron-withdrawing substituents both underwent the reaction smoothly with dibenzenesulfonimide (**7ab**-**7af**). In addition, the procedure was also compatible with trisubstituted aryl alkenes to form corresponding imidation product (**7ag**-**7ah**). The allylimine could be obtained as the only isomer when methylenecyclohexane was used as the coupling-partner (**7ai**).

### Deprotection of arylimines

**Fig. 6:**
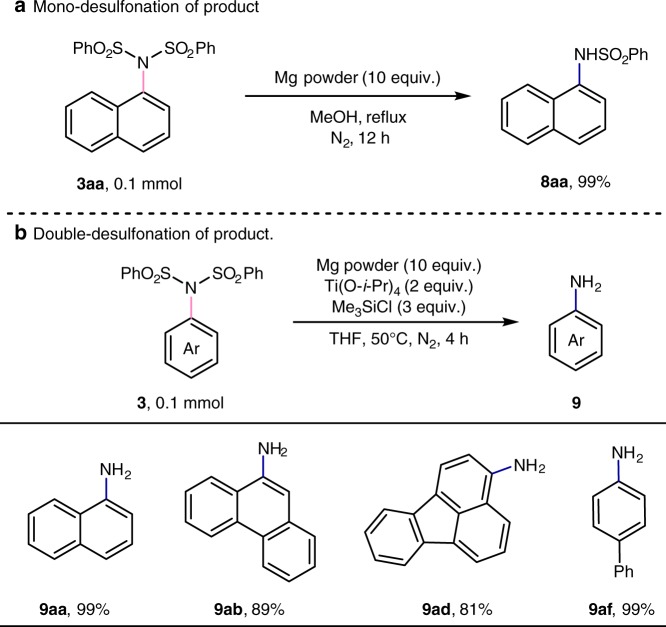
Deprotection of arylimines. **a** Mono-desulfonation of the product. **b** Double-desulfonation of the product.

Since sulfonyl groups are useful and removable protecting groups for amino groups, above C(sp^2^)-H imidation reaction can be further utilized in the synthesis of substituted sulfonamide and aryl amines (Fig. 6). Treating **3aa** with magnesium in methanol at refluxed temperature, *N*-(naphthalen-1-yl)benzenesulfonamide (**8aa**) can be prepared in quantitative yield with high selectivity through the mono-desulfonation process (Fig. 6a). In addition, the double deprotection of sulonimides **3** can be achieved by adding 10 equivalent of magnesium, 2 equivalent of titanium isopropoxide, and 3 equivalent of trimethylsilyl chloride (Fig. 6b). Several aromatic primary amines (**9aa**, **9ab**, **9ad**, **9af**) can be obtained.

## Discussion

**Fig. 7:**
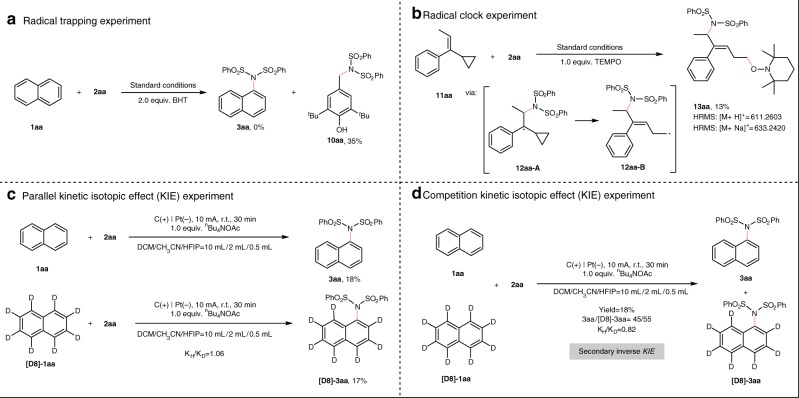
Mechanistic experiments. **a** Radical trapping experiment. **b** Radical clock experiment. **c** Parallel kinetic isotopic effect experiment. **d** Competition kinetic isotopic effect experiment.

To gain a deeper insight into the mechanism of this transformation, some mechanistic experiments were performed. By adding 2,6-di-tert-butyl-4-methylphenol (BHT, 2.0 equivalent) as the radical scavenger, the reaction was completely suppressed, and the benzylic imidation product **10aa** was obtained in 35% isolated yield (Fig. 7a). Furthermore, in the presence of stoichiometric amount of 2,2,6,6-tetramethyl-1-piperidinyloxy (TEMPO), the reaction with radical clock substrate **11aa** gave rise to ring-opening and radical trapping product **13aa** in 13% yield (Fig. 7b), which should be resulted from a radical cross-coupling of intermediate **12aa-B** with TEMPO. These results revealed that radical process was involved in this transformation. Furthermore, parallel reaction and intermolecular competitive reaction kinetic isotope effect (KIE) experiments were also performed (Figs. 7c and d), thus giving KIE values of 1.06 and 0.82, respectively. This interesting secondary inverse KIE provided important information for the understanding of the mechanism. Although the inverse secondary KIE is rarely reported, it can be observed in electrophilic nitration of benzene and carbonyl addition reactions, in which sp^2^ → sp^3^ rehybridization of C–H (C–D) bond is considered to be the rate-determining step^68^. Moreover, the Ritter group^69^ and Itami group^70^ have reported quite distinct KIE values in the aromatic C–H imidation. These results implicated that the addition of the imidyl radical to the arenes might be involved in the rate-determining step of the transformation.

**Fig. 8:**
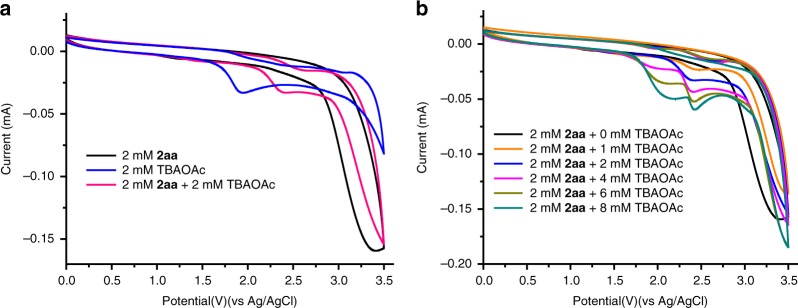
Cyclic voltammograms studies. **a** Cyclic voltammograms of dibenzenesulfonimide (2 mM), ^*n*^Bu_4_NOAc (2 mM), and equivalent mixture of the dibenzenesulfonimide with ^*n*^Bu_4_NOAc in acetonitrile containing 0.1 M ^*n*^Bu_4_NBF_4_. **b** Cyclic voltammograms of dibenzenesulfonimide (2 mM) in acetonitrile containing 0.1 M ^*n*^Bu_4_NBF_4_ and varying concentrations of ^*n*^Bu_4_NOAc.

To investigate the interaction between acetate anion and sulfonimide, thus understanding the elementary steps for the generation of *N*-centered imidyl radical, the cyclic voltammetry studies were performed (Fig. 8). We found that the oxidation potential of dibenzenesulfonimide alone is relatively high (*E*_1/2_ = +3.28 V vs Ag/AgCl in acetonitrile). The oxidation potential of ^*n*^Bu_4_NOAc (tetrabutylammonium acetate) could be observed at 1.78 V (vs Ag/AgCl in acetonitrile). Specifically, we also carried out cyclic voltammetry studies on dibenzenesulfonimide in acetonitrile containing 0.1 M ^*n*^Bu_4_NBF_4_ in the presence of 1 equivalent of ^*n*^Bu_4_NOAc. The oxidation peaks of the dibenzenesulfonimide and ^*n*^Bu_4_NOAc disappeared with the generation of a new oxidation peak. Compared with dibenzenesulfonimide, the oxidation potential and onset potential of the new peak were shifted to less positive potentials. Importantly, we found that the current response increased  with increasing concentrations of ^*n*^Bu_4_NOAc. These outcomes were consistent with a proton-coupled electron transfer process in this transformation.

**Fig. 9:**
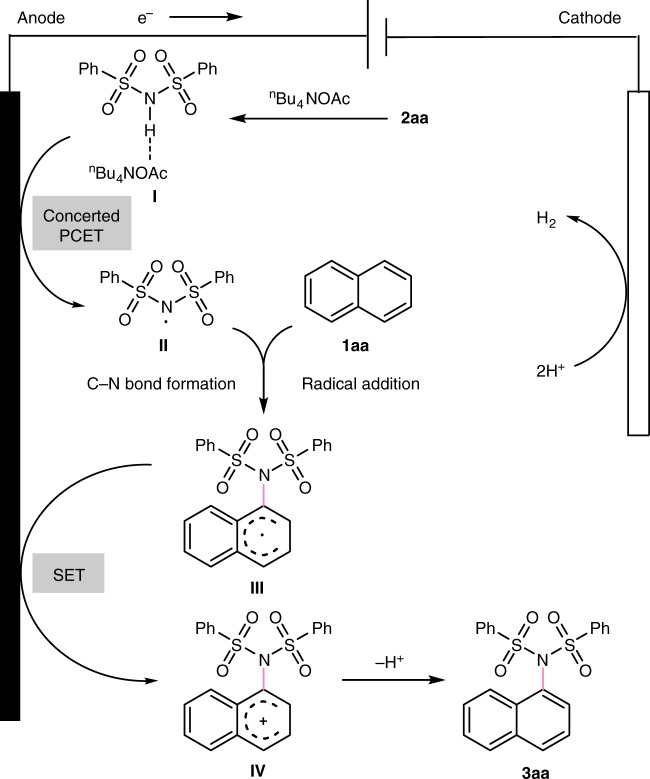
Proposed mechanism of electrochemical oxidation-induced intermolecular aromatic C–H imidation. A plausible mechanism involves the homolysis of the N–H bond to afford intermediate **II**, subsequent radical addition, single electron oxidation, and deprotonation to form the desired product **3aa**. Protons undergo cathodic reduction to generate hydrogen.

A plausible mechanism for the electrochemical oxidation-induced dehydrogenative imidation of arenes is outlined in Fig. 9. The tetrabutylammonium acetate would first form a hydrogen bonded complex **I** with the dibenzenesulfonimide N–H bond. The resulting adduct **I** would undergo a concerted proton-coupled electron transfer event with the single electron oxidation on the anode, leading the homolysis of the N–H bond and generation of the key sulfonimidyl radical intermediate **II**. The subsequent radical addition to naphthalene furnished a new C–N bond and a vicinal carbon-centered radical **III**. Then the radical species underwent further oxidation to furnish a carbon cation intermediate **IV**, which finally was aromatized to provide the aryl C(sp^2^)–H imidation product through proton elimination. Concomitant cathodic reduction of generated protons would generate H_2_ during the reaction process, which can avoid the requirement of external oxidant in the transformation.

In summary, we have developed a method for the electrochemical oxidation-induced dehydrogenative C(sp^2^)-H/N-H cross-coupling between arenes/alkenes and sulfonimides. Neither transition metal catalyst nor stoichiometric additional oxidant was required in this transformation. The electro-synthetic method exhibited a broad substrate scope and provided access to various aryl sulfonimides and alkenyl sulfonimides. The reaction can be easily scaled up, demonstrating the synthetic potential of this method. The cyclic voltammetry confirmed that the *N*-centered imidyl radical intermediate was generated through a concerted proton-coupled electron transfer process, demonstrating the potential benefits of concerted proton-coupled electron transfer activation in electrochemical synthesis.

## Methods

### General procedure

The synthesis of **3aa** is representative: naphthalene (0.4 mmol, 1 equiv.), diphenylsulfonimide (0.6 mmol, 1.5 equiv.), ^*n*^Bu4NOAc (0.40 mmol, 1 equiv.) were placed in an oven-dried undivided three-necked bottle (25 mL). The bottle was equipped with a stir bar, a carbon rod (Φ 6 mm) anode and a platinum plate (15 mm × 15 mm × 0.3 mm) cathode. The bottle was flushed with nitrogen. Degased dry dichloromethane (DCM, 10 mL), degased dry acetonitrile (MeCN, 2 mL) and commercially available hexafluoroisopropanol (HFIP, 0.5 mL) were added. The reaction mixture was stirred and electrolyzed at a constant current of 10 mA at room temperature for 4 h. After completion of the reaction, the product was identified by TLC. The solvent was removed under reduced pressure by an aspirator, then the pure product was obtained by flash column chromatography on silica gel (eluent: petroleum ether/ethyl acetate = 10:1). Full experimental details can be found in the Supplementary Methods.

## Supplementary information

Supplementary Information

## Data Availability

The authors declare that the data supporting the findings of this study are available within the article and its supplementary information files and from the authors upon reasonable request.
