# Supplementary material for: Electrochemical oxidation induced intermolecular aromatic C-H imidation
Source: Nat Commun. 2019 Nov 29;10:5467. doi: 10.1038/s41467-019-13524-4 (PMC6884519; doi:10.1038/s41467-019-13524-4)
Supplement: Supplementary file 1 — Supplementary Information [file 41467_2019_13524_MOESM1_ESM.pdf]

Supplementary Information

Electrochemical Oxidation Induced Intermolecular Aromatic C-H Imidation

Hu et al.

Supplementary Figures

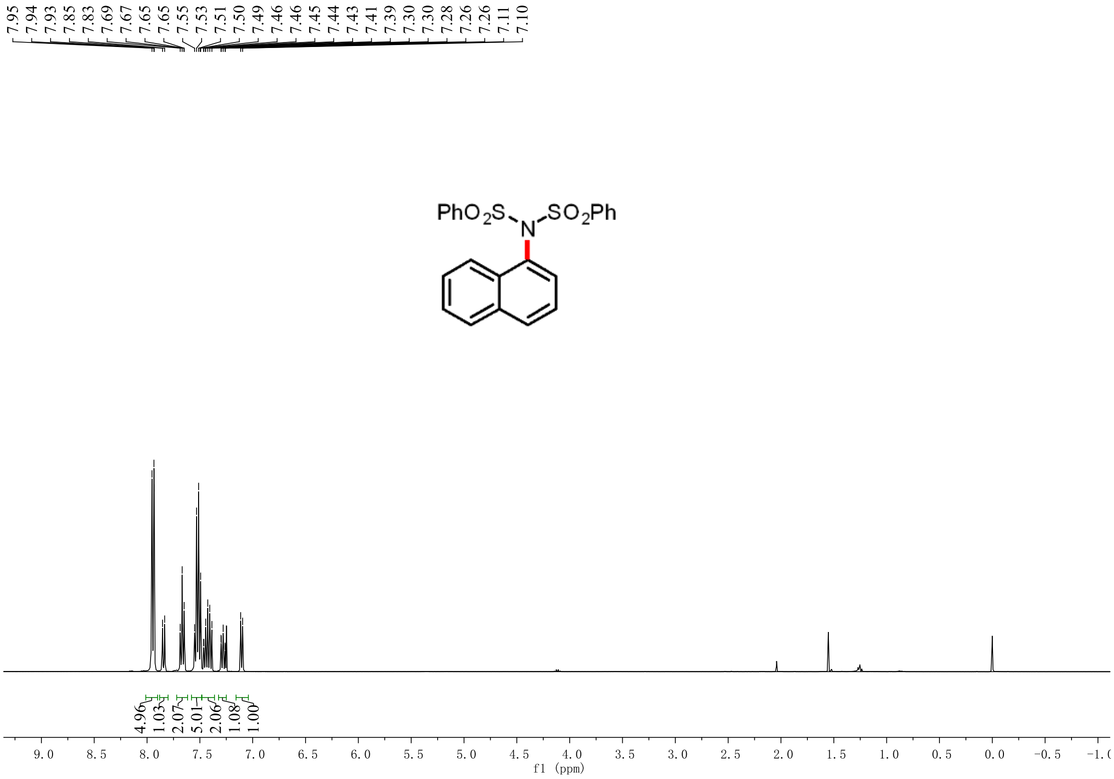

Supplementary Figure 1. ^{1}H NMR (400 MHz, $CDCl_3$) spectrum of **3aa**

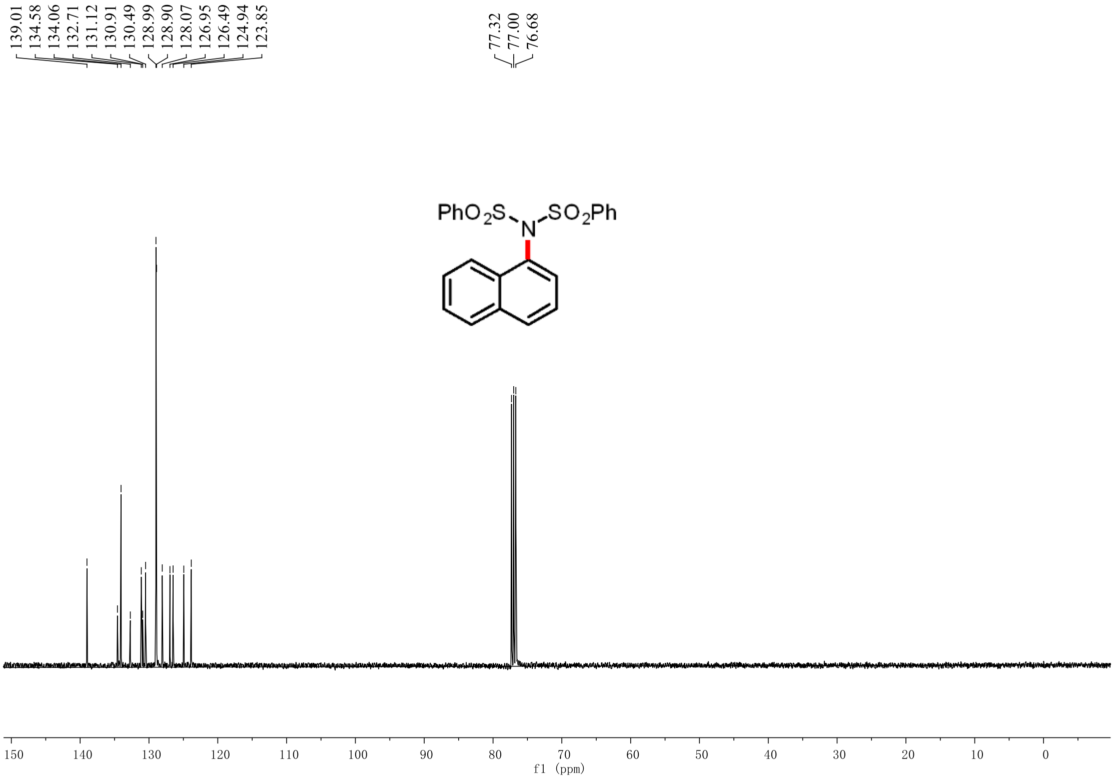

Supplementary Figure 2. ^{13}C NMR (101 MHz, $CDCl_3$) spectrum of **3aa**

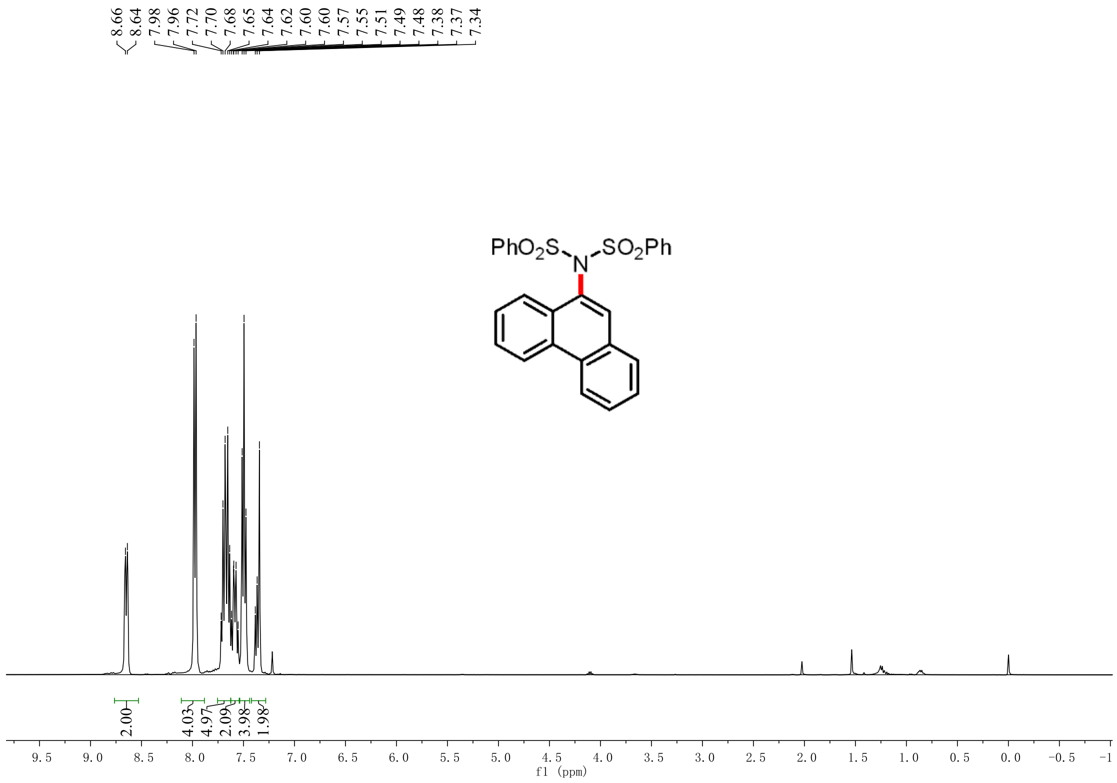

Supplementary Figure 3. ^{1}H NMR (400 MHz, $CDCl_3$) spectrum of **3ab**

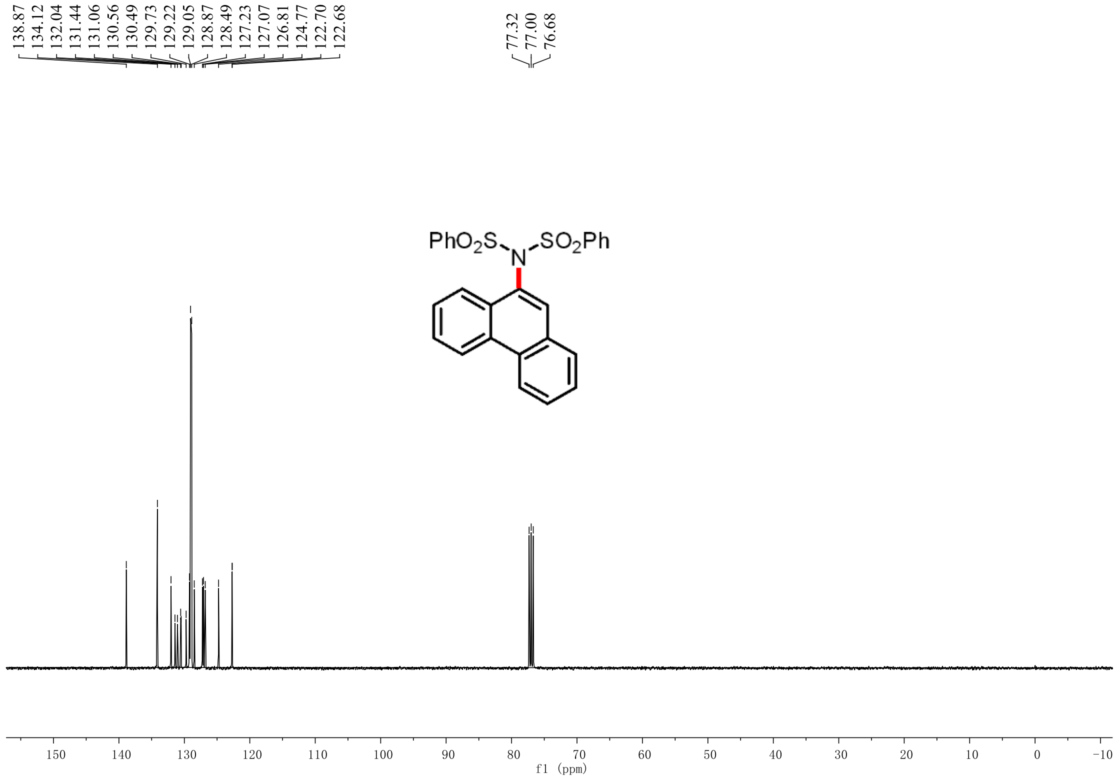

Supplementary Figure 4. ^{13}C NMR (101 MHz, $CDCl_3$) spectrum of **3aa**

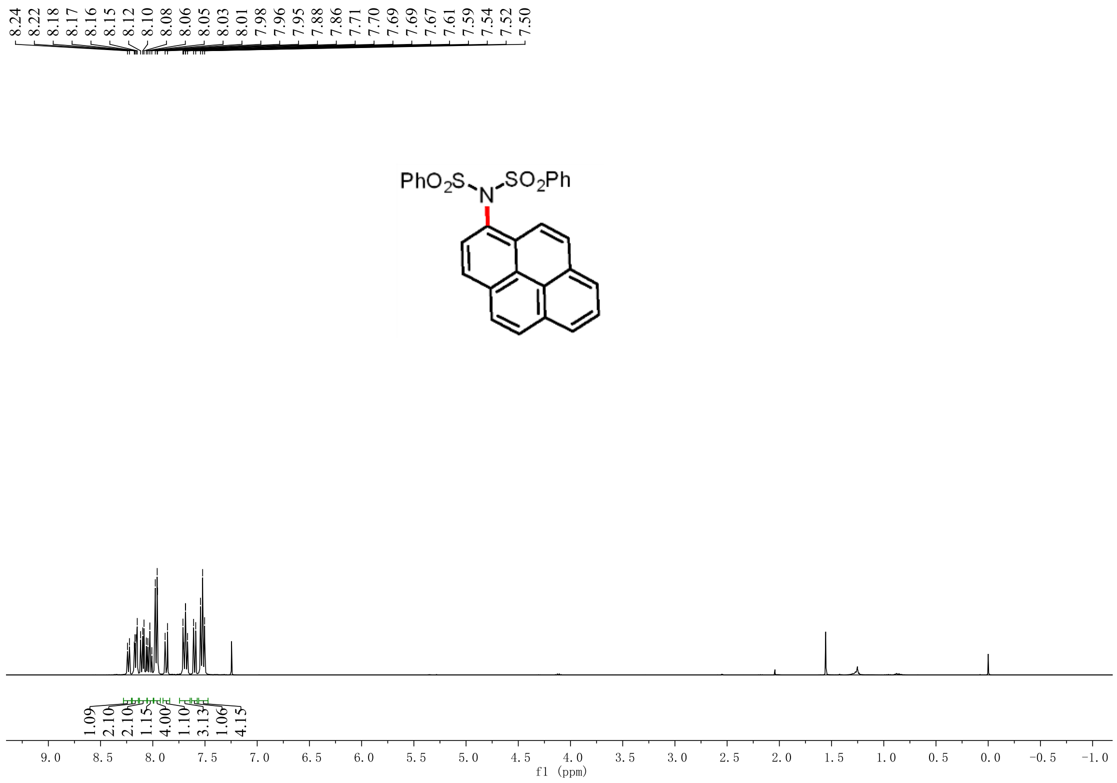

Supplementary Figure 5. ^{1}H NMR (400 MHz, $CDCl_3$) spectrum of **3ac**

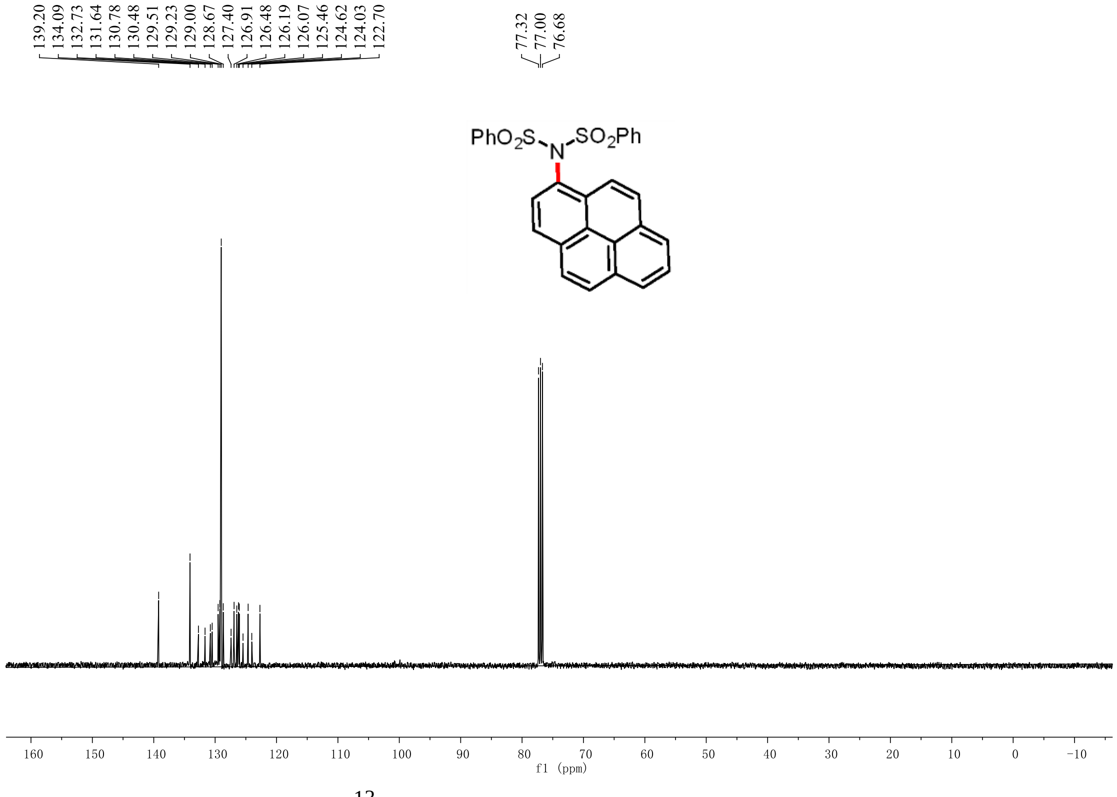

Supplementary Figure 6. ^{13}C NMR (101 MHz, $CDCl_3$) spectrum of **3ac**

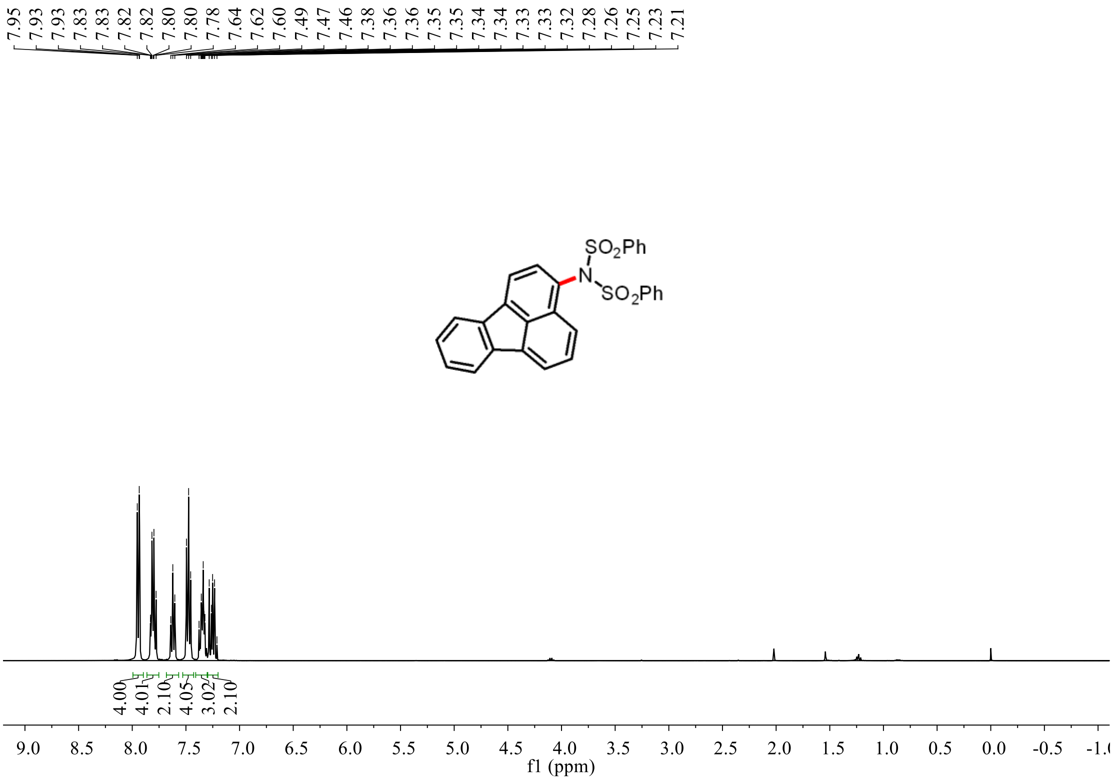

Supplementary Figure 7. ^{1}H NMR (400 MHz, $CDCl_3$) spectrum of **3ad**

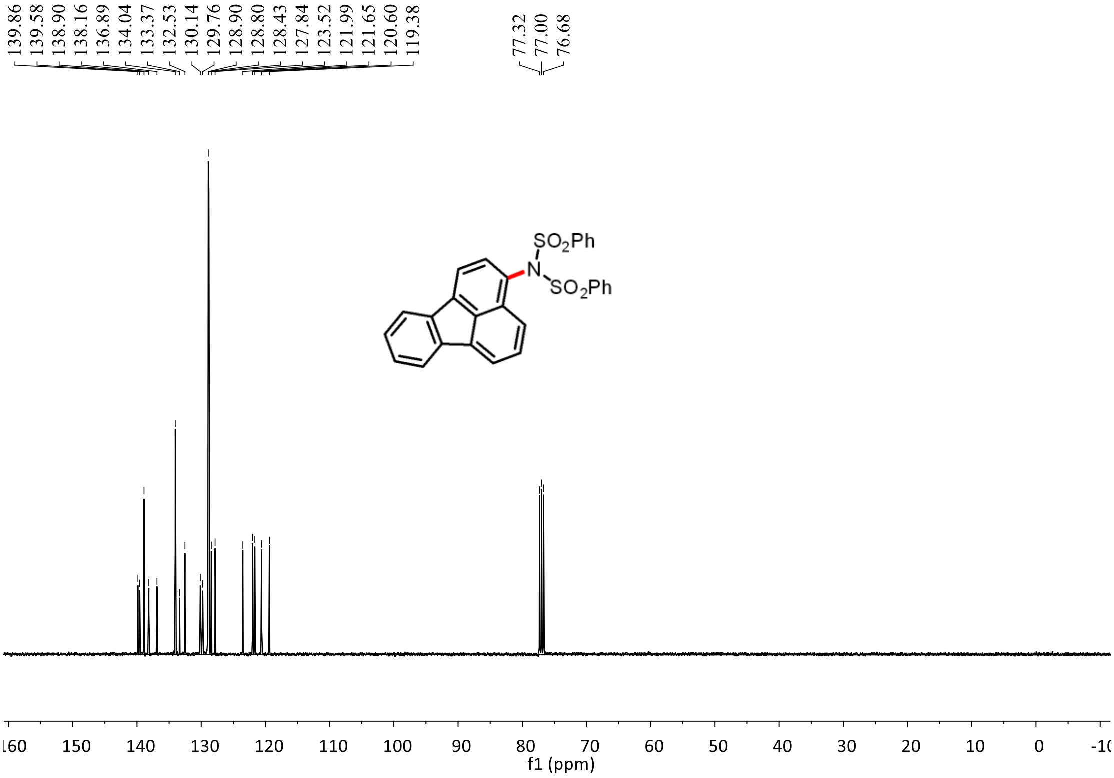

Supplementary Figure 8. ^{13}C NMR (101 MHz, $CDCl_3$) spectrum of **3ad**

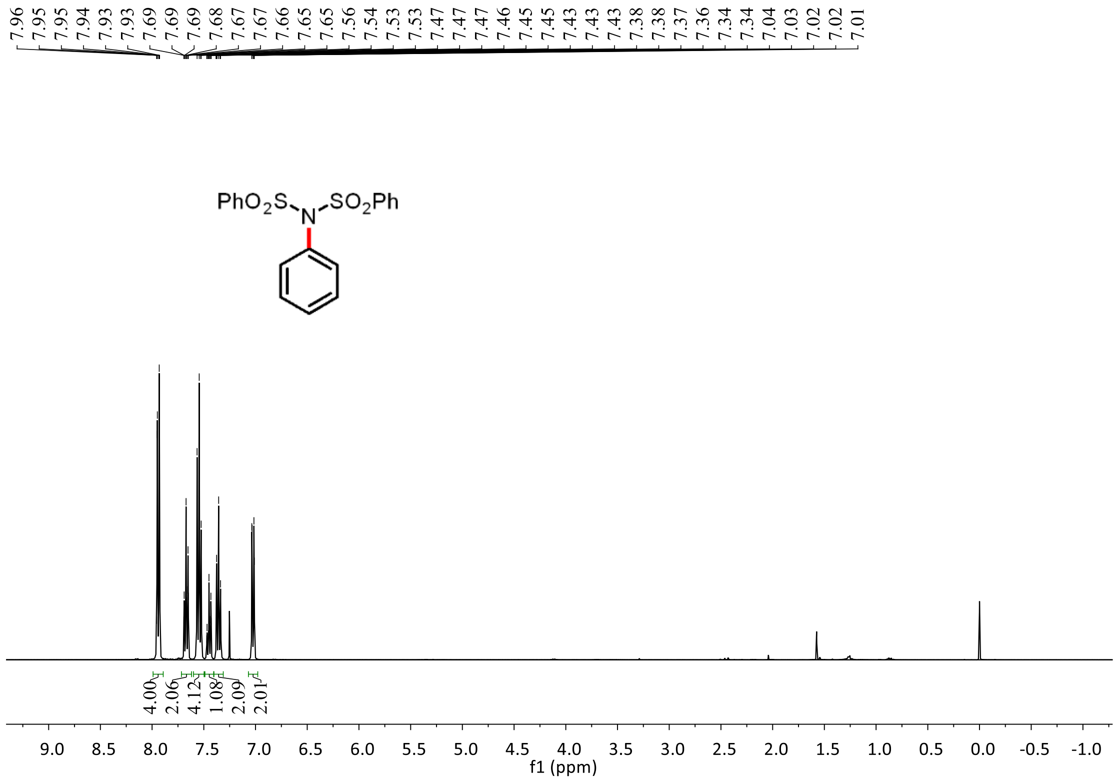

Supplementary Figure 9. ^{1}H NMR (400 MHz, $CDCl_3$) spectrum of **3ae**

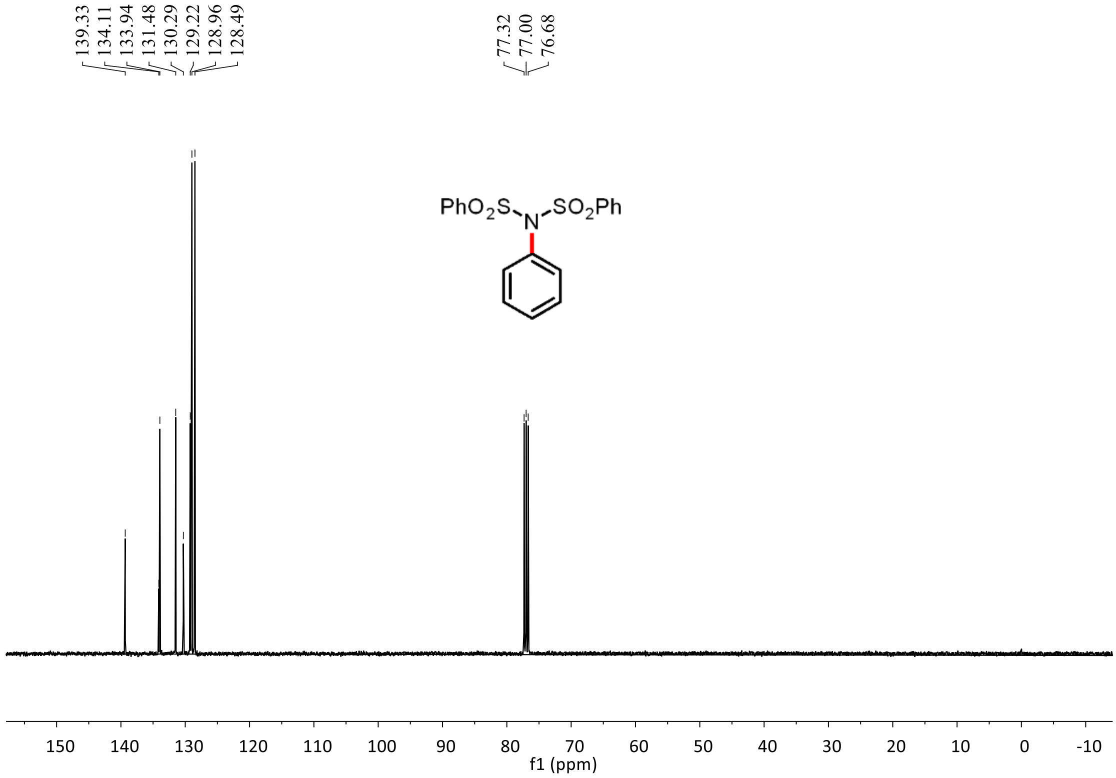

Supplementary Figure 10. ^{13}C NMR (101 MHz, $CDCl_3$) spectrum of **3ae**

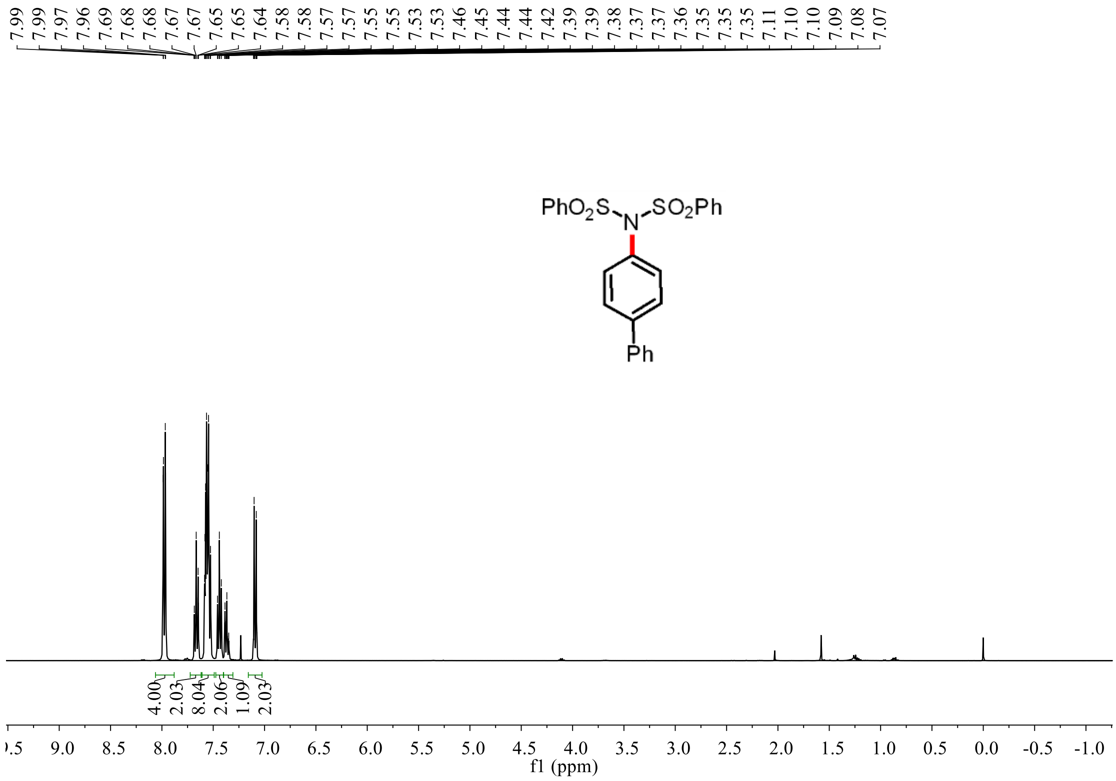

Supplementary Figure 11. ^{1}H NMR (400 MHz, $CDCl_3$) spectrum of **3af**

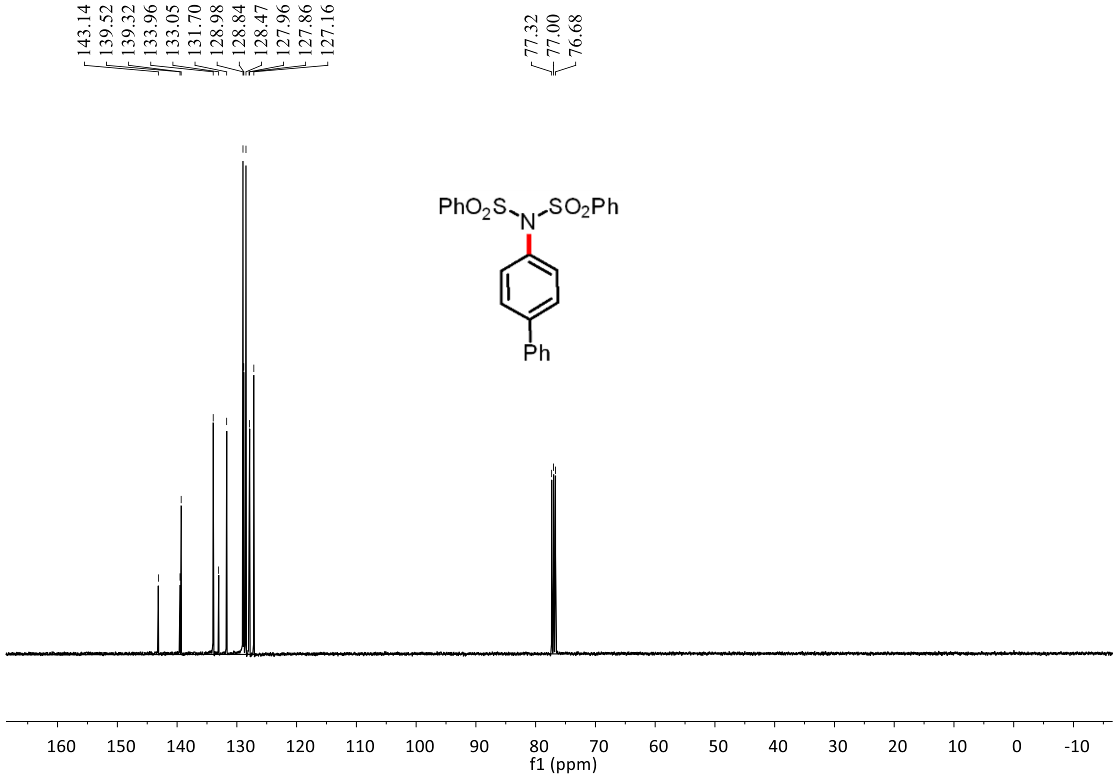

Supplementary Figure 12. ^{13}C NMR (101 MHz, $CDCl_3$) spectrum of **3af**

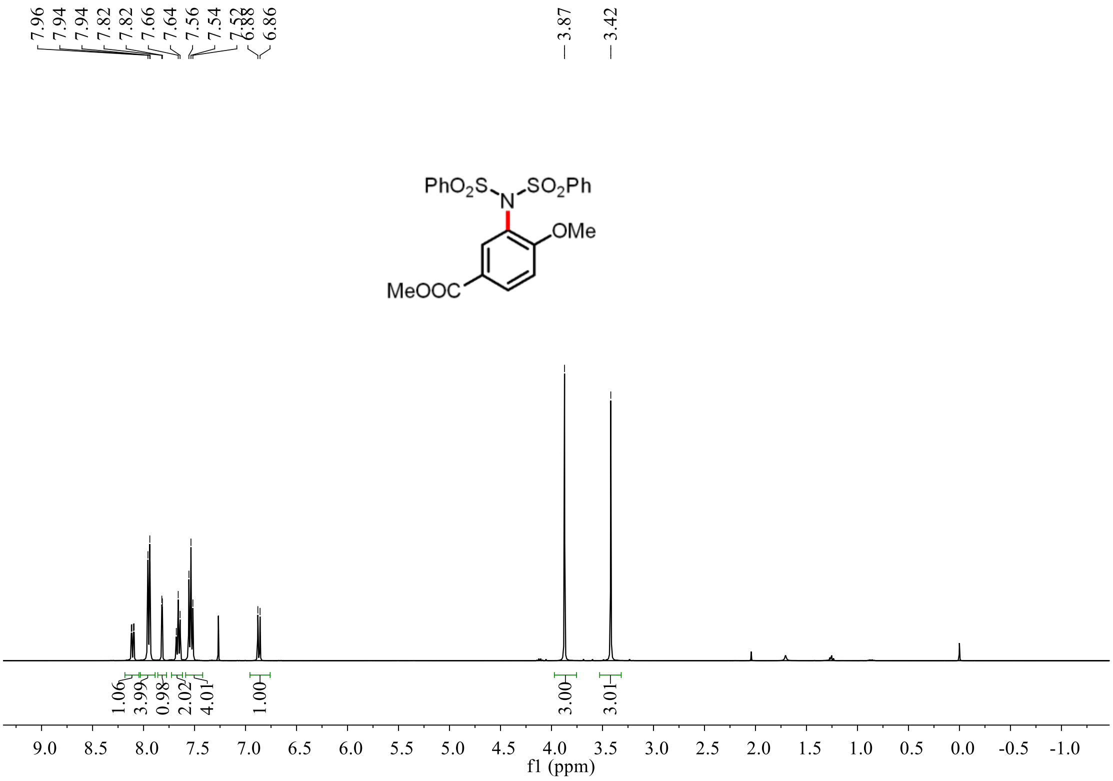

Supplementary Figure 13. ^{1}H NMR (400 MHz, $CDCl_3$) spectrum of **3ag**

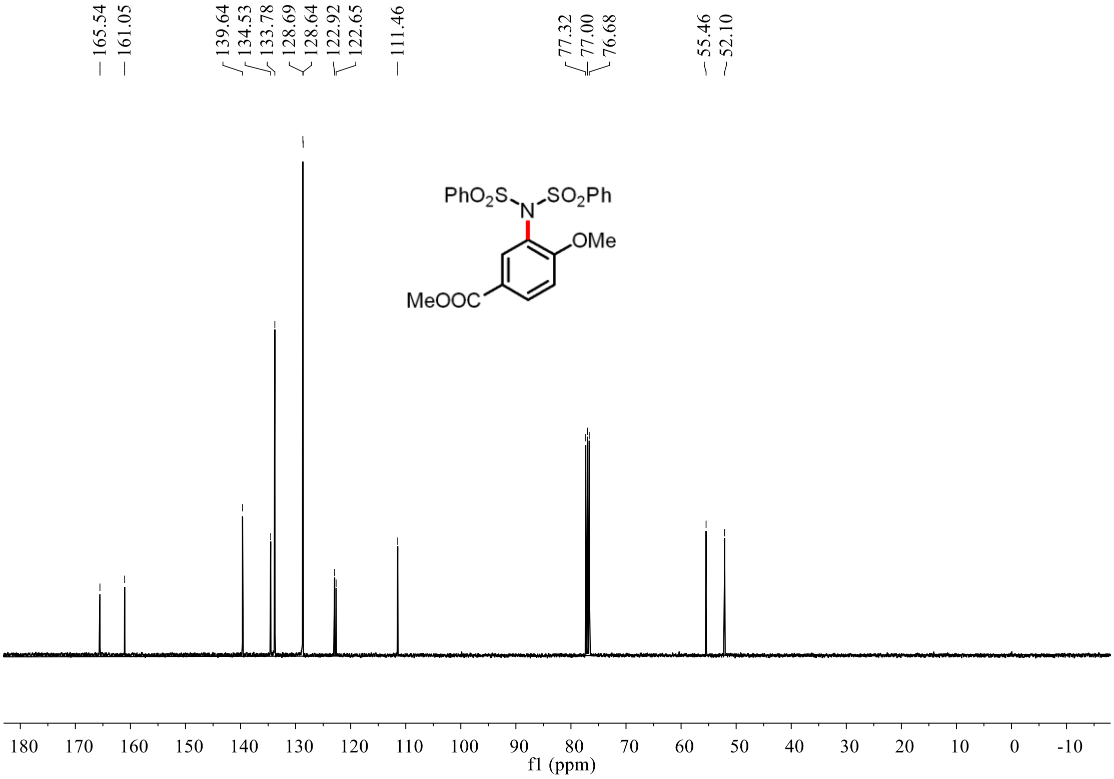

Supplementary Figure 14. ^{13}C NMR (101 MHz, $CDCl_3$) spectrum of **3ag**

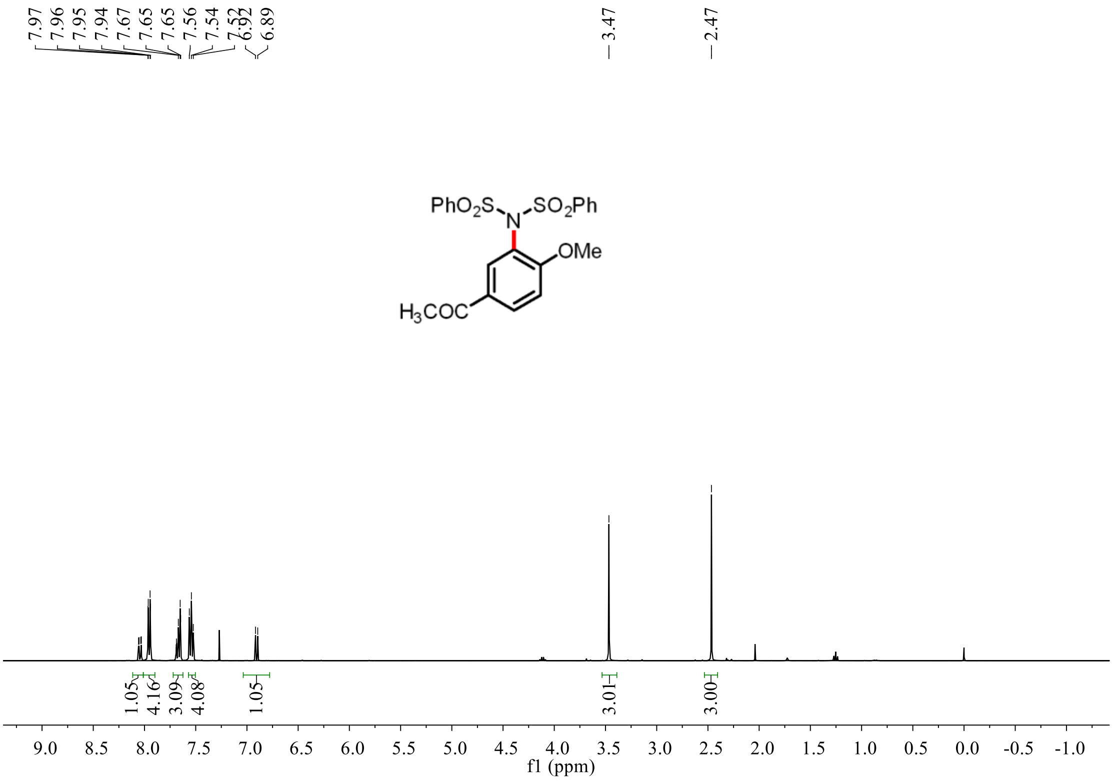

Supplementary Figure 15. ^{1}H NMR (400 MHz, $CDCl_3$) spectrum of **3ah**

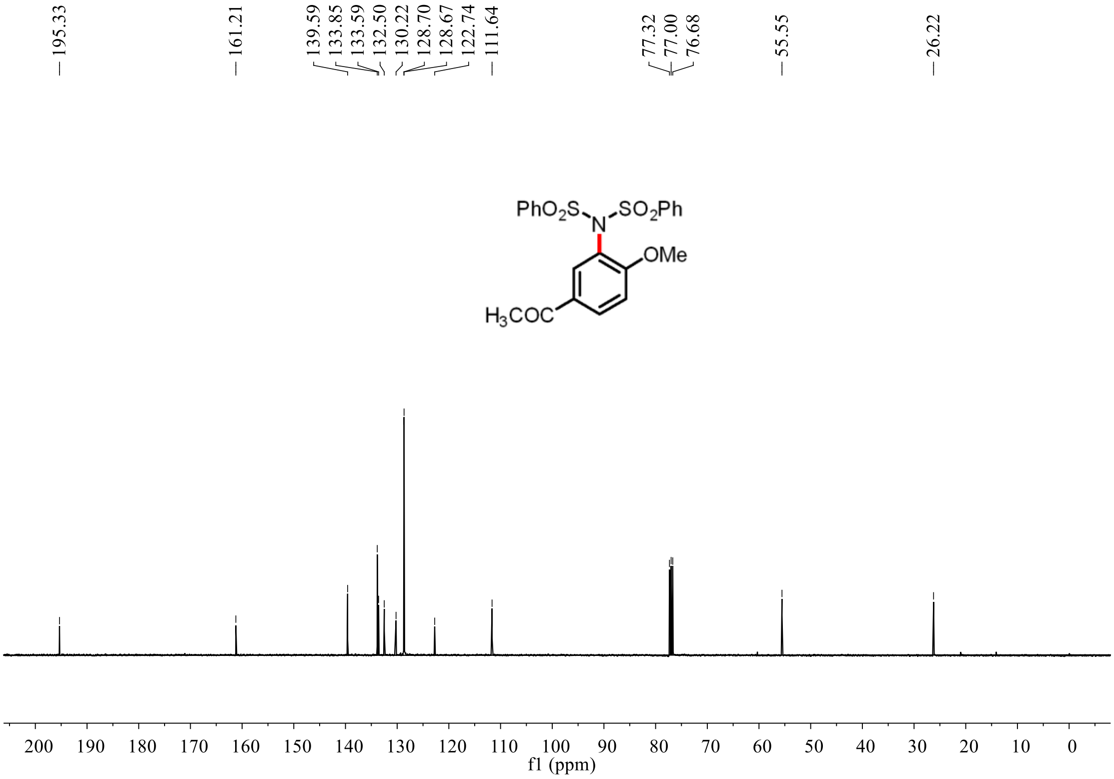

Supplementary Figure 16. ^{13}C NMR (101MHz, $CDCl_3$) spectrum of **3ah**

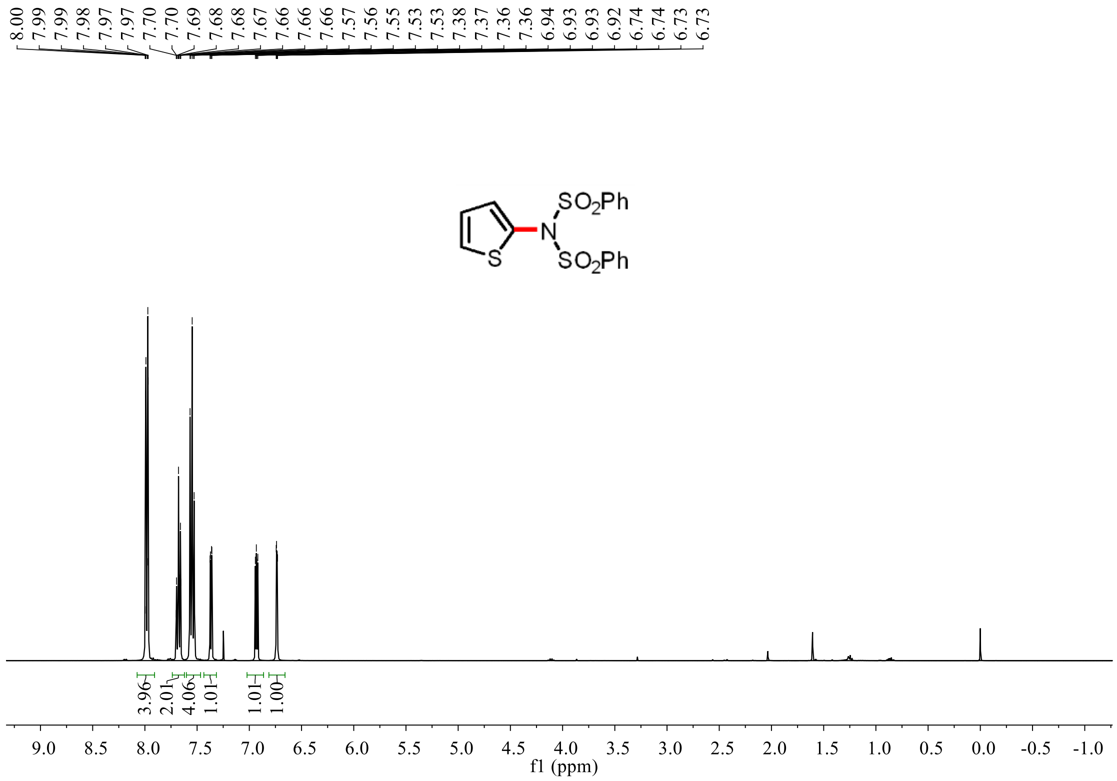

Supplementary Figure 17. ^{1}H NMR (400 MHz, $CDCl_3$) spectrum of **3ai**

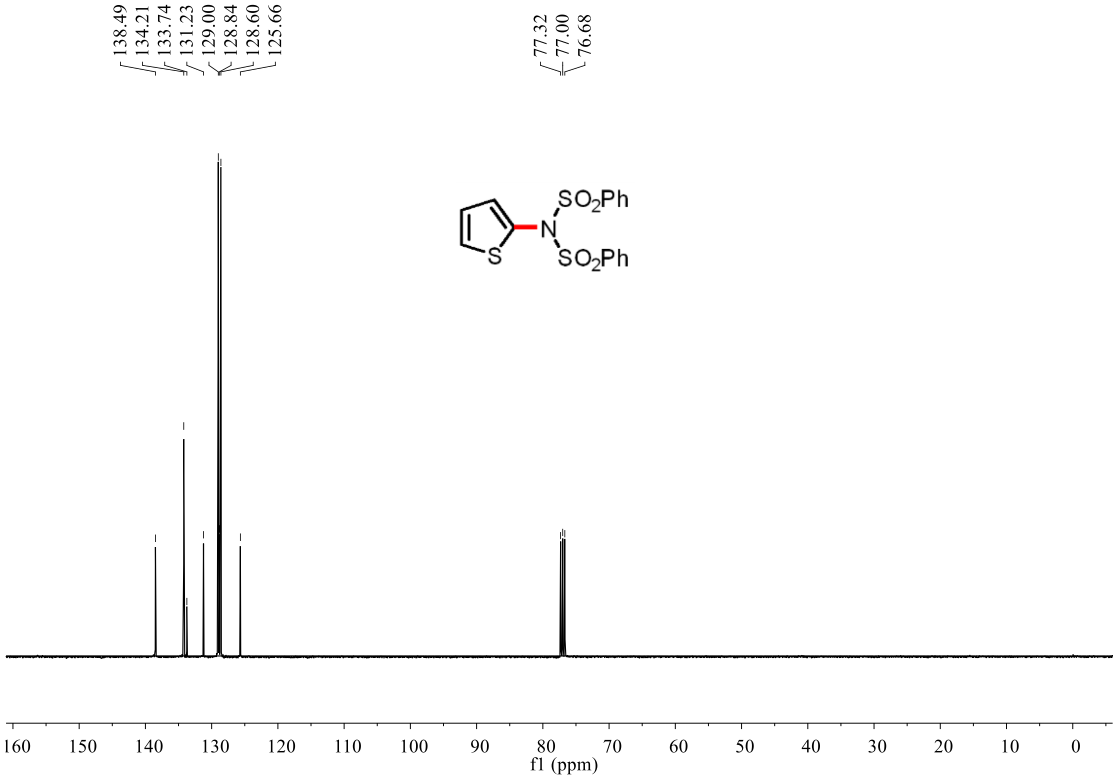

Supplementary Figure 18. ^{13}C NMR (101 MHz, $CDCl_3$) spectrum of **3ai**

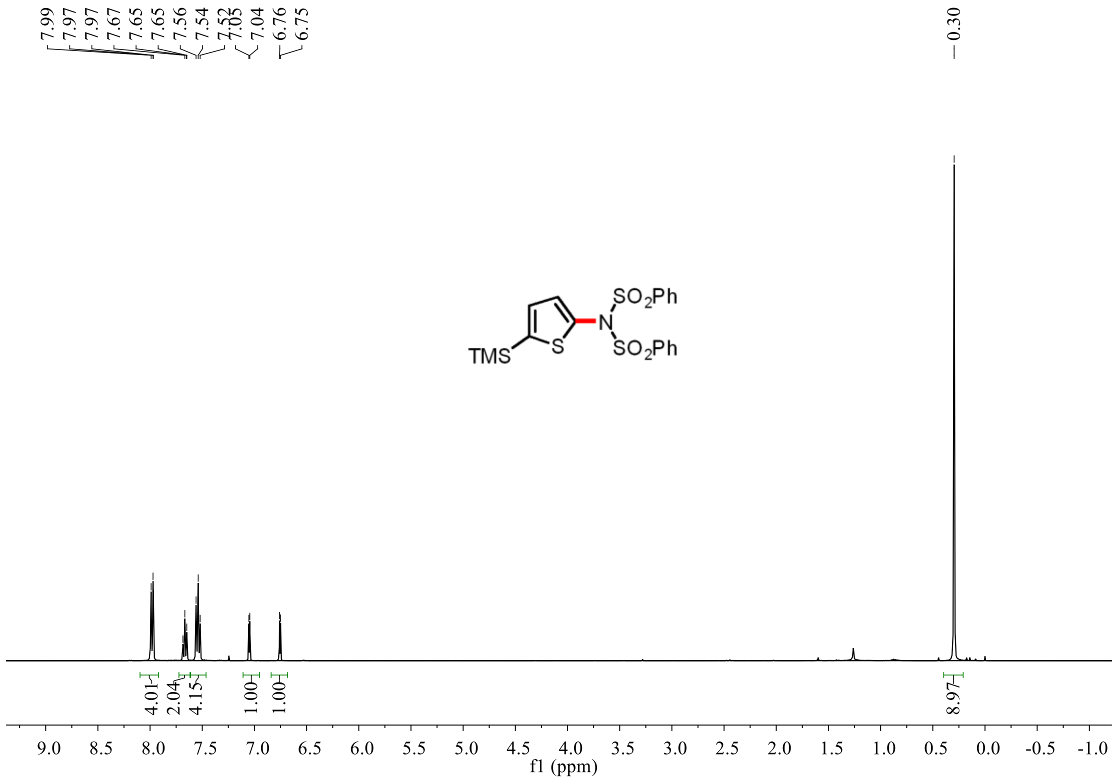

Supplementary Figure 19. ^{1}H NMR (400 MHz, $CDCl_3$) spectrum of **3aj**

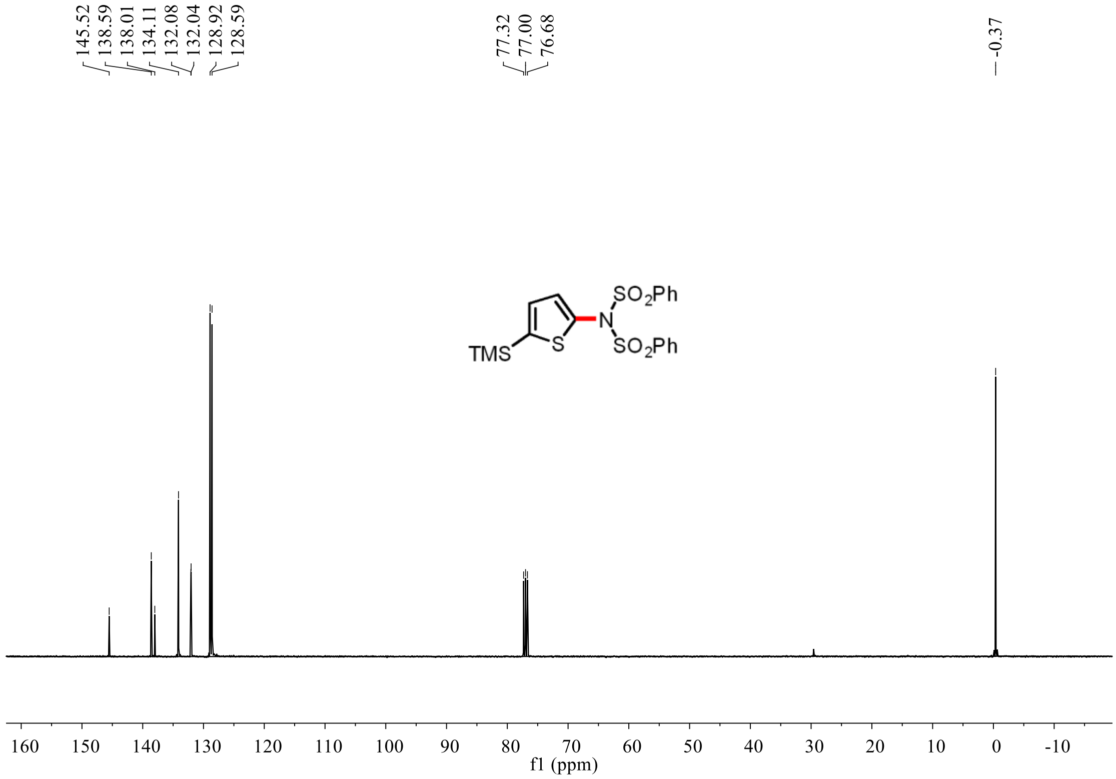

Supplementary Figure 20. ^{13}C NMR (101 MHz, $CDCl_3$) spectrum of **3aj**

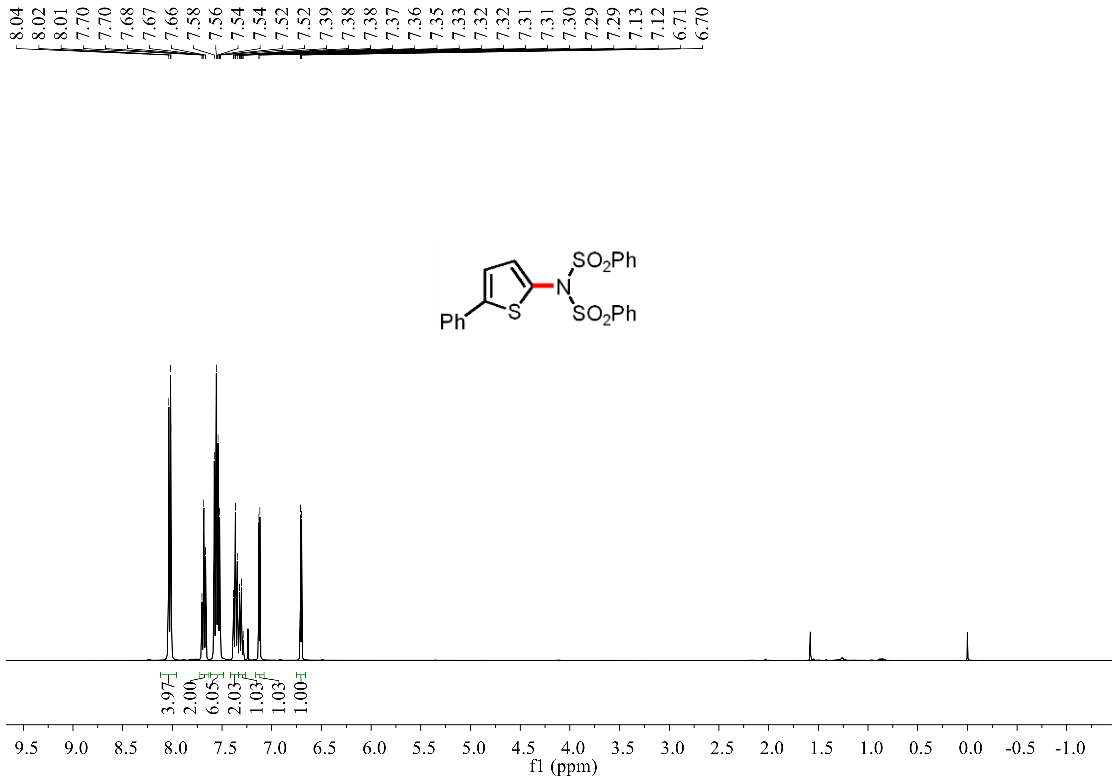

Supplementary Figure 21. ^{1}H NMR (400 MHz, $CDCl_3$) spectrum of **3ak**

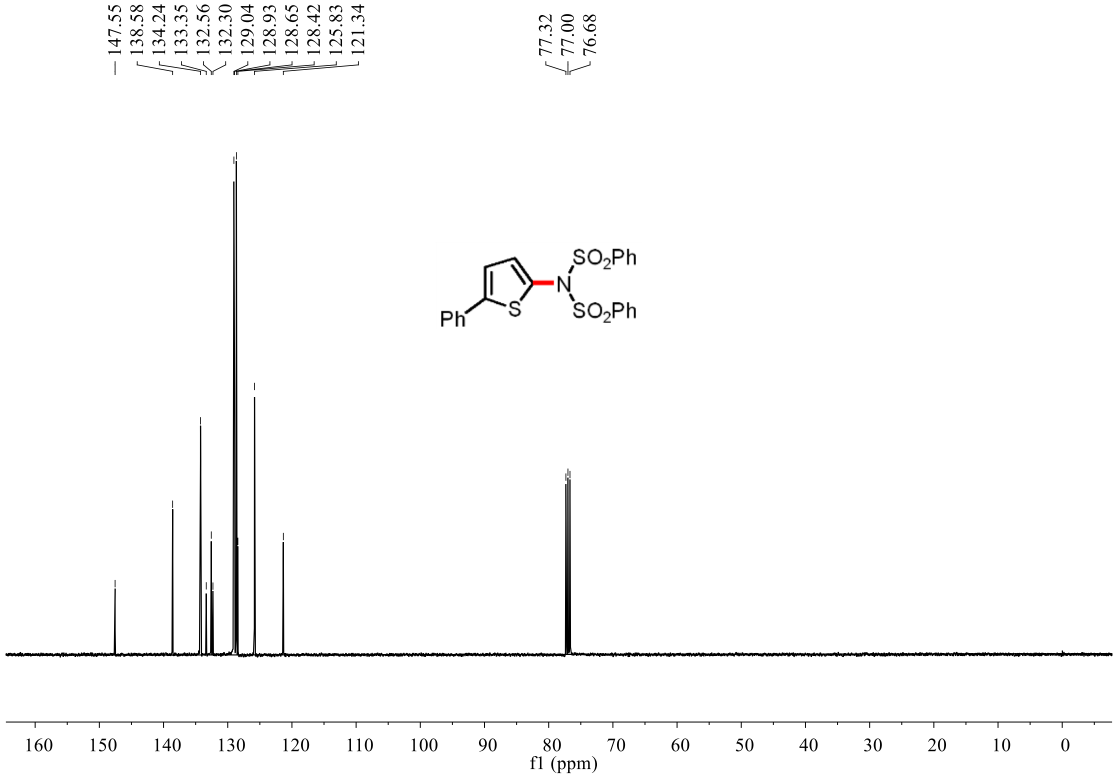

Supplementary Figure 22. ^{13}C NMR (101 MHz, $CDCl_3$) spectrum of **3ak**

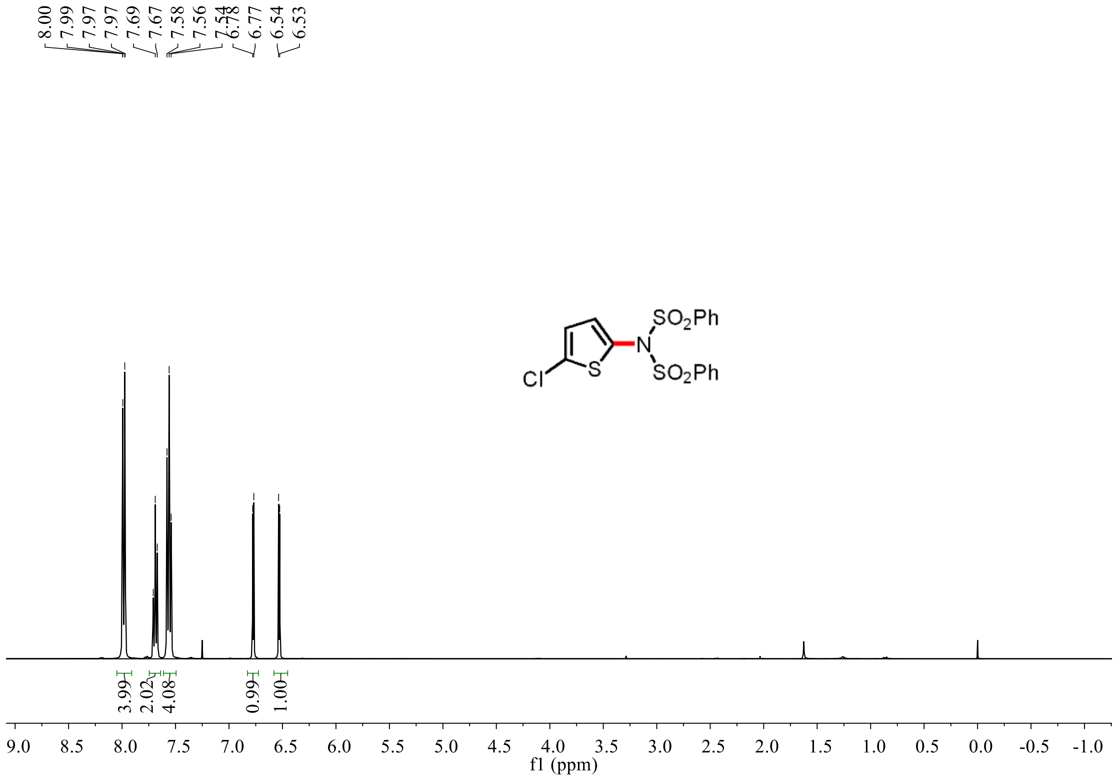

Supplementary Figure 23. ^{1}H NMR (400 MHz, $CDCl_3$) spectrum of **3al**

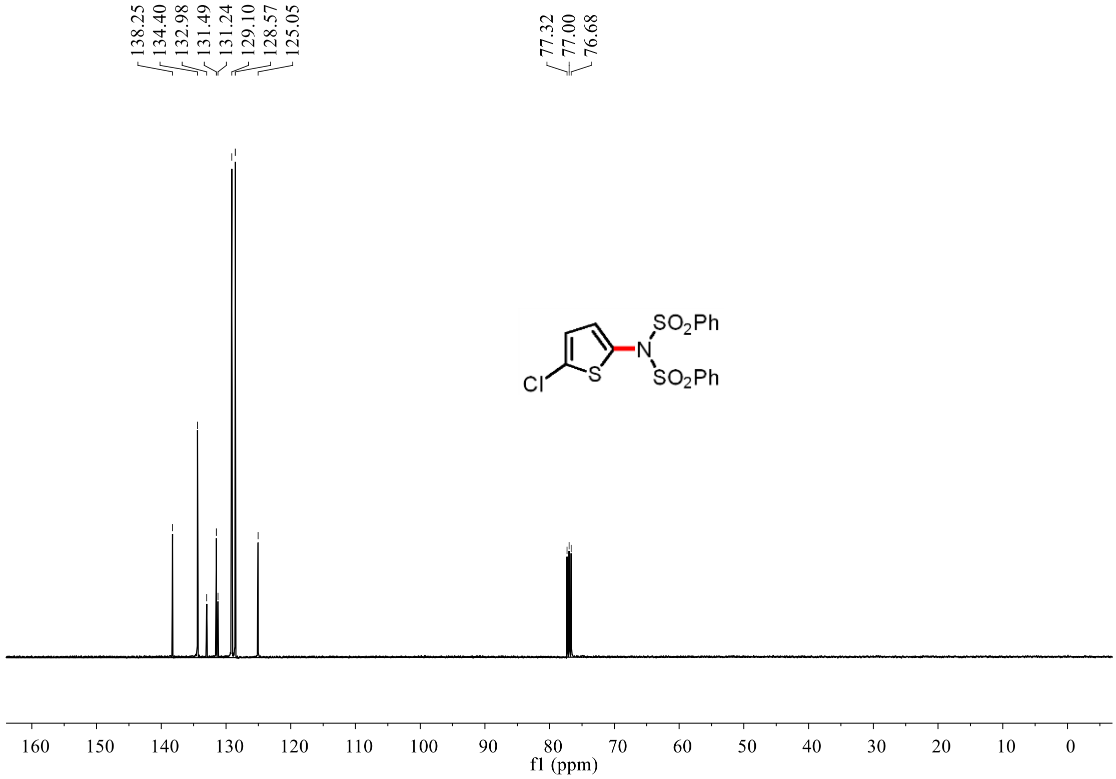

Supplementary Figure 24. ^{13}C NMR (101 MHz, $CDCl_3$) spectrum of **3al**

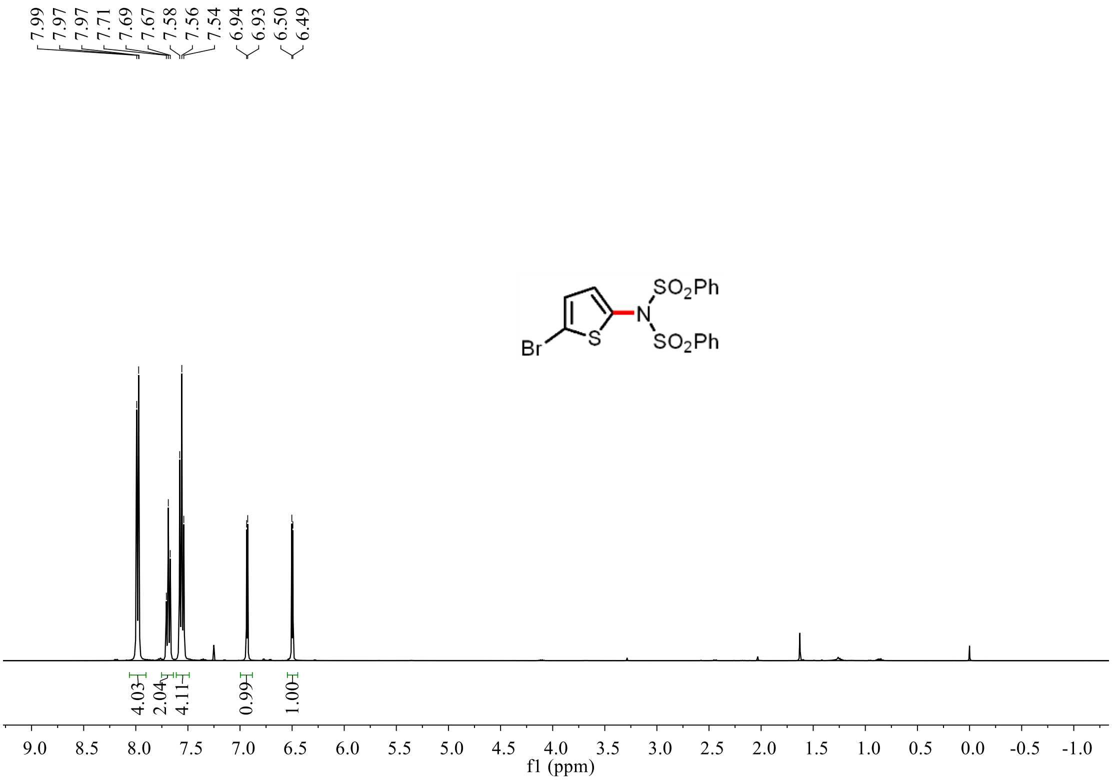

Supplementary Figure 25. ^{1}H NMR (400 MHz, $CDCl_3$) spectrum of **3am**

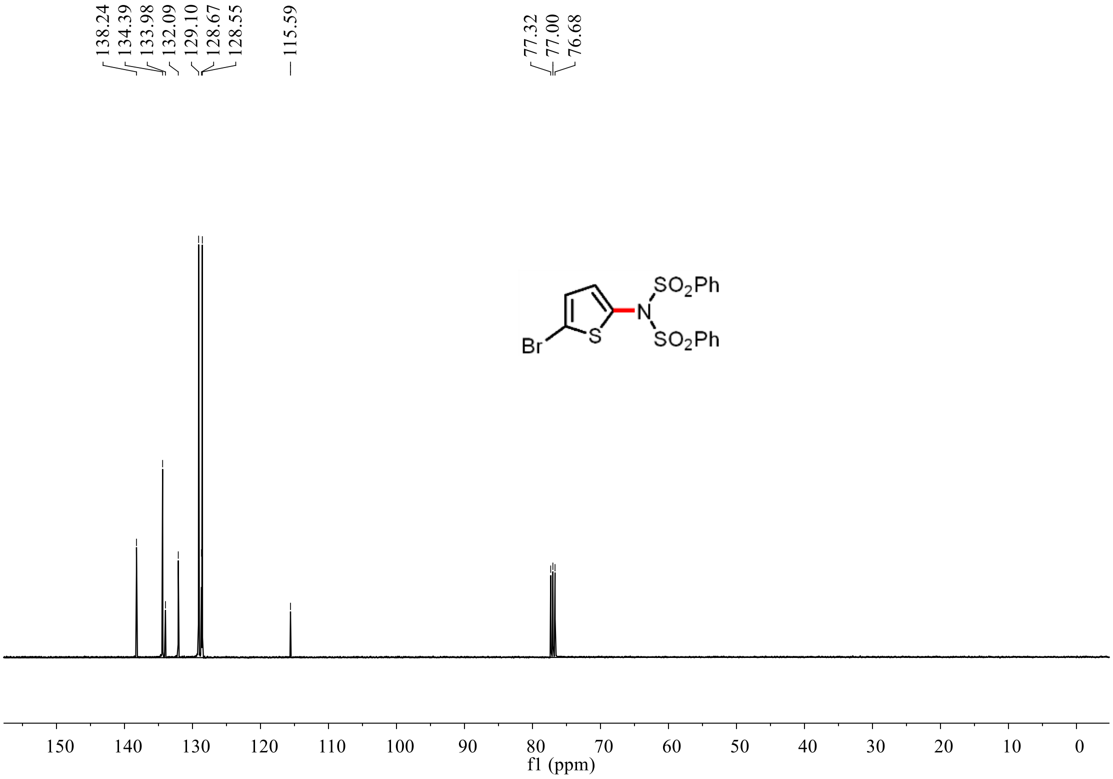

Supplementary Figure 26. ^{13}C NMR (101 MHz, $CDCl_3$) spectrum of **3am**

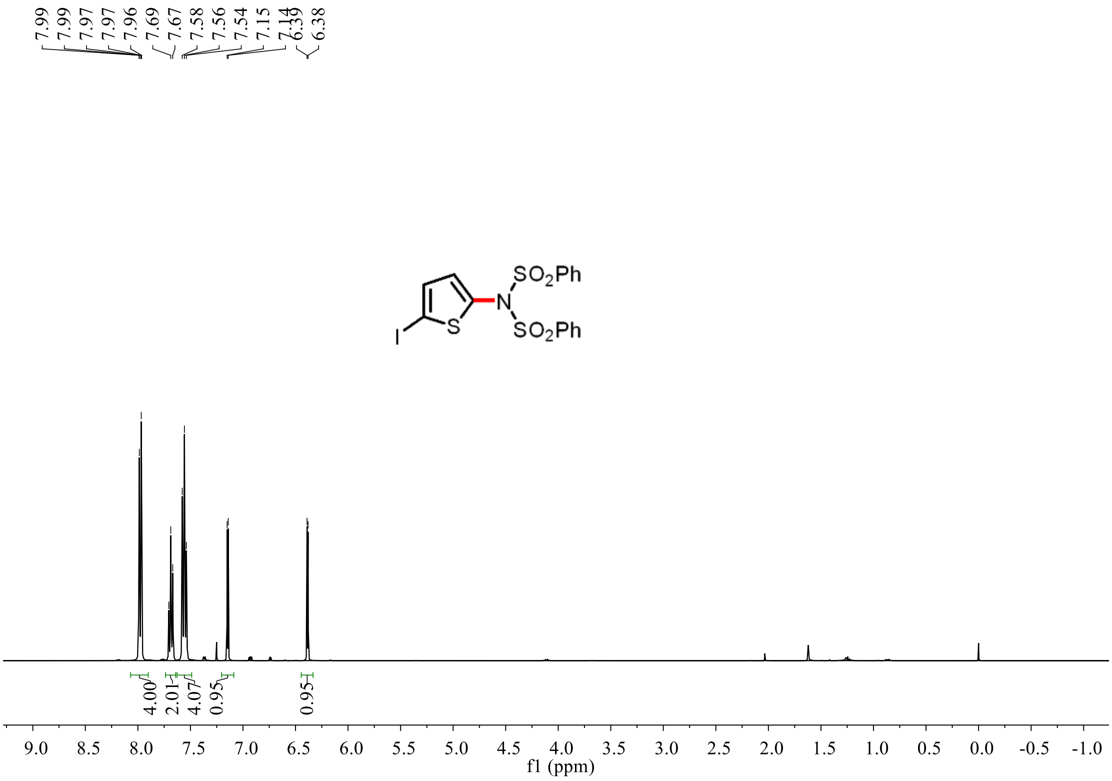

Supplementary Figure 27. ^{1}H NMR (400 MHz, $CDCl_3$) spectrum of **3an**

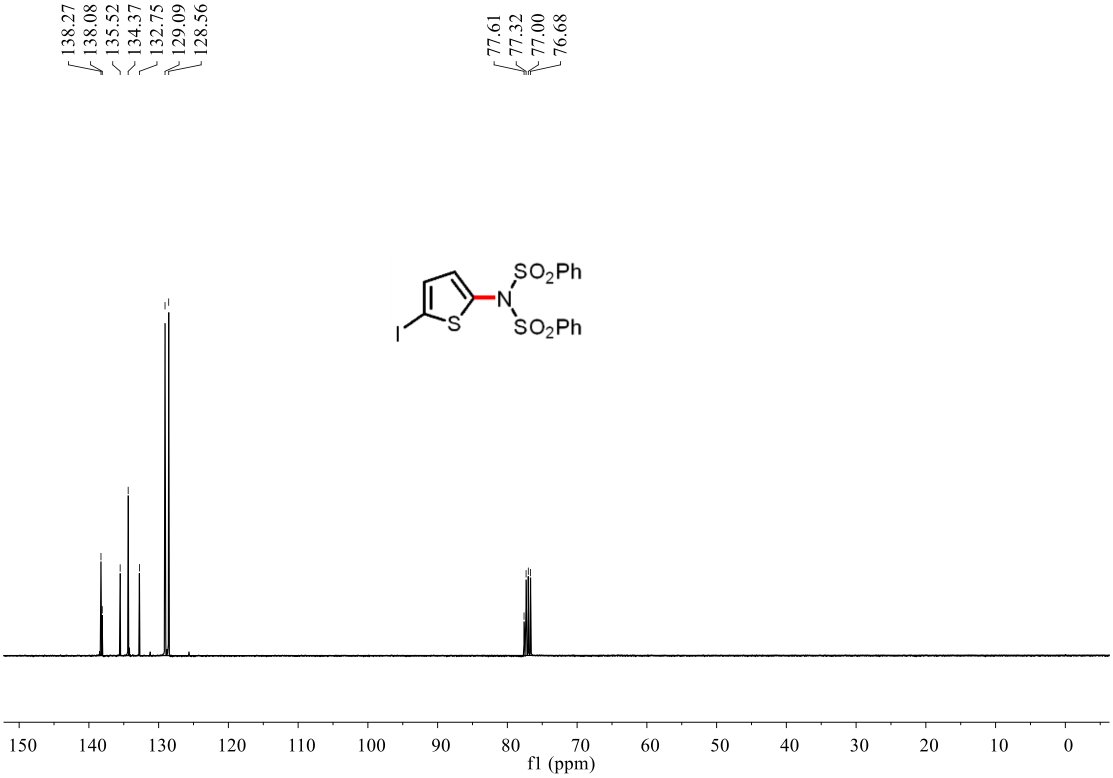

Supplementary Figure 28. ^{13}C NMR (101 MHz, $CDCl_3$) spectrum of **3an**

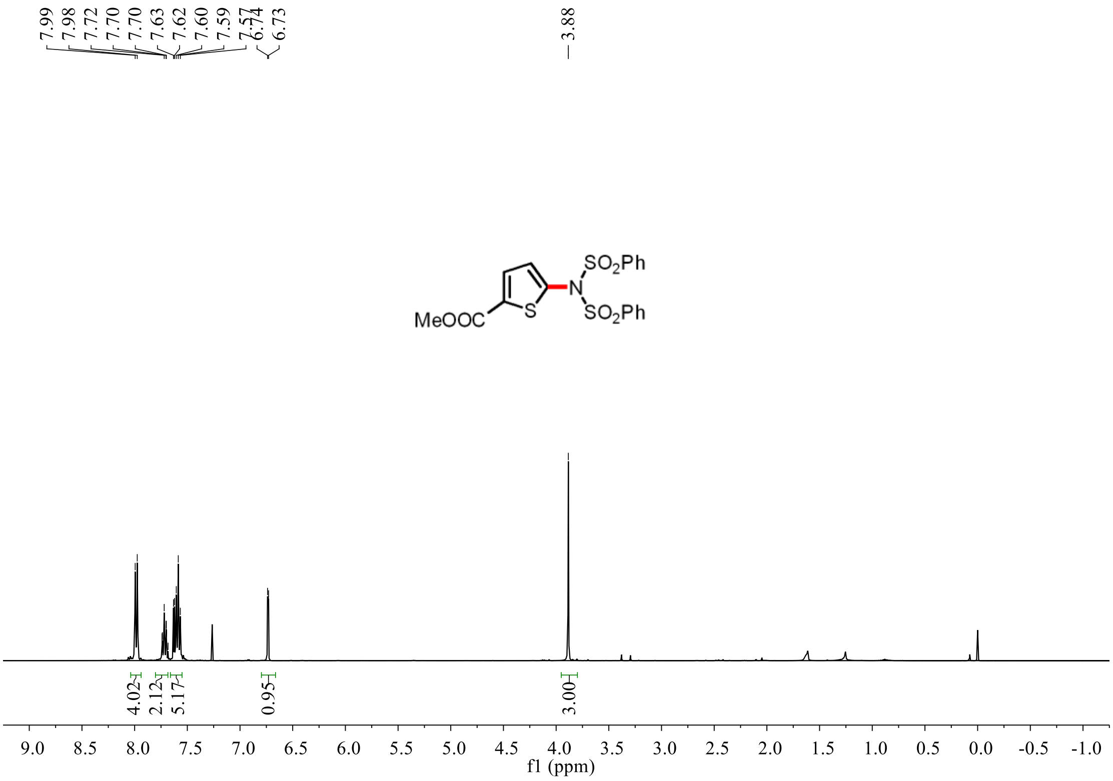

Supplementary Figure 29. ^{1}H NMR (400 MHz, $CDCl_3$) spectrum of **3ao**

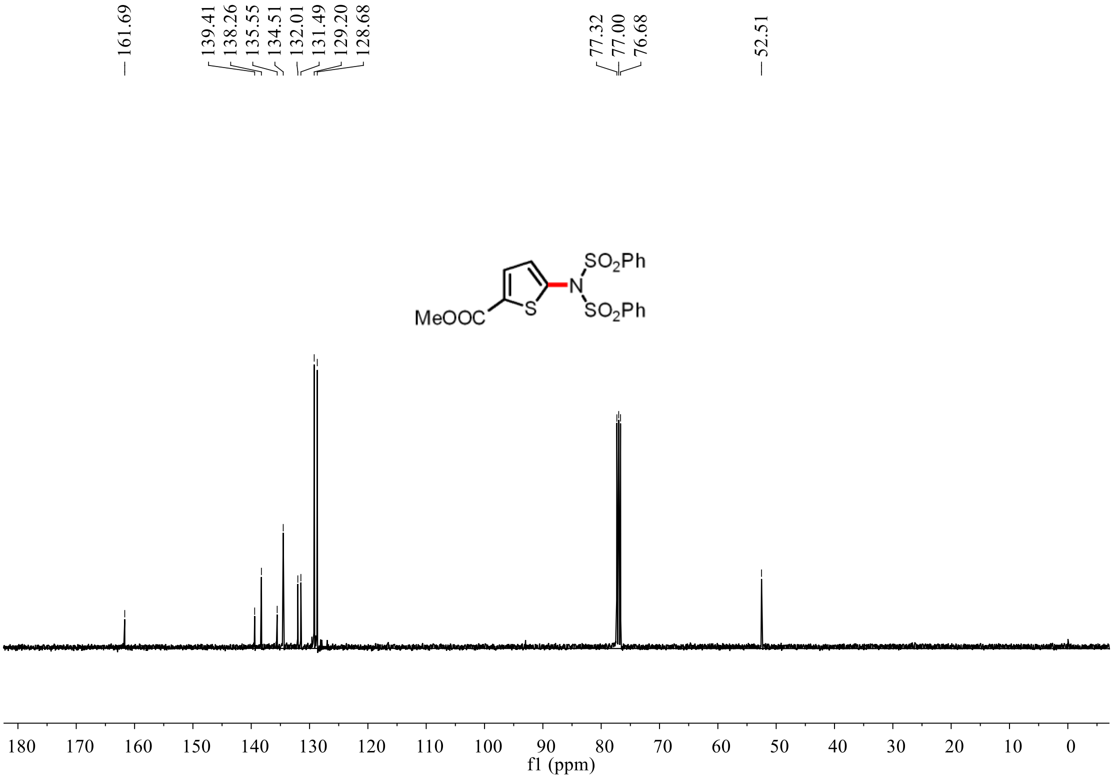

Supplementary Figure 30. ^{13}C NMR (101 MHz, $CDCl_3$) spectrum of **3ao**

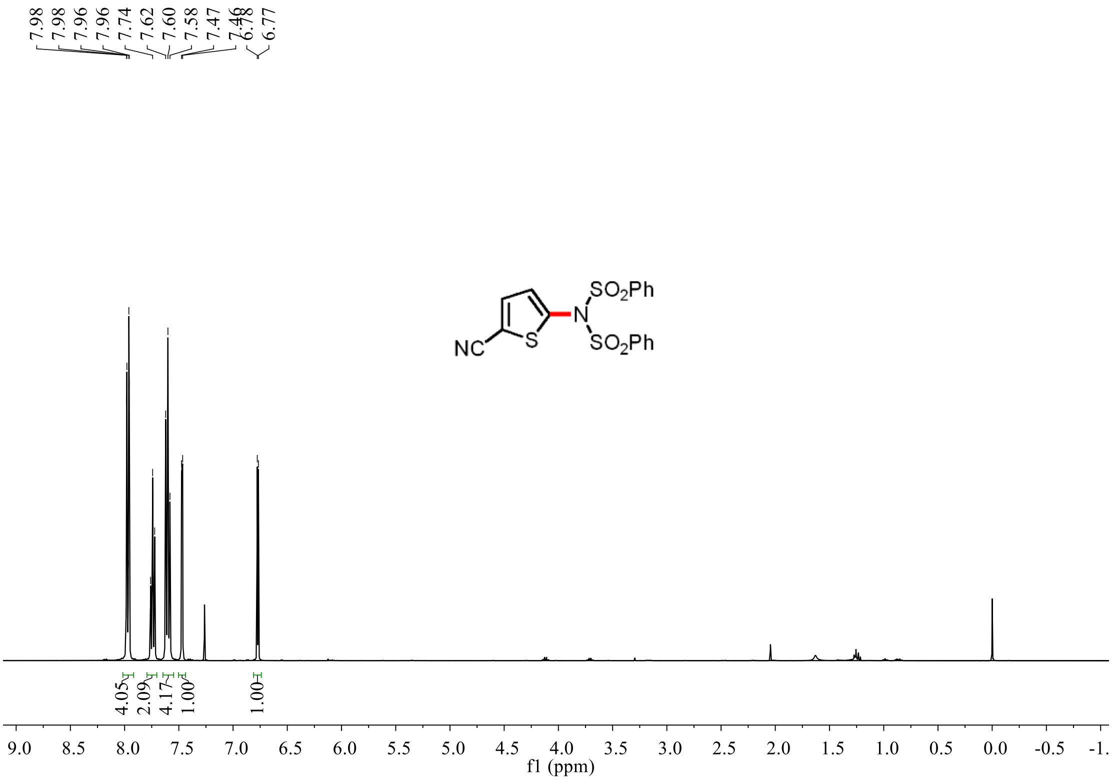

Supplementary Figure 31. ^{1}H NMR (400 MHz, $CDCl_3$) spectrum of **3ap**

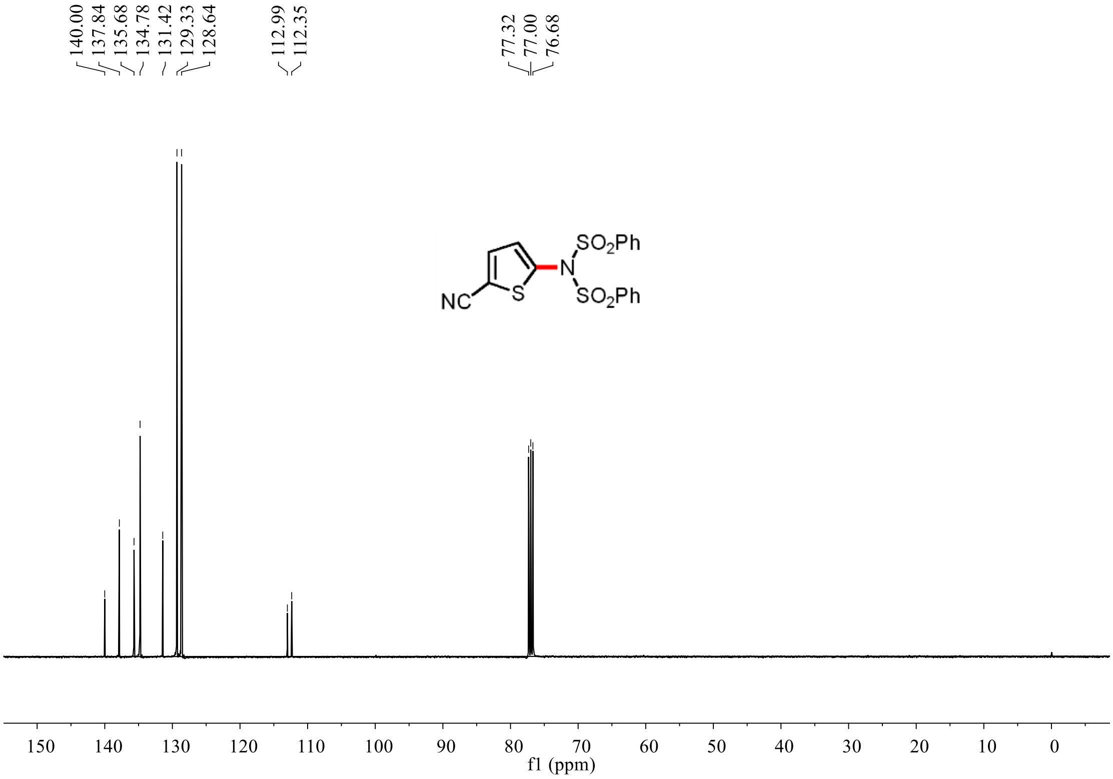

Supplementary Figure 32. ^{13}C NMR (101 MHz, $CDCl_3$) spectrum of **3ap**

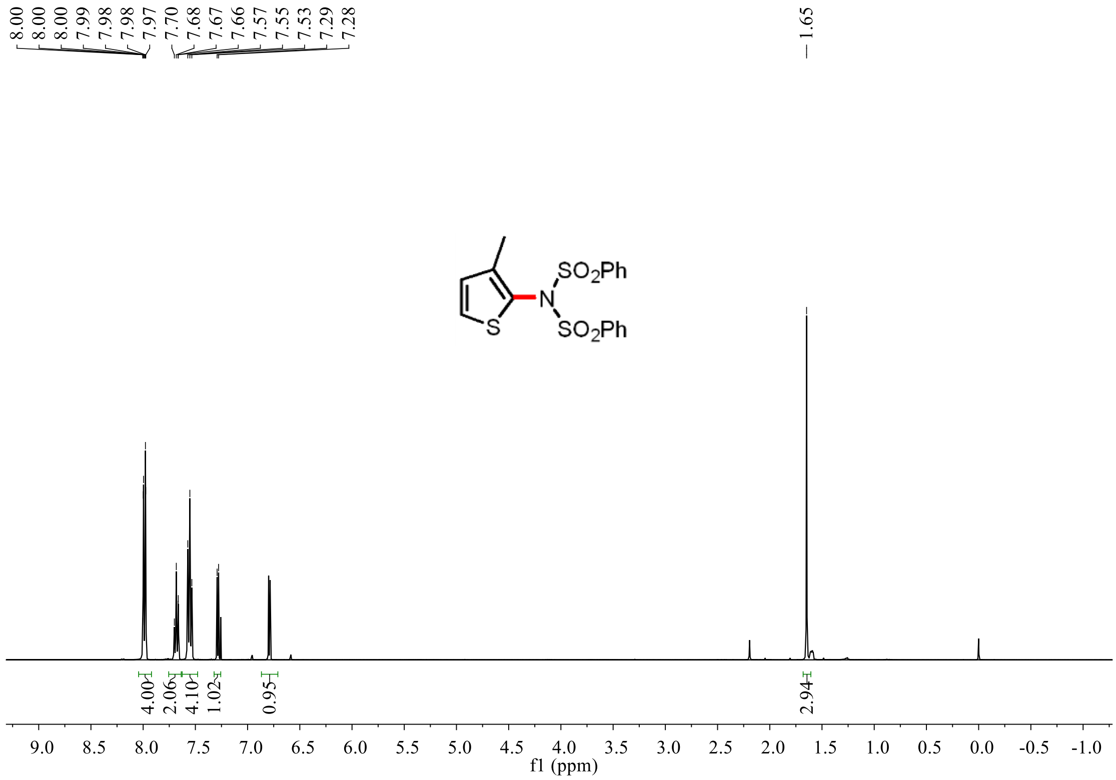

Supplementary Figure 33. ^{1}H NMR (400 MHz, $CDCl_3$) spectrum of **3aq**

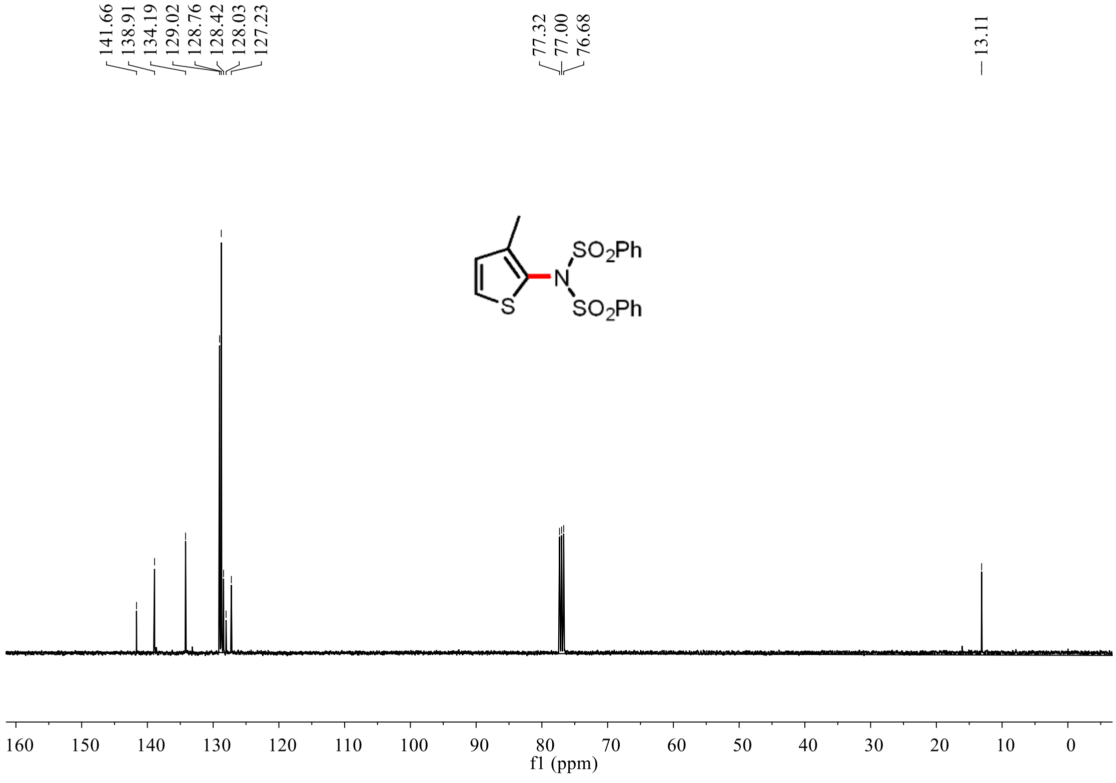

Supplementary Figure 34. ^{13}C NMR (101 MHz, $CDCl_3$) spectrum of **3aq**

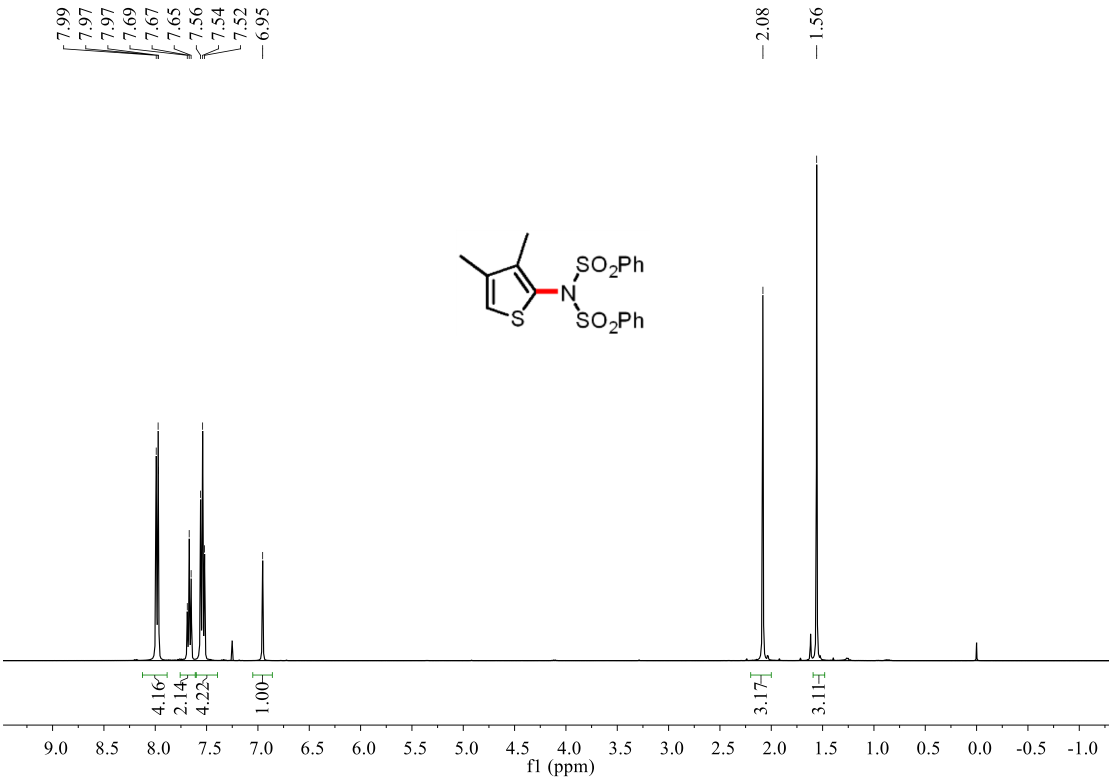

Supplementary Figure 35. ^{1}H NMR (400 MHz, $CDCl_3$) spectrum of **3ar**

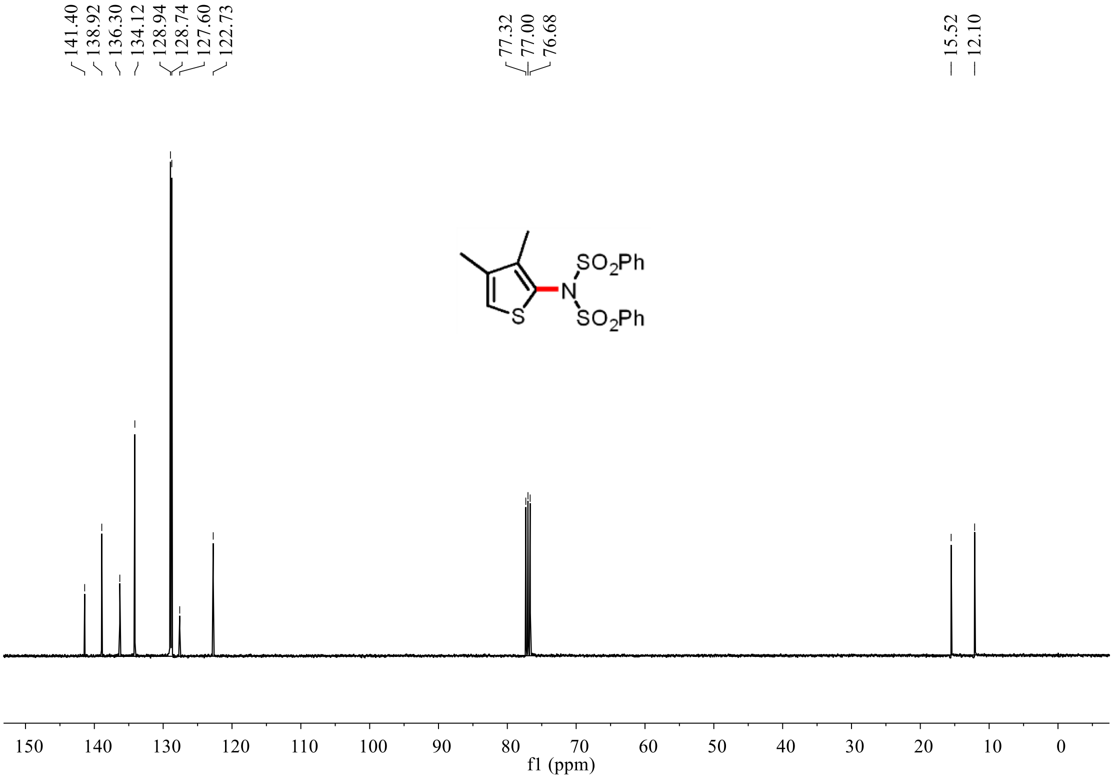

Supplementary Figure 36. ^{13}C NMR (101 MHz, $CDCl_3$) spectrum of **3ar**

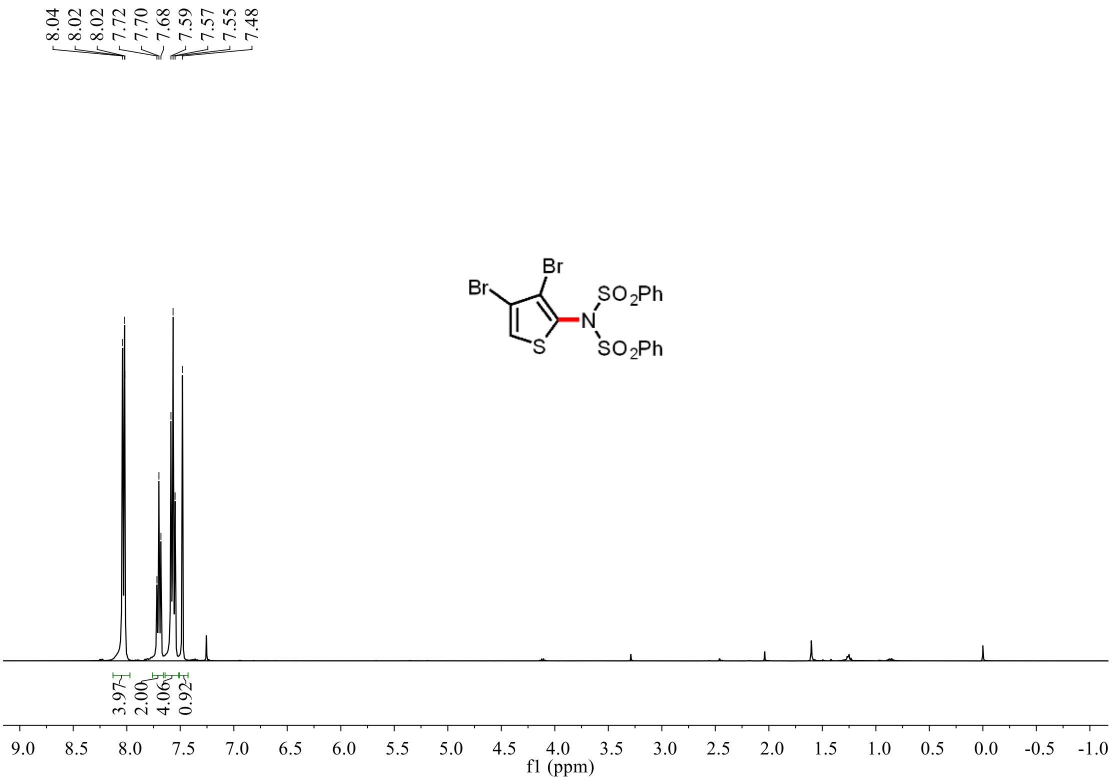

Supplementary Figure 37. ^{1}H NMR (400 MHz, $CDCl_3$) spectrum of **3as**

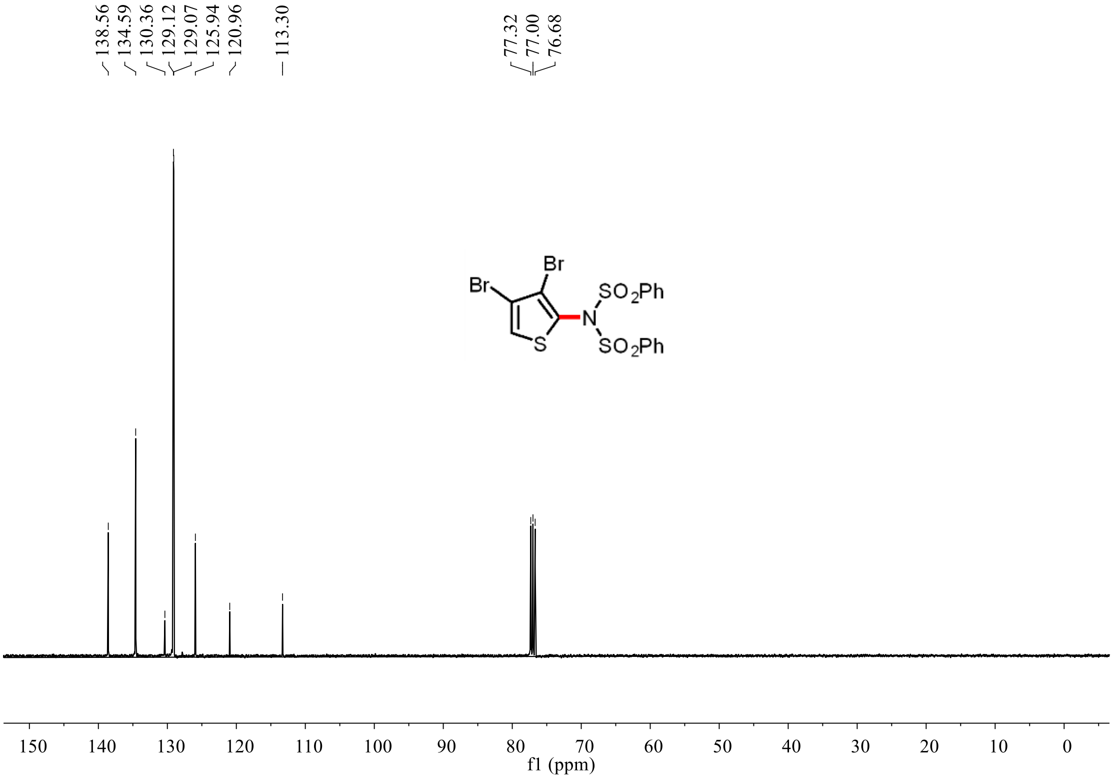

Supplementary Figure 38. ^{13}C NMR (101 MHz, $CDCl_3$) spectrum of **3as**

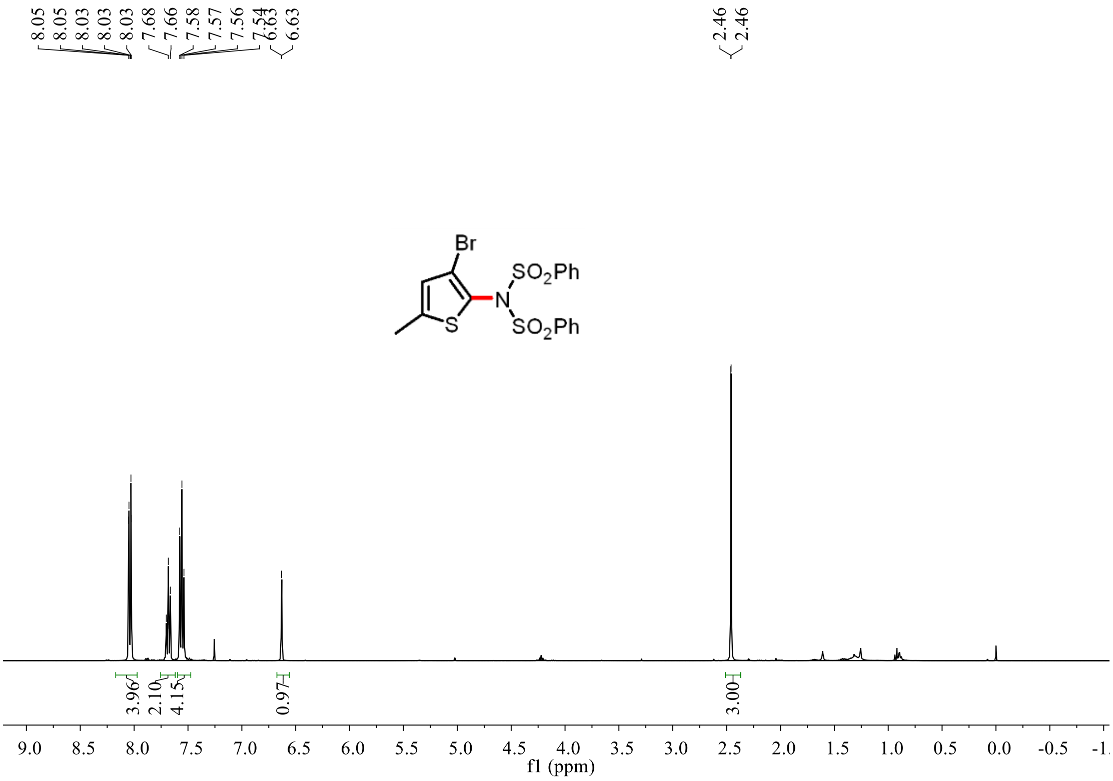

Supplementary Figure 39. ^{1}H NMR (400 MHz, $CDCl_3$) spectrum of **3at**

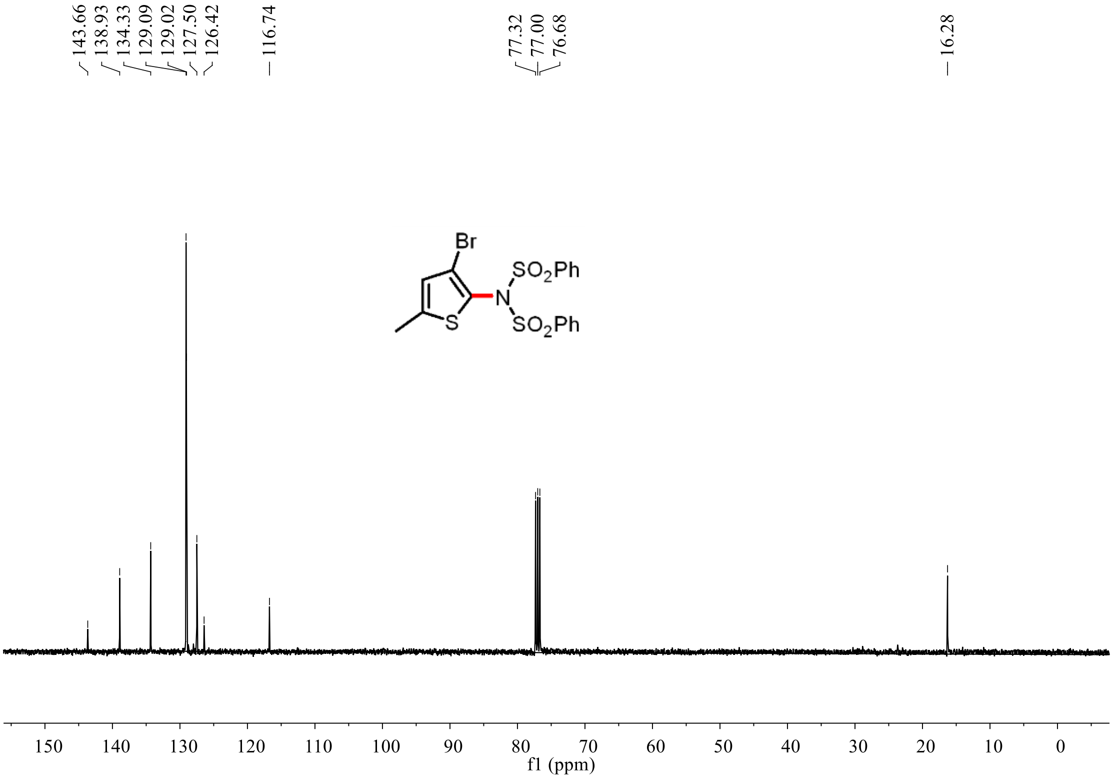

Supplementary Figure 40. ^{13}C NMR (101 MHz, $CDCl_3$) spectrum of **3at**

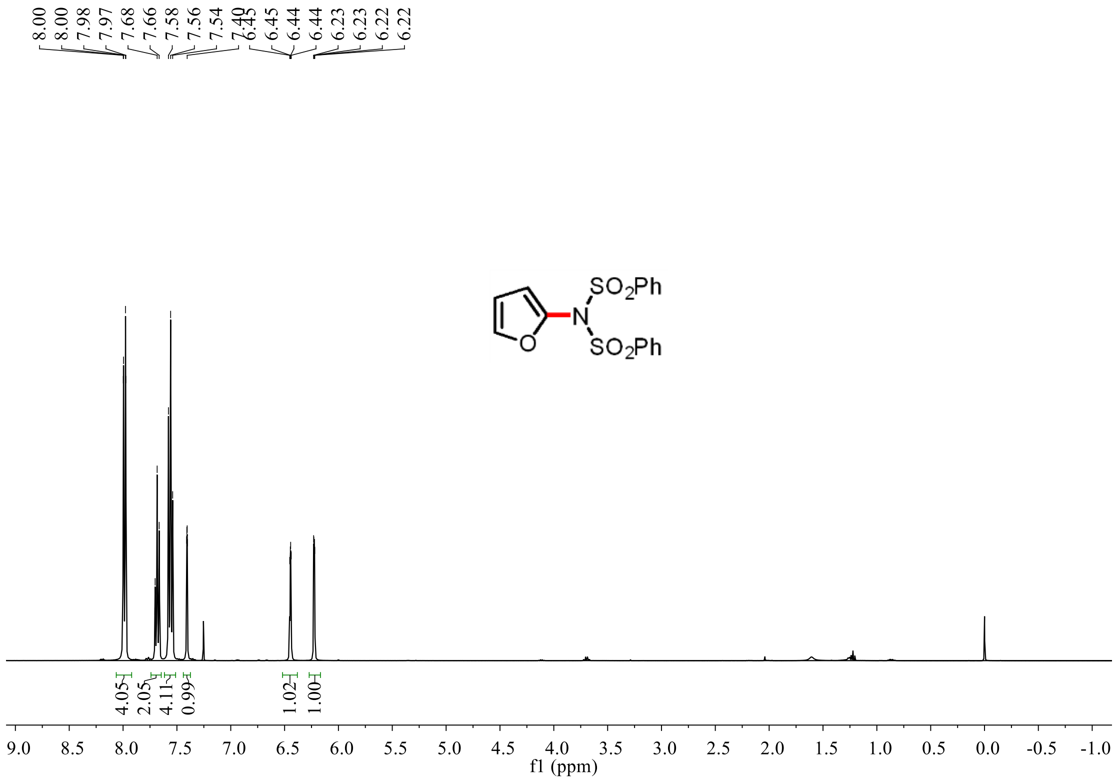

Supplementary Figure 41. ^{1}H NMR (400 MHz, $CDCl_3$) spectrum of **3au**

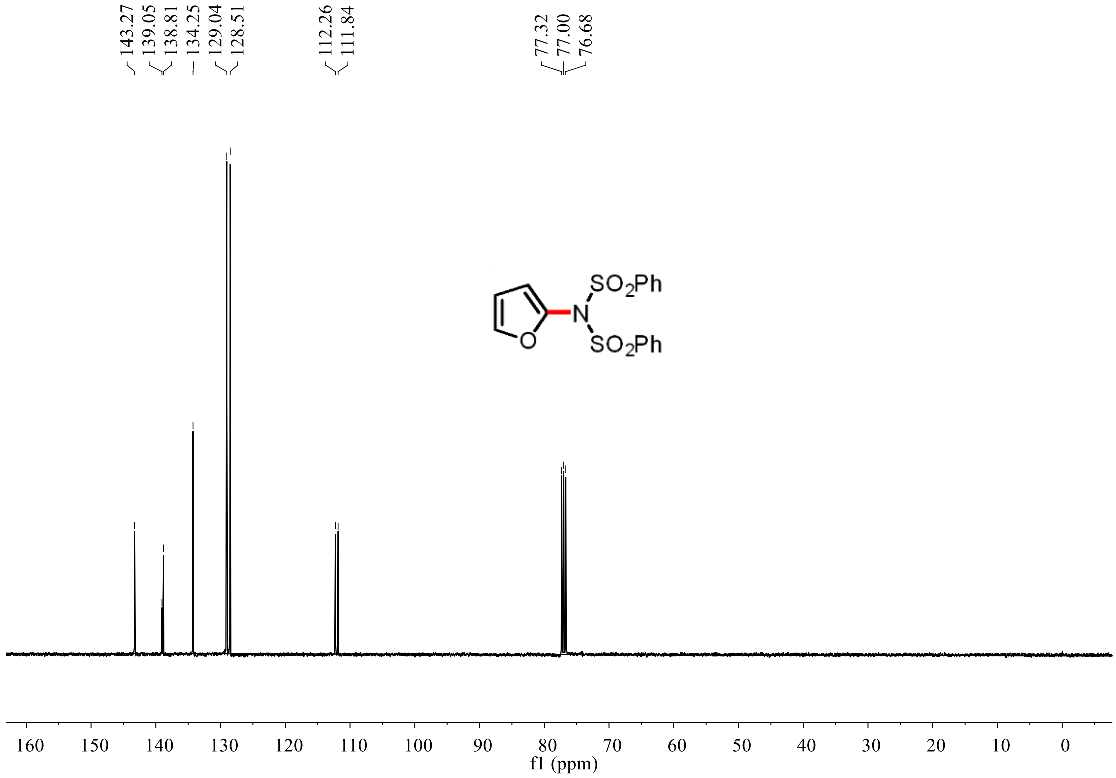

Supplementary Figure 42. ^{13}C NMR (101 MHz, $CDCl_3$) spectrum of **3au**

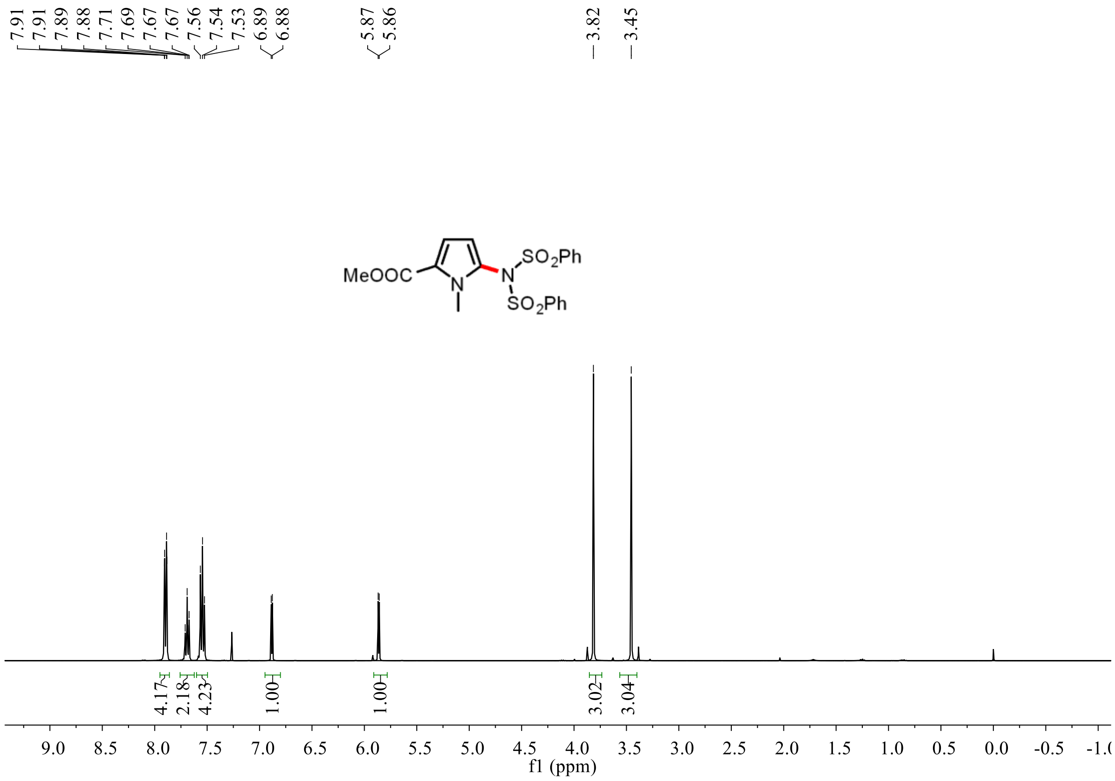

Supplementary Figure 43. ^{1}H NMR (400 MHz, $CDCl_3$) spectrum of **3av**

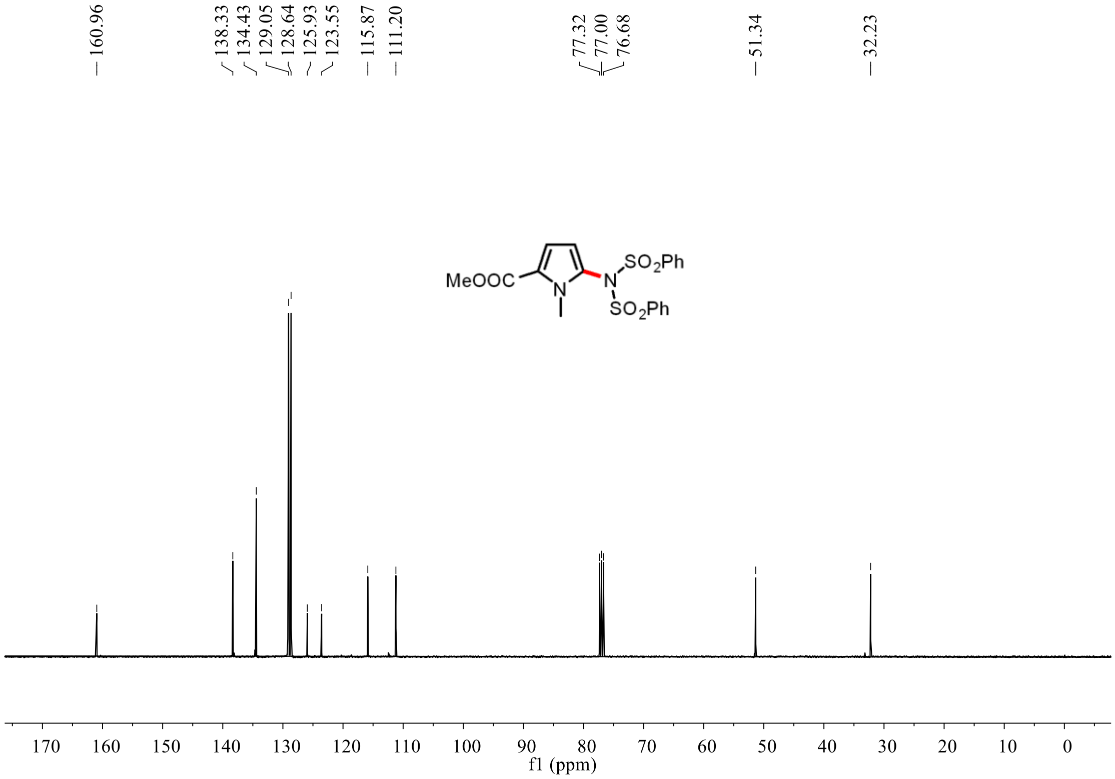

Supplementary Figure 44. ^{13}C NMR (101 MHz, $CDCl_3$) spectrum of **3av**

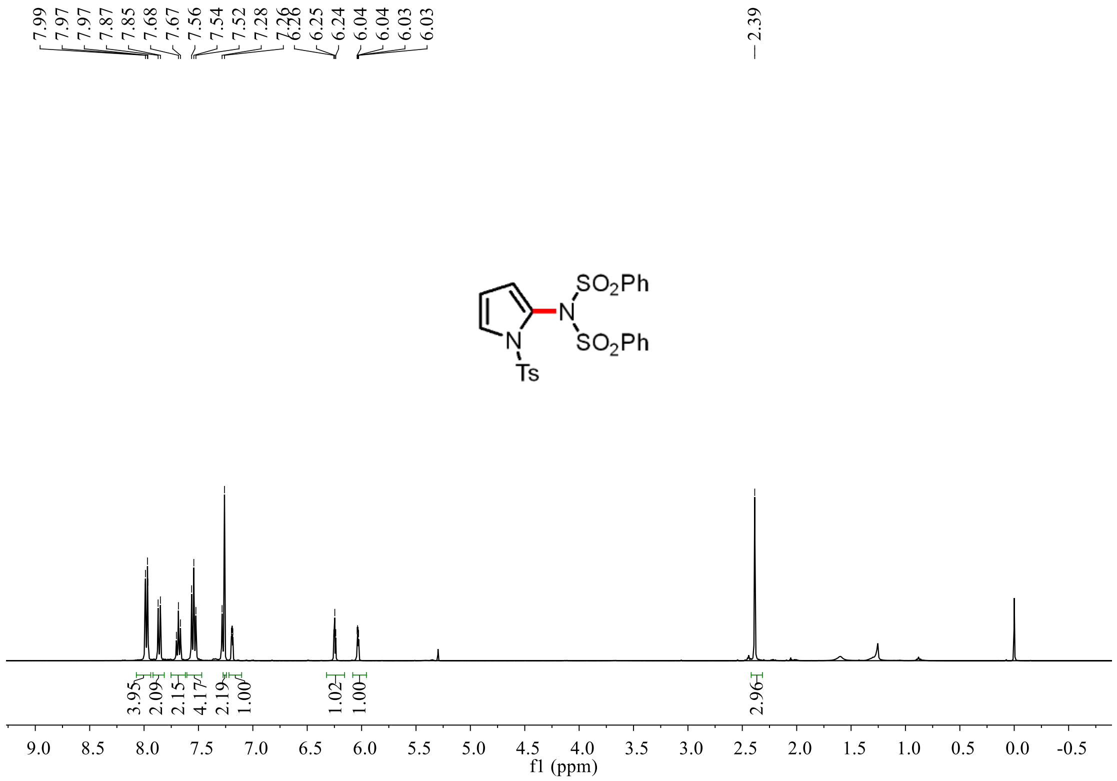

Supplementary Figure 45. ^{1}H NMR (400 MHz, $CDCl_3$) spectrum of **3aw**

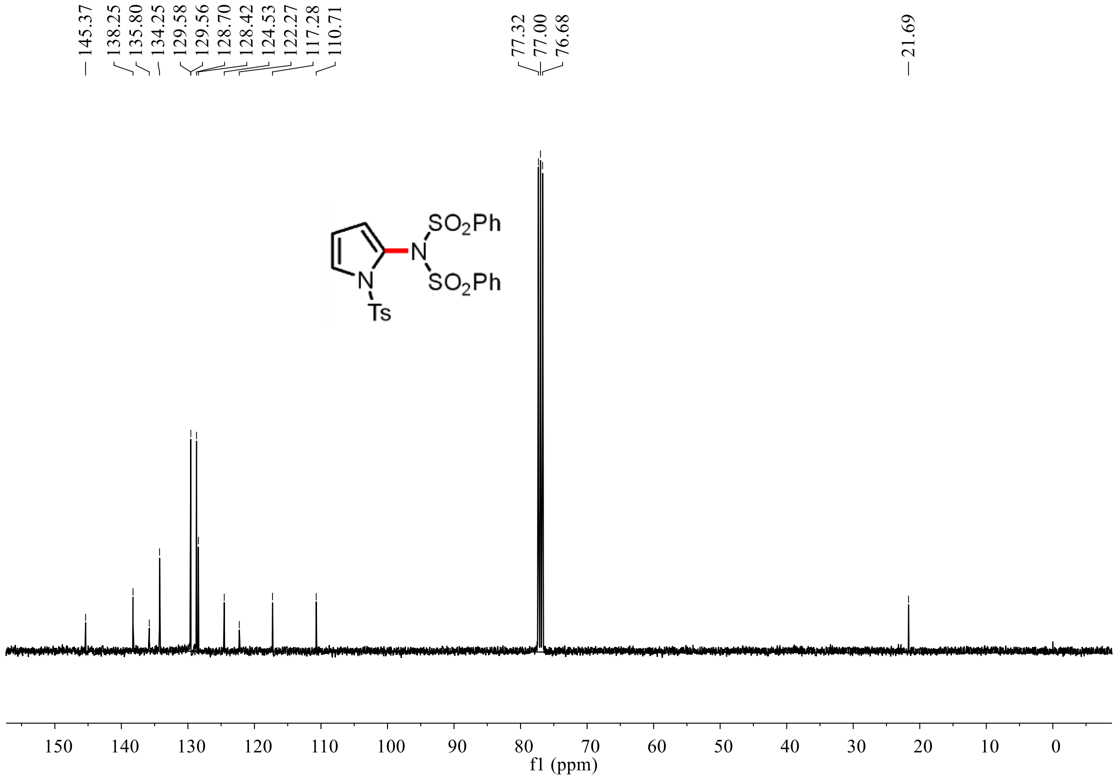

Supplementary Figure 46. ^{13}C NMR (101 MHz, $CDCl_3$) spectrum of **3aw**

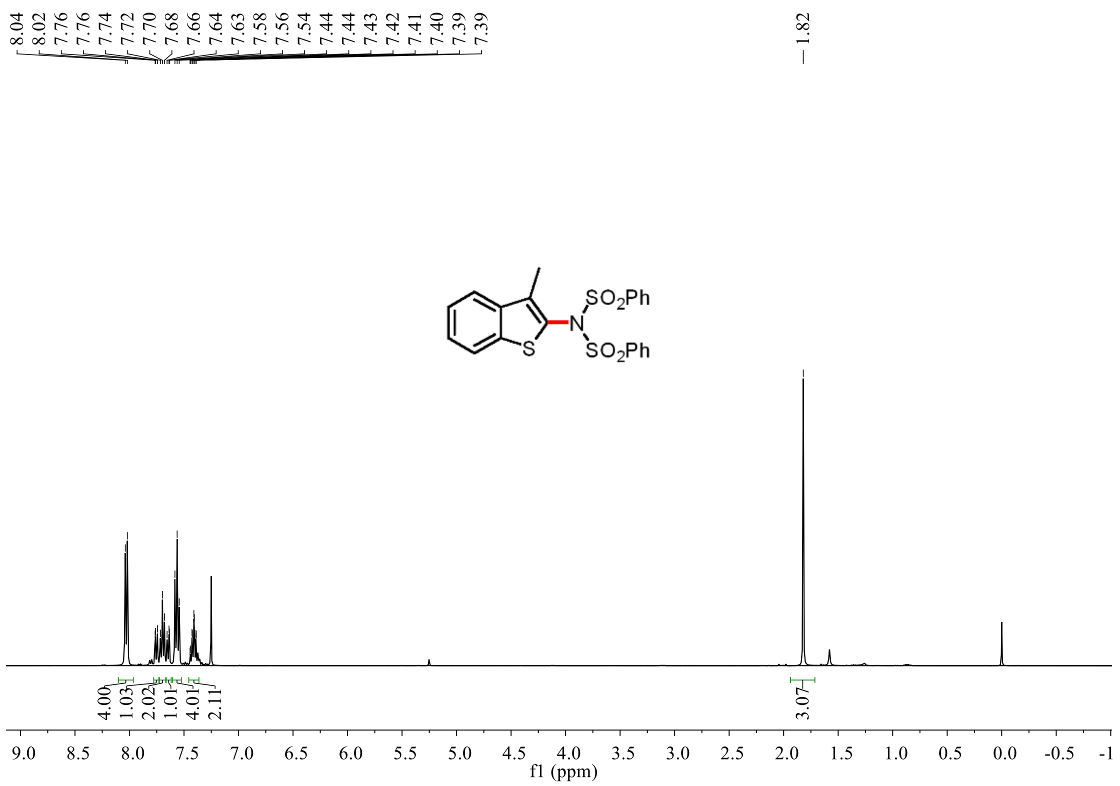

Supplementary Figure 47. ^{1}H NMR (400 MHz, $CDCl_3$) spectrum of **3ax**

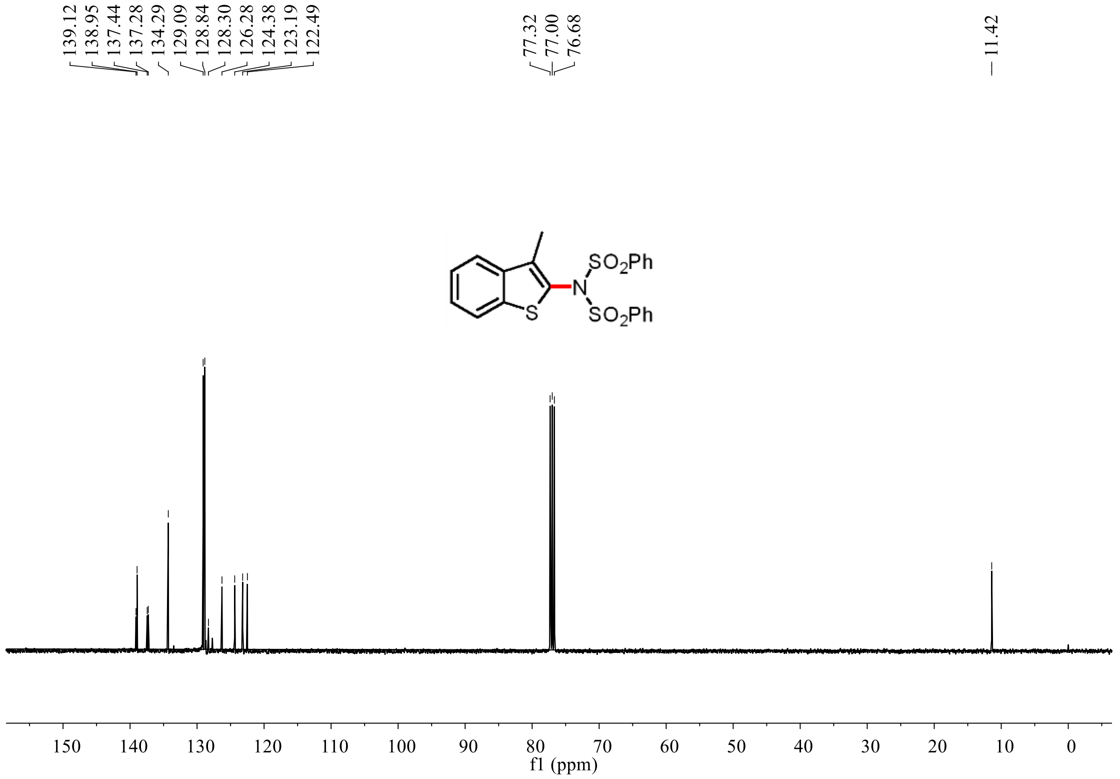

Supplementary Figure 48. ^{13}C NMR (101 MHz, $CDCl_3$) spectrum of **3ax**

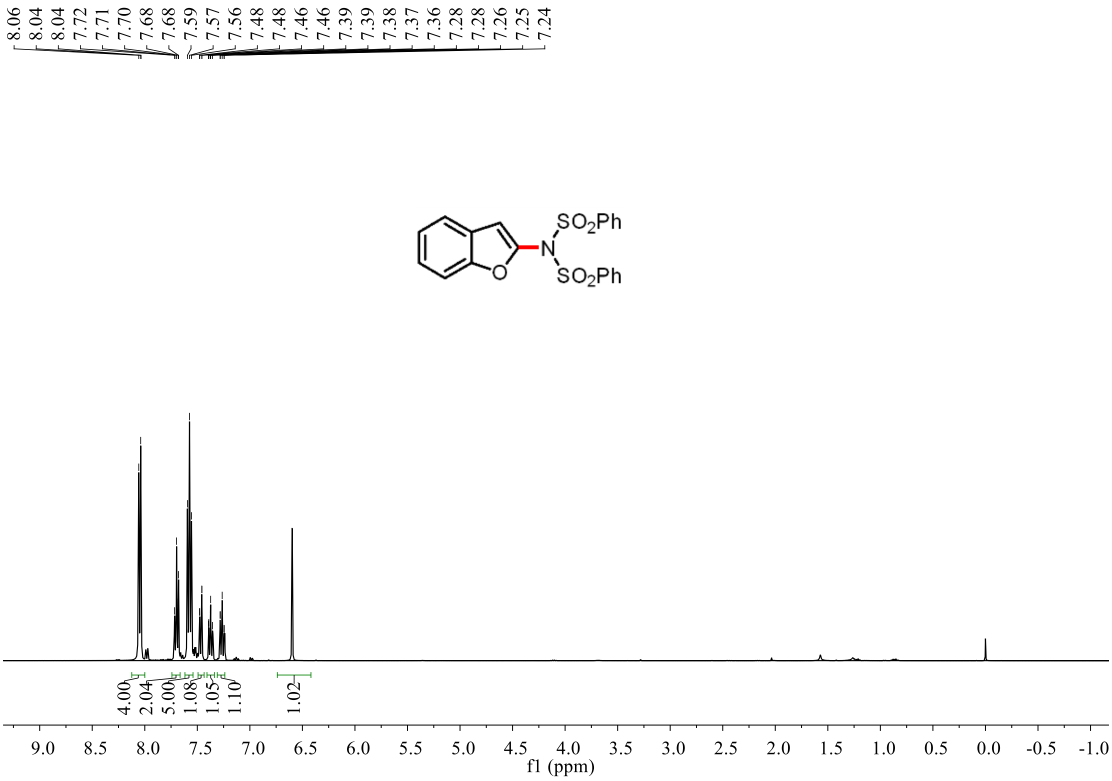

Supplementary Figure 49. ^{1}H NMR (400 MHz, $CDCl_3$) spectrum of **3ay**

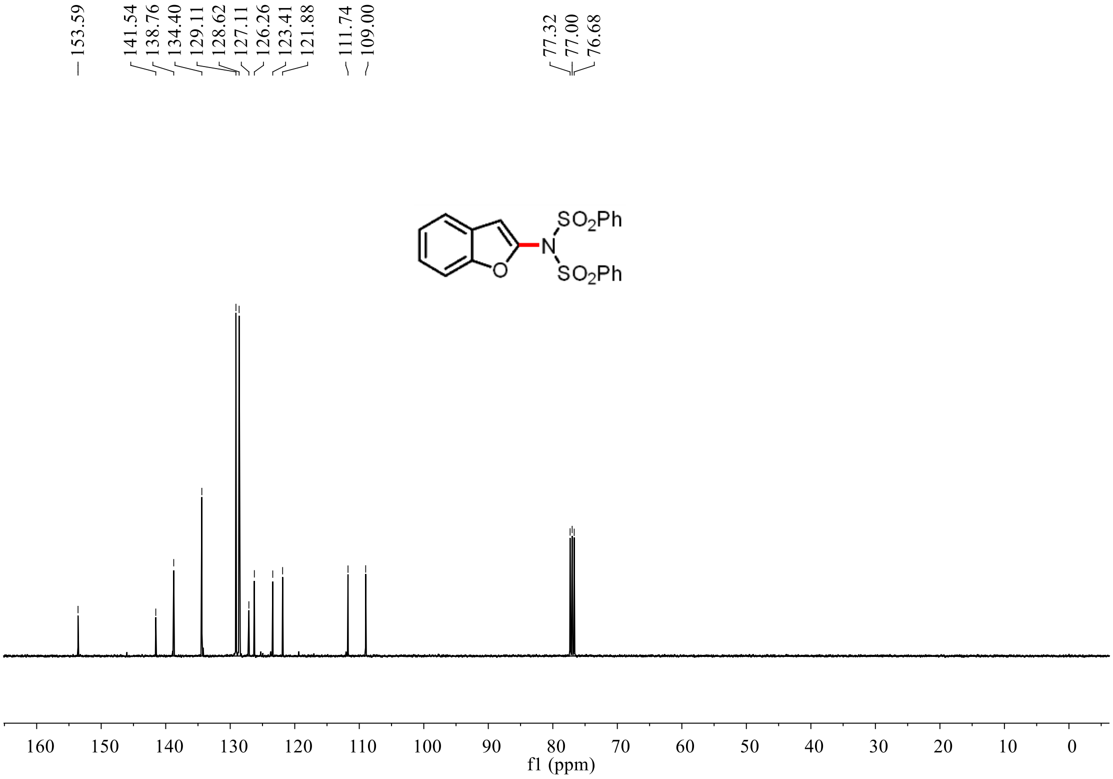

Supplementary Figure 50. ^{13}C NMR (101 MHz, $CDCl_3$) spectrum of **3ay**

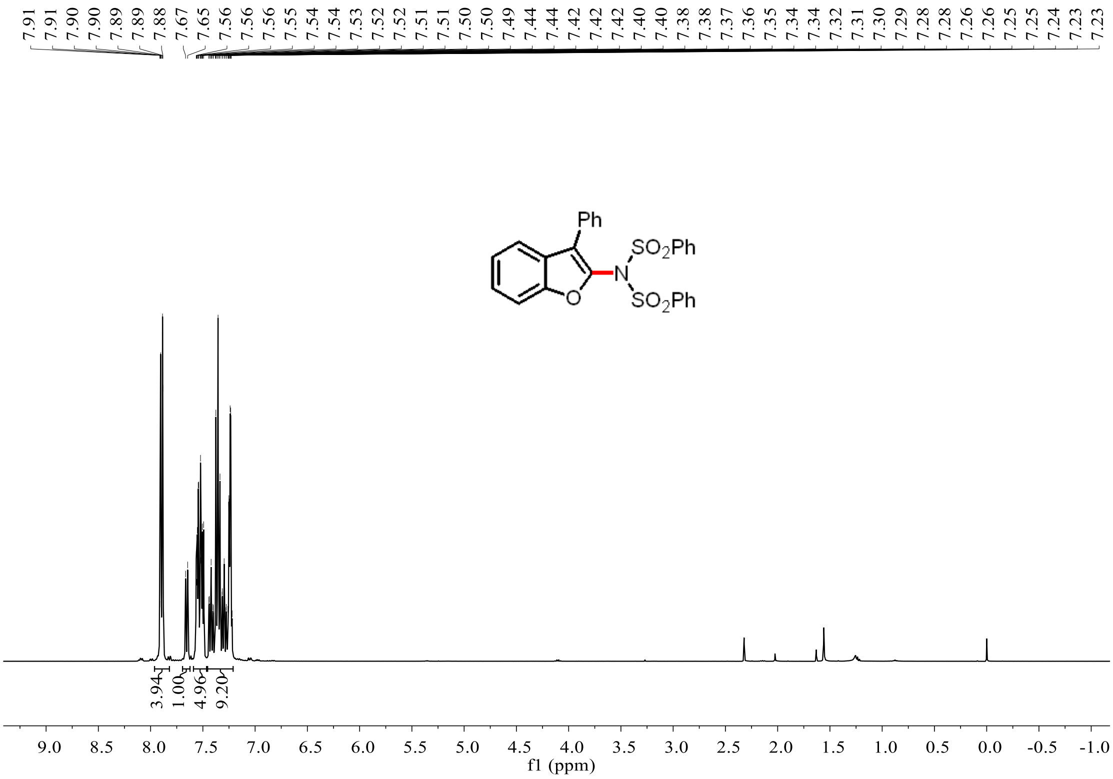

Supplementary Figure 51. ^{1}H NMR (400 MHz, $CDCl_3$) spectrum of **3az**

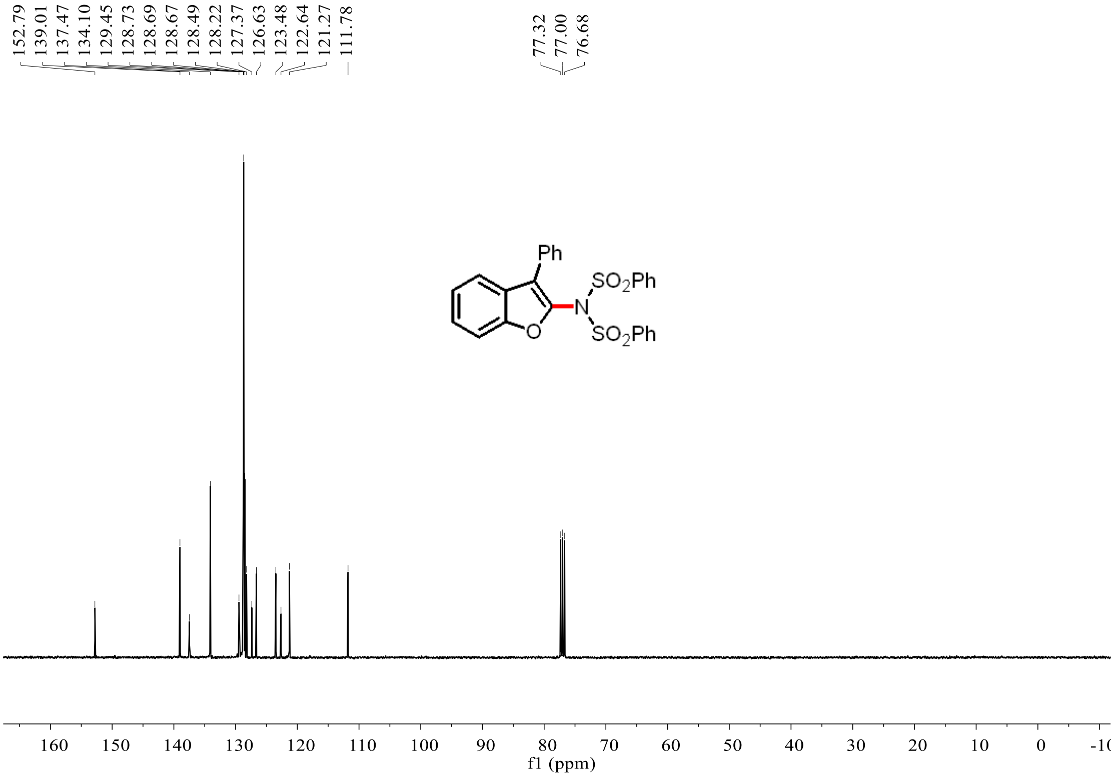

Supplementary Figure 52. ^{13}C NMR (101 MHz, $CDCl_3$) spectrum of **3az**

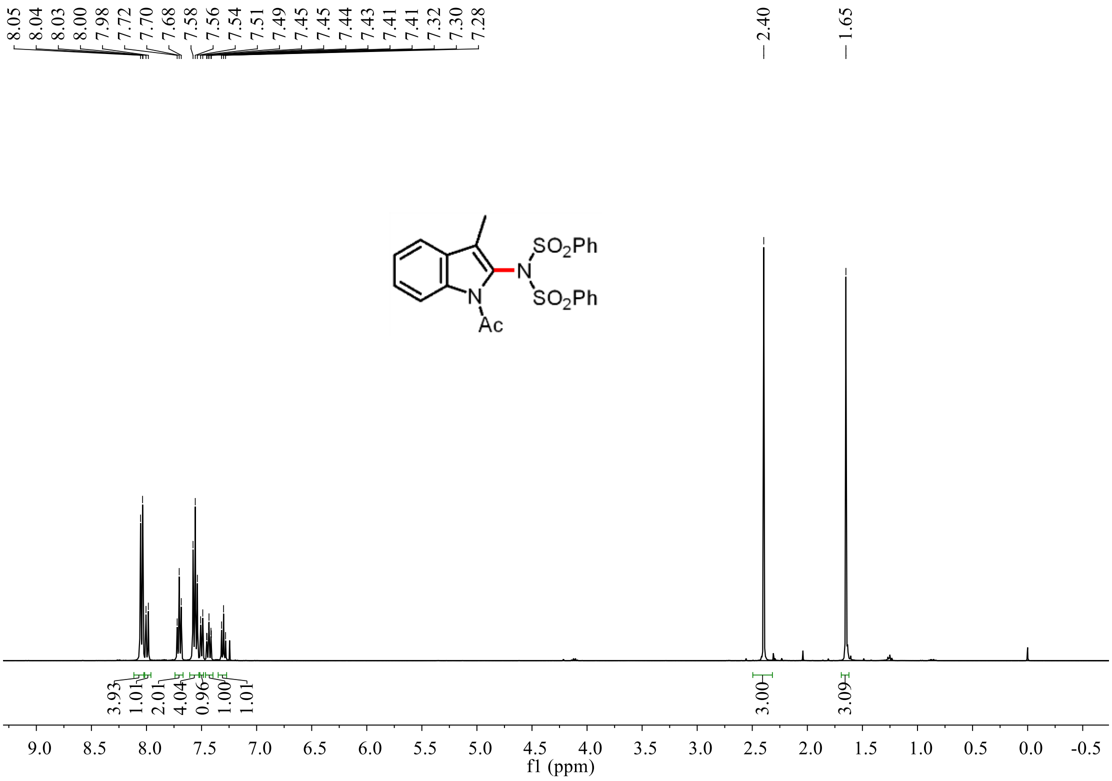

Supplementary Figure 53. ^{1}H NMR (400 MHz, $CDCl_3$) spectrum of **3ba**

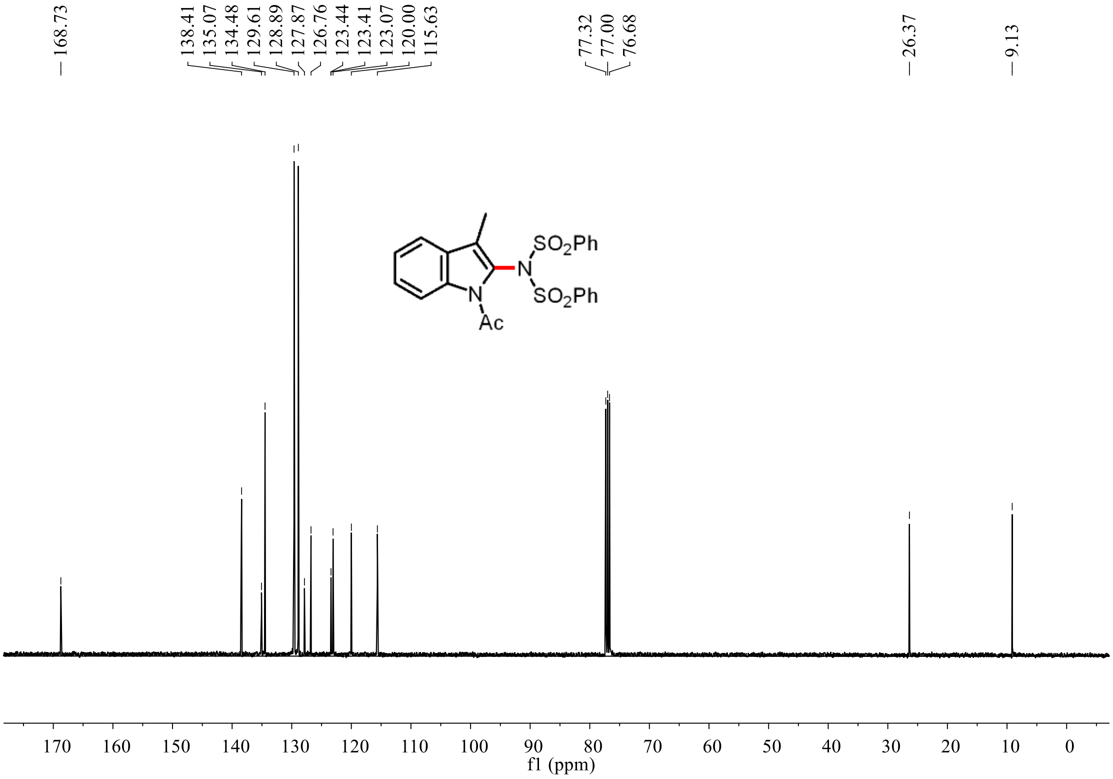

Supplementary Figure 54. ^{13}C NMR (101 MHz, $CDCl_3$) spectrum of **3ba**

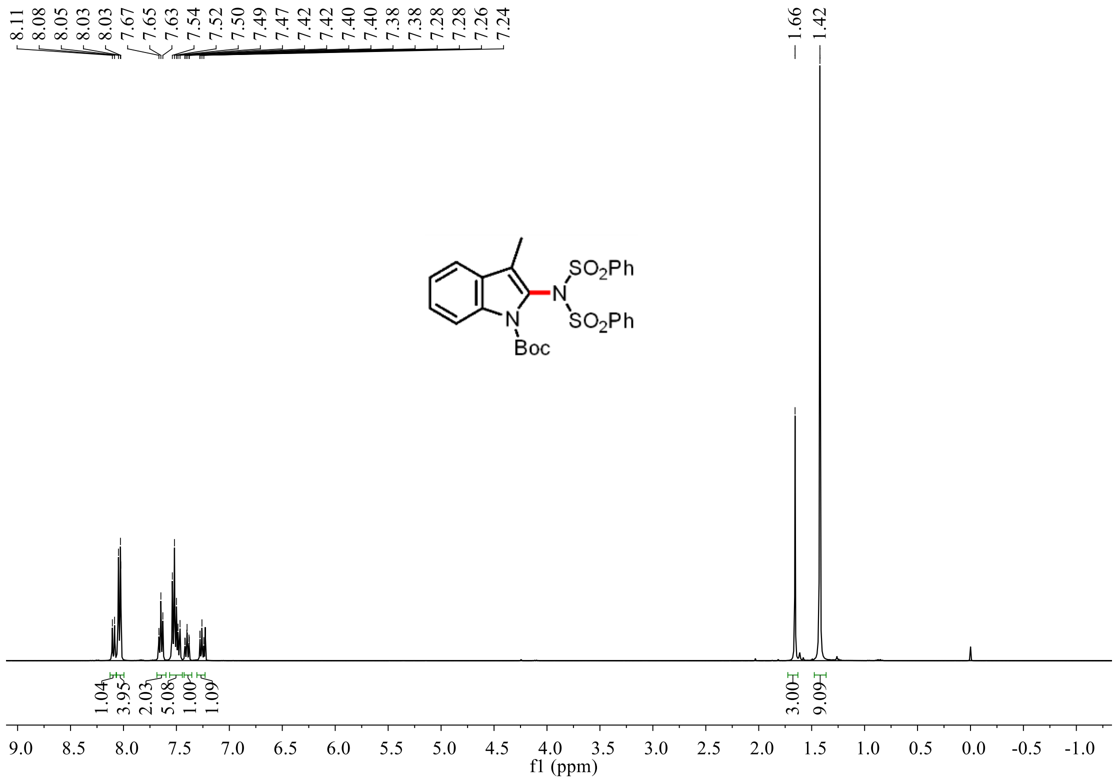

Supplementary Figure 55. ^{1}H NMR (400 MHz, $CDCl_3$) spectrum of **3bb**

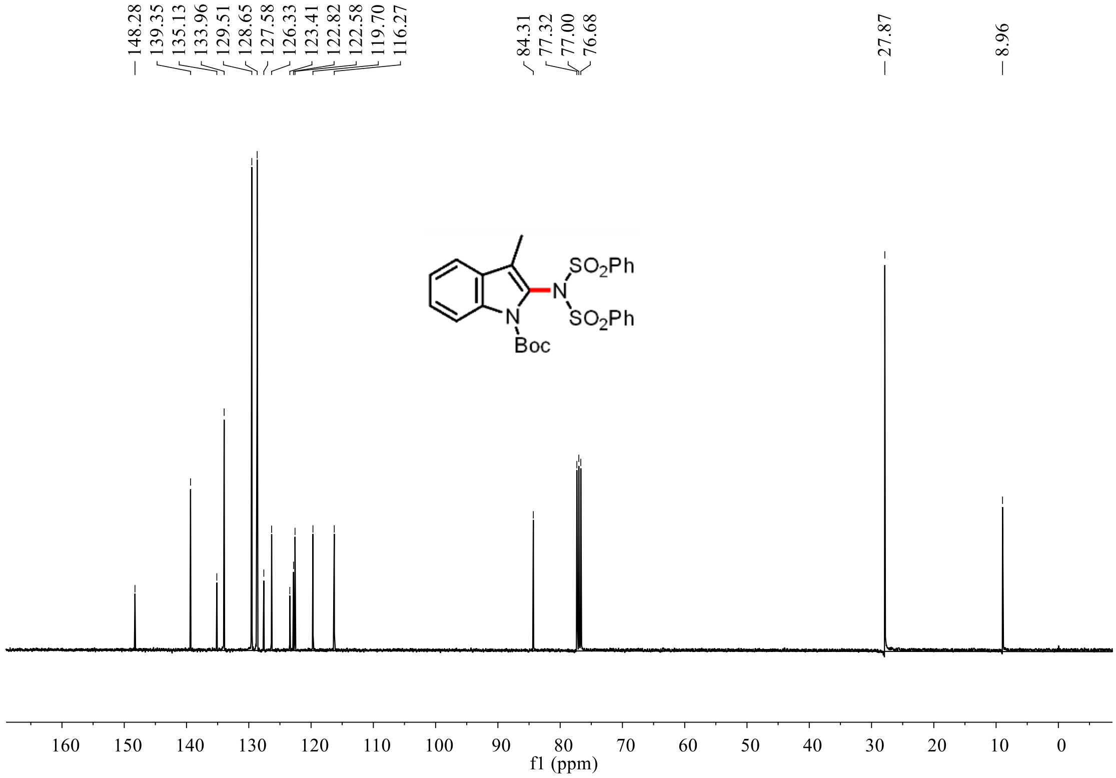

Supplementary Figure 56. ^{13}C NMR (101 MHz, $CDCl_3$) spectrum of **3bb**

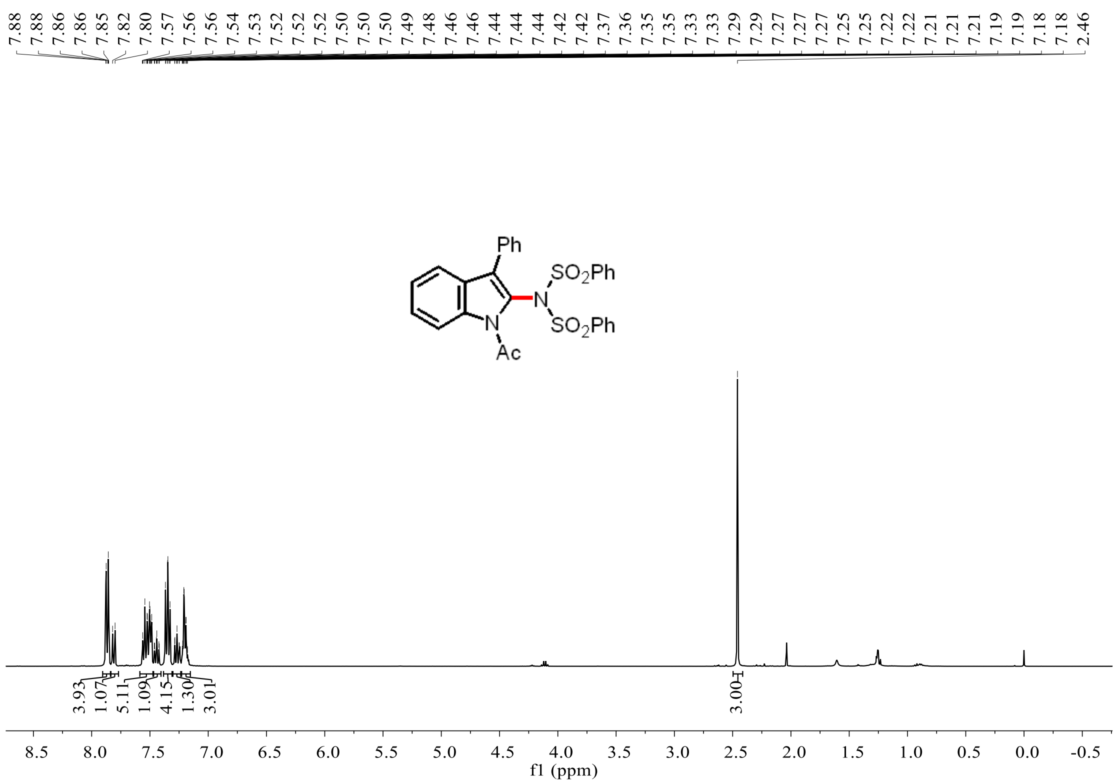

Supplementary Figure 57. ^{1}H NMR (400 MHz, $CDCl_3$) spectrum of **3bc**

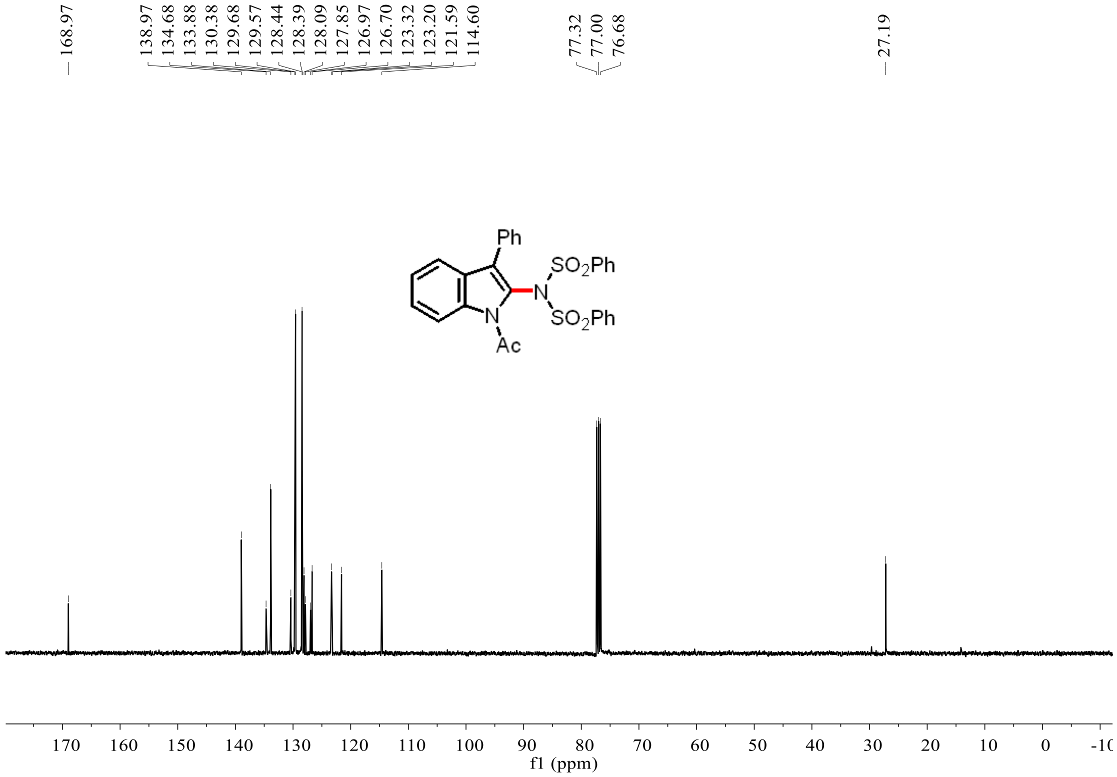

Supplementary Figure 58. ^{13}C NMR (101 MHz, $CDCl_3$) spectrum of **3bc**

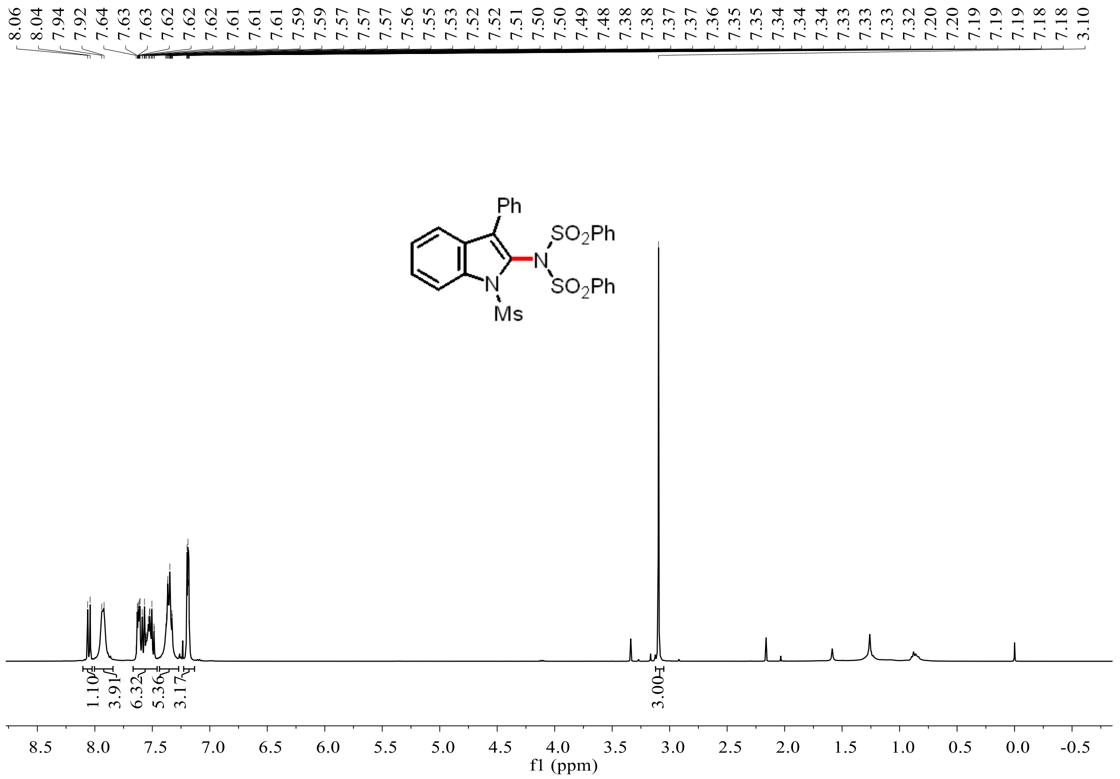

Supplementary Figure 59. ^{1}H NMR (400 MHz, $CDCl_3$) spectrum of **3bd**

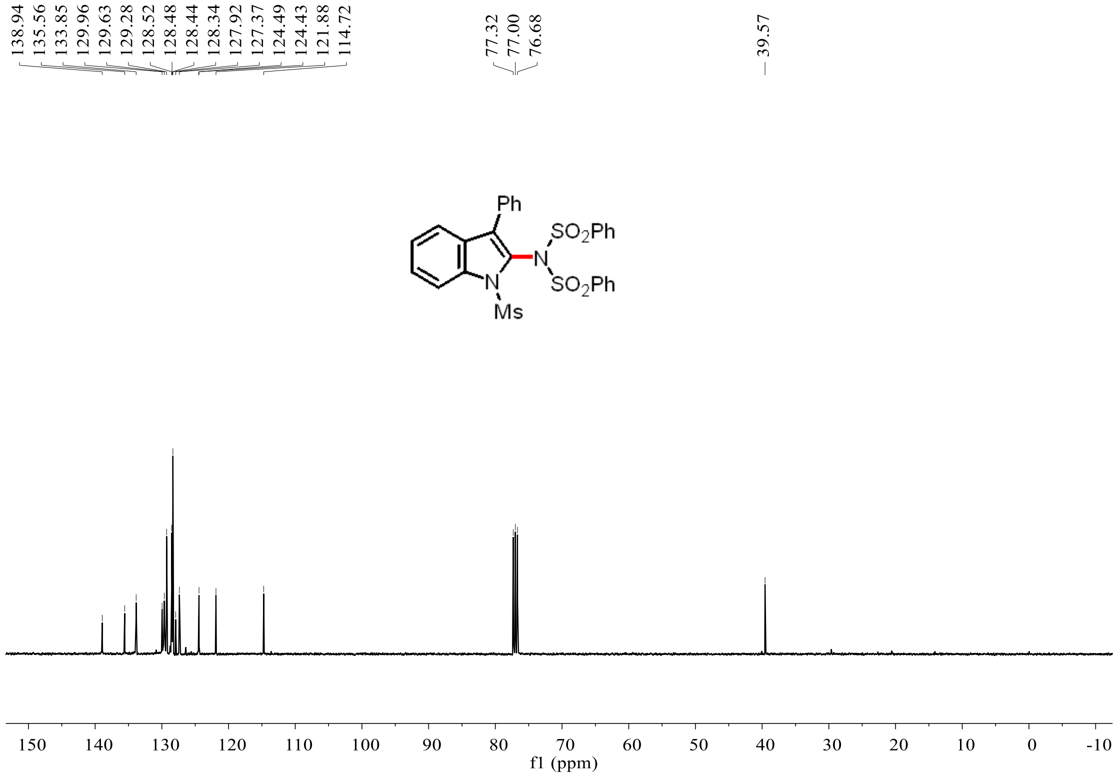

Supplementary Figure 60. ^{13}C NMR (101 MHz, $CDCl_3$) spectrum of **3bd**

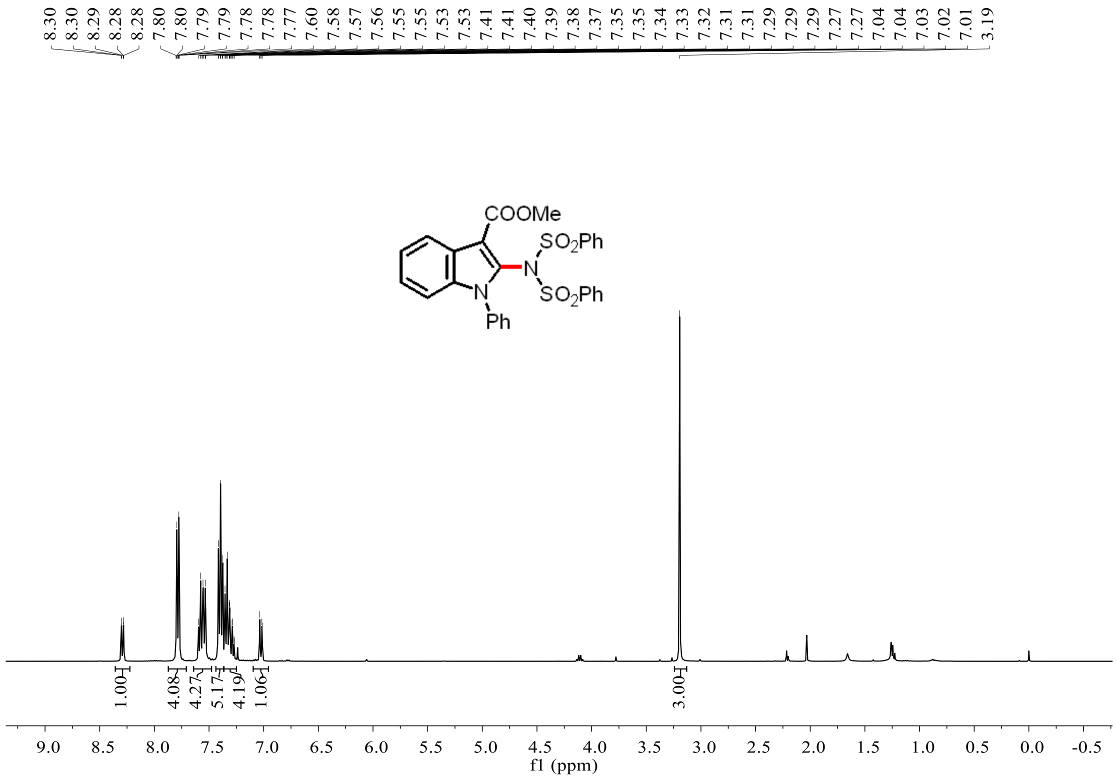

Supplementary Figure 61. ^{1}H NMR (400 MHz, $CDCl_3$) spectrum of **3be**

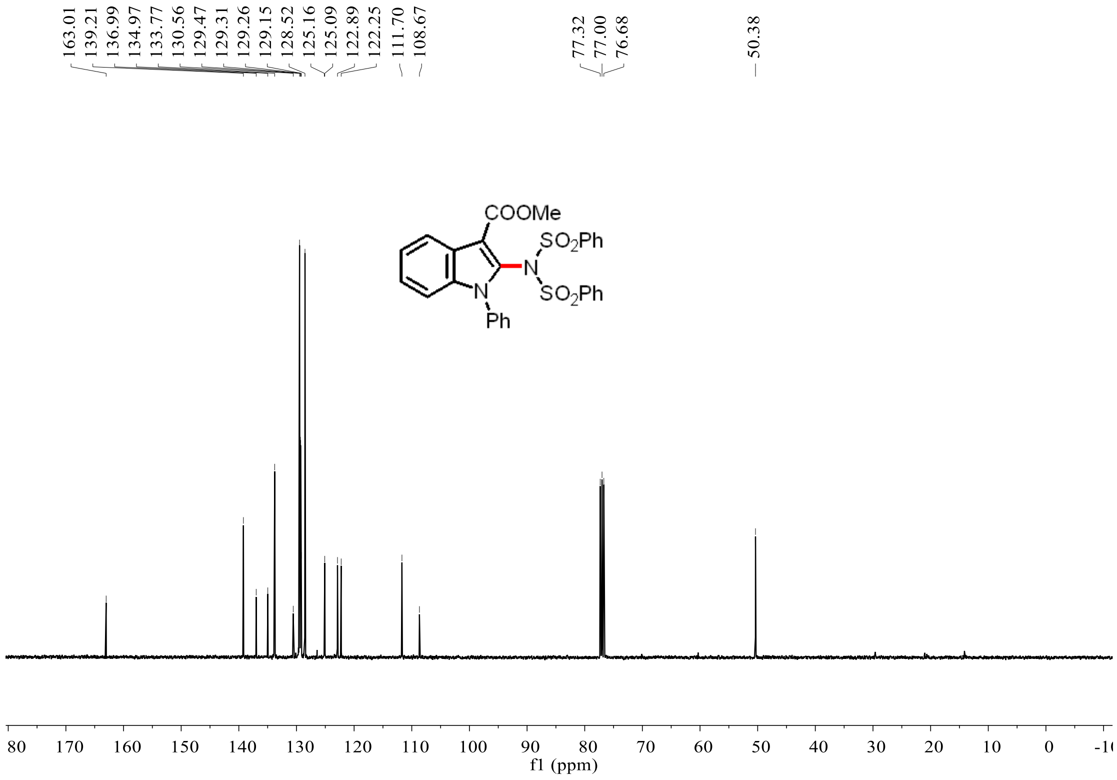

Supplementary Figure 62. ^{13}C NMR (101 MHz, $CDCl_3$) spectrum of **3be**

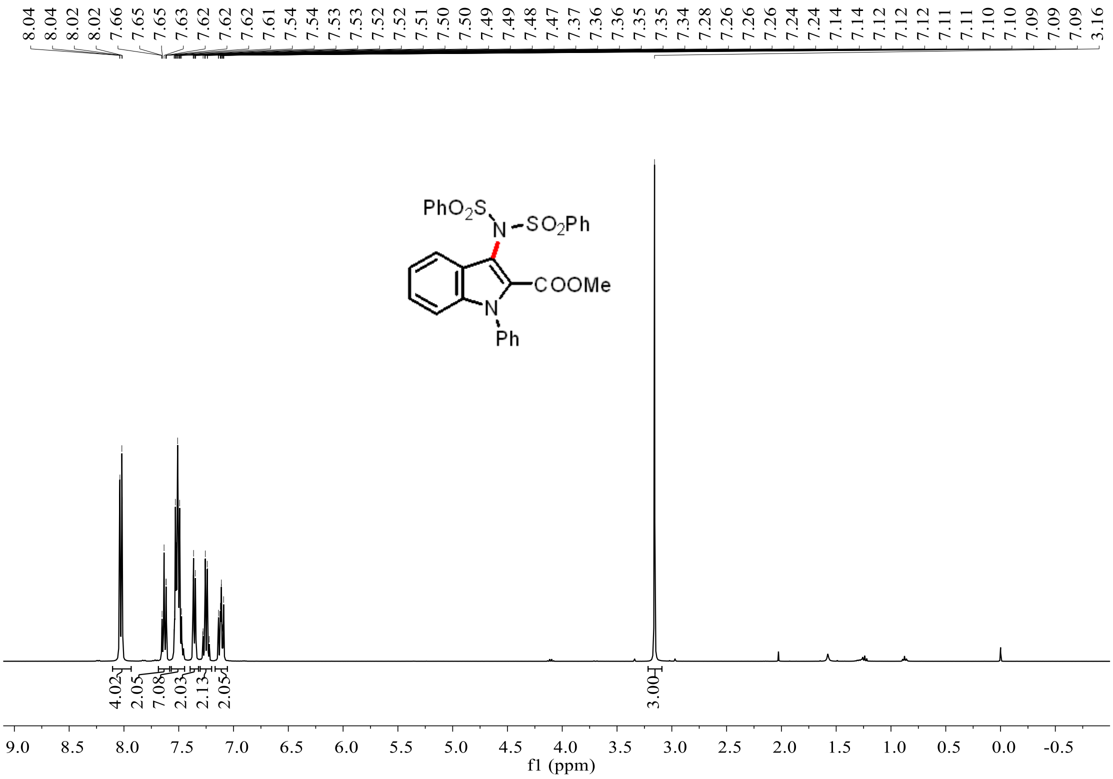

Supplementary Figure 63. ^{1}H NMR (400 MHz, $CDCl_3$) spectrum of **3bf**

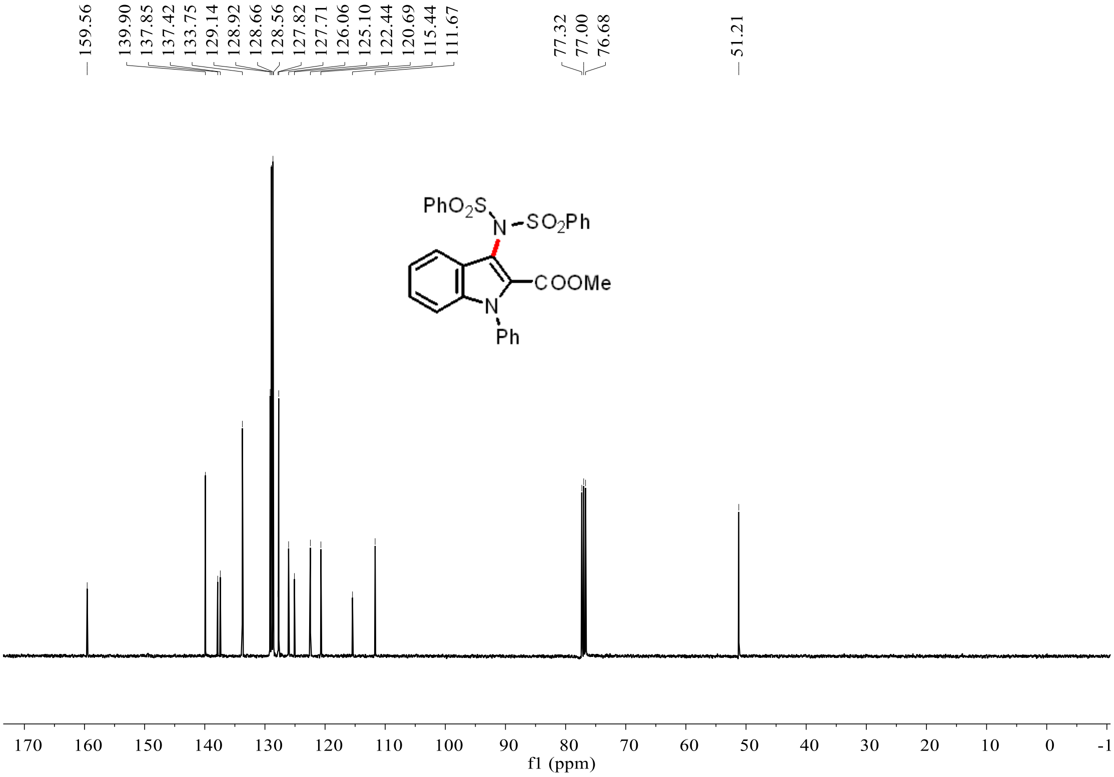

Supplementary Figure 64. ^{13}C NMR (101 MHz, $CDCl_3$) spectrum of **3bf**

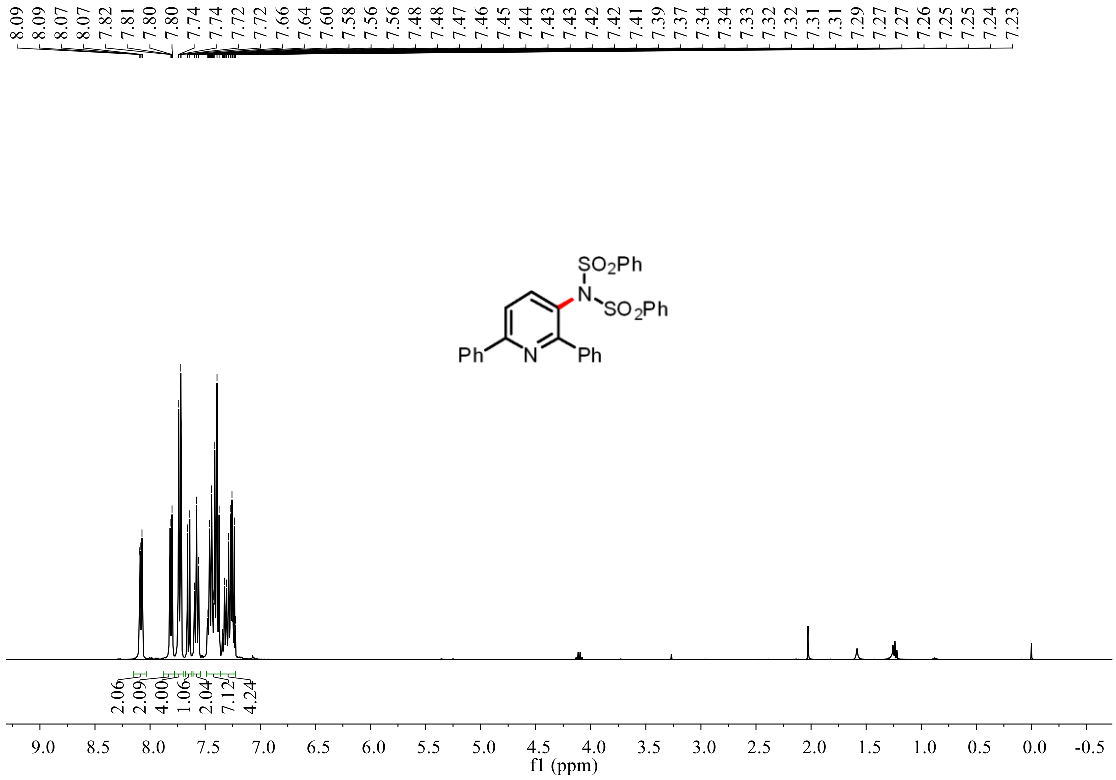

Supplementary Figure 65. ^{1}H NMR (400 MHz, $CDCl_3$) spectrum of **3bg**

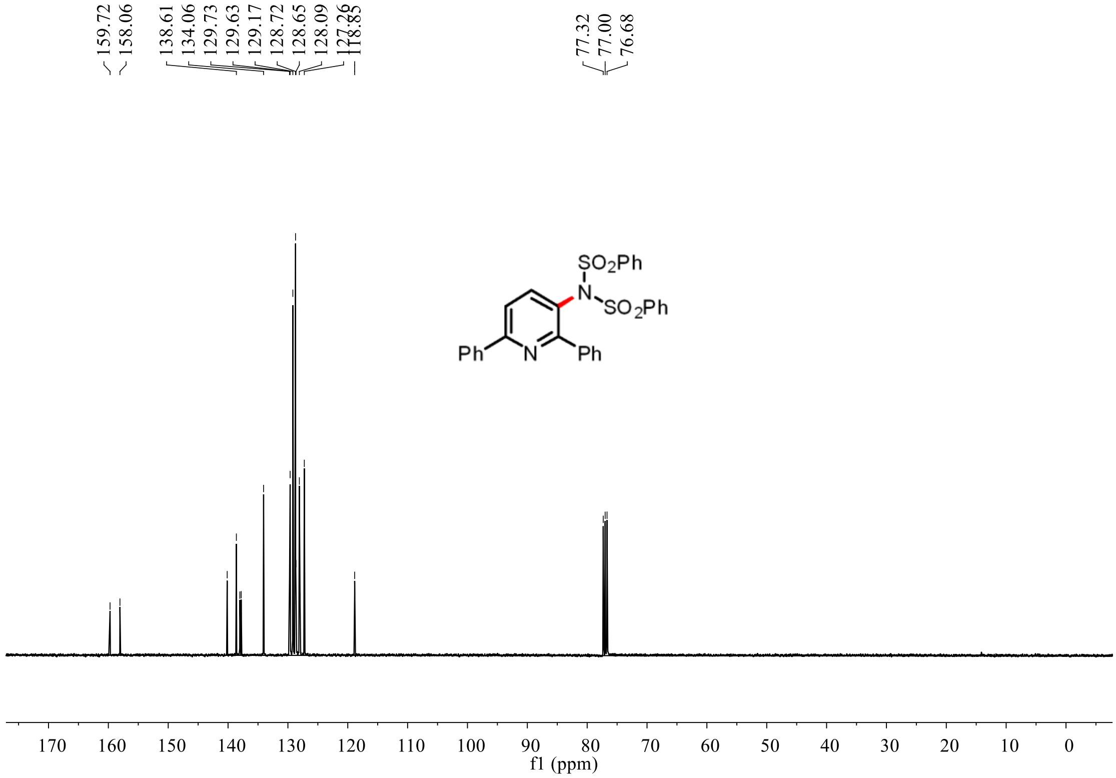

Supplementary Figure 66. ^{13}C NMR (101 MHz, $CDCl_3$) spectrum of **3bg**

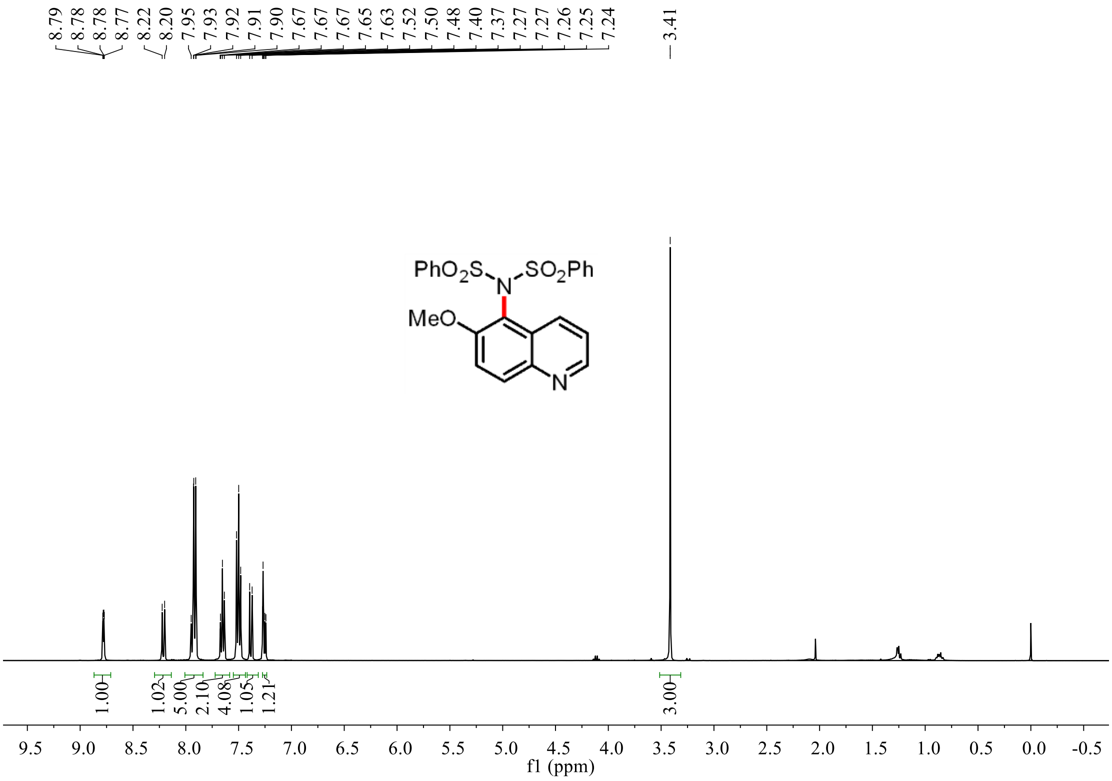

Supplementary Figure 67. ^{1}H NMR (400 MHz, $CDCl_3$) spectrum of **3bh**

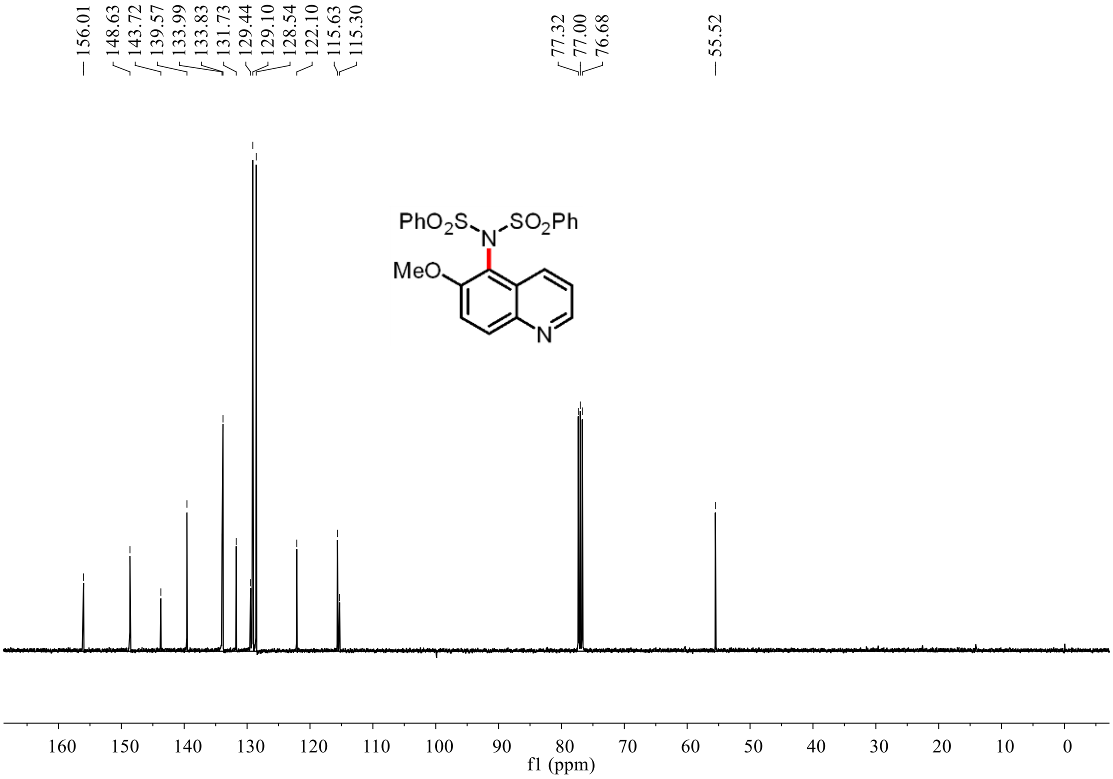

Supplementary Figure 68. ^{13}C NMR (101 MHz, $CDCl_3$) spectrum of **3bh**

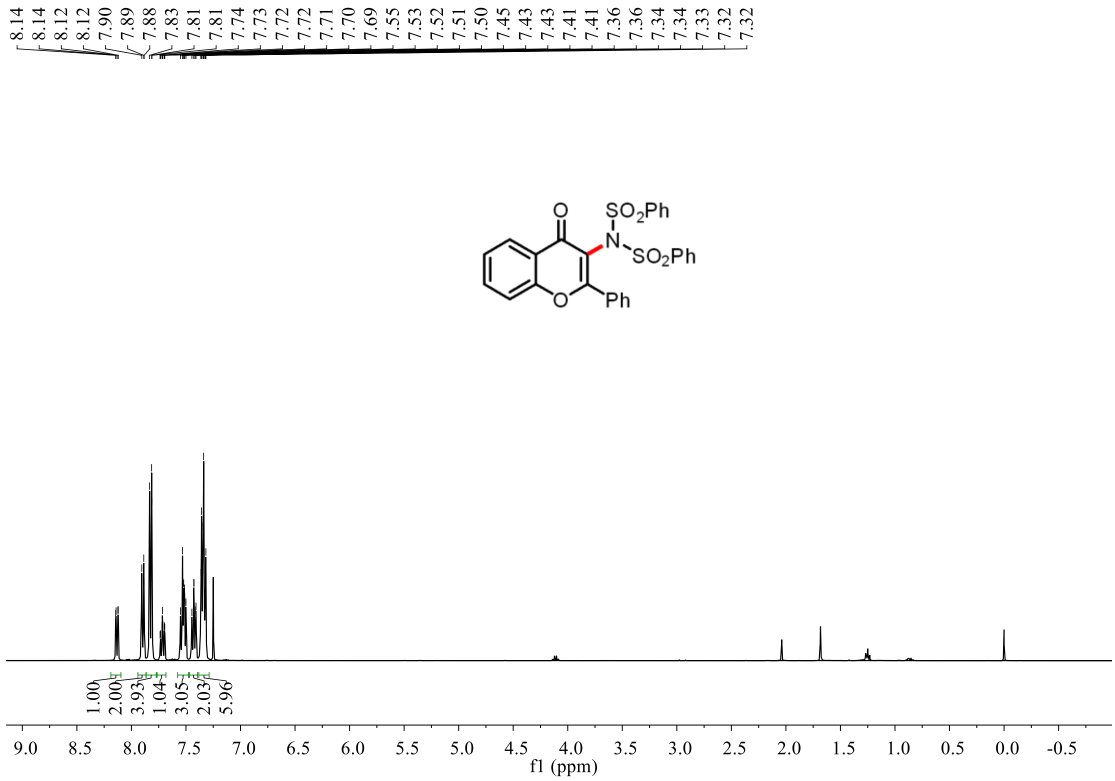

Supplementary Figure 69. ^{1}H NMR (400 MHz, $CDCl_3$) spectrum of **3bi**

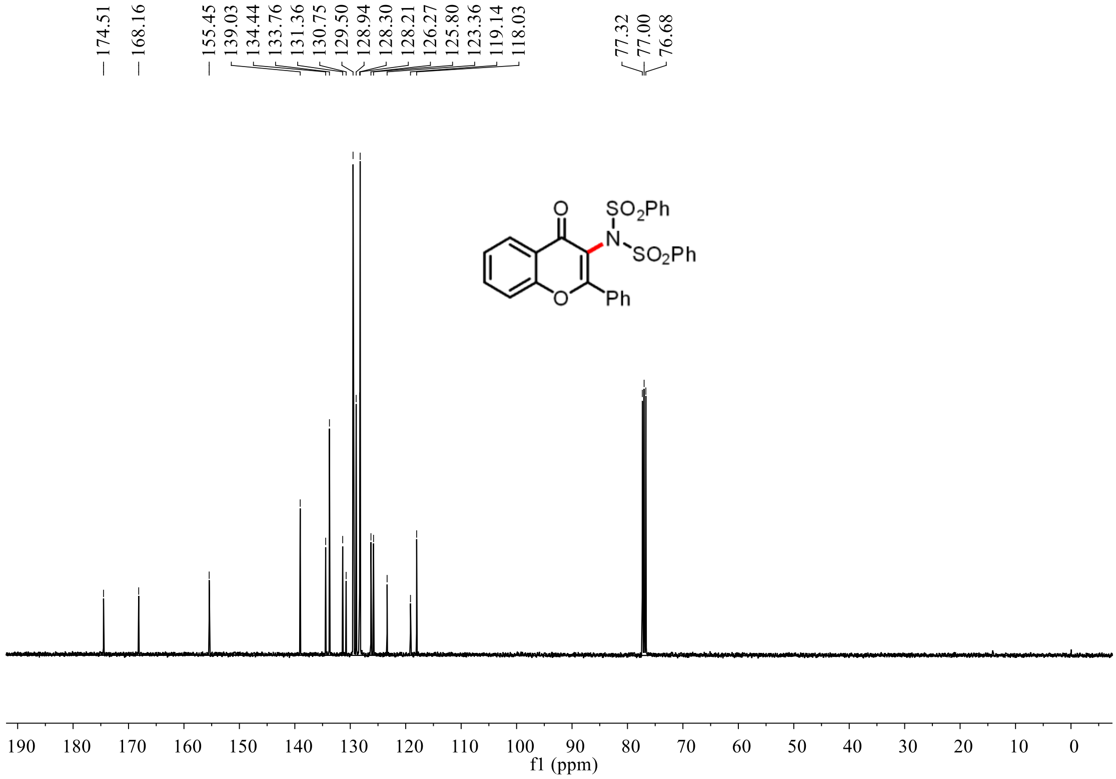

Supplementary Figure 70. ^{13}C NMR (101 MHz, $CDCl_3$) spectrum of **3bi**

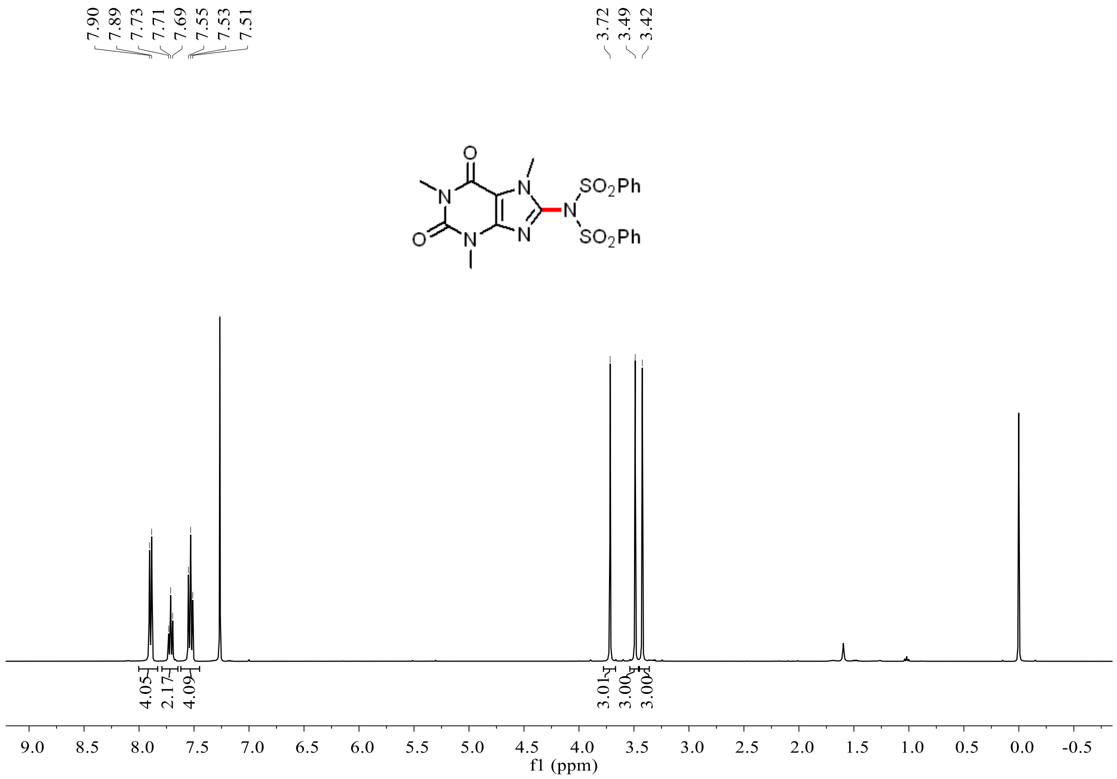

Supplementary Figure 71. ^{1}H NMR (400 MHz, $CDCl_3$) spectrum of **3bj**

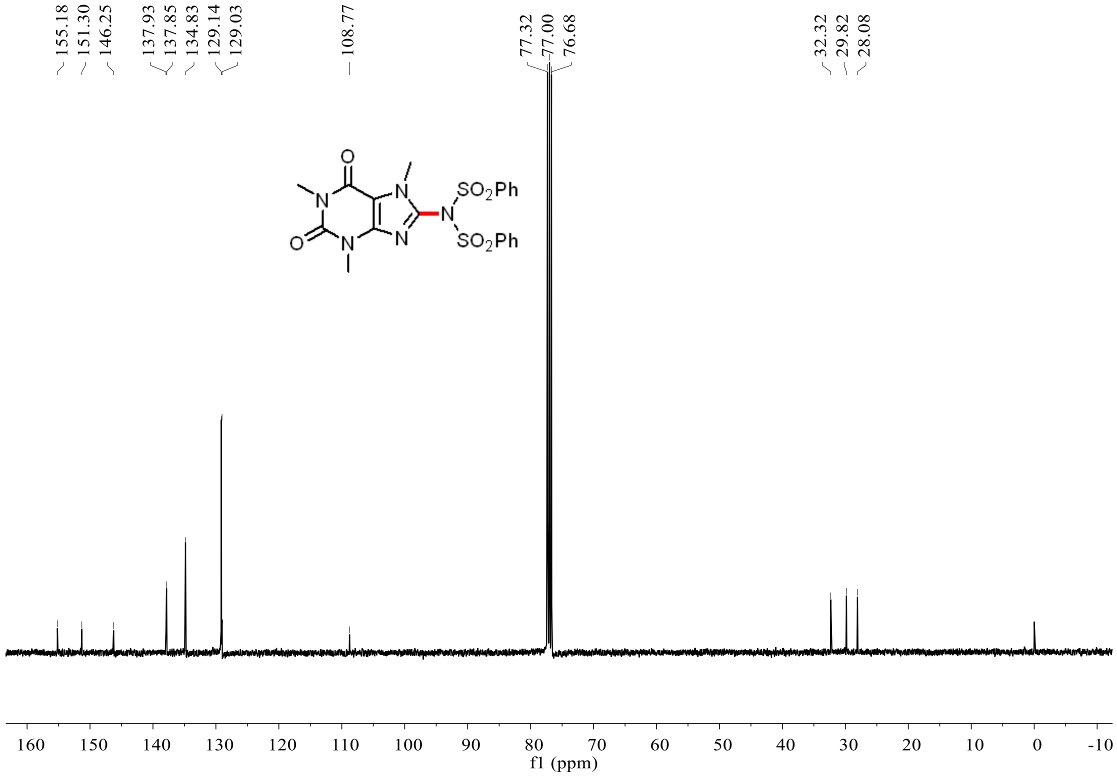

Supplementary Figure 72. ^{13}C NMR (101 MHz, $CDCl_3$) spectrum of **3bj**

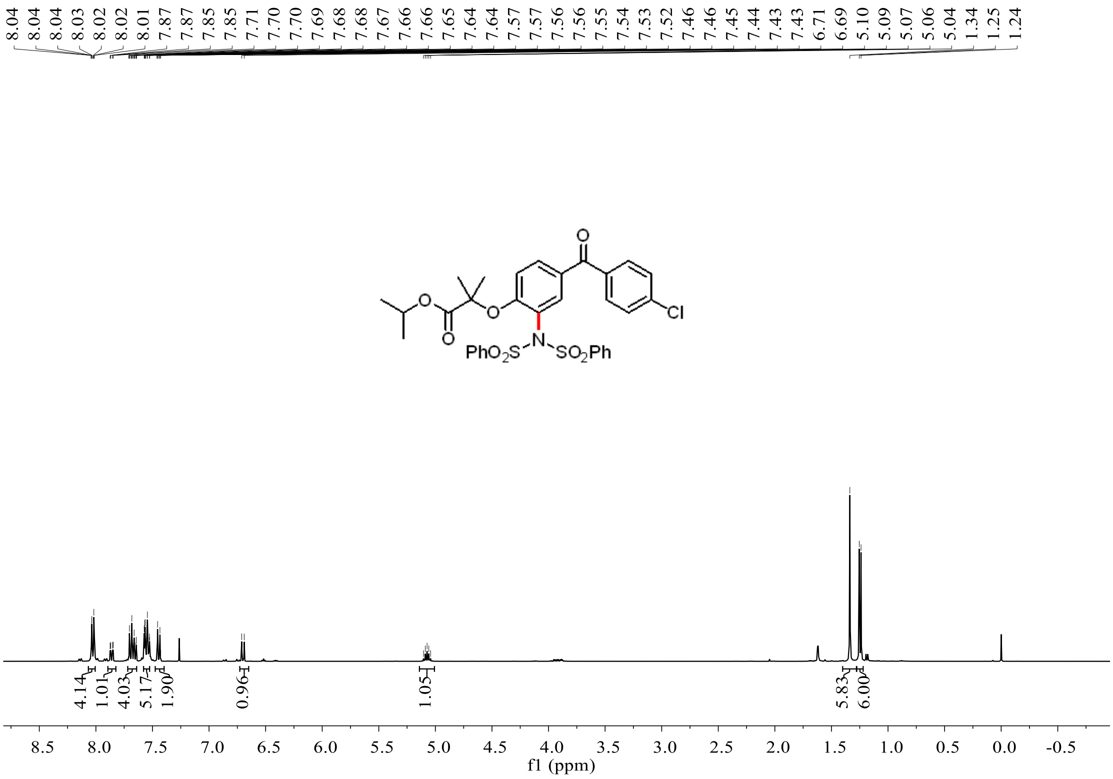

Supplementary Figure 73. ^{1}H NMR (400 MHz, $CDCl_3$) spectrum of **3bk**

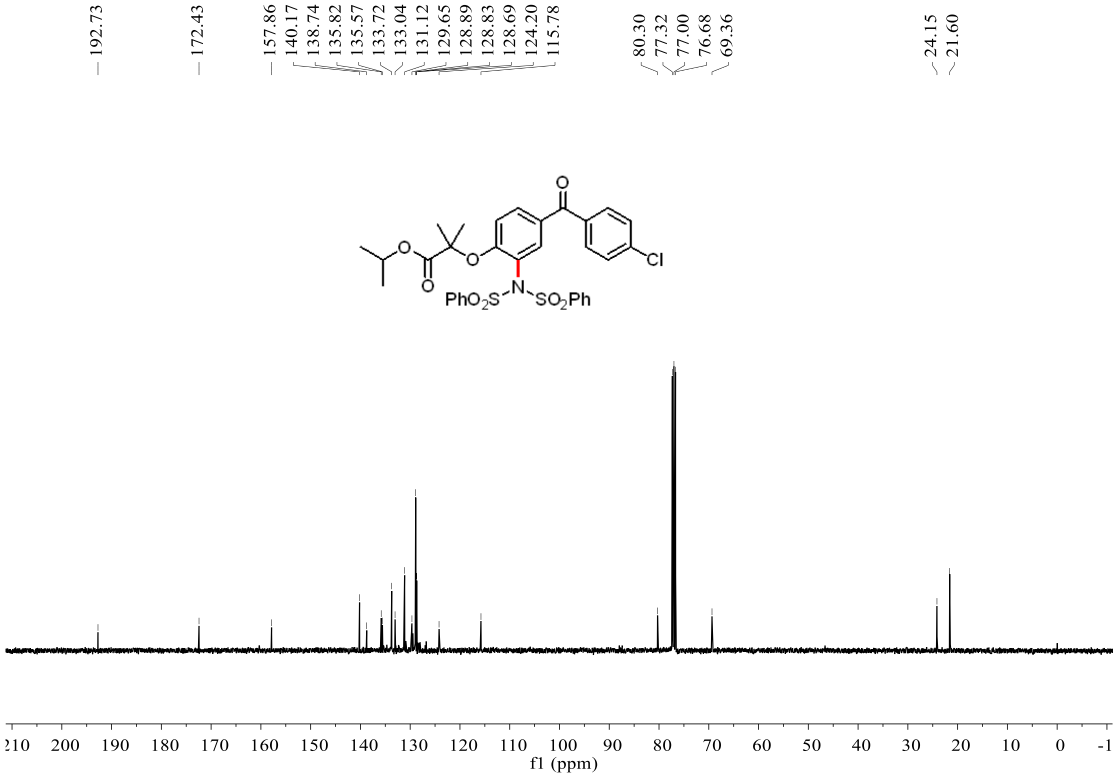

Supplementary Figure 74. ^{13}C NMR (101 MHz, $CDCl_3$) spectrum of **3bk**

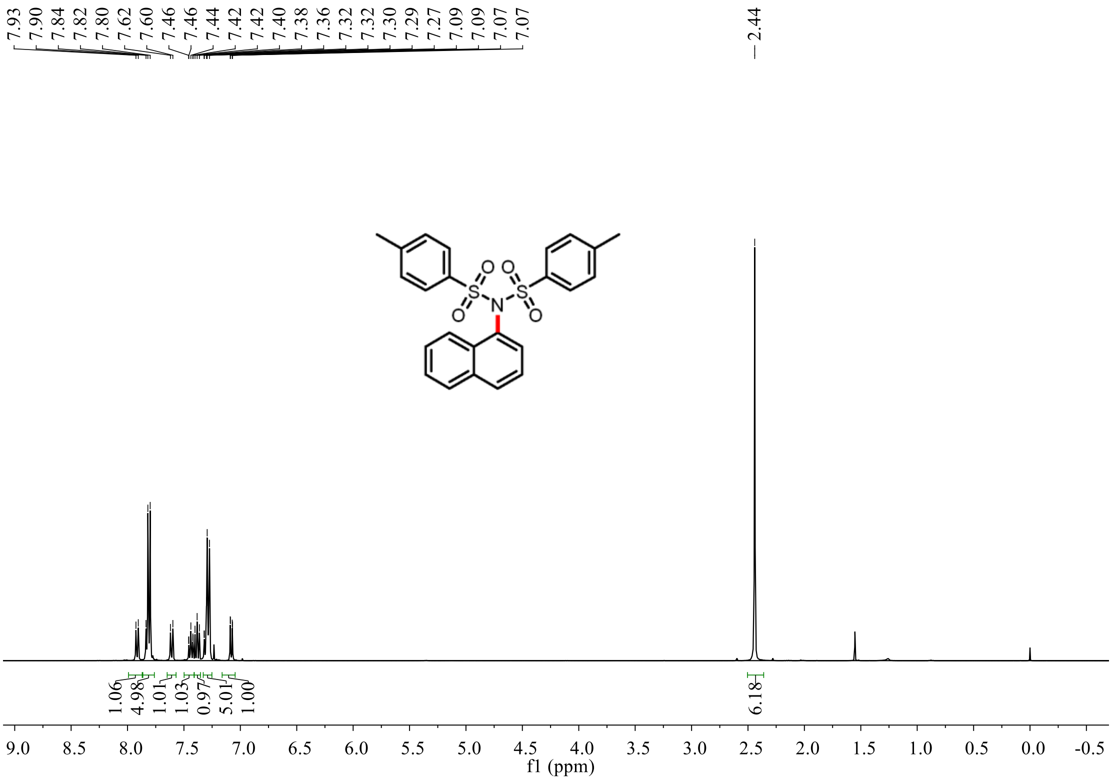

Supplementary Figure 75. ^{1}H NMR (400 MHz, $CDCl_3$) spectrum of **3bl**

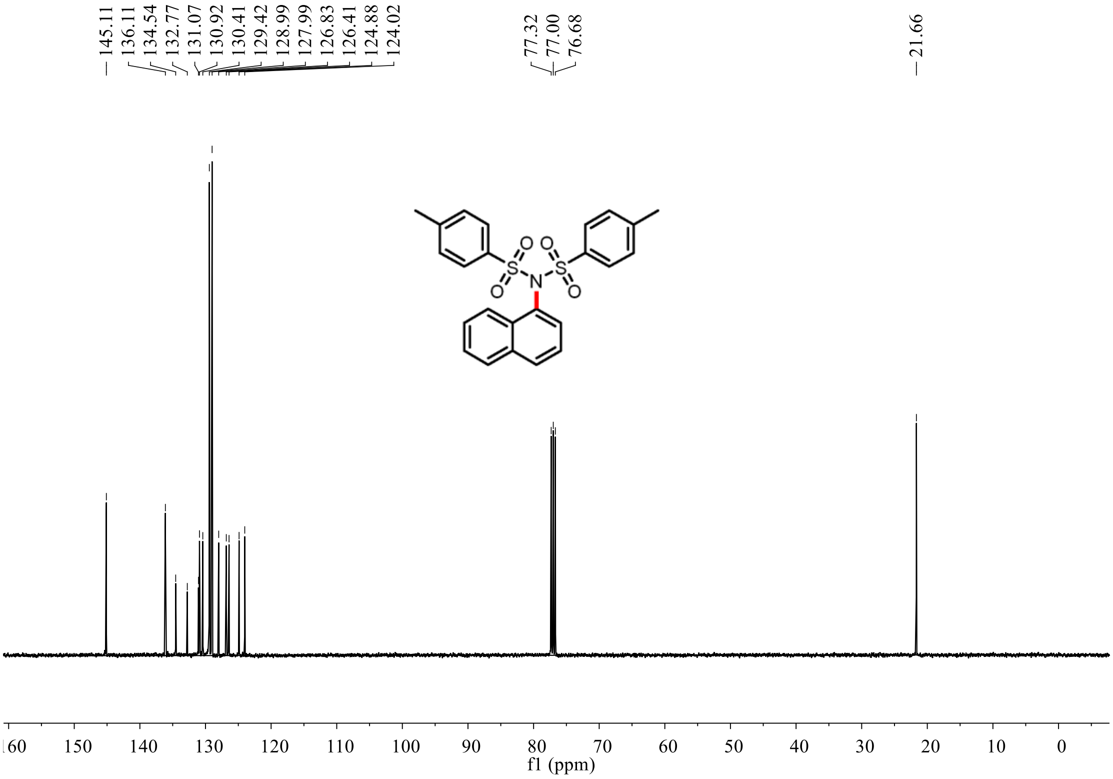

Supplementary Figure 76. ^{13}C NMR (101 MHz, $CDCl_3$) spectrum of **3bl**

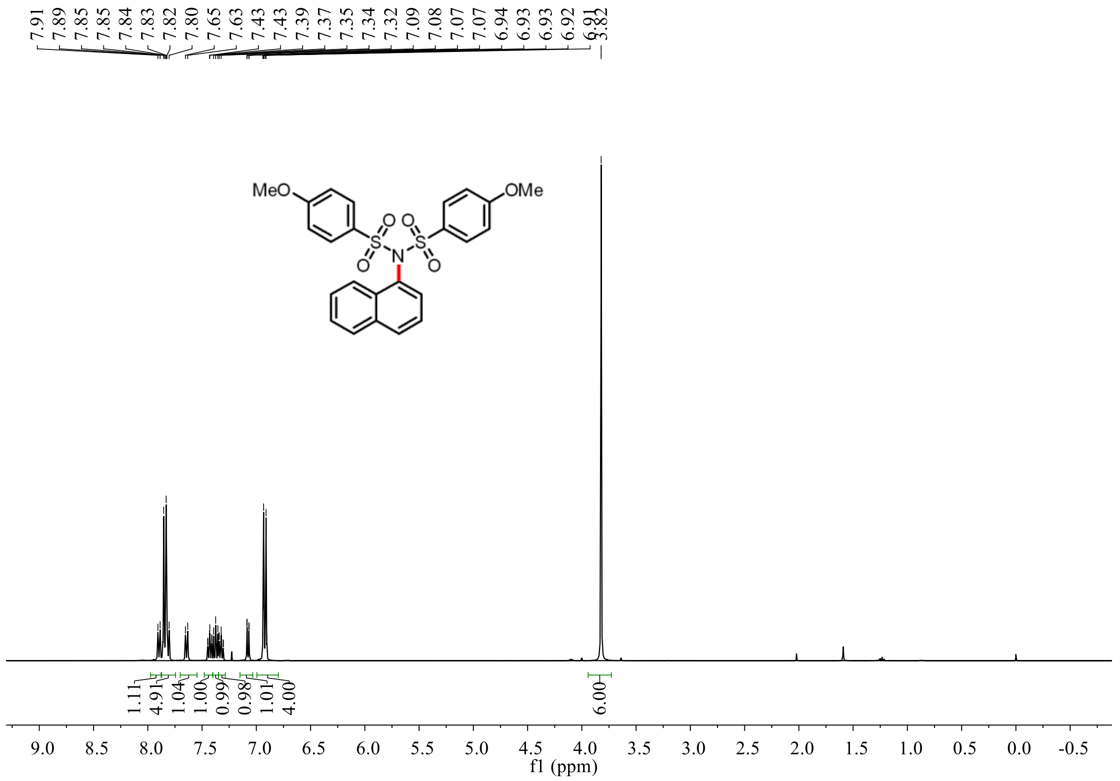

Supplementary Figure 77. ^{1}H NMR (400 MHz, $CDCl_3$) spectrum of **3bm**

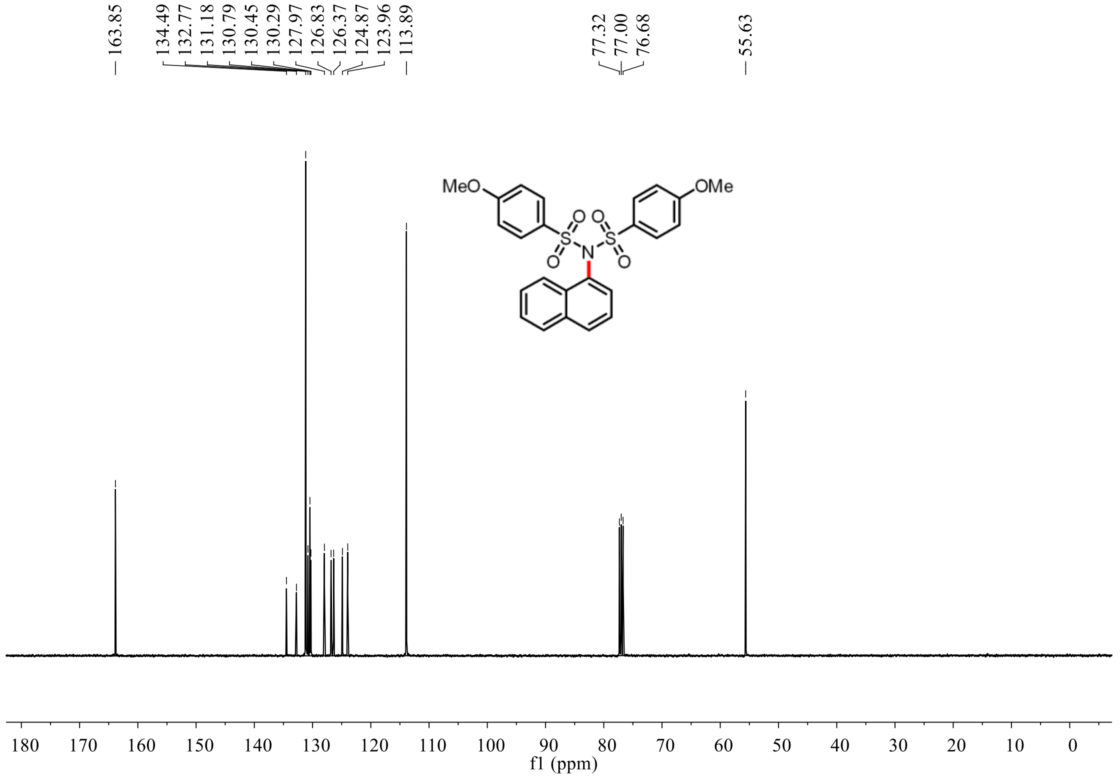

Supplementary Figure 78. ^{13}C NMR (101 MHz, $CDCl_3$) spectrum of **3bm**

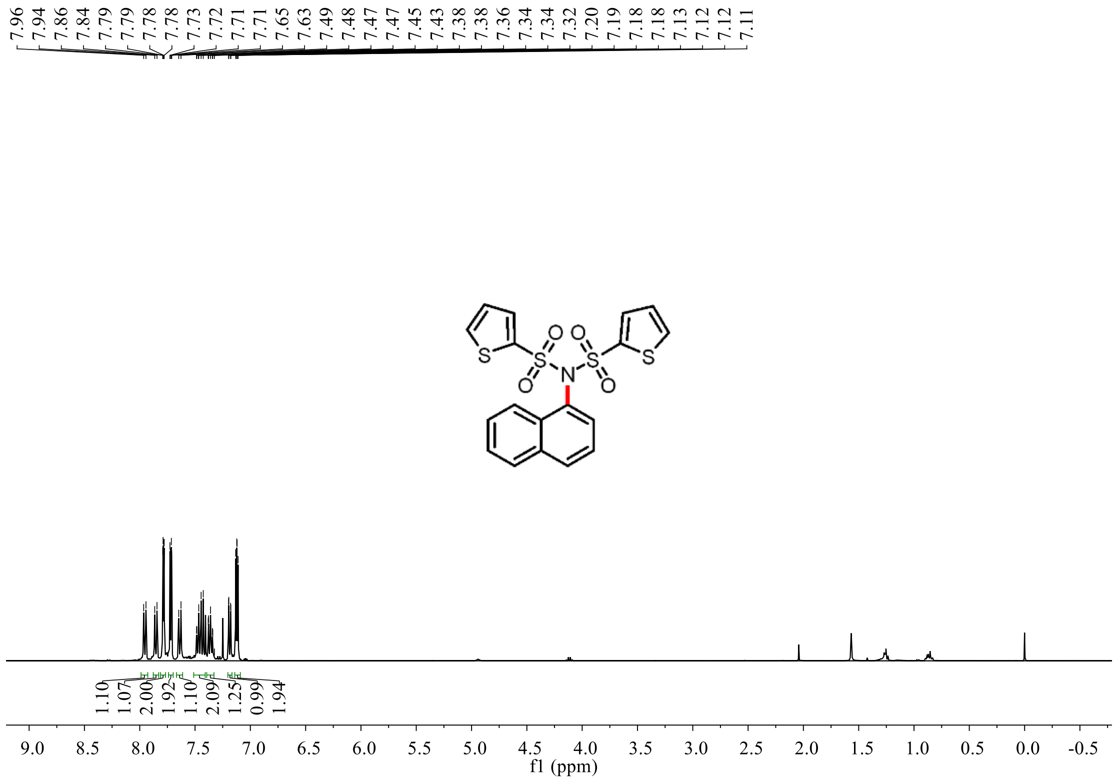

Supplementary Figure 79. ^{1}H NMR (400 MHz, $CDCl_3$) spectrum of **3bn**

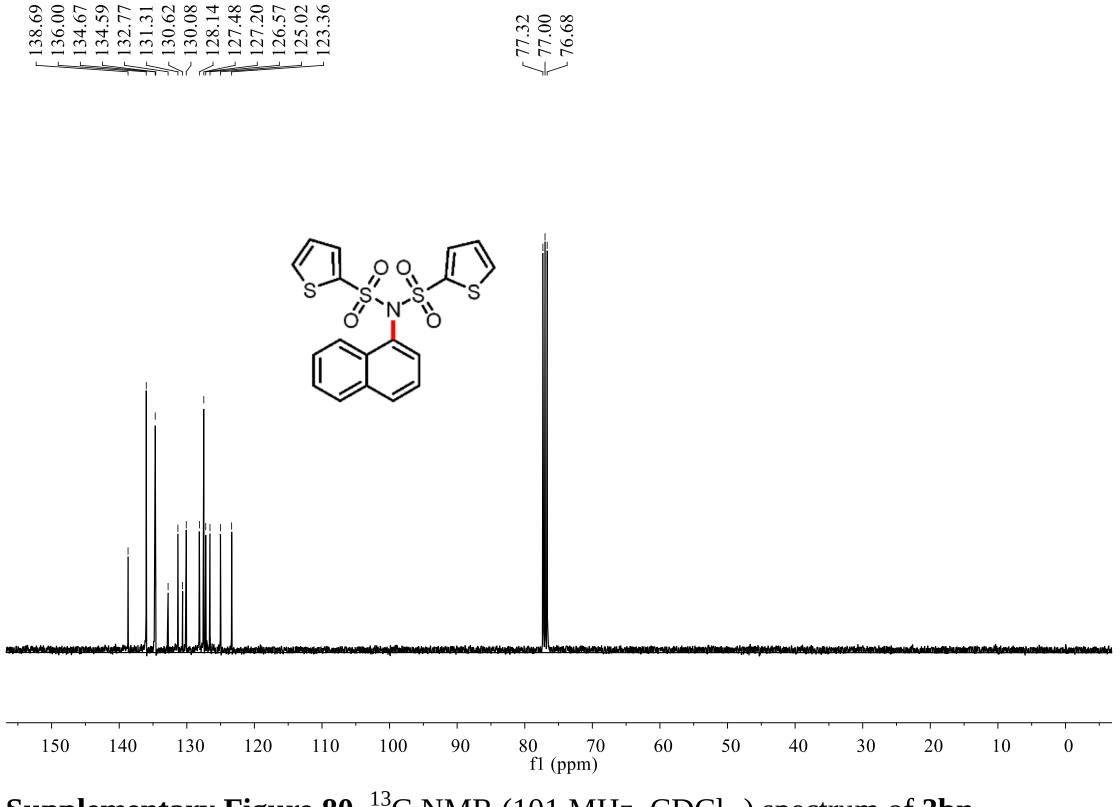

Supplementary Figure 80. ^{13}C NMR (101 MHz, $CDCl_3$) spectrum of **3bn**

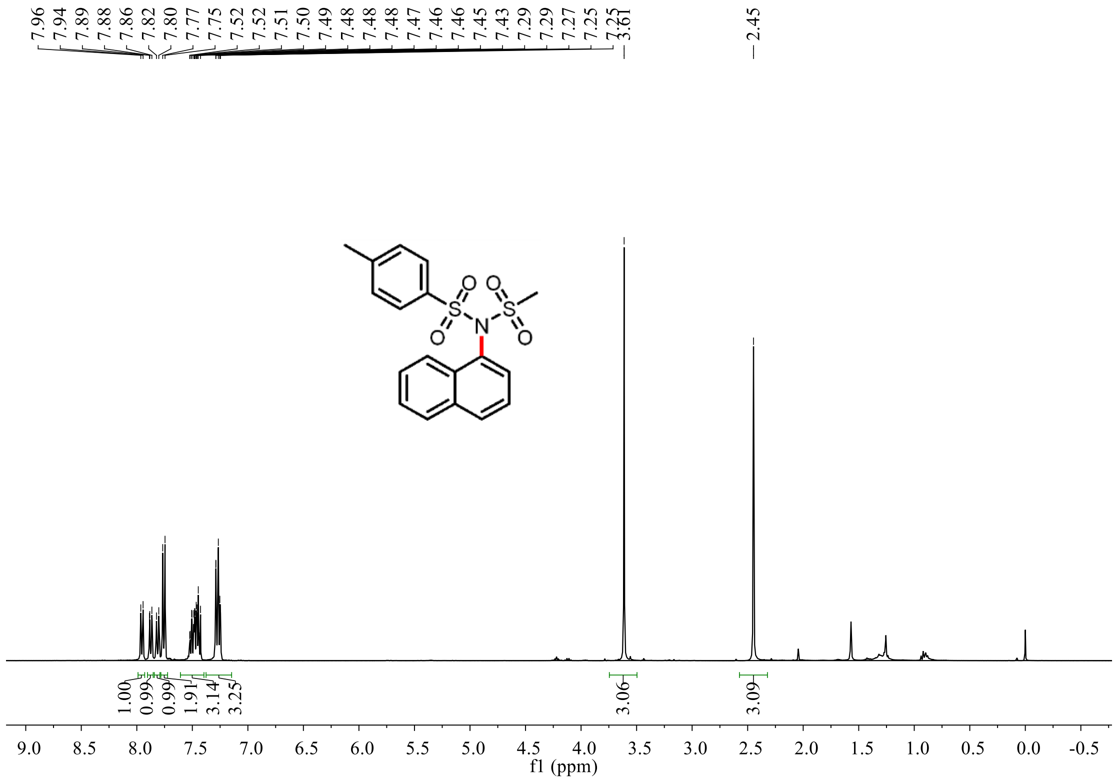

Supplementary Figure 81. ^{1}H NMR (400 MHz, $CDCl_3$) spectrum of **3bo**

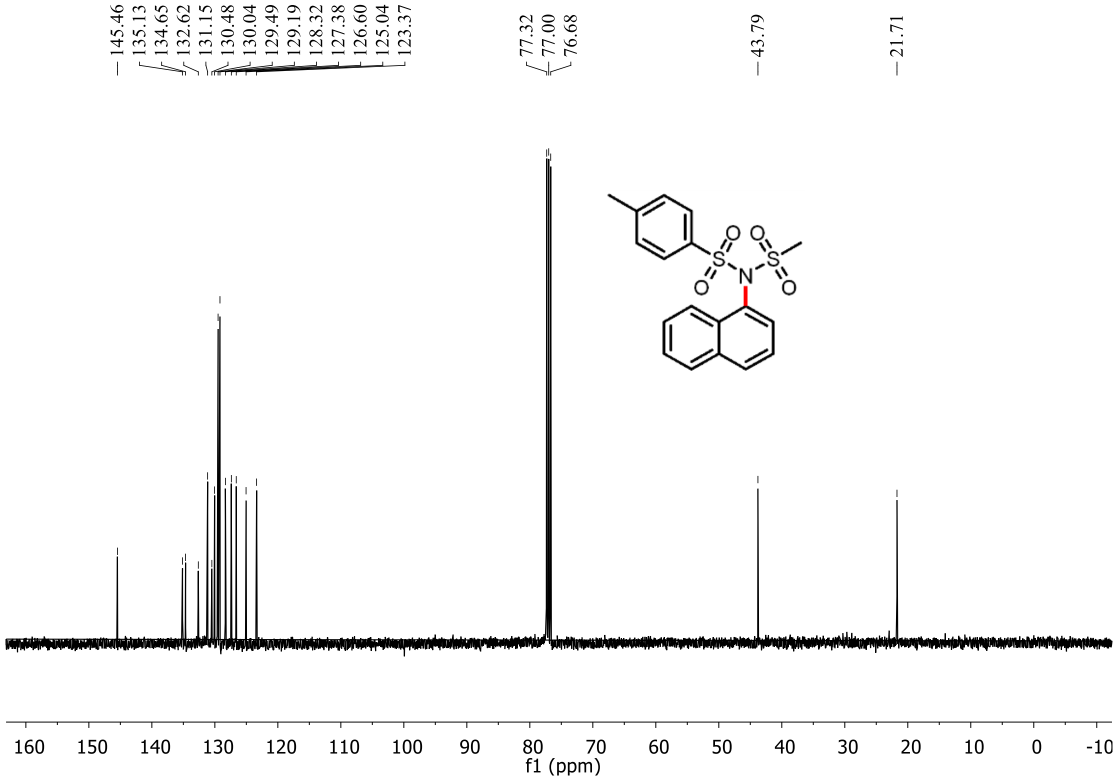

Supplementary Figure 82. ^{13}C NMR (101 MHz, $CDCl_3$) spectrum of **3bo**

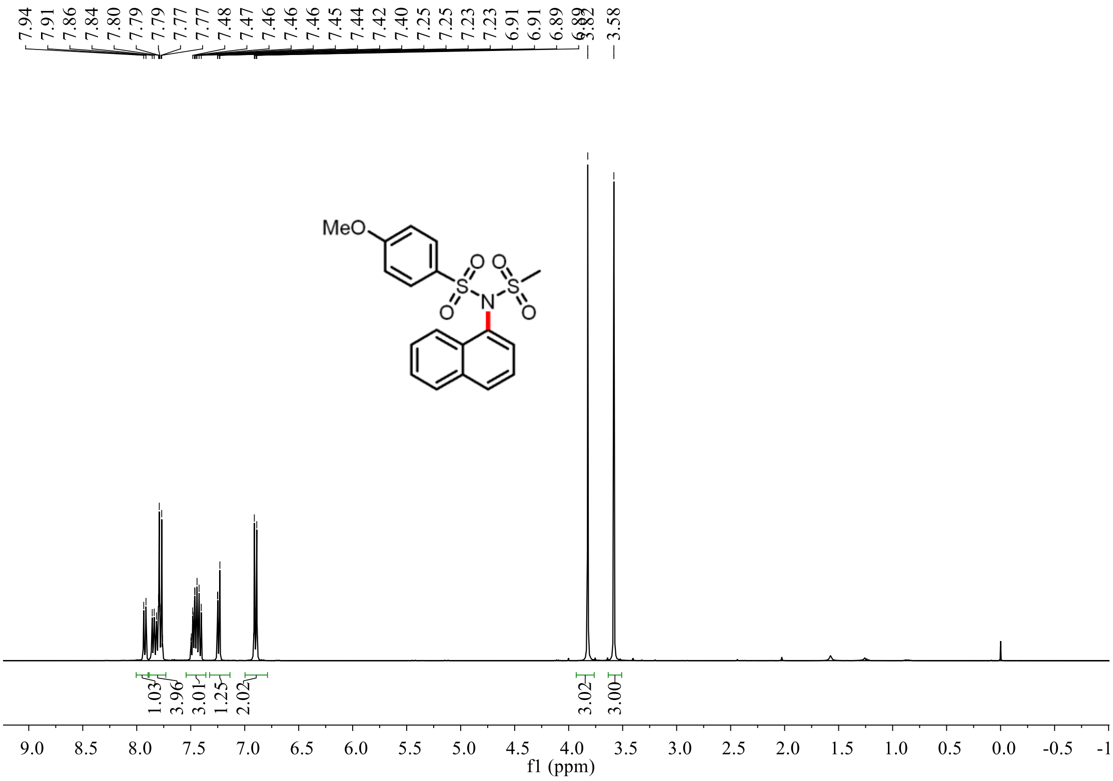

Supplementary Figure 83. ^{1}H NMR (400 MHz, $CDCl_3$) spectrum of **3bp**

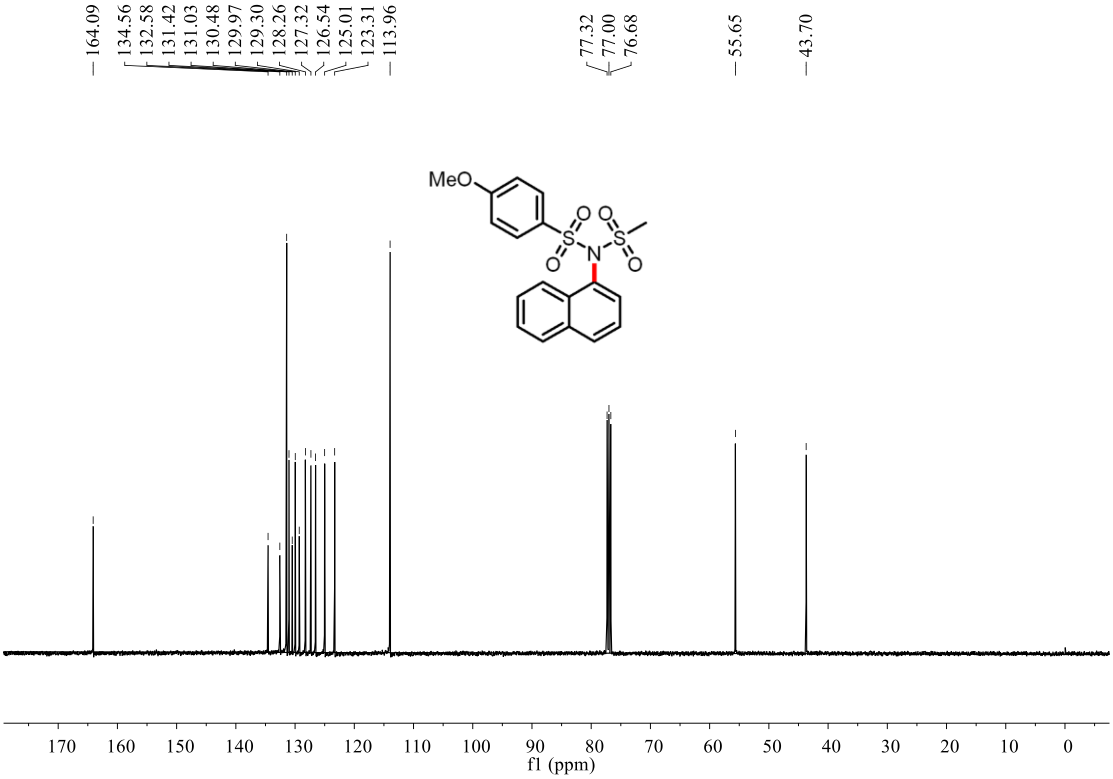

Supplementary Figure 84. ^{13}C NMR (101 MHz, $CDCl_3$) spectrum of **3bp**

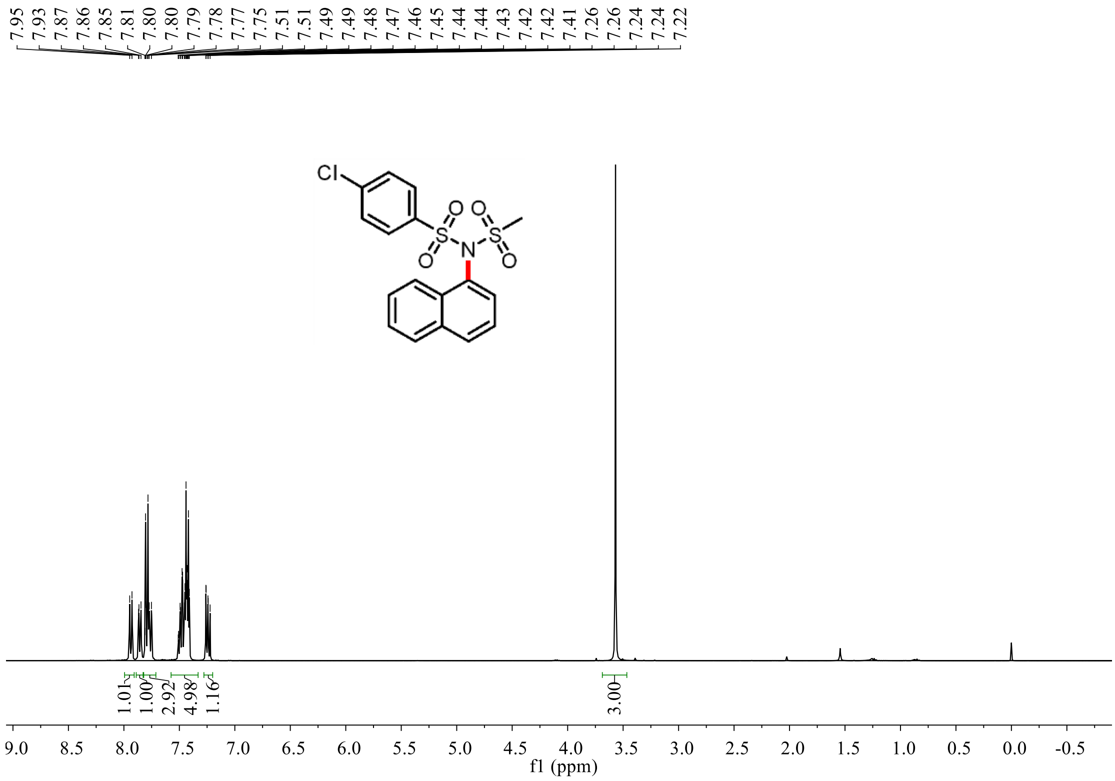

Supplementary Figure 85. ^{1}H NMR (400 MHz, $CDCl_3$) spectrum of **3bq**

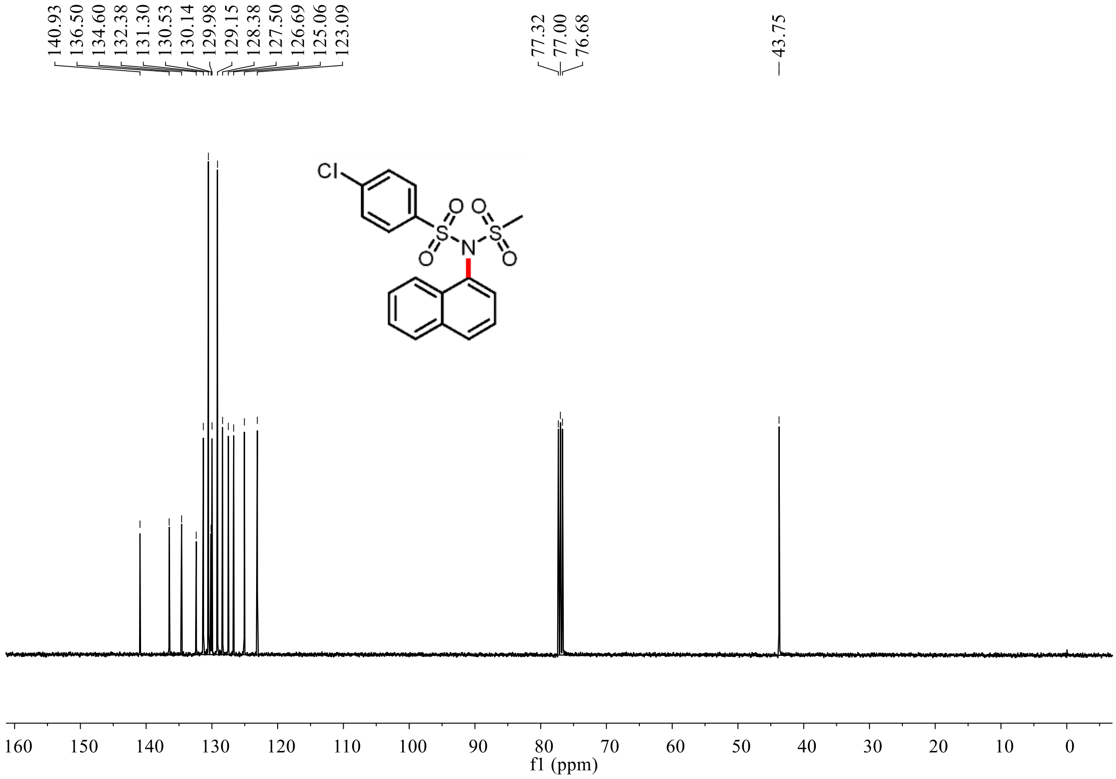

Supplementary Figure 86. ^{13}C NMR (101 MHz, $CDCl_3$) spectrum of **3bq**

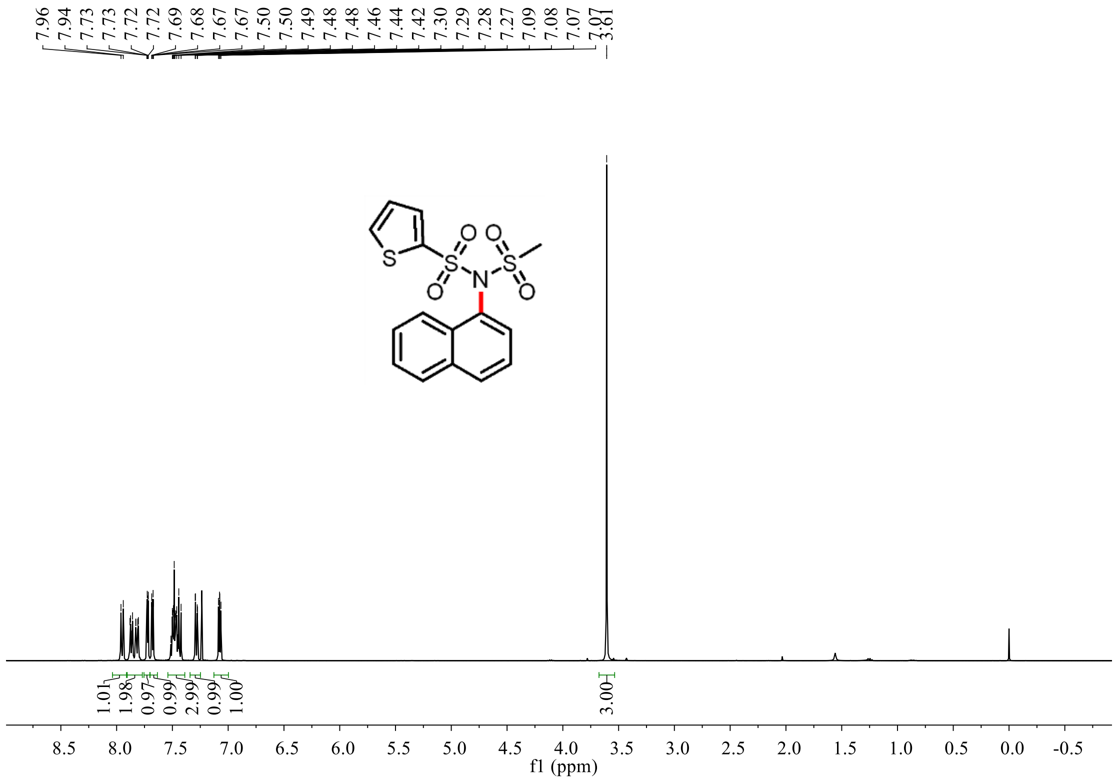

Supplementary Figure 87. ^{1}H NMR (400 MHz, $CDCl_3$) spectrum of **3br**

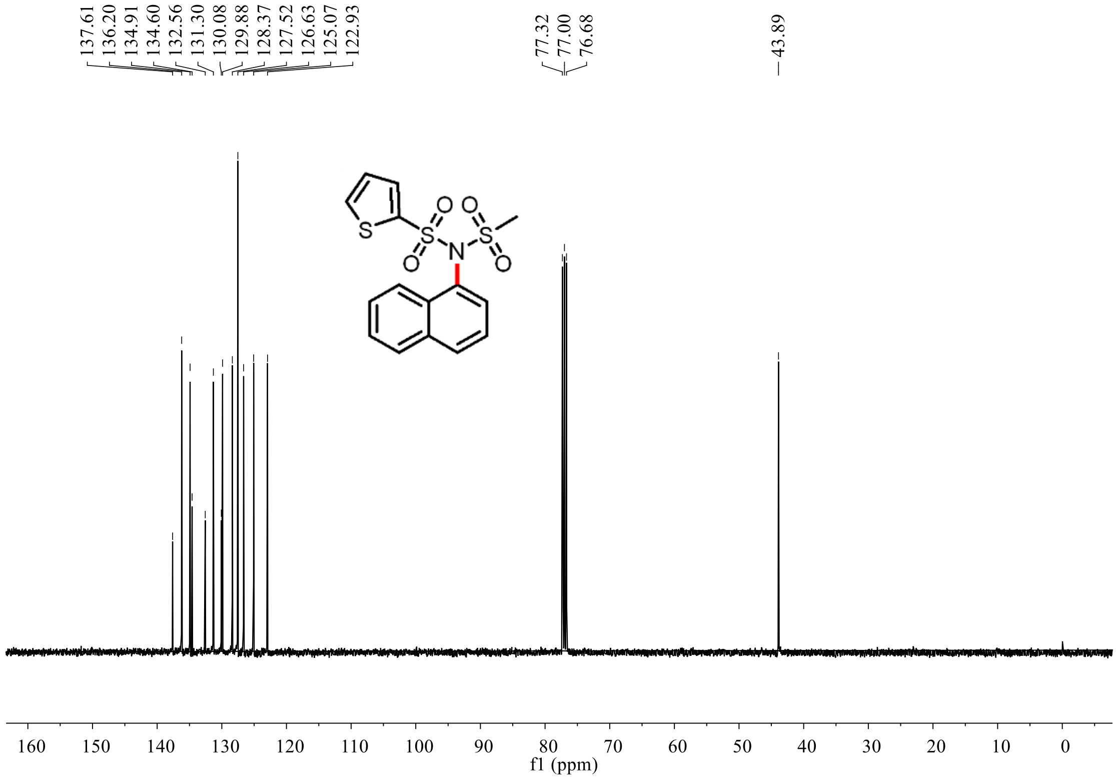

Supplementary Figure 88. ^{13}C NMR (101 MHz, $CDCl_3$) spectrum of **3br**

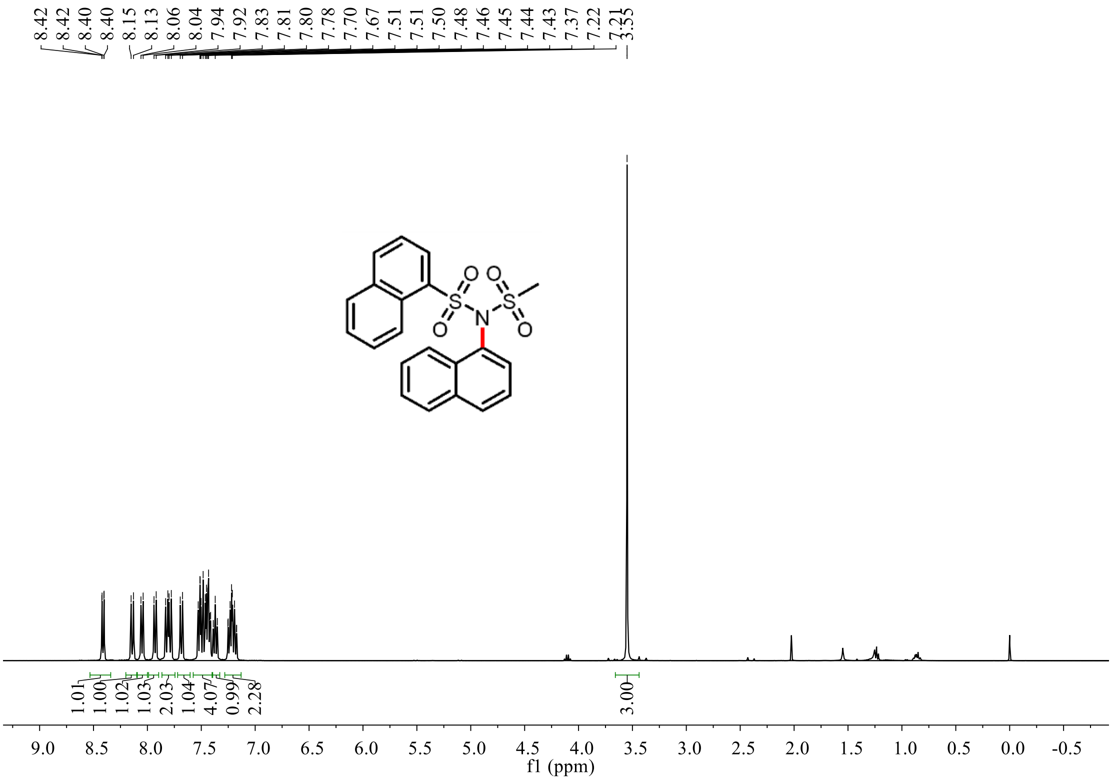

Supplementary Figure 89. ^{1}H NMR (400 MHz, $CDCl_3$) spectrum of **3bs**

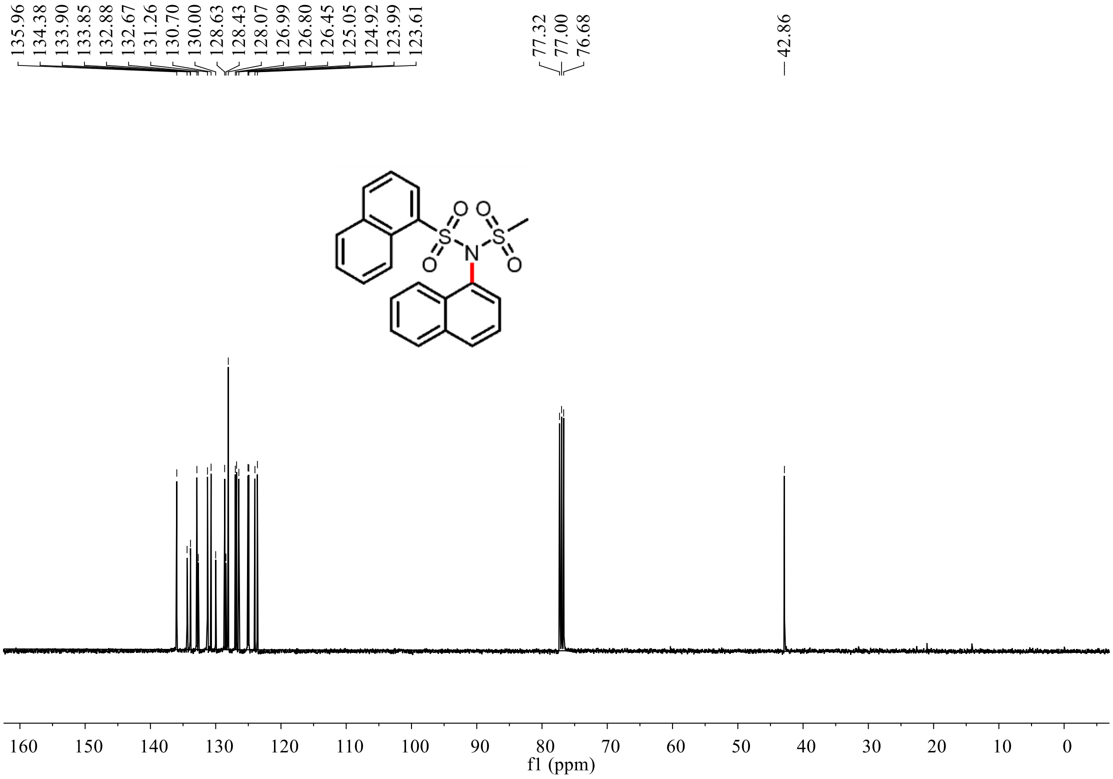

Supplementary Figure 90. ^{13}C NMR (101 MHz, $CDCl_3$) spectrum of **3bs**

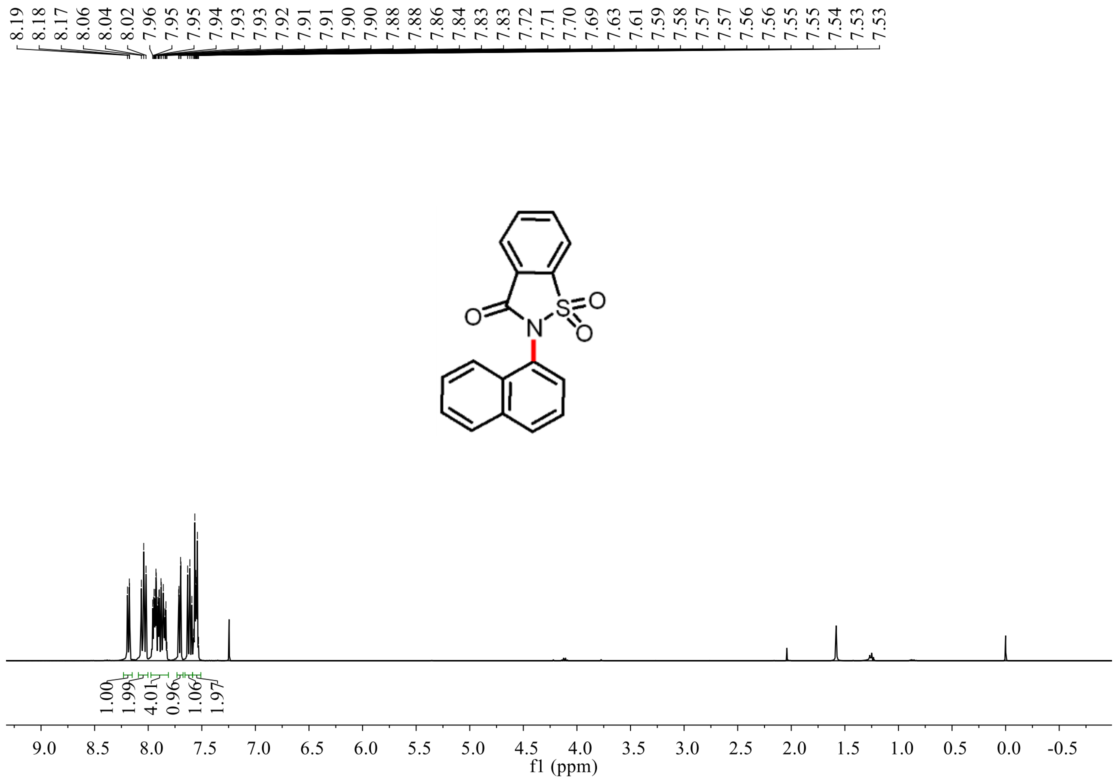

Supplementary Figure 91. ^{1}H NMR (400 MHz, $CDCl_3$) spectrum of **3bt**

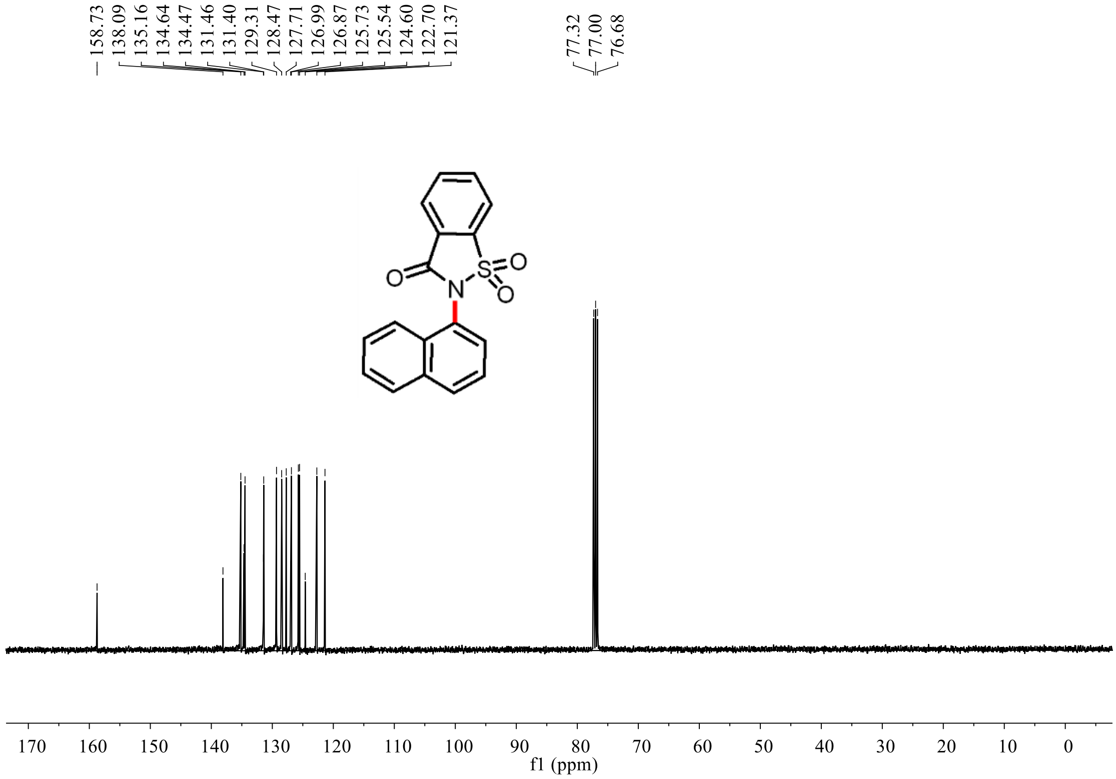

Supplementary Figure 92. ^{13}C NMR (101 MHz, $CDCl_3$) spectrum of **3bt**

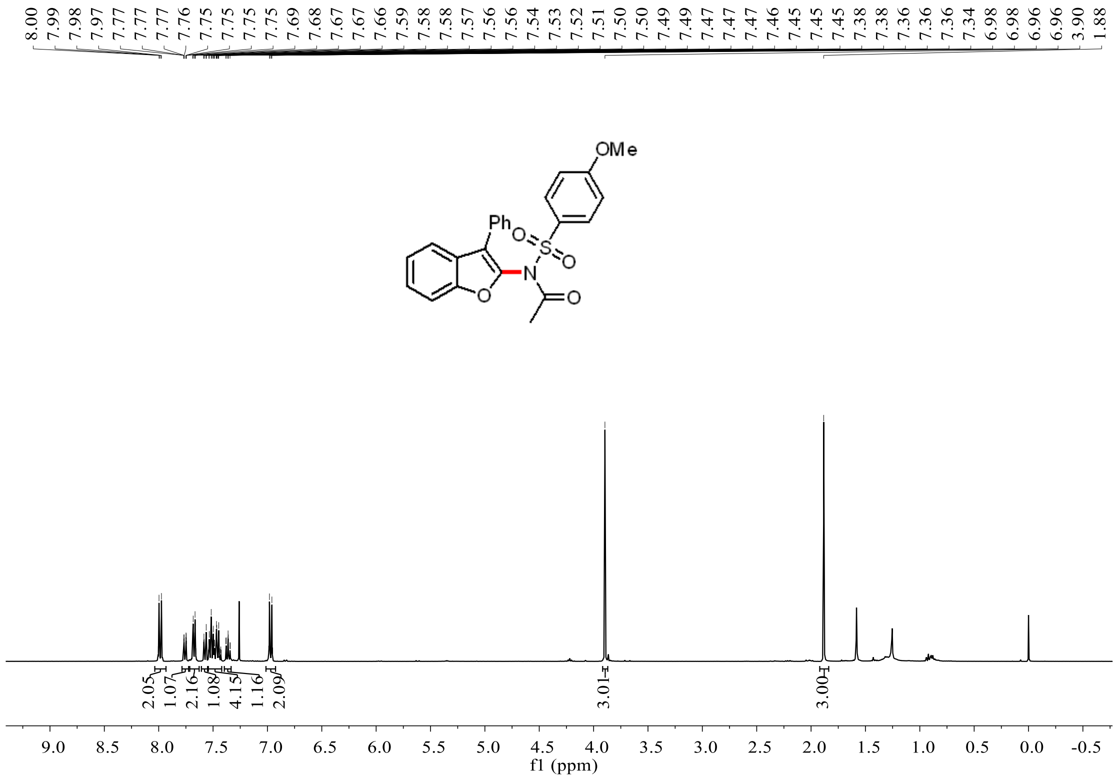

Supplementary Figure 93. ^{1}H NMR (400 MHz, $CDCl_3$) spectrum of **3bu**

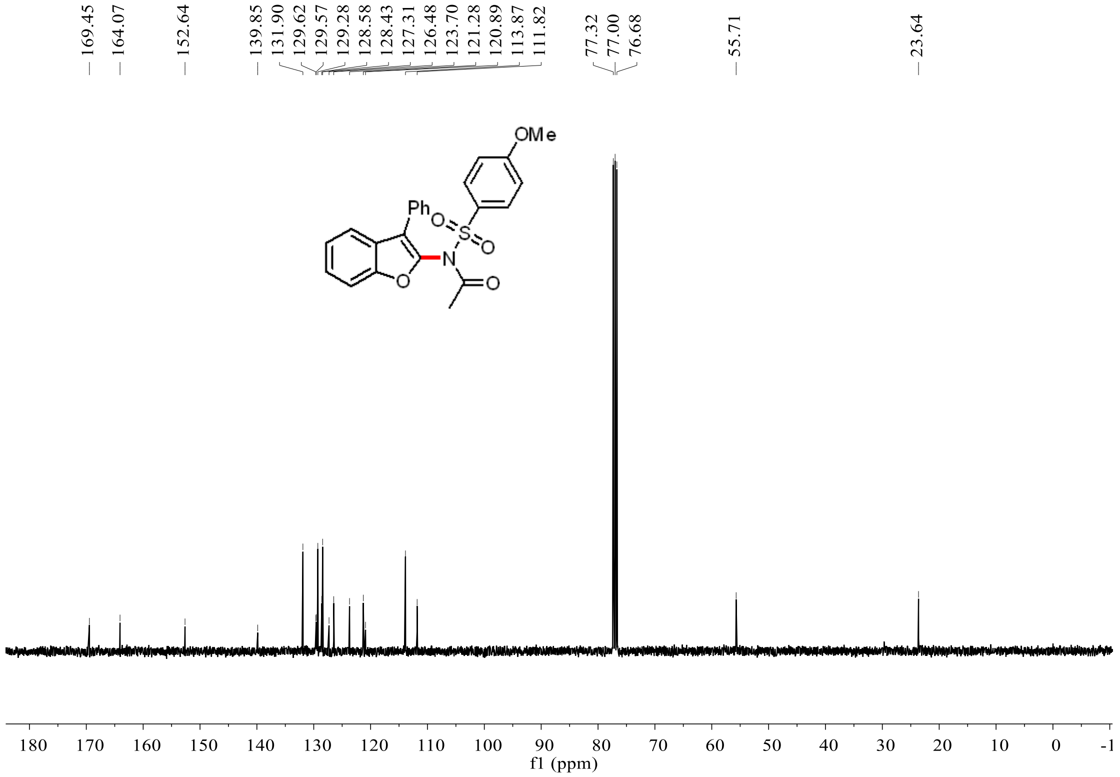

Supplementary Figure 94. ^{13}C NMR (101 MHz, $CDCl_3$) spectrum of **3bu**

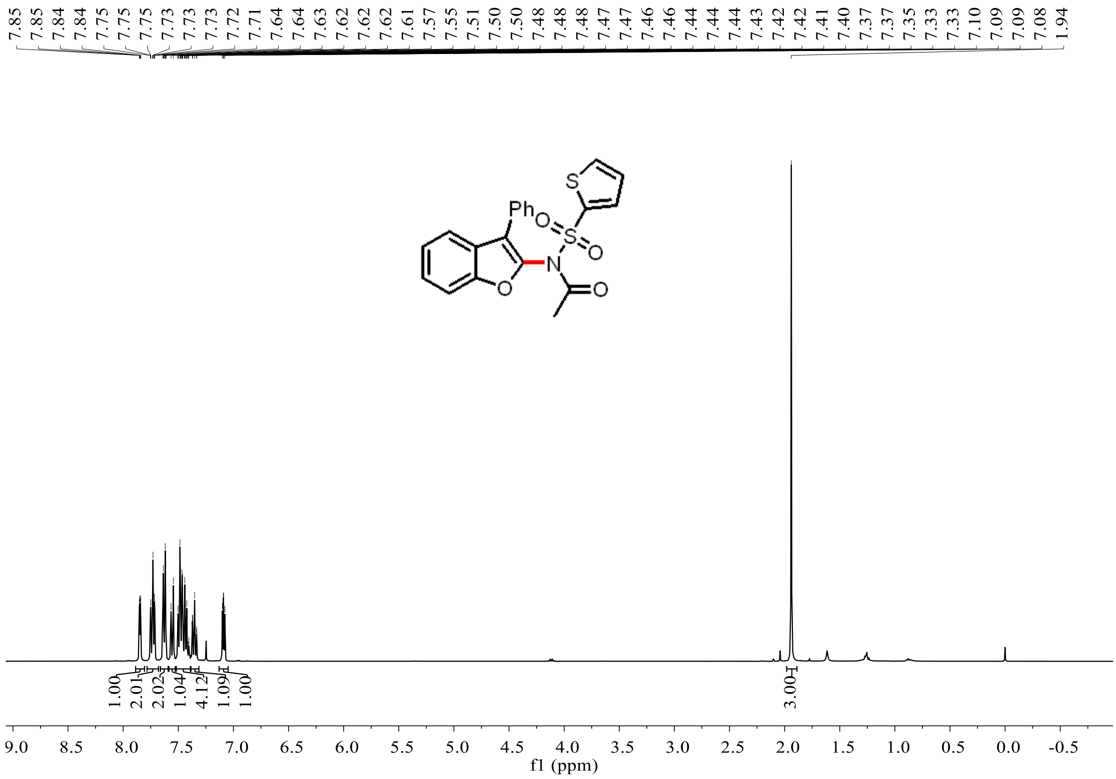

Supplementary Figure 95. ^{1}H NMR (400 MHz, $CDCl_3$) spectrum of **3bv**

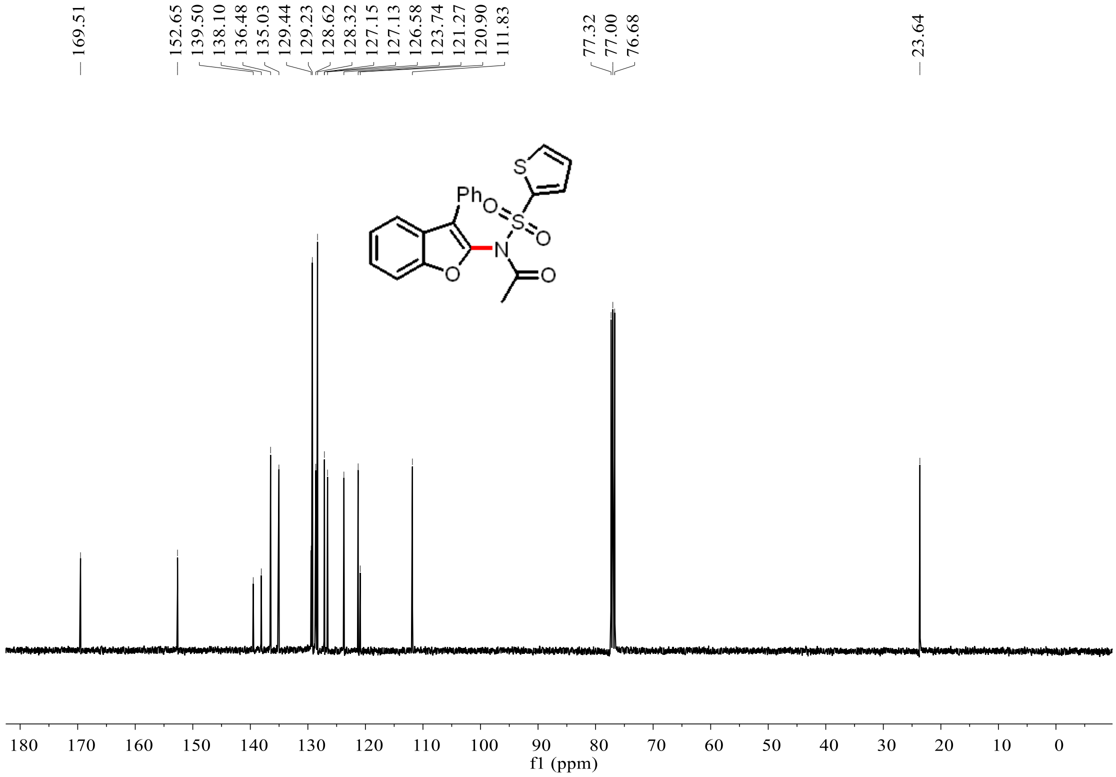

Supplementary Figure 96. ^{13}C NMR (101 MHz, $CDCl_3$) spectrum of **3bv**

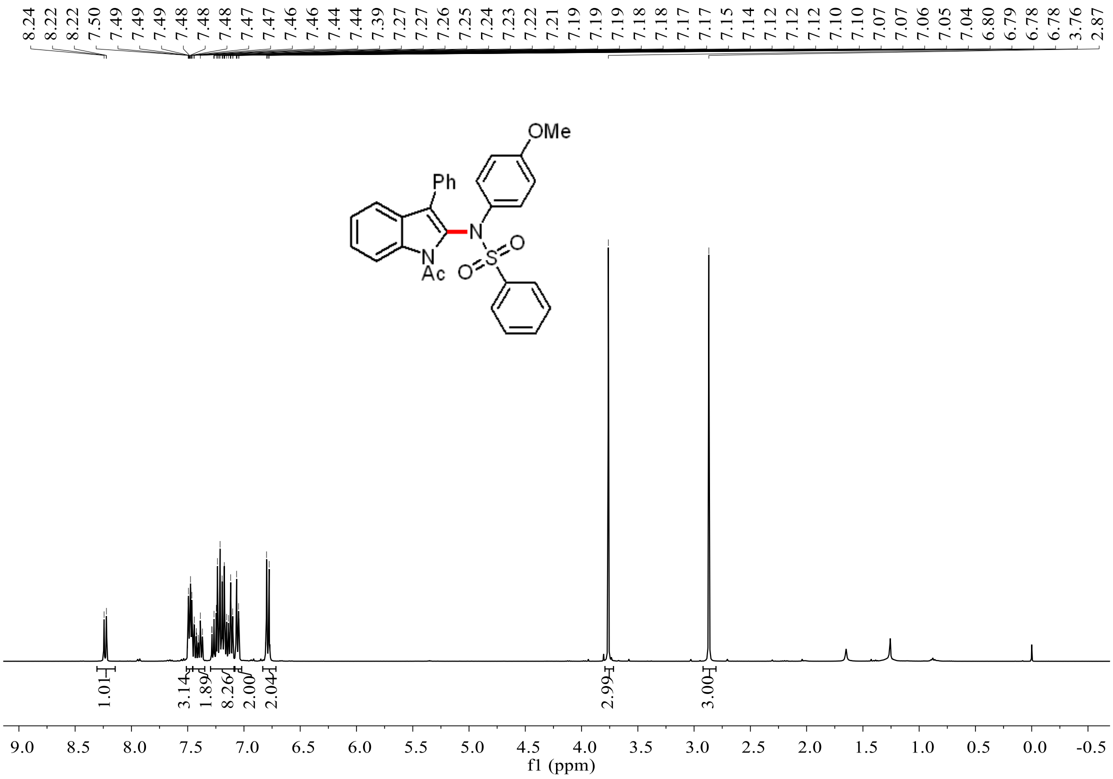

Supplementary Figure 97. ^{1}H NMR (400 MHz, $CDCl_3$) spectrum of **5aa**

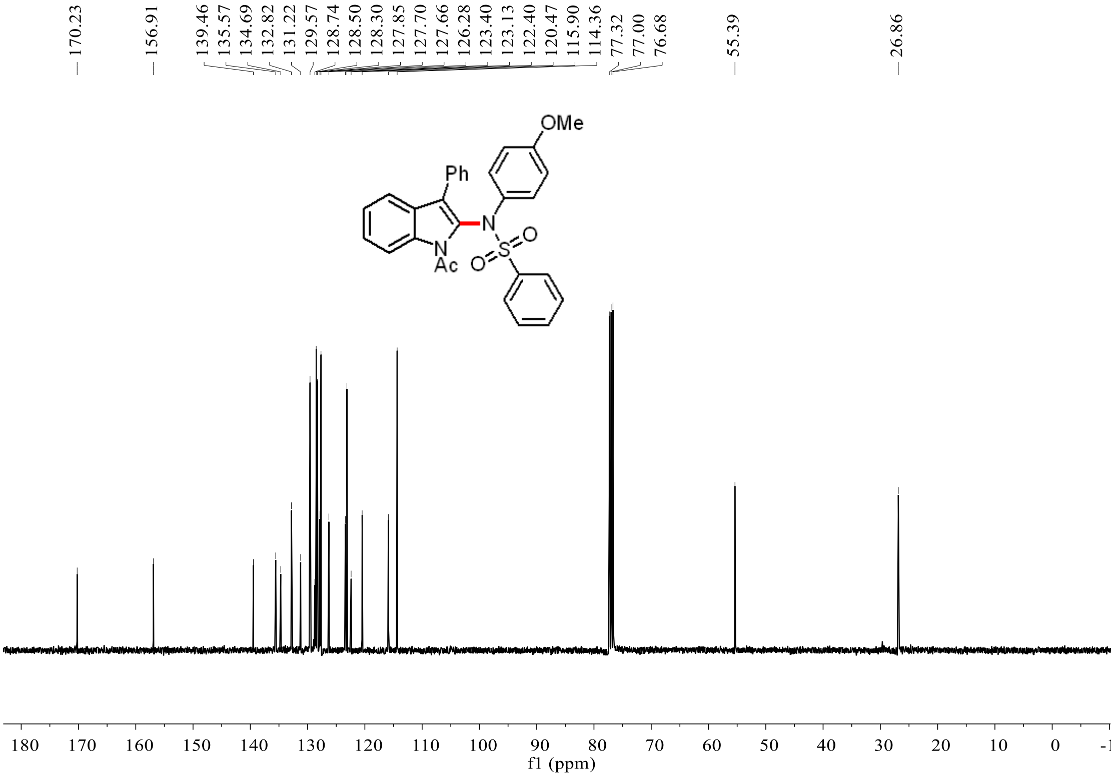

Supplementary Figure 98. ^{13}C NMR (101 MHz, $CDCl_3$) spectrum of **5aa**

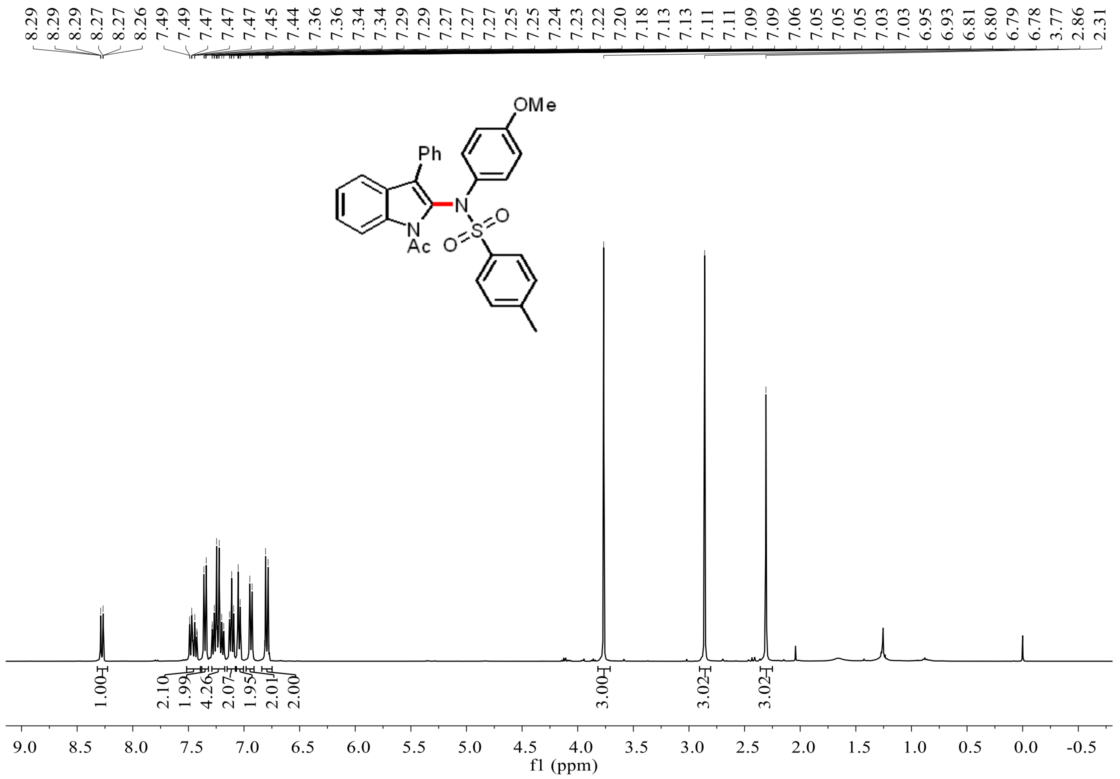

Supplementary Figure 99. ^{1}H NMR (400 MHz, $CDCl_3$) spectrum of **5ab**

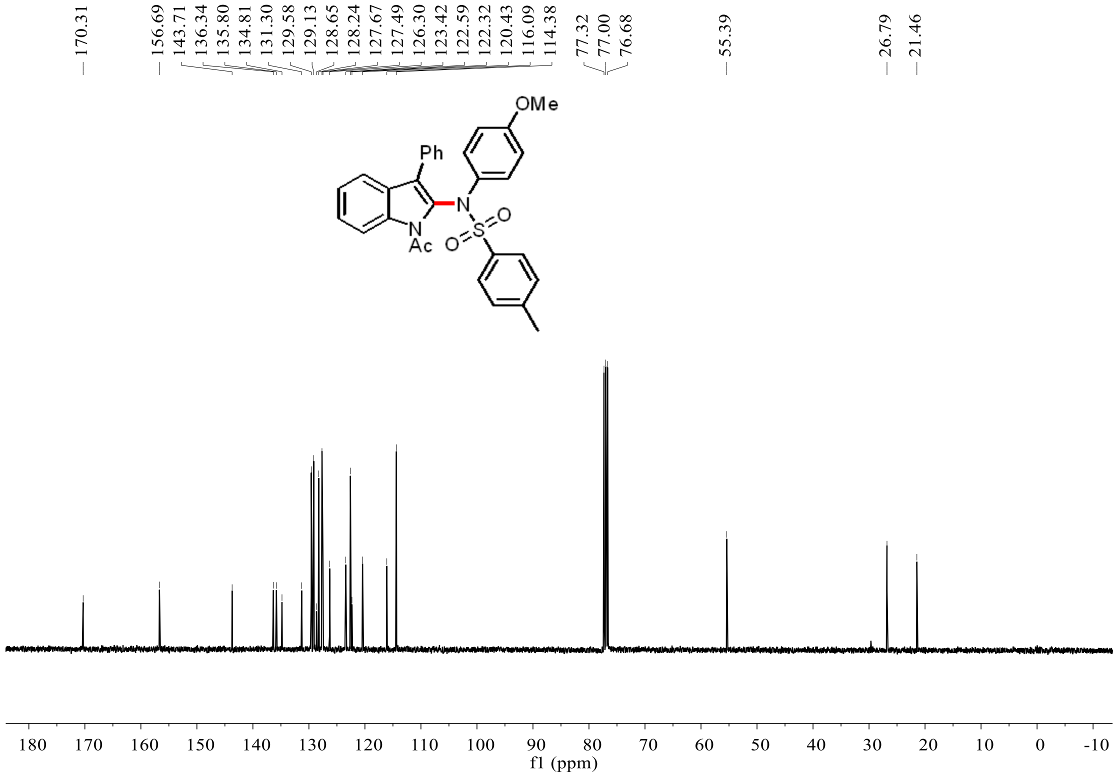

Supplementary Figure 100. ^{13}C NMR (101 MHz, $CDCl_3$) spectrum of **5ab**

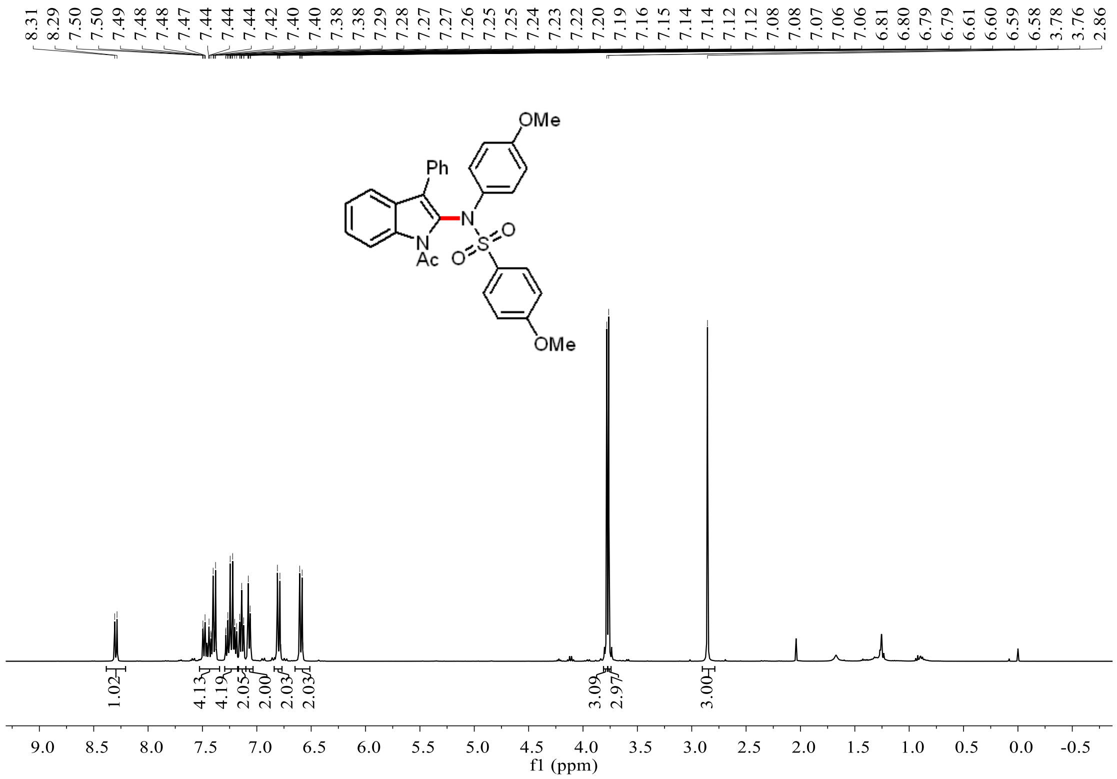

Supplementary Figure 101. ^{1}H NMR (400 MHz, $CDCl_3$) spectrum of **5ac**

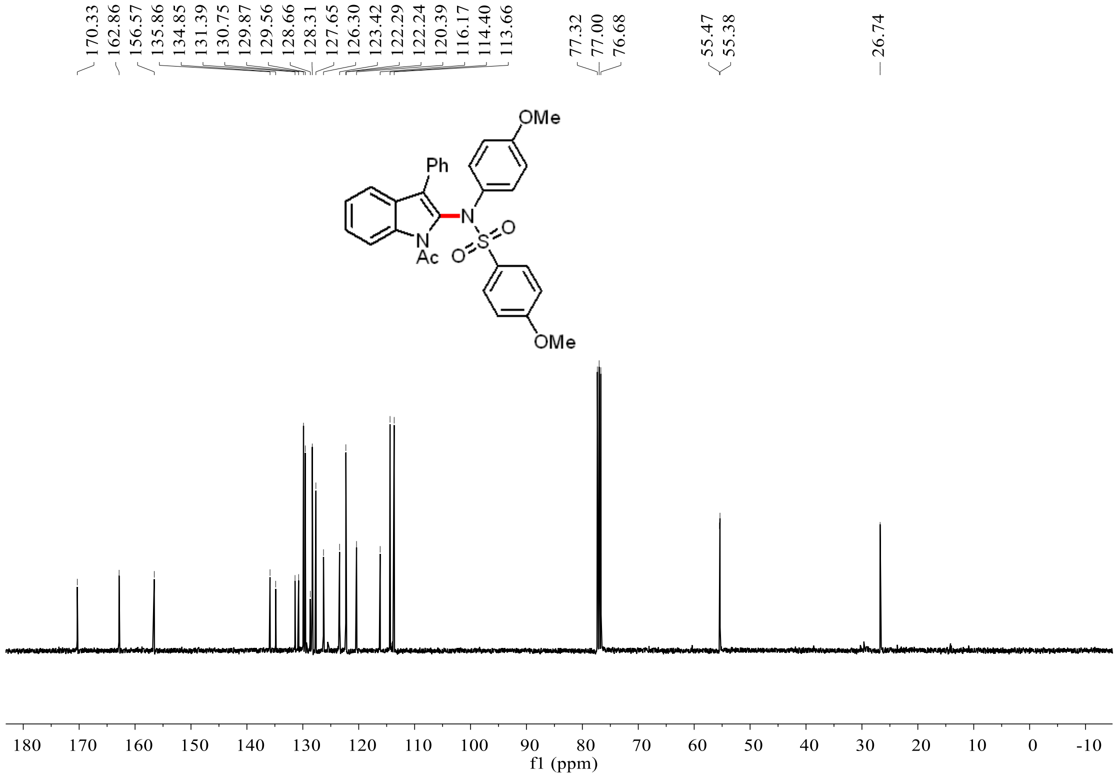

Supplementary Figure 102. ^{13}C NMR (101 MHz, $CDCl_3$) spectrum of **5ac**

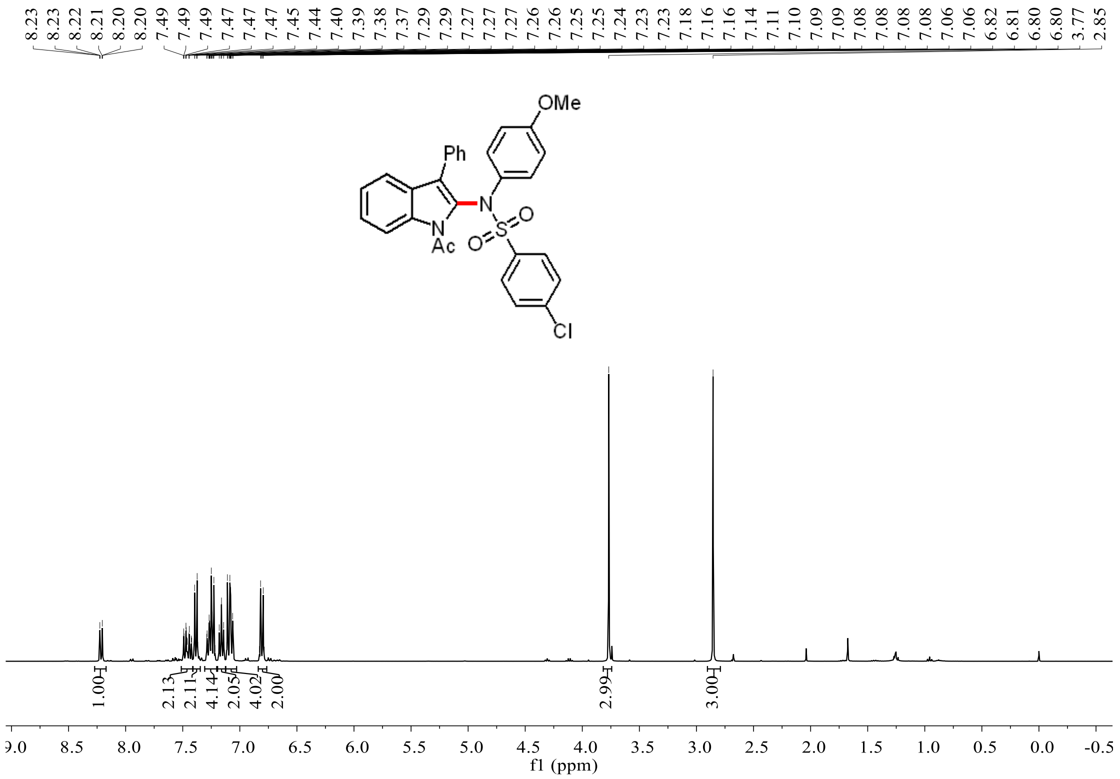

Supplementary Figure 103. ^{1}H NMR (400 MHz, $CDCl_3$) spectrum of **5ad**

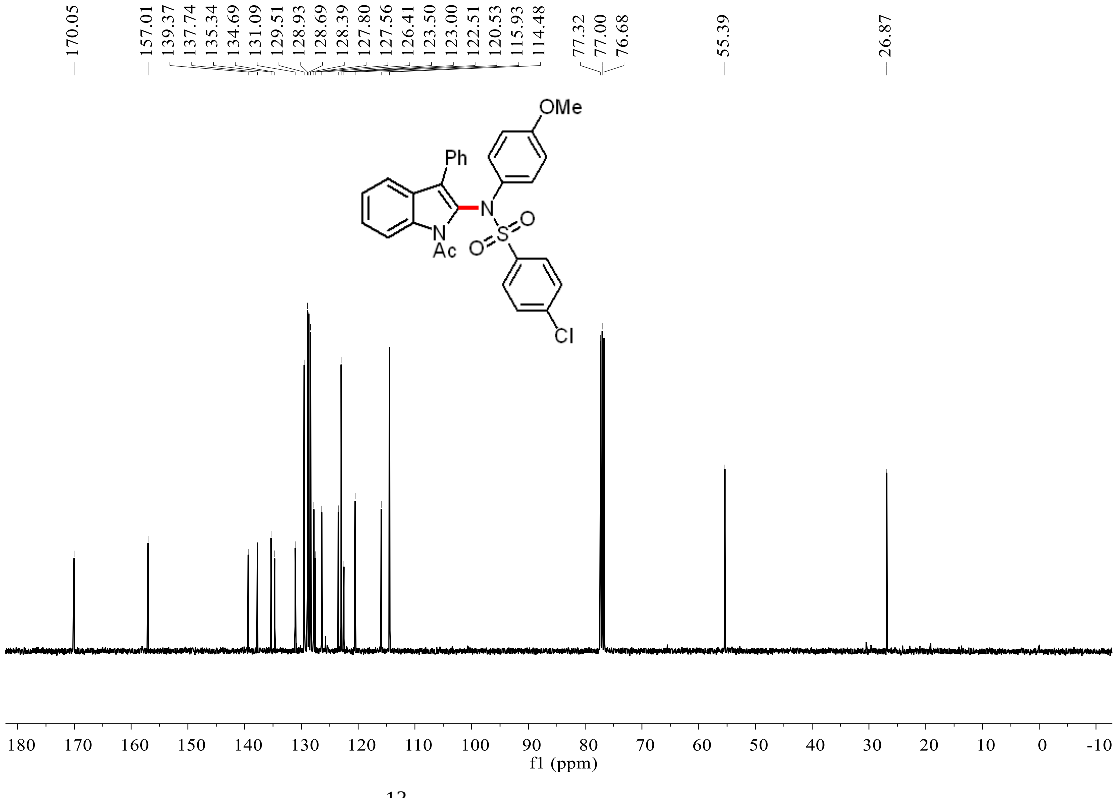

Supplementary Figure 104. ^{13}C NMR (101 MHz, $CDCl_3$) spectrum of **5ad**

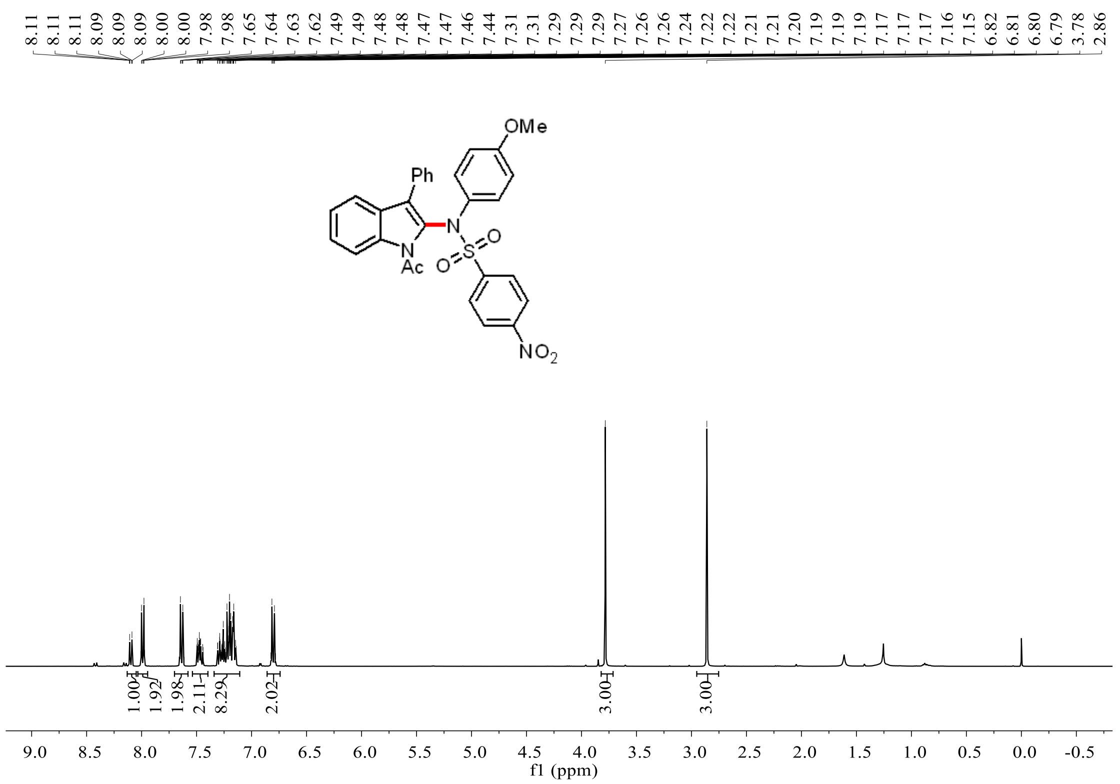

Supplementary Figure 105. ^{1}H NMR (400 MHz, $CDCl_3$) spectrum of **5ae**

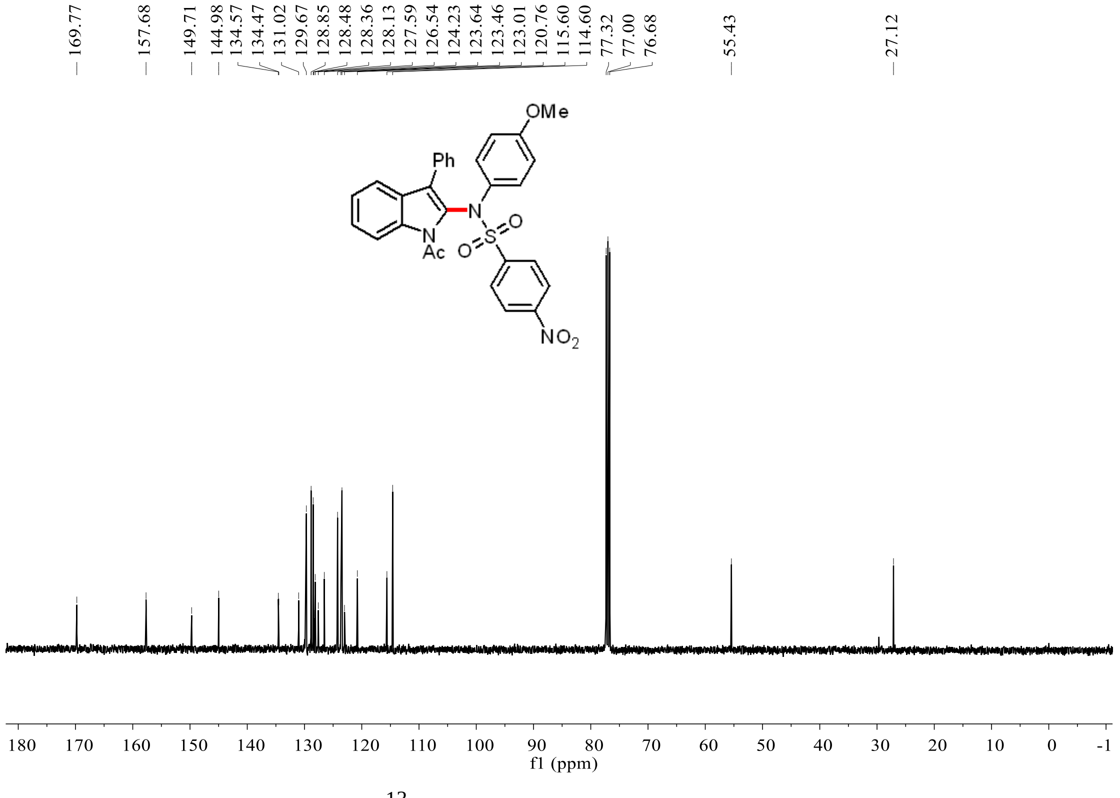

Supplementary Figure 106. ^{13}C NMR (101MHz, $CDCl_3$) spectrum of **5ae**

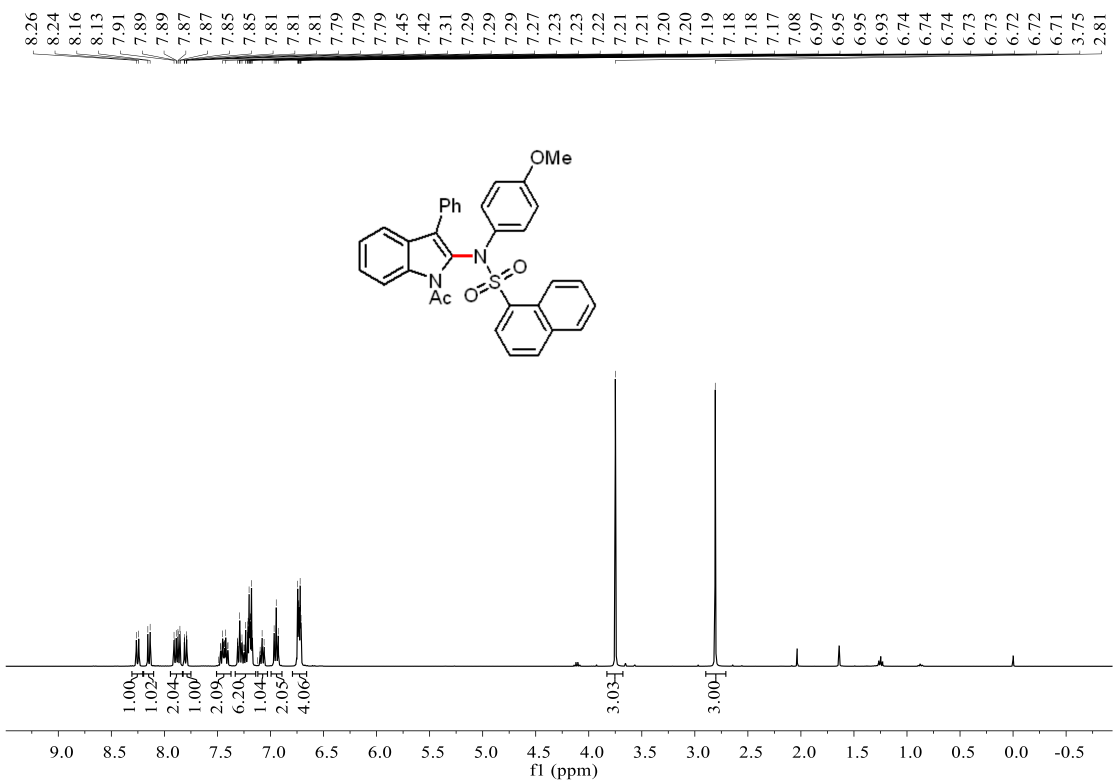

Supplementary Figure 107. ^{1}H NMR (400 MHz, $CDCl_3$) spectrum of **5af**

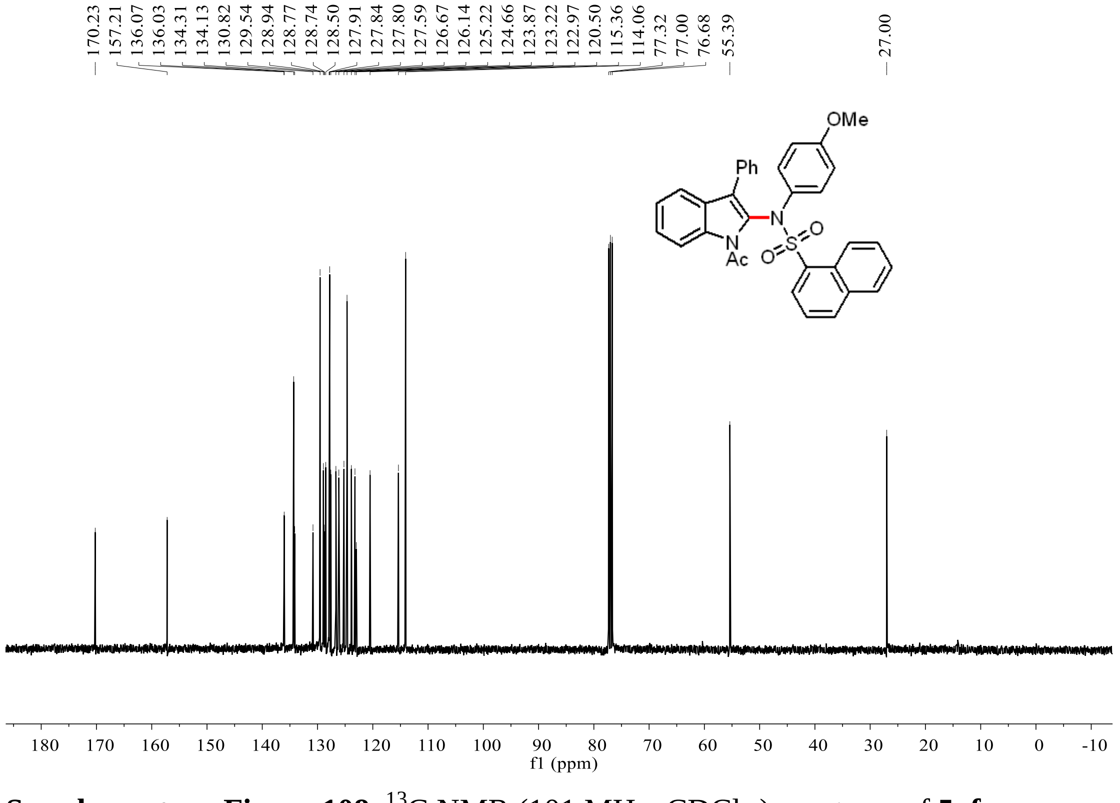

Supplementary Figure 108. ^{13}C NMR (101 MHz, $CDCl_3$) spectrum of **5af**

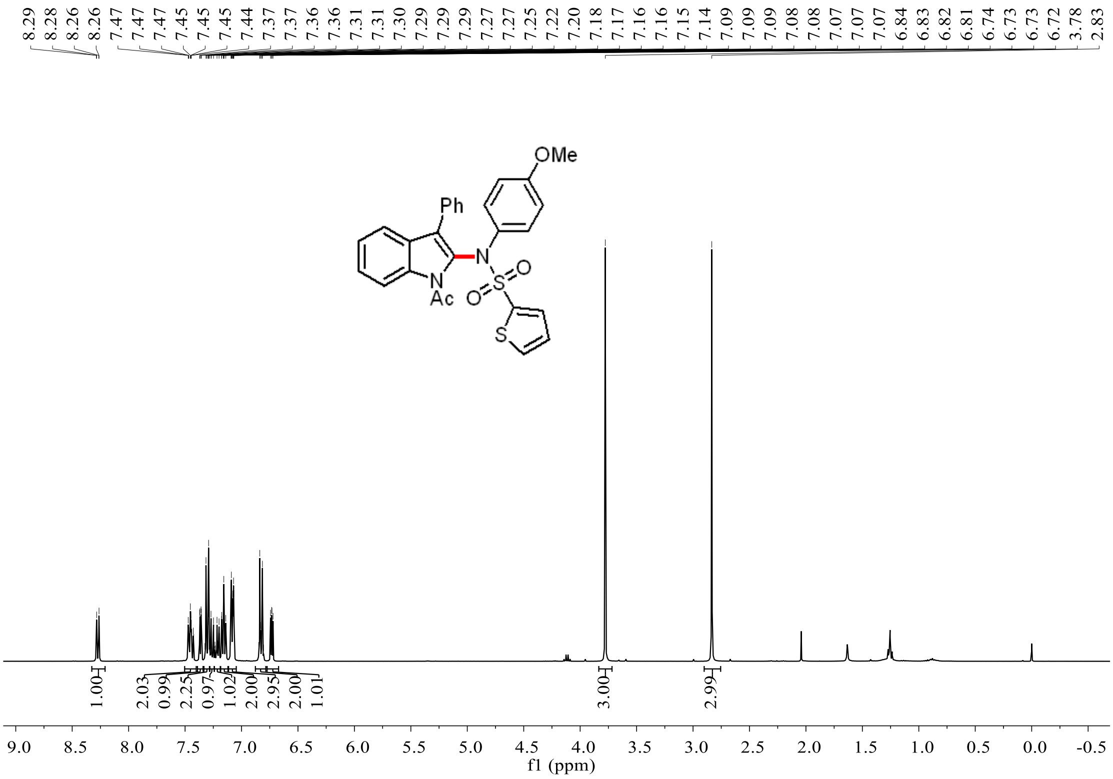

Supplementary Figure 109. ^{1}H NMR (400 MHz, $CDCl_3$) spectrum of **5ag**

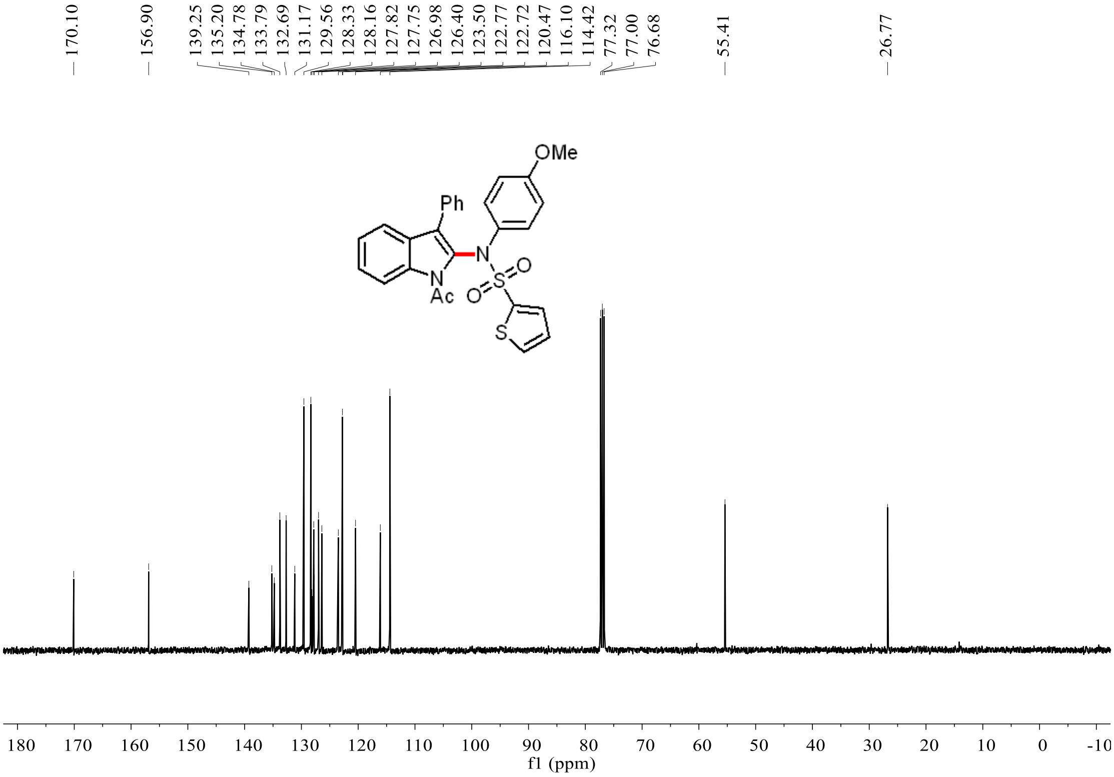

Supplementary Figure 110. ^{13}C NMR (101 MHz, $CDCl_3$) spectrum of **5ag**

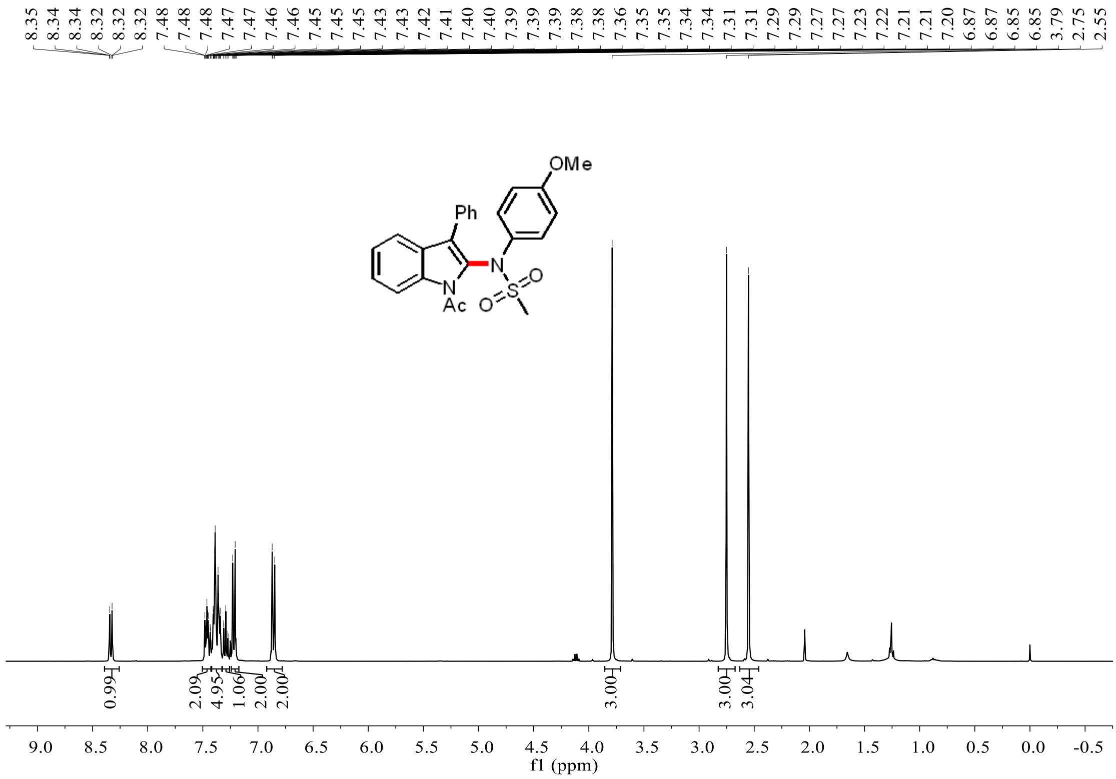

Supplementary Figure 111. ^{1}H NMR (400 MHz, $CDCl_3$) spectrum of **5ah**

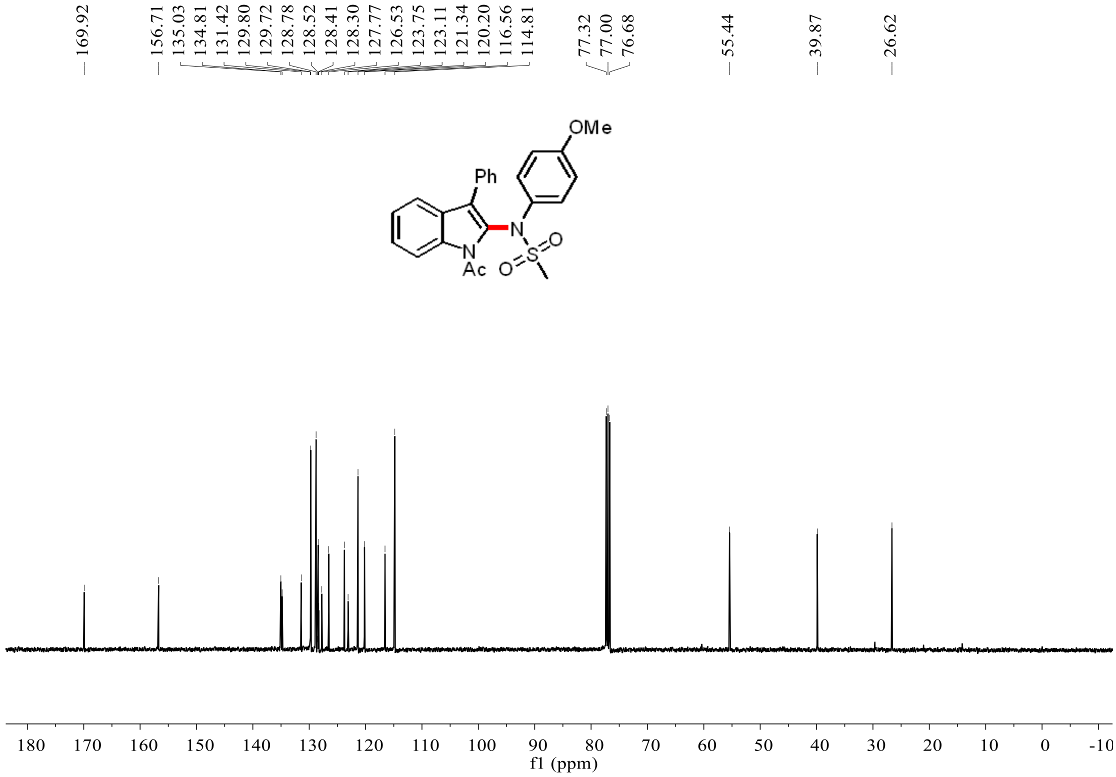

Supplementary Figure 112. ^{13}C NMR (101 MHz, $CDCl_3$) spectrum of **5ah**

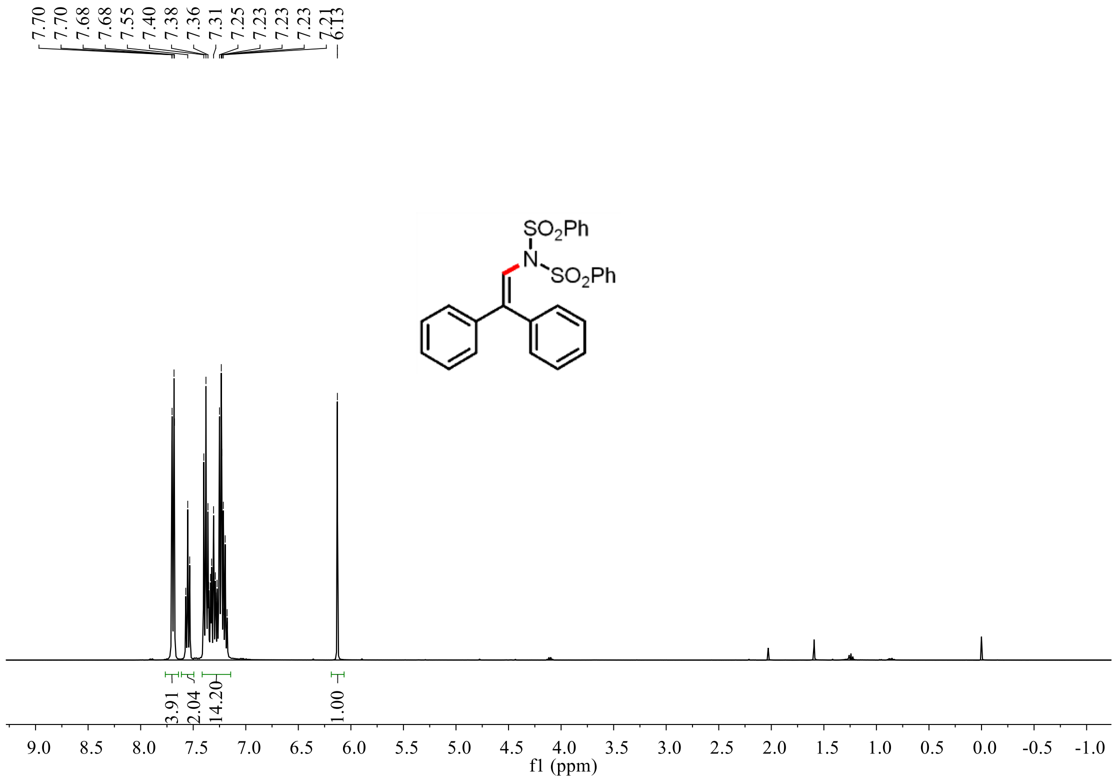

Supplementary Figure 113. ^{1}H NMR (400 MHz, $CDCl_3$) spectrum of **7aa**

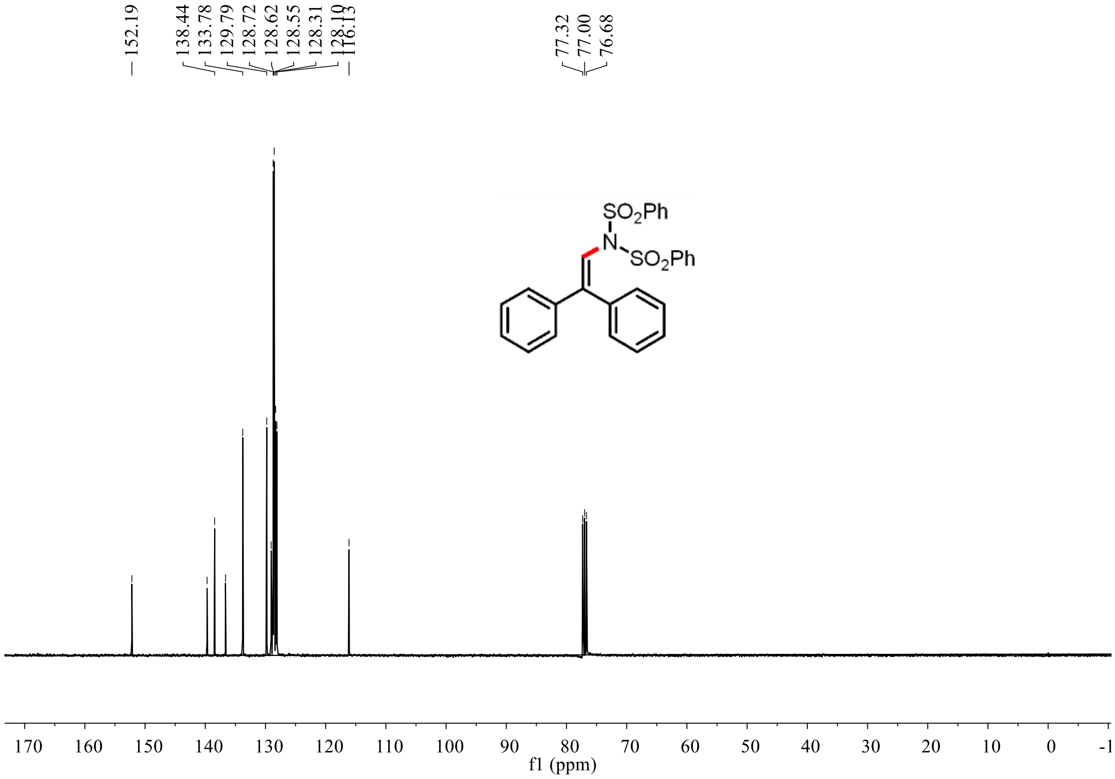

Supplementary Figure 114. ^{13}C NMR (101 MHz, $CDCl_3$) spectrum of **7aa**

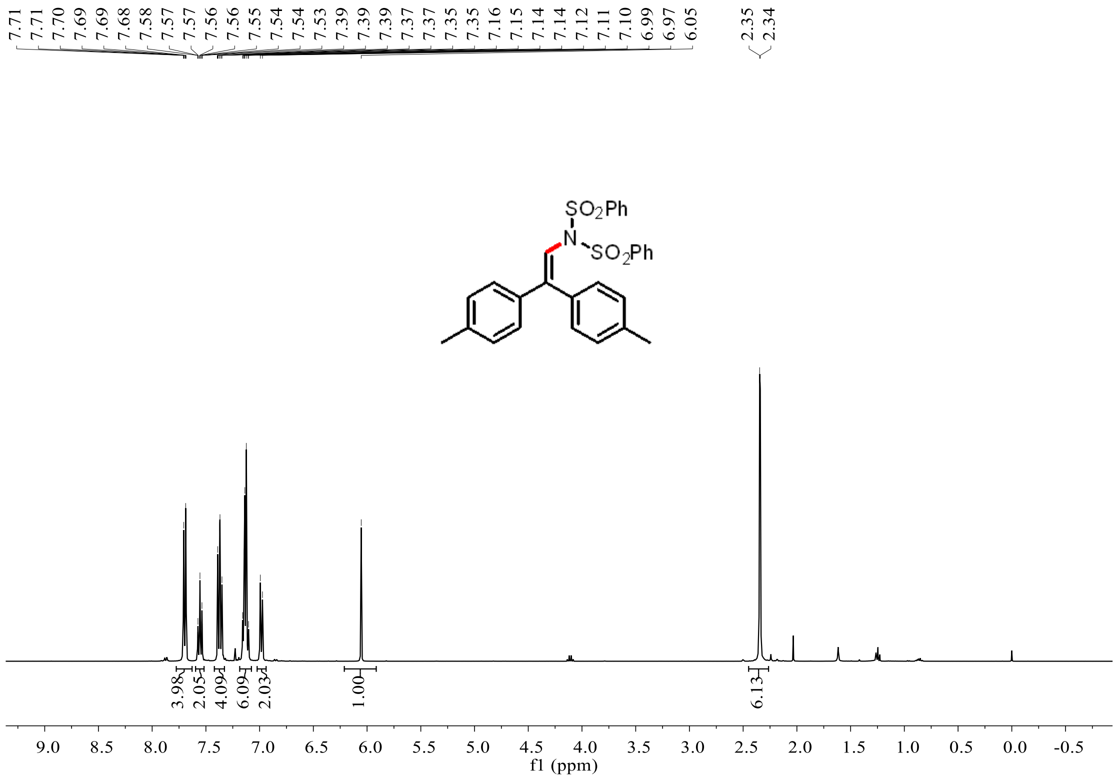

Supplementary Figure 115. ^{1}H NMR (400 MHz, $CDCl_3$) spectrum of **7ab**

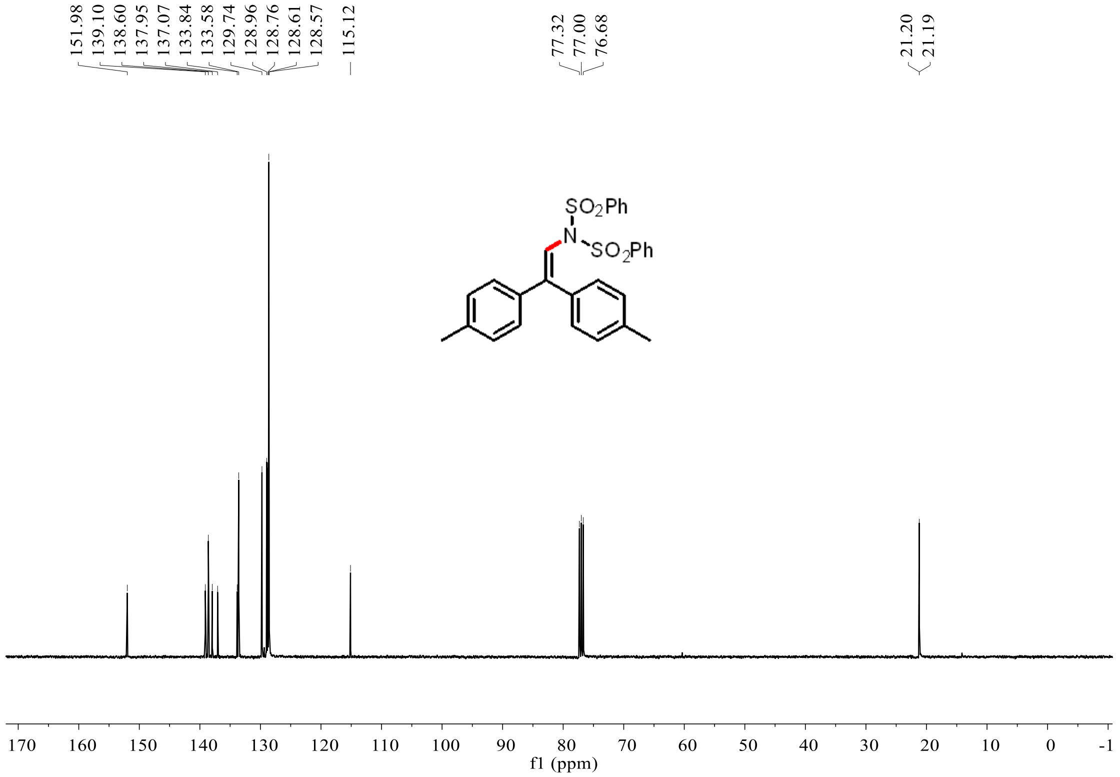

Supplementary Figure 116. ^{13}C NMR (101 MHz, $CDCl_3$) spectrum of **7ab**

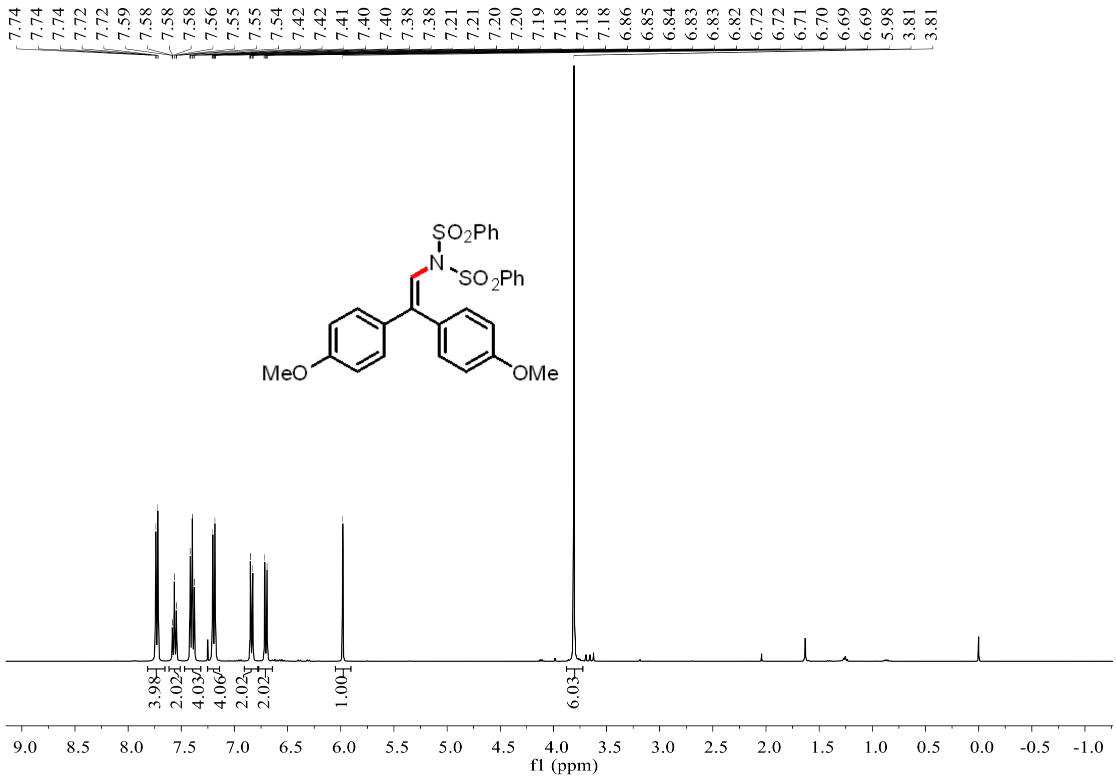

Supplementary Figure 117. ^{1}H NMR (400 MHz, $CDCl_3$) spectrum of **7ac**

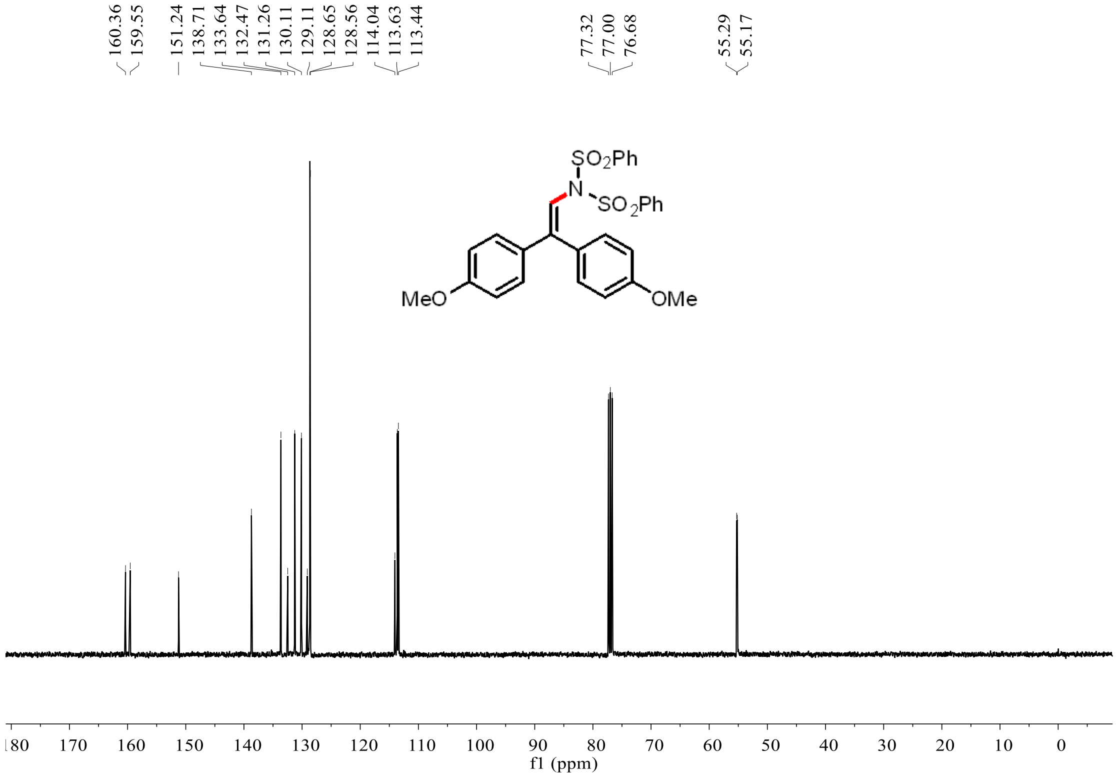

Supplementary Figure 118. ^{13}C NMR (101 MHz, $CDCl_3$) spectrum of **7ac**

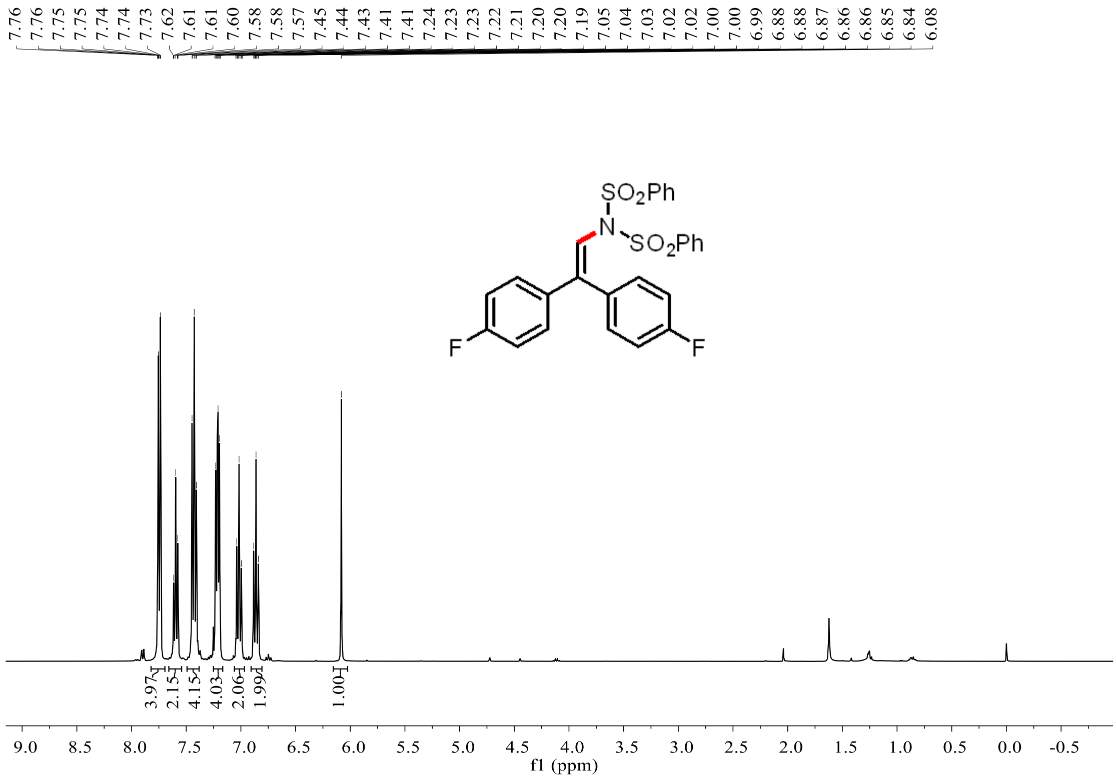

Supplementary Figure 119. ^{1}H NMR (400 MHz, $CDCl_3$) spectrum of **7ad**

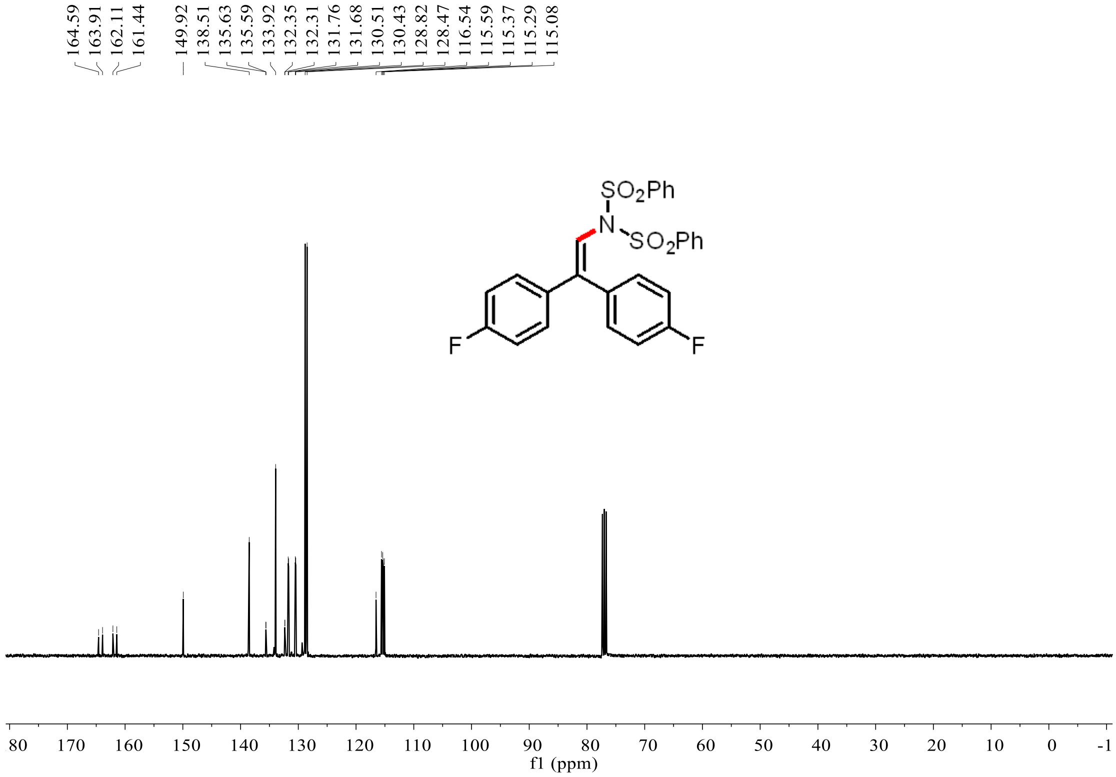

Supplementary Figure 120. ^{13}C NMR (101 MHz, $CDCl_3$) spectrum of **7ad**

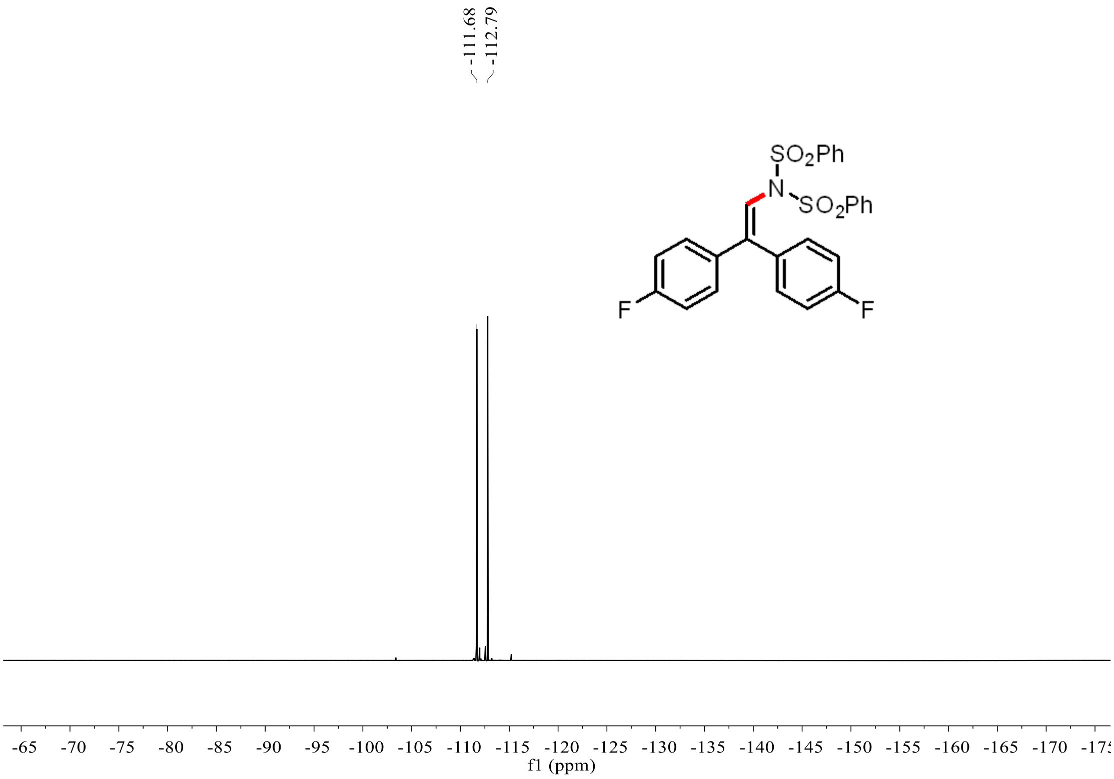

Supplementary Figure 121. ^{1}F NMR (377 MHz, $CDCl_3$) spectrum of **7ad**

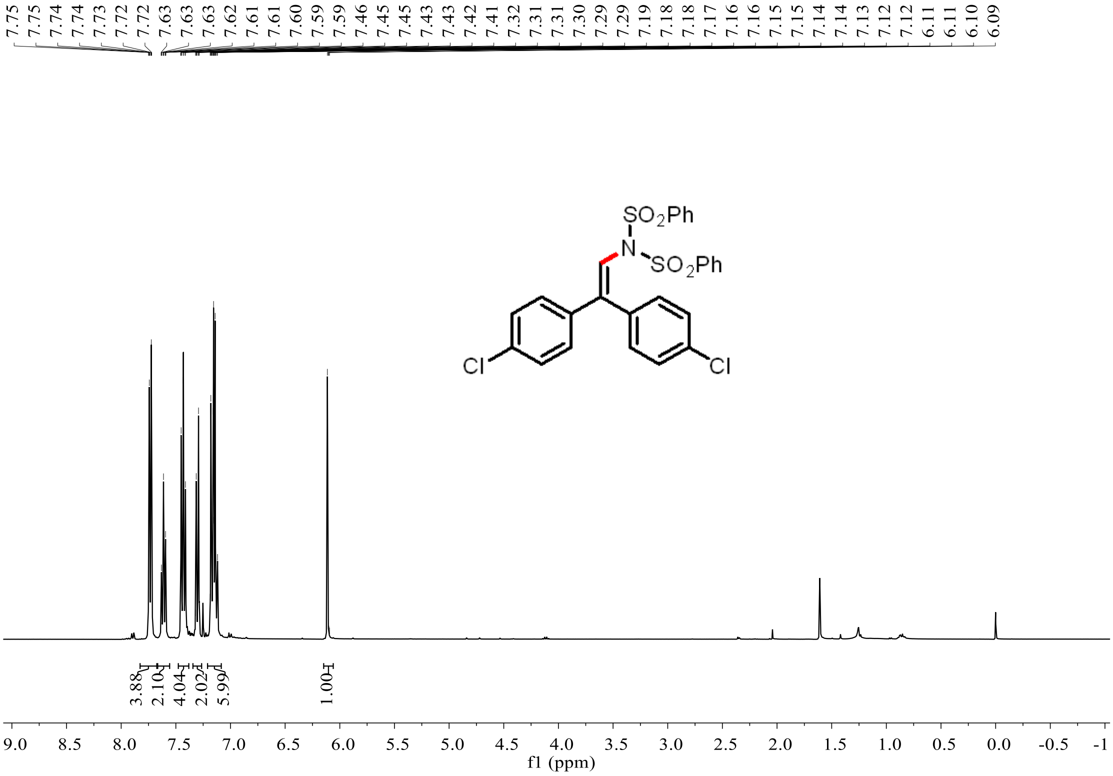

Supplementary Figure 122. ^{1}H NMR (400 MHz, $CDCl_3$) spectrum of **7ae**

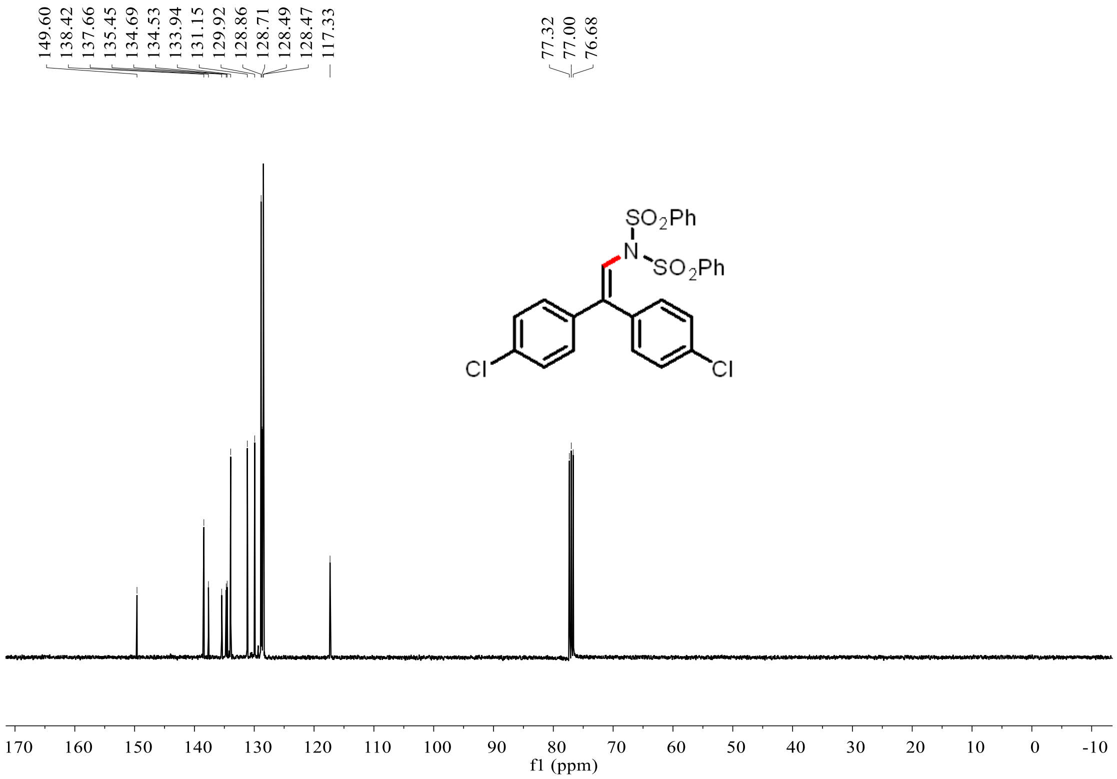

Supplementary Figure 123. ^{13}C NMR (101 MHz, $CDCl_3$) spectrum of **7ae**

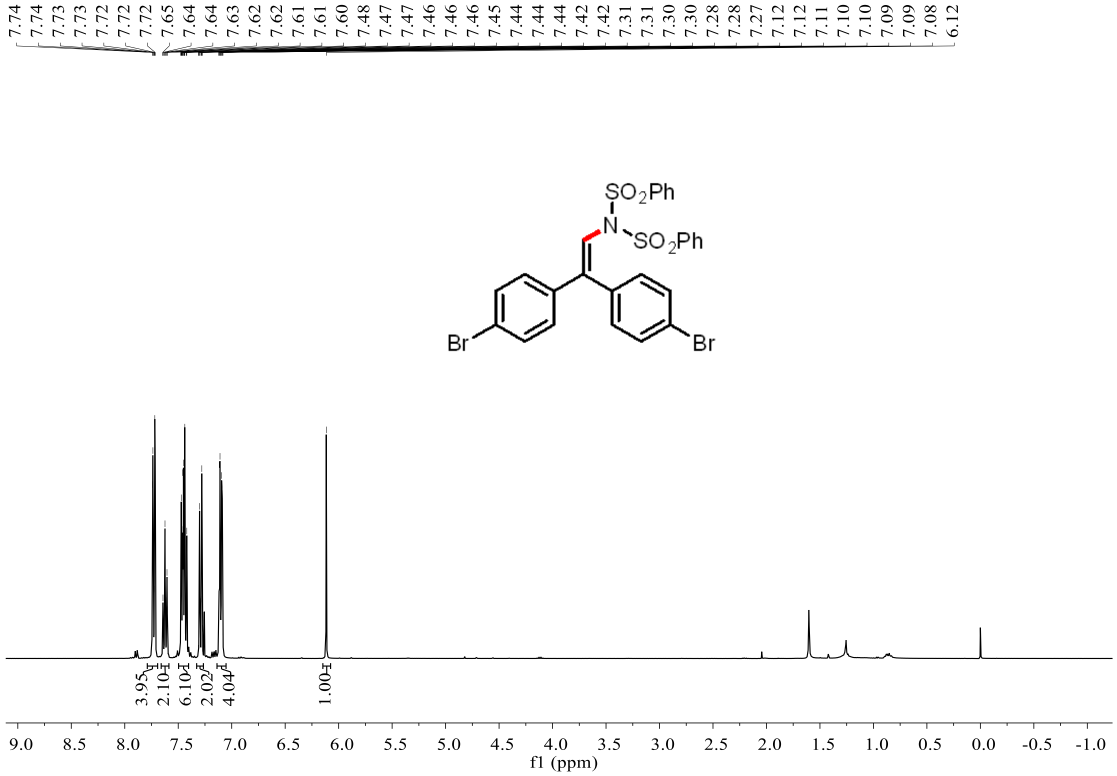

Supplementary Figure 124. ^{1}H NMR (400 MHz, $CDCl_3$) spectrum of **7af**

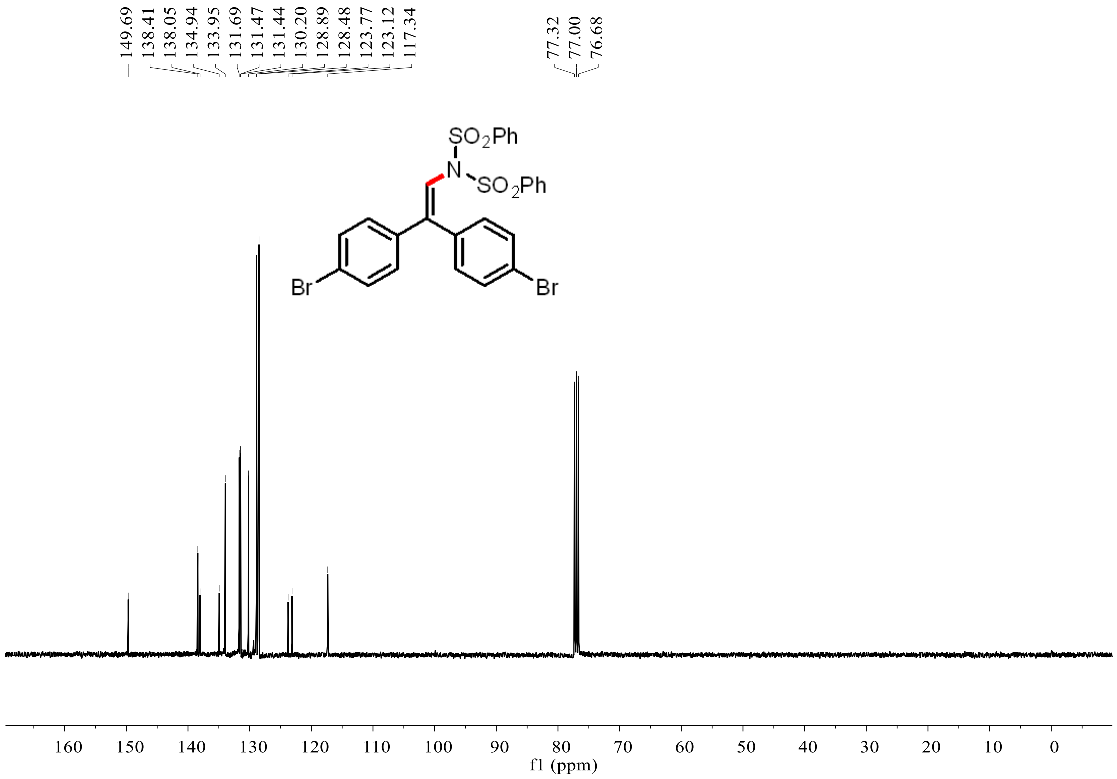

Supplementary Figure 125. ^{13}C NMR (101 MHz, $CDCl_3$) spectrum of **7af**

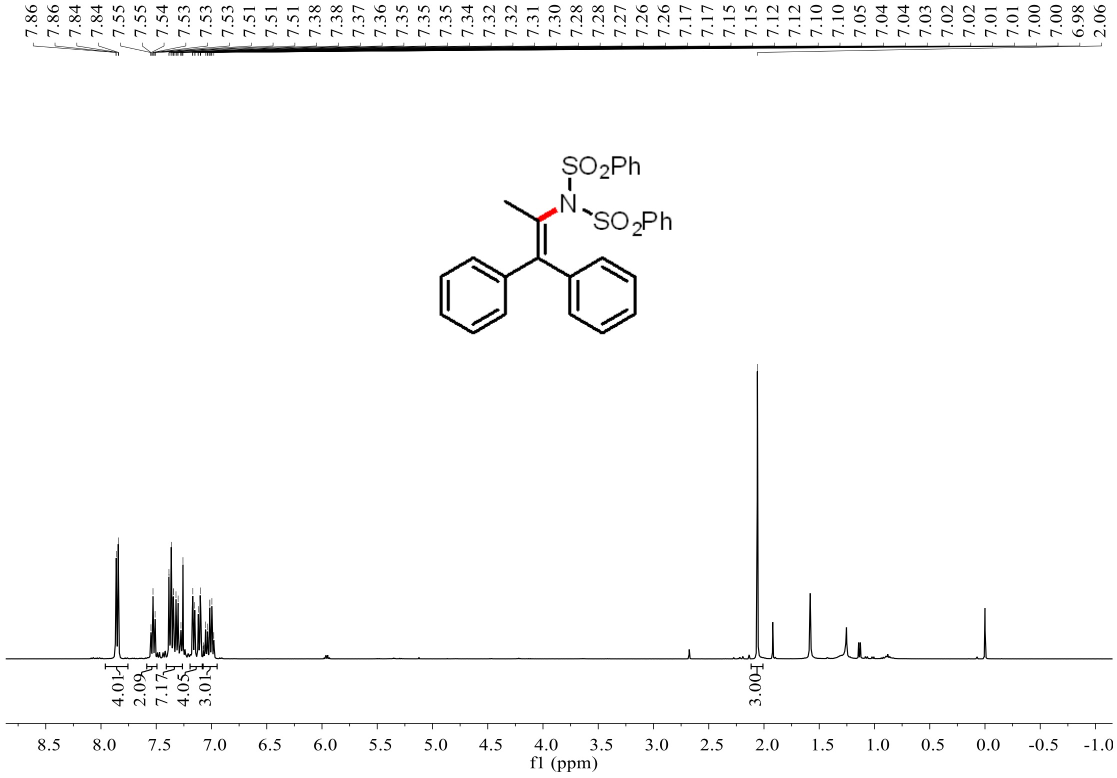

Supplementary Figure 126. ^{1}H NMR (400 MHz, $CDCl_3$) spectrum of **7ag**

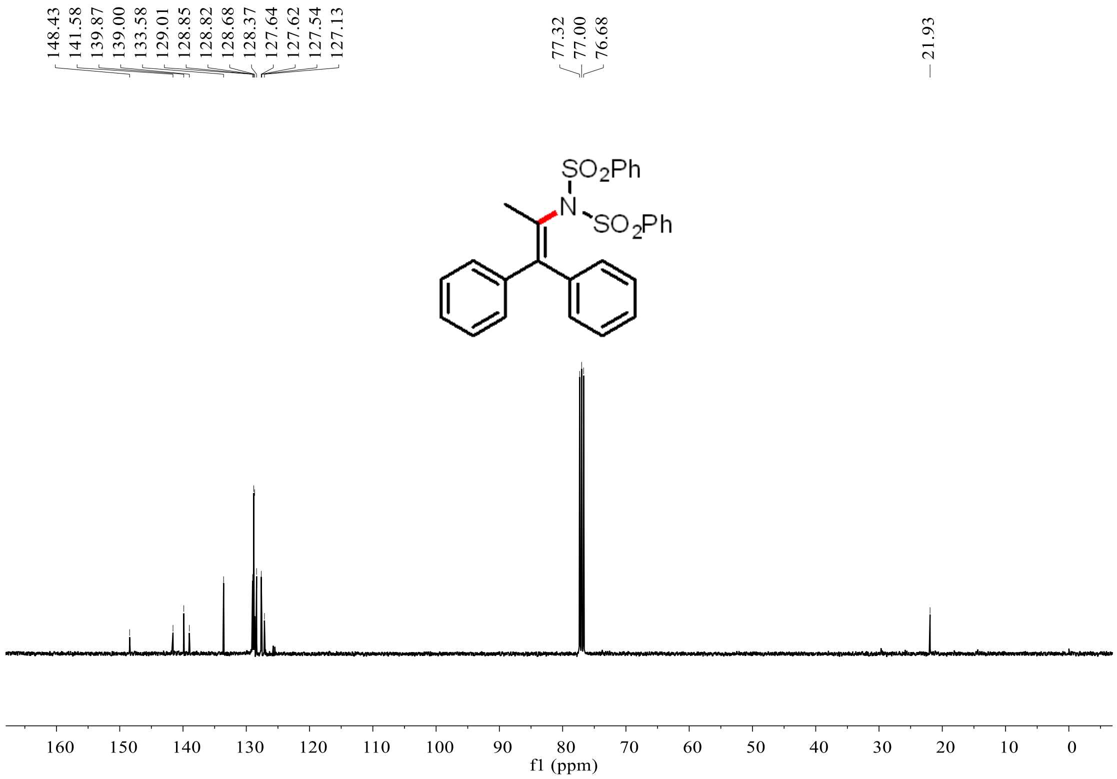

Supplementary Figure 127. ^{13}C NMR (101 MHz, $CDCl_3$) spectrum of **7ag**

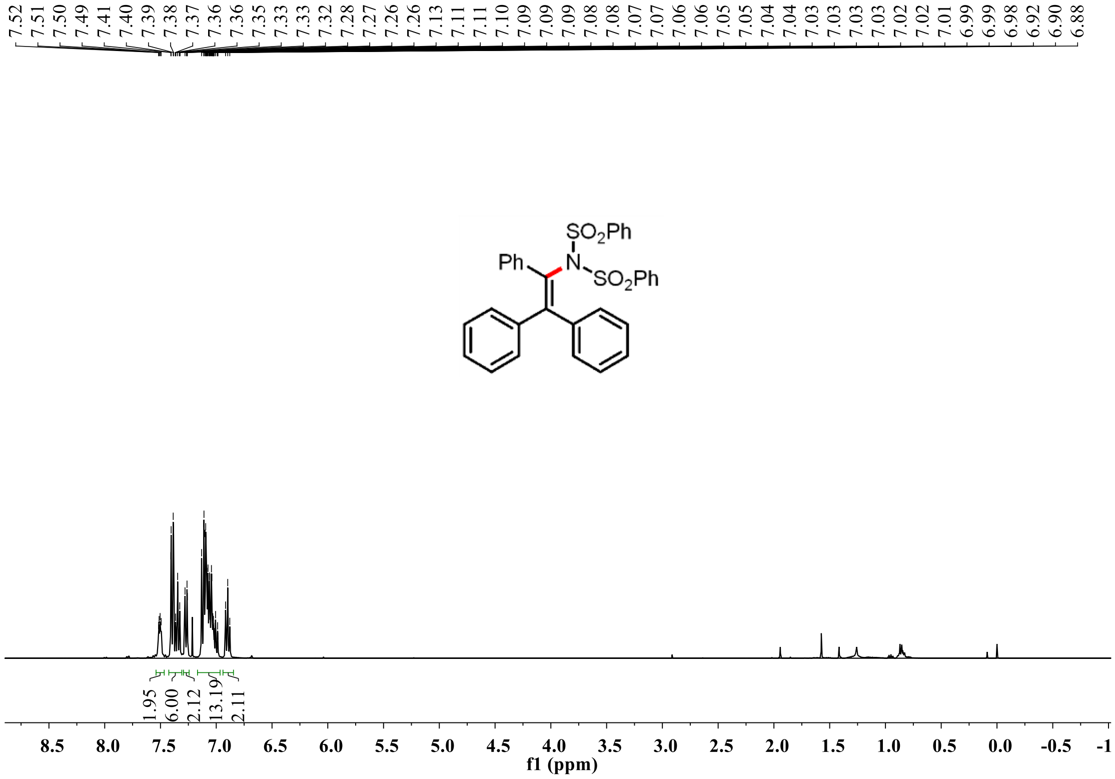

Supplementary Figure 128. ^{1}H NMR (400 MHz, $CDCl_3$) spectrum of **7ah**

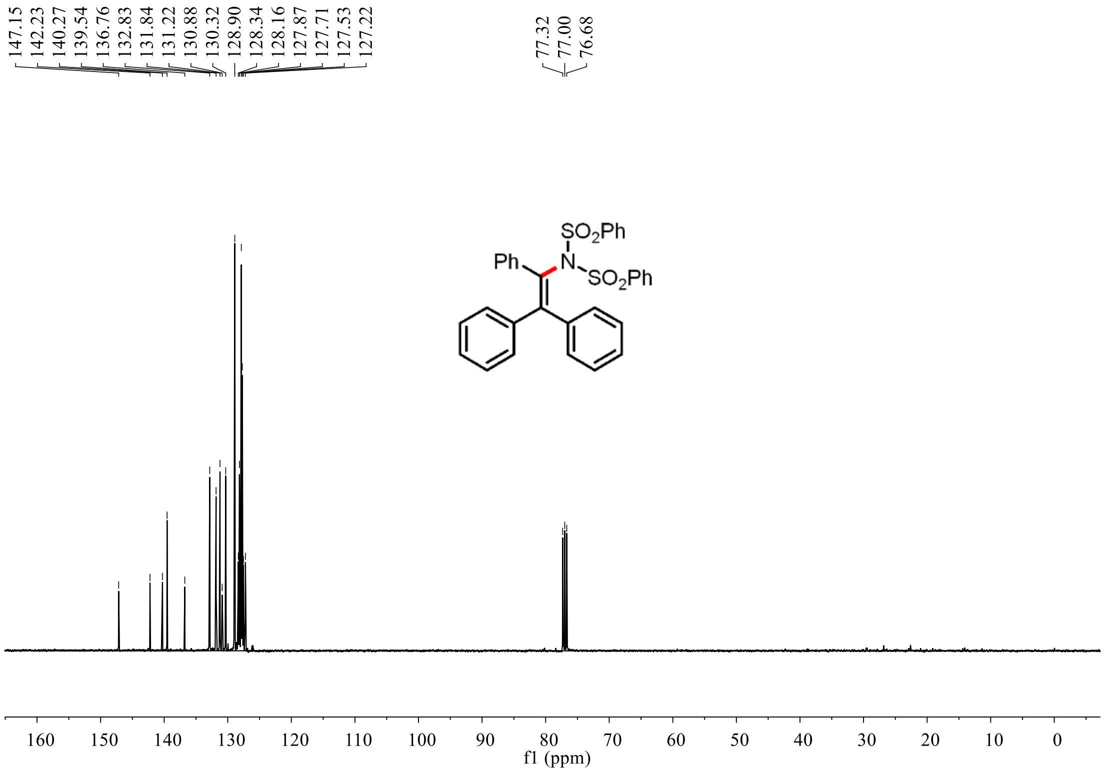

Supplementary Figure 129. ^{13}C NMR (101 MHz, $CDCl_3$) spectrum of **7ah**

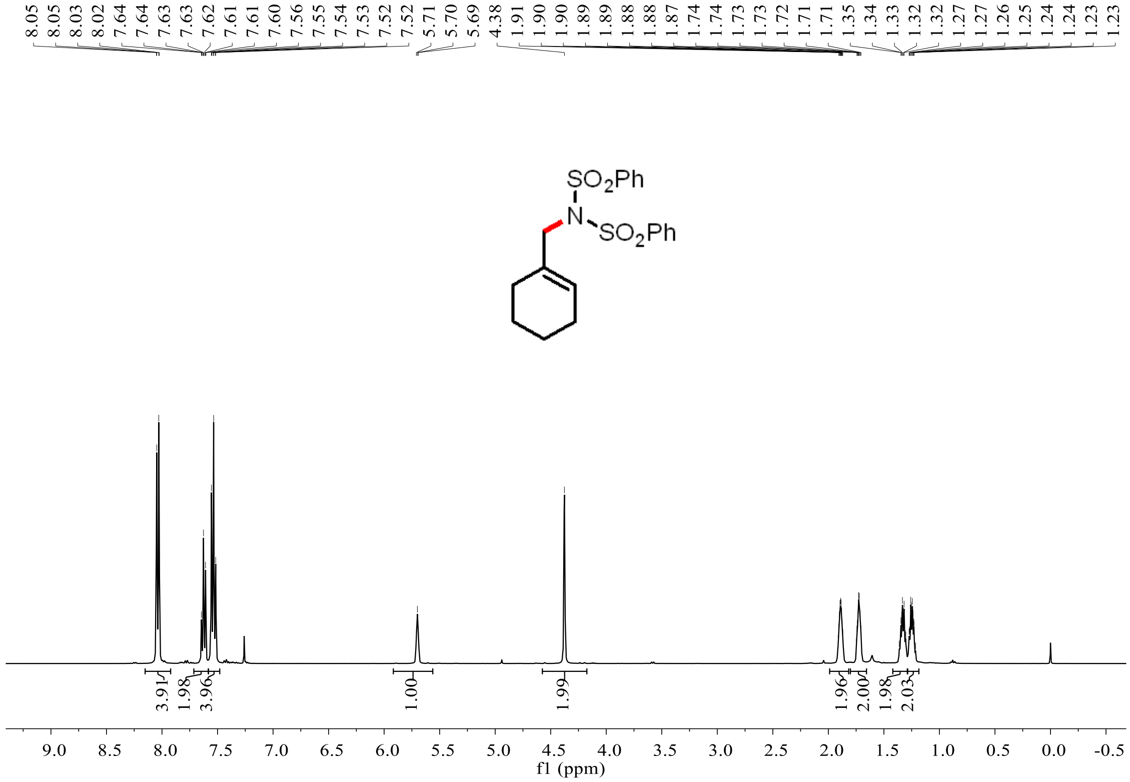

Supplementary Figure 130. ^{1}H NMR (400 MHz, $CDCl_3$) spectrum of **7ai**

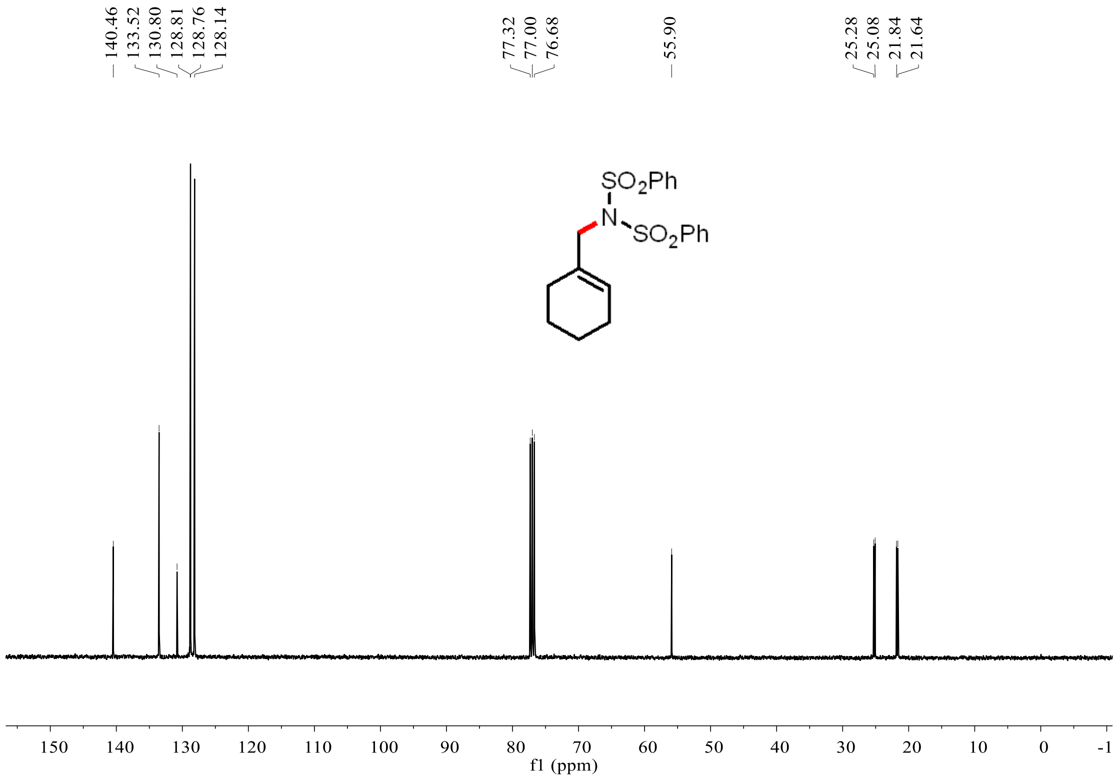

Supplementary Figure 131. ^{13}C NMR (101 MHz, $CDCl_3$) spectrum of **7ai**

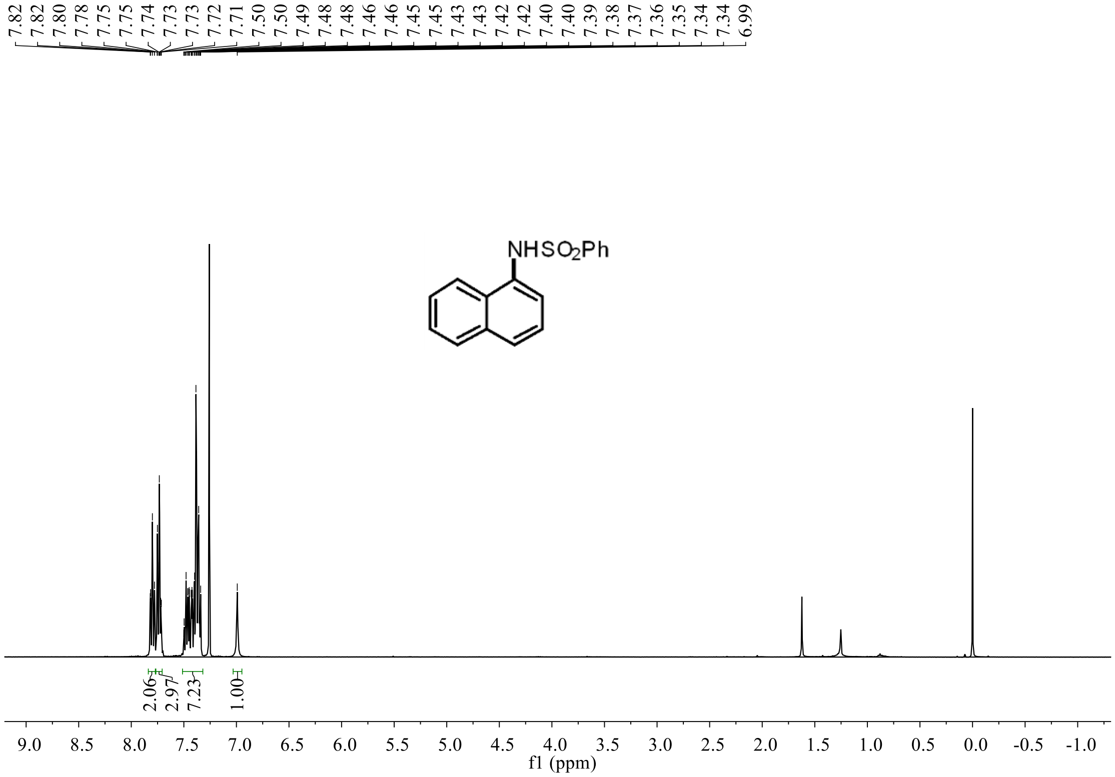

Supplementary Figure 132. ^{1}H NMR (400 MHz, $CDCl_3$) spectrum of **8aa**

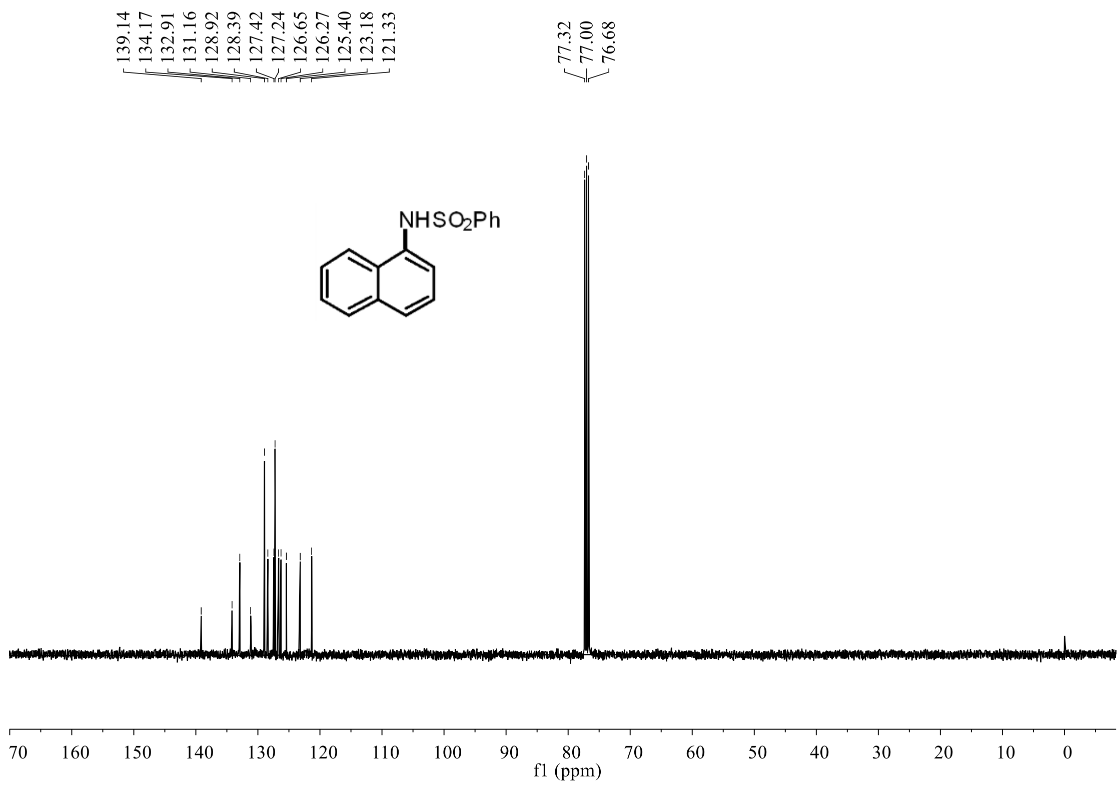

Supplementary Figure 133. ^{13}C NMR (101 MHz, $CDCl_3$) spectrum of **8aa**

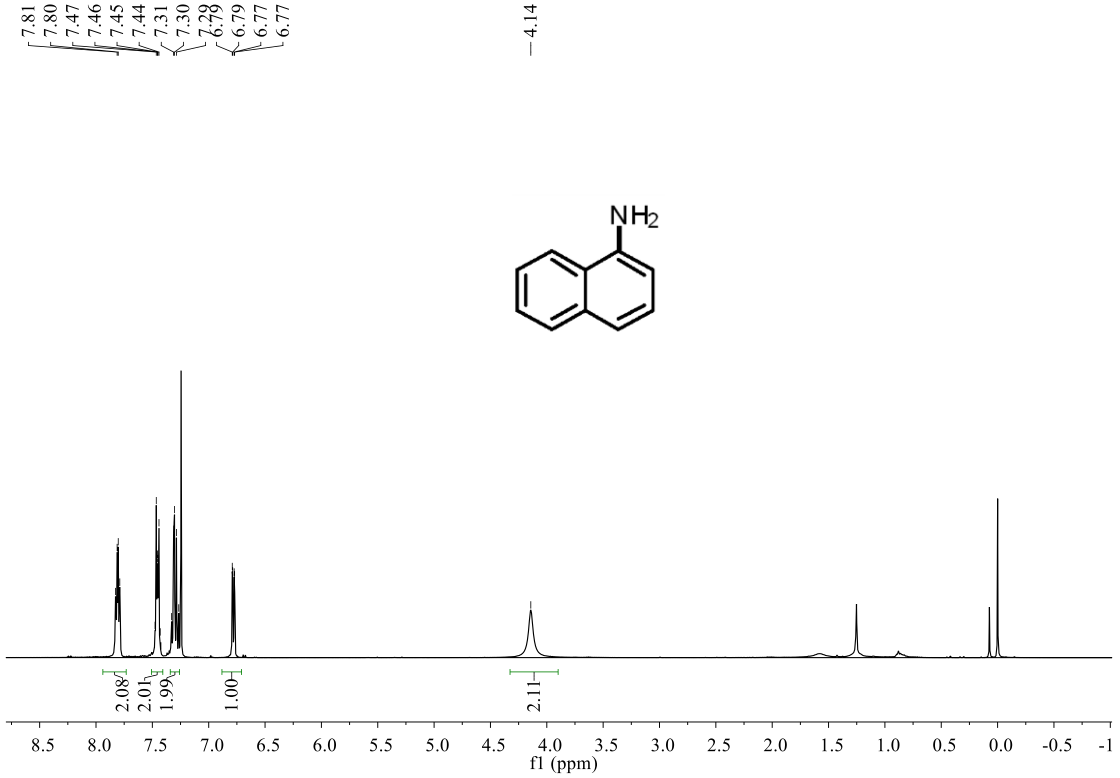

Supplementary Figure 134. ^{1}H NMR (400 MHz, $CDCl_3$) spectrum of **9aa**

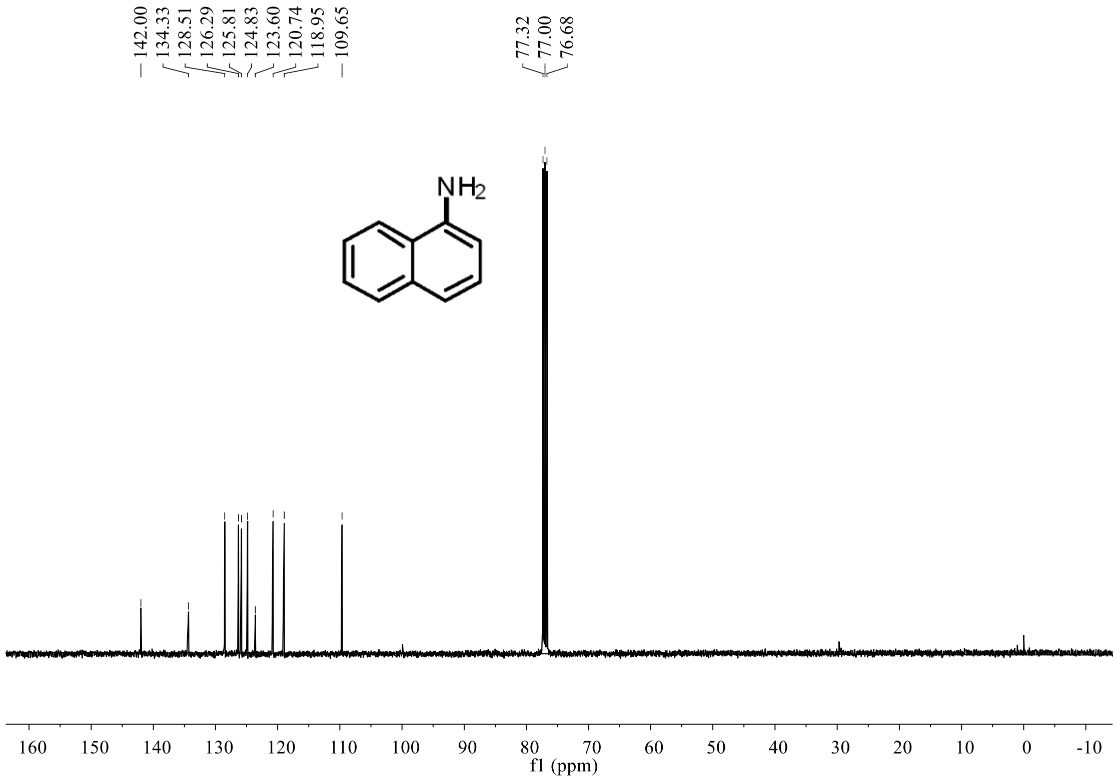

Supplementary Figure 135. ^{13}C NMR (101 MHz, $CDCl_3$) spectrum of **9aa**

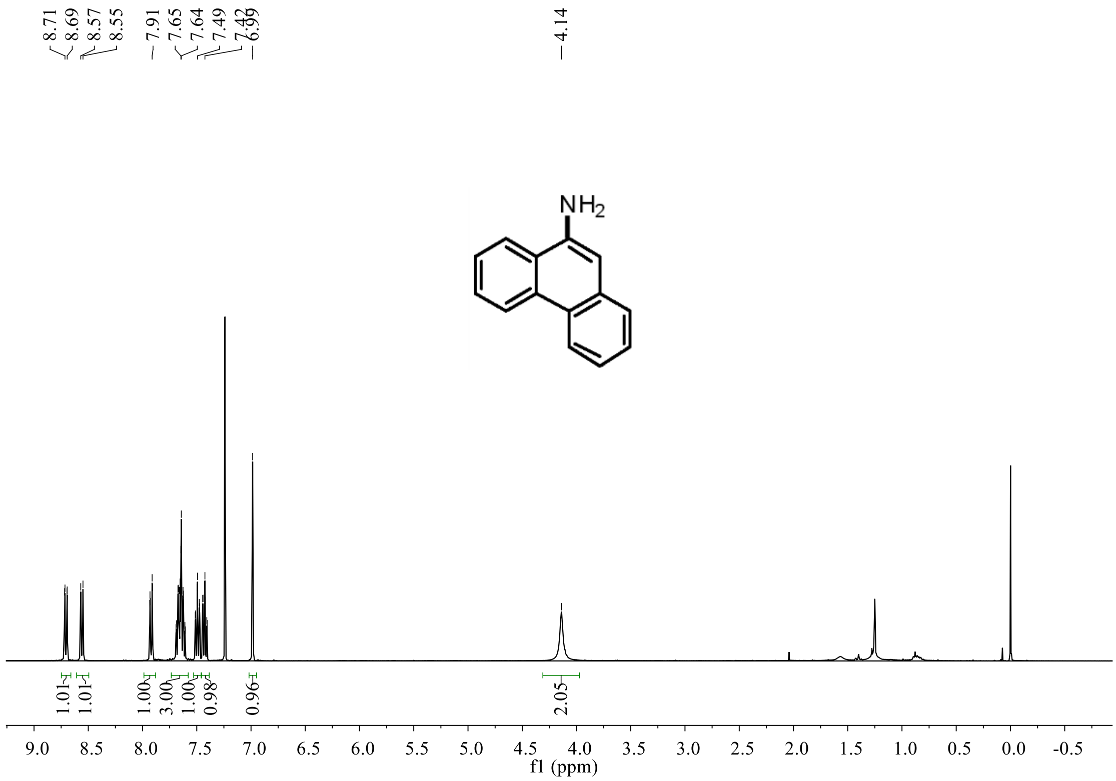

Supplementary Figure 136. ^{1}H NMR (400 MHz, $CDCl_3$) spectrum of **9ab**

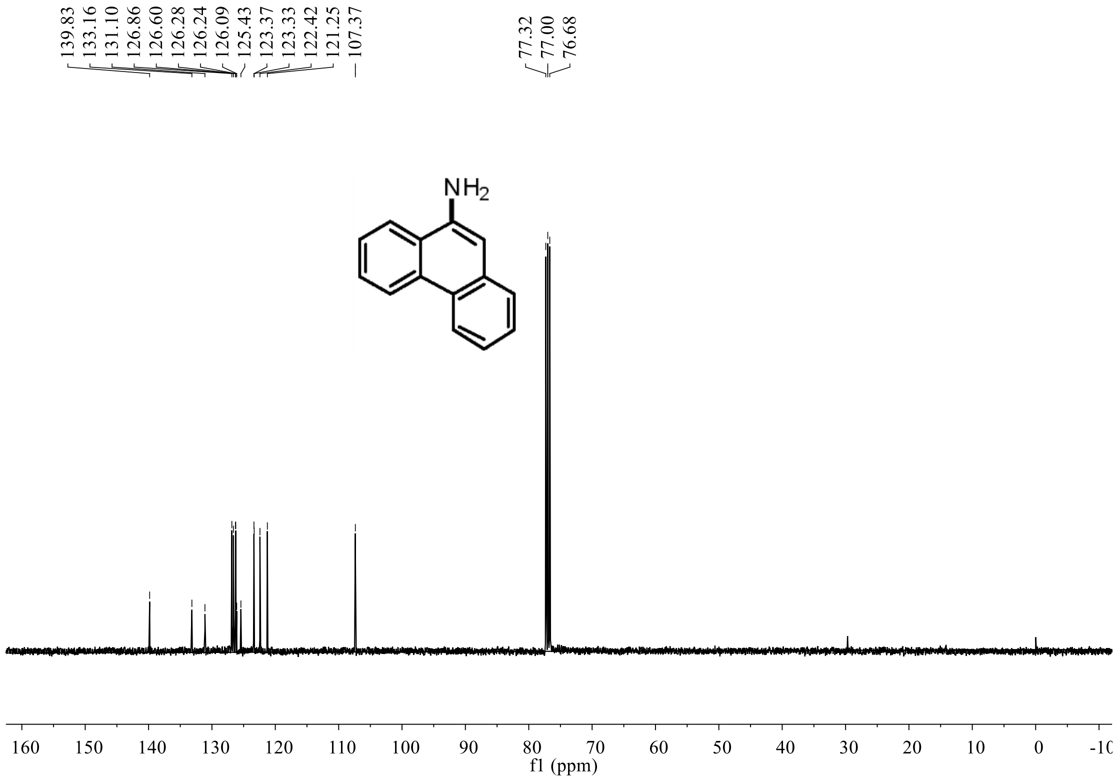

Supplementary Figure 137. ^{13}C NMR (101 MHz, $CDCl_3$) spectrum of **9ab**

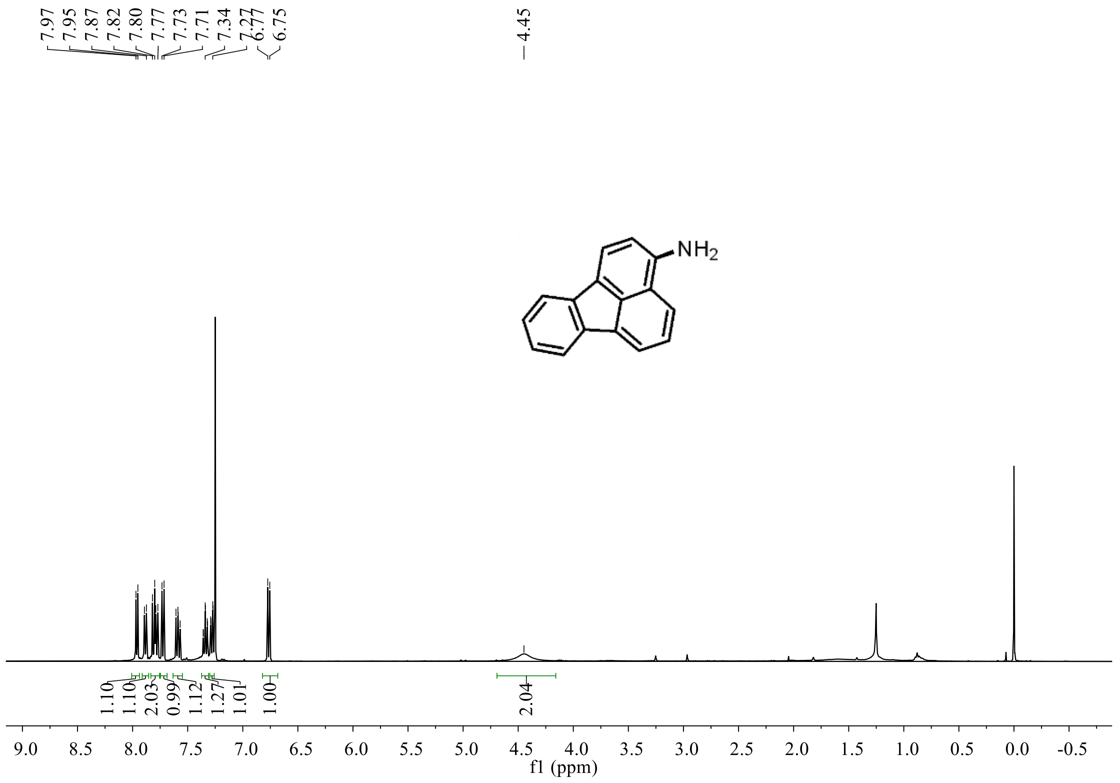

Supplementary Figure 138. ^{1}H NMR (400 MHz, $CDCl_3$) spectrum of **9ad**

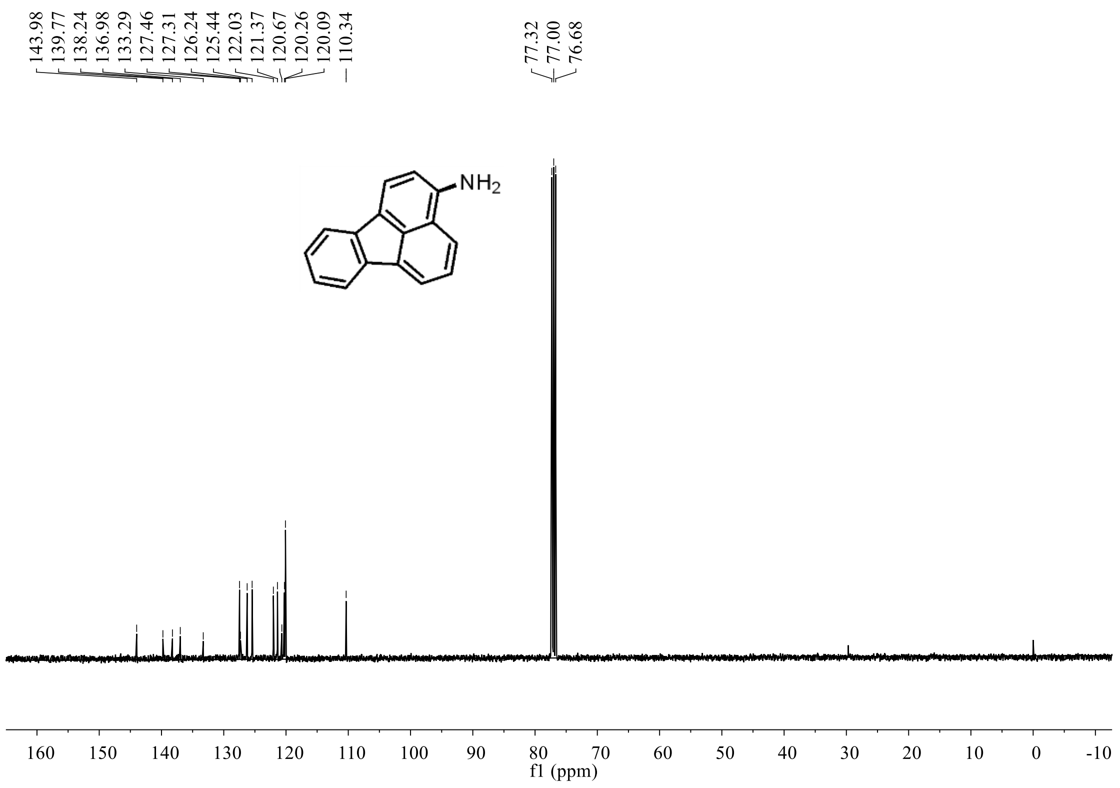

Supplementary Figure 139. ^{13}C NMR (101 MHz, $CDCl_3$) spectrum of **9ad**

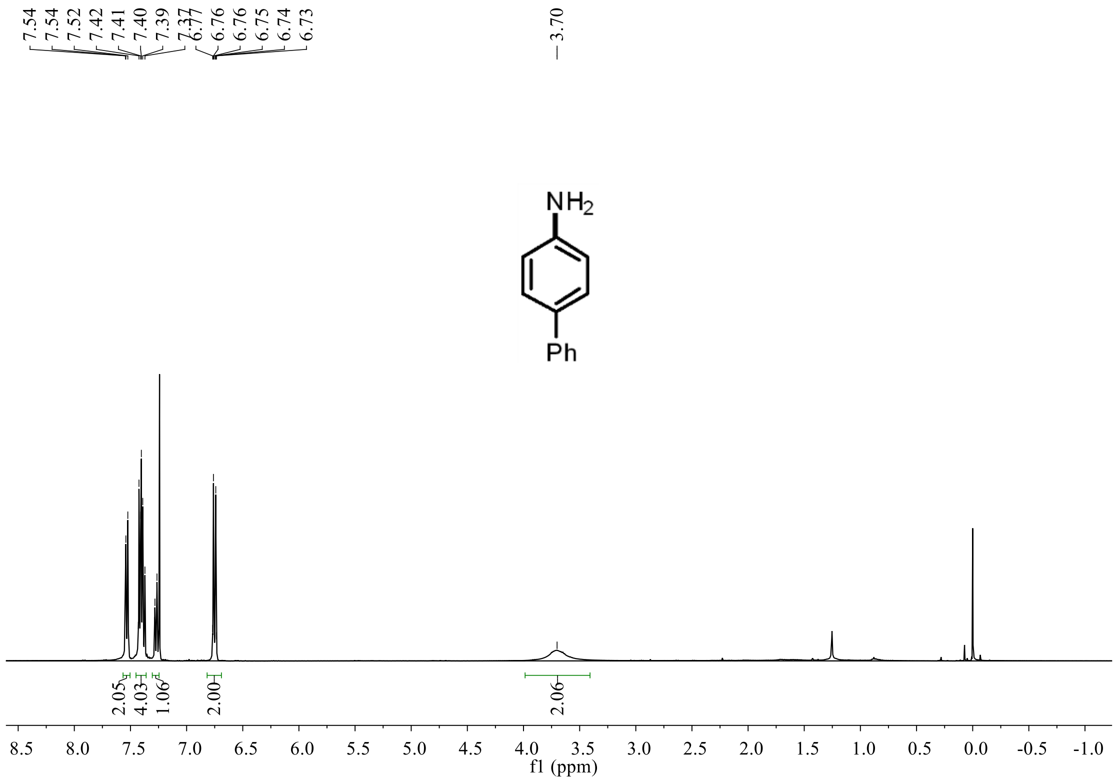

Supplementary Figure 140. ^{1}H NMR (400 MHz, $CDCl_3$) spectrum of **9af**

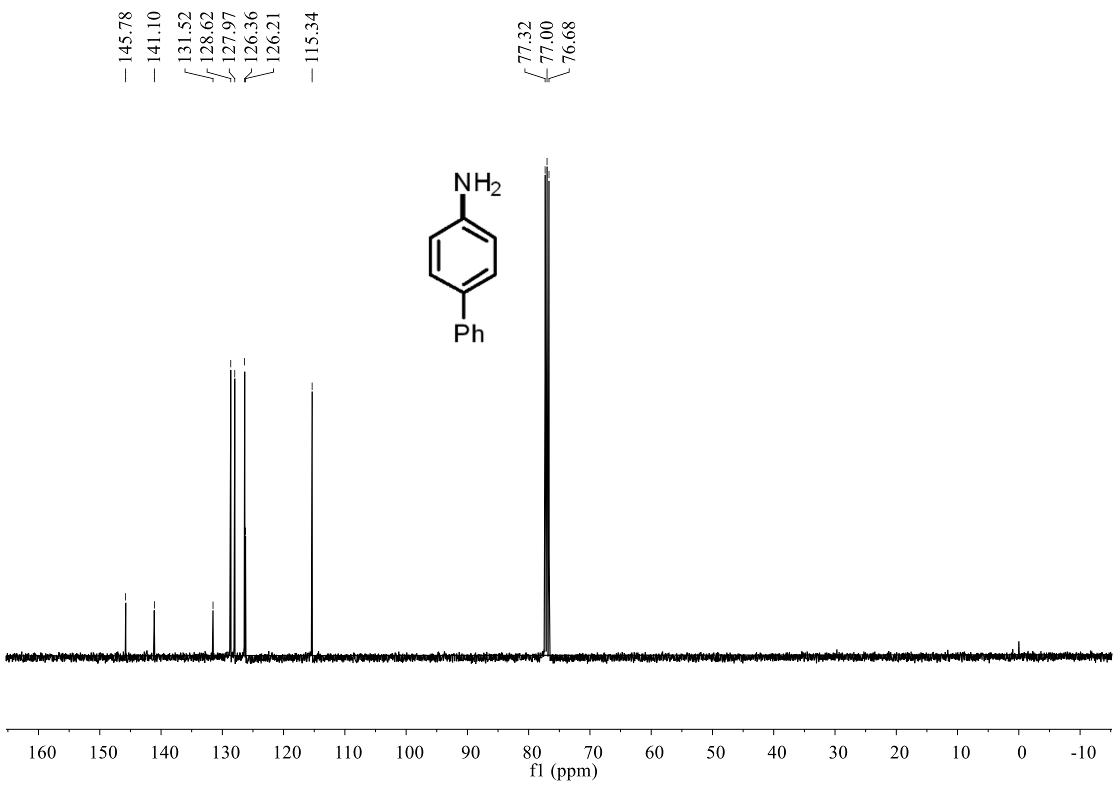

Supplementary Figure 141. ^{13}C NMR (101 MHz, $CDCl_3$) spectrum of **9af**

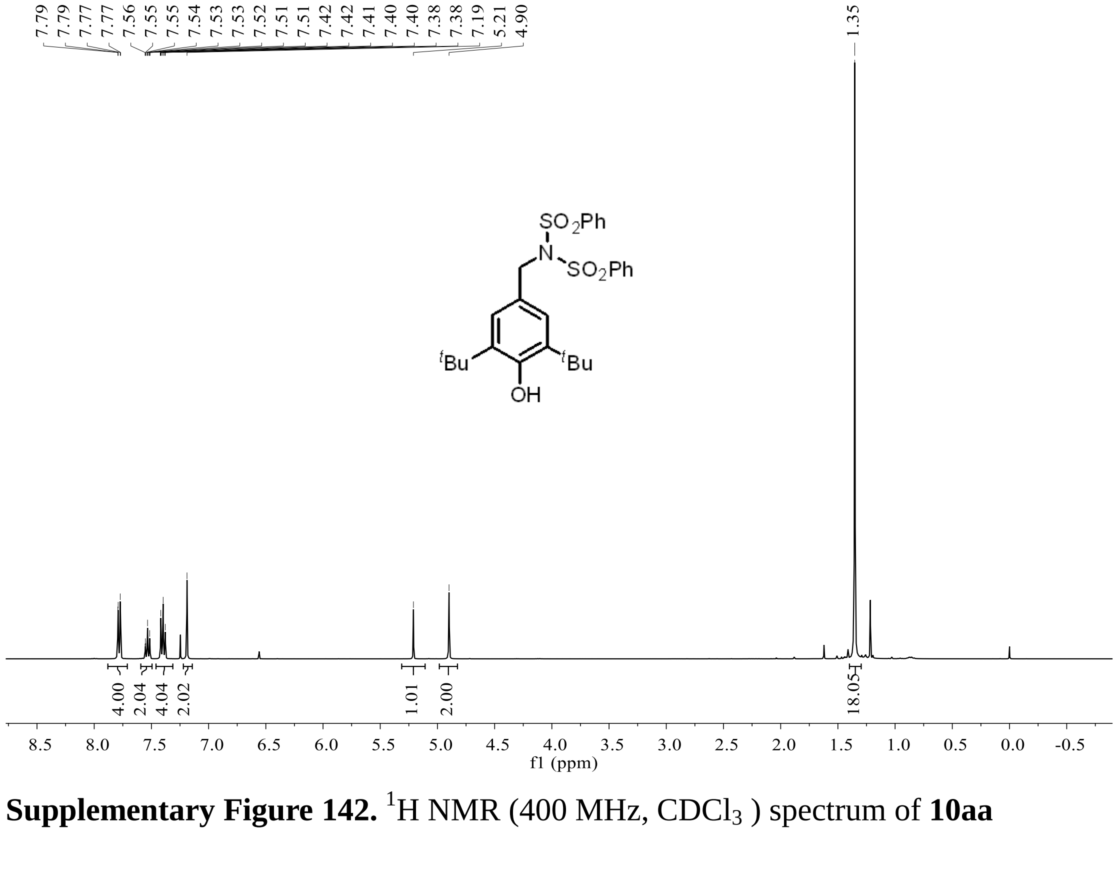

Supplementary Figure 142. ^{1}H NMR (400 MHz, $CDCl_3$) spectrum of **10aa**

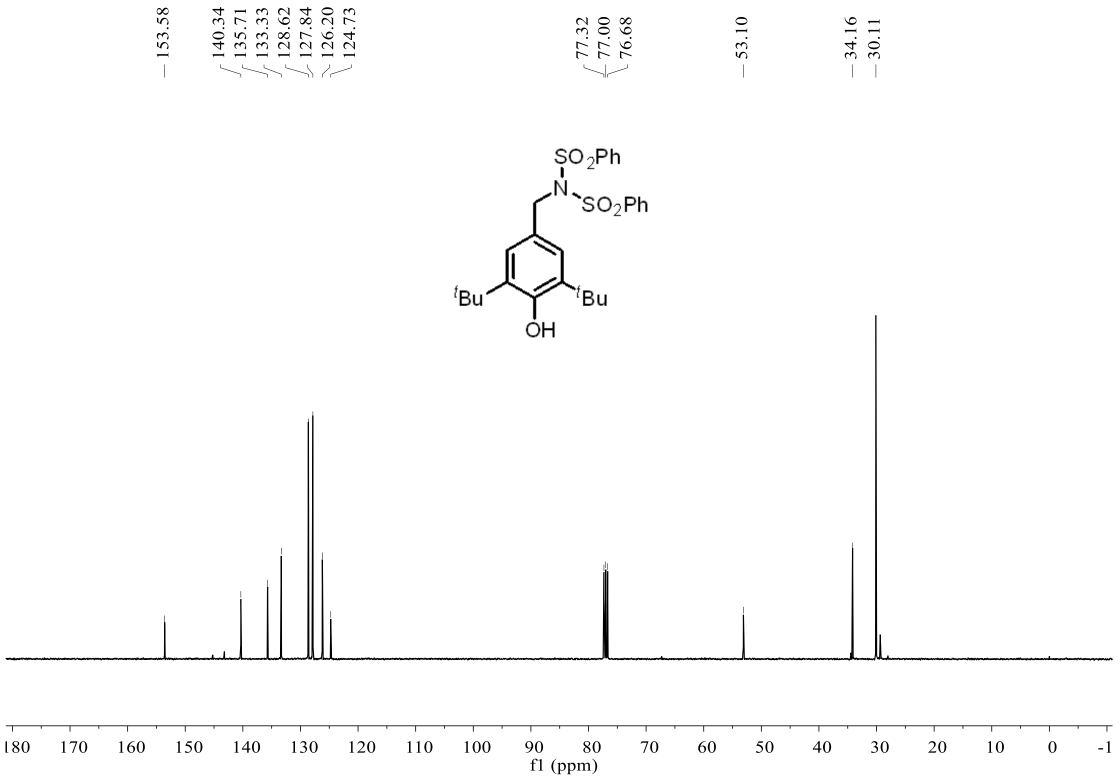

Supplementary Figure 143. ^{13}C NMR (101 MHz, $CDCl_3$) spectrum of **10aa**

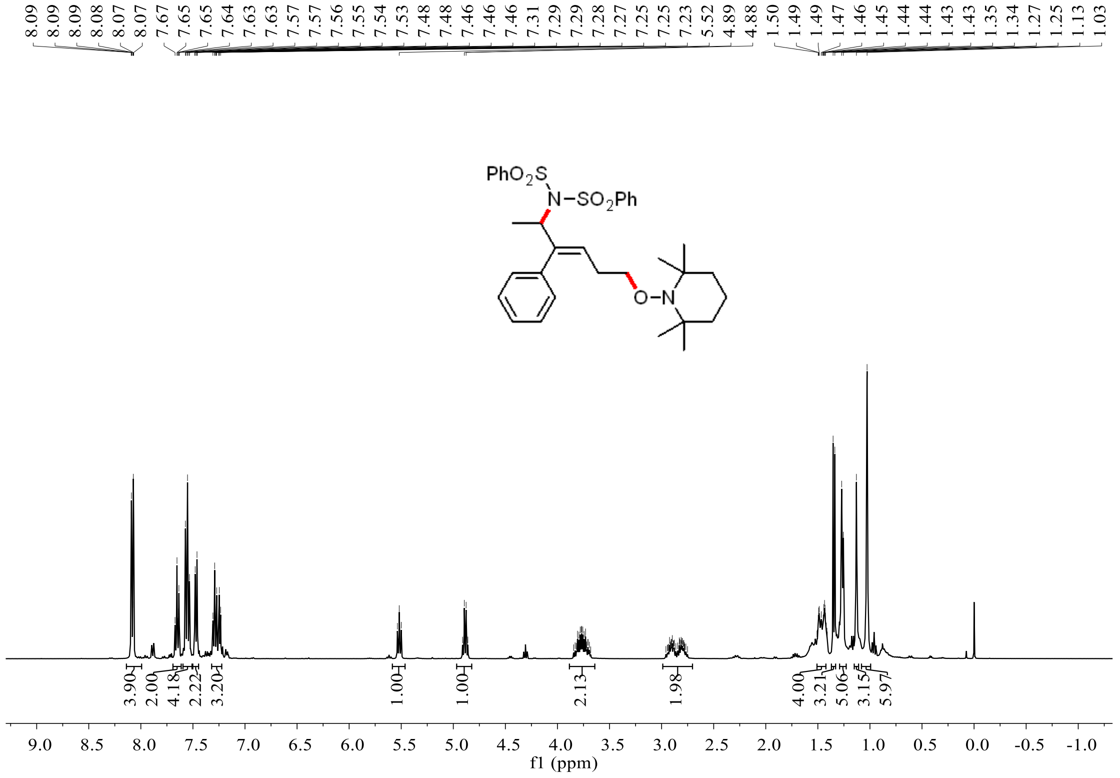

Supplementary Figure 144. ^{1}H NMR (400 MHz, $CDCl_3$) spectrum of **13aa**

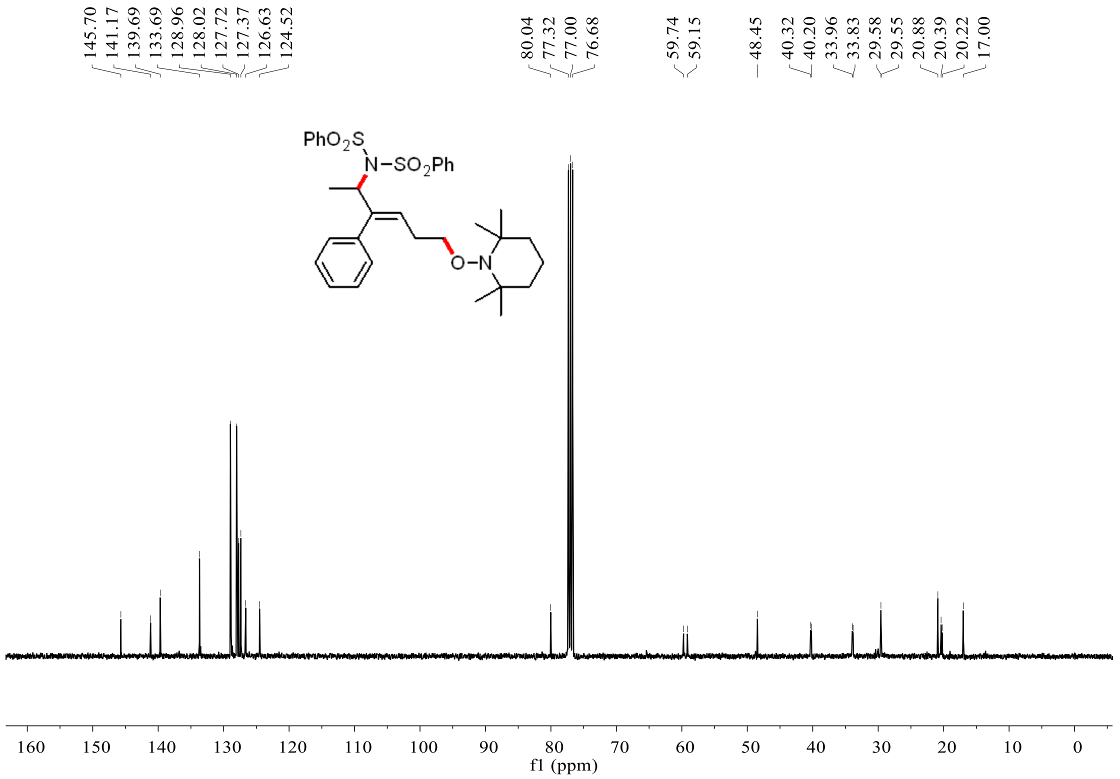

Supplementary Figure 145. ^{13}C NMR (101 MHz, $CDCl_3$) spectrum of **13aa**

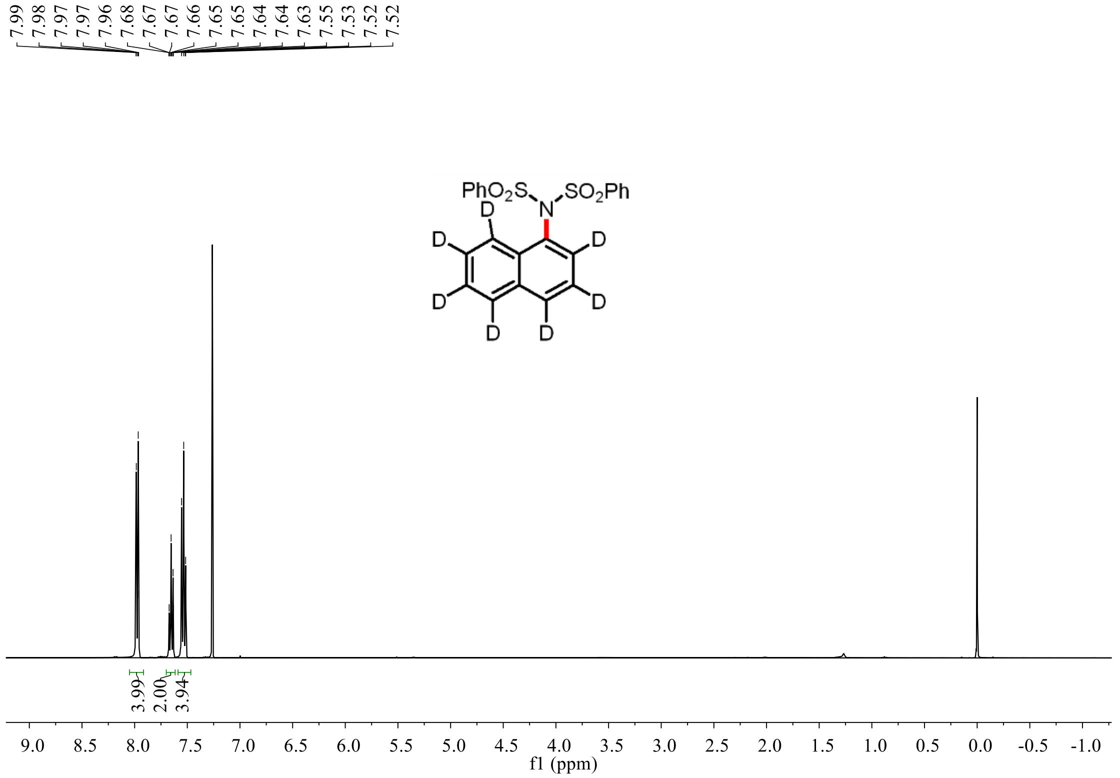

Supplementary Figure 146. ^{1}H NMR (400 MHz, $CDCl_3$) spectrum of **[D8]-3aa**

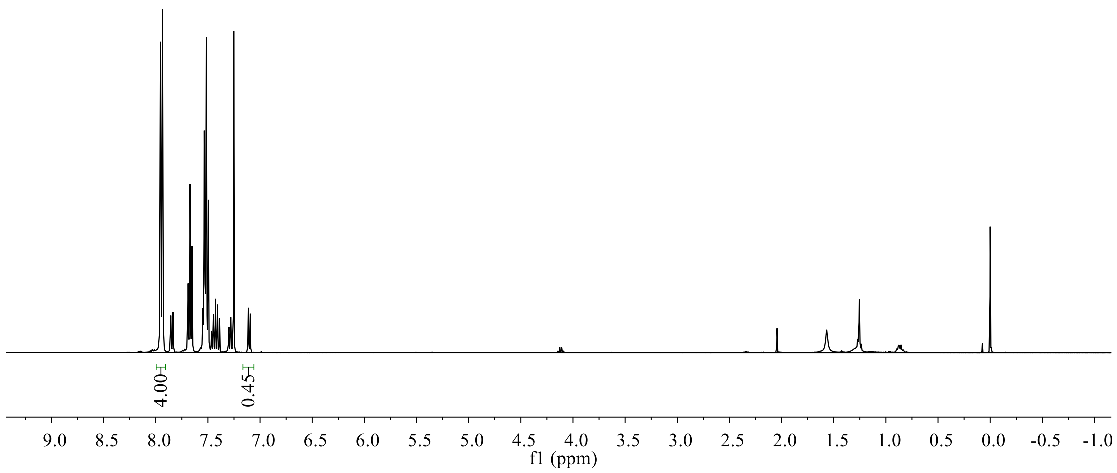

Supplementary Figure 147. ^{1}H NMR (400 MHz, $CDCl_3$) spectrum of **3aa: [D8]-3aa** = 0.82: 1

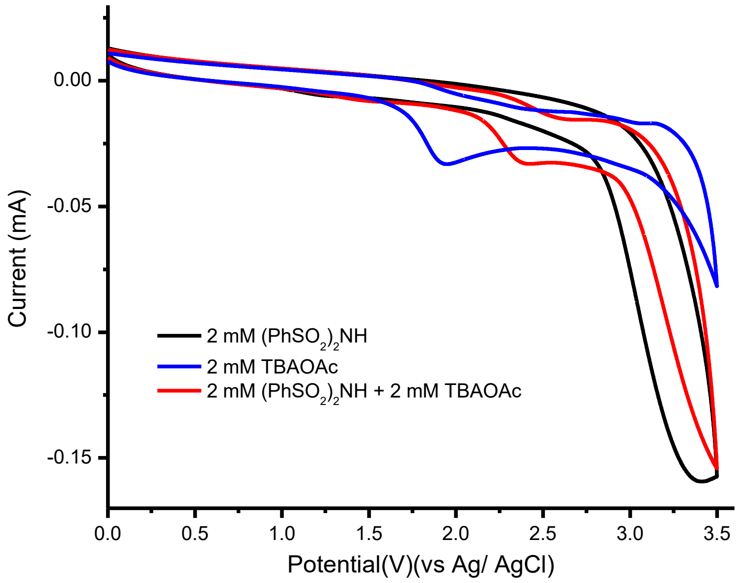

Supplementary Figure 148. Cyclic voltammetry studies. Cyclic voltammetry of **2aa** (0.002M), $^nBu_4NOAc$(0.002M), and a mixture of **2aa** (0.002M) with 1 equivalent $^nBu_4NOAc$ (0.002M) in acetonitrile containing 0.1 M $^nBu_4NBF_4$ under nitrogen at a glass carbon electrode at a scan rate of v=0.05 V/s.

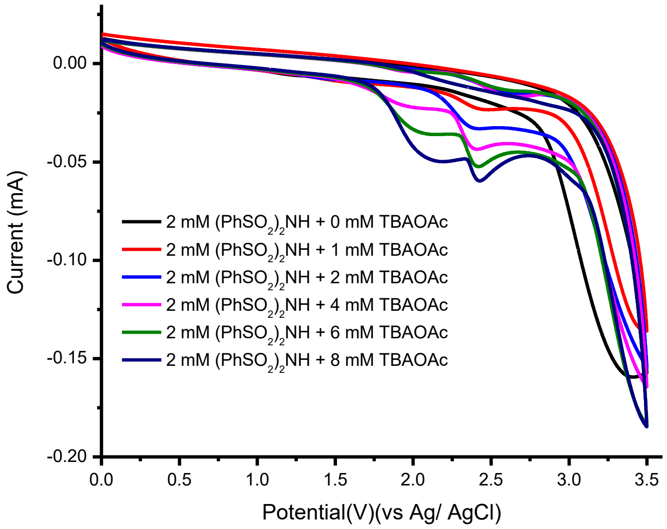

Supplementary Figure 149. Cyclic voltammetry studies. Cyclic voltammetry of **2aa** (0.002M) in acetonitrile containing $^{n}Bu_4NBF_4$ (0.1 M) and varying concentrations of $^{n}Bu_4NOAc$ under nitrogen at a glass carbon electrode at a scan rate of v=0.05 V/s.

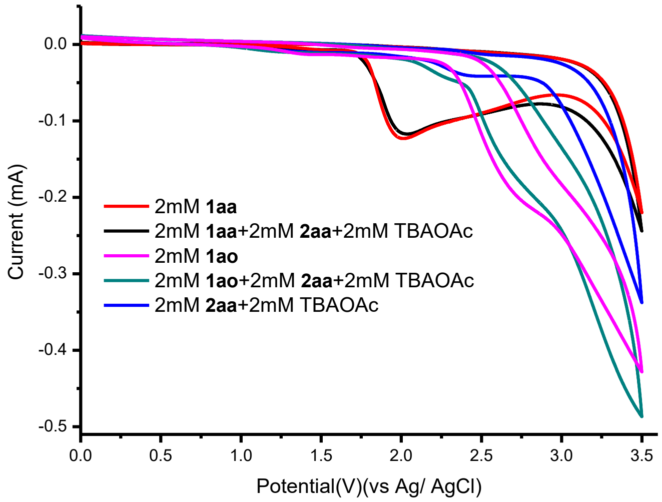

Supplementary Figure 150. Cyclic voltammetry studies. Cyclic voltammetry of **1aa** (0.002M), a mixture of **2aa** (0.002M) with 1 equivalent $^{n}Bu_4NOAc$ (0.002M), a mixture of **1aa** (0.002M) with 1 equivalent **2aa** (0.002M) and 1 equivalent $^{n}Bu_4NOAc$ (0.002M), **1ao** (0.002M), a mixture of **1ao** (0.002M) with 1 equivalent **2aa** (0.002M) and 1 equivalent $^{n}Bu_4NOAc$ (0.002M) in acetonitrile containing 0.1 M $^{n}Bu_4NBF_4$ under nitrogen at a glass carbon electrode at a scan rate of v=0.05 V/s.

Supplementary Methods

General Information: Unless otherwise stated, analytical grade solvents and commercially available reagents were used without further purification. All solvents were analytical reagent or better and were degassed prior to use. The instrument for electrolysis is dual display potentiostat (DJS-292B) (made in China). The anode electrode is carbon rod electrodes (Φ 6mm) and the cathode electrode is platinum plate electrodes (15 mm×15 mm×0.3 mm). Thin layer chromatography (TLC) employed glass 0.25 mm silica gel plates. Flash chromatography columns were packed with 200-300 mesh silica gel in petroleum ether (bp. 60−90 °C). Gradient flash chromatography was conducted eluting with a continuous gradient from petroleum ether to the ethyl acetate. Gas chromatographic analyses were performed on SHIMADZU GC-2014 gas chromatography instrument with a FID detector and biphenyl was added as internal standard. All new compounds were characterized by ^{1}H NMR, ^{13}C NMR and HRMS. The known compounds were characterized by ^{1}H NMR and ^{13}C NMR. The ^{1}H and ^{13}C NMR spectra were recorded on a Bruker 400 MHz NMR spectrometer. The chemical shifts (δ) were given in part per million relative to internal tetramethyl silane (TMS, 0 ppm for ^{1}H), $CDCl_3$ (77.0 ppm for ^{13}C). All chemical shifts (δ) were reported in ppm and coupling constants (*J*) in Hz. High resolution mass spectra (HRMS) were measured with a Waters Micromass GCT instrument and accurate masses were reported for the molecular ion + Sodium $(M+Na)^+$.

Procedure for Electrochemical Oxidative C(sp^2)-H Imidation: The synthesis of **3aa** is representative: Naphthalene (0.4 mmol, 1 equiv.), diphenylsulfonimide (0.6 mmol, 1.5 equiv.), $^{n}Bu_4NOAc$ (0.40 mmol, 1 equiv.) were placed in an oven-dried undivided three-necked bottle (25 mL). The bottle was equipped with a stir bar, a carbon rod (Φ 6mm) anode and a platinum plate (15 mm×15 mm×0.3 mm) cathode. The bottle was flushed with nitrogen. Degased dry dichloromethane (DCM, 10 mL), degased dry acetonitrile (MeCN, 2 mL) and commercially available hexafluoroisopropanol (HFIP, 0.5 mL) were added. The reaction mixture was stirred and electrolyzed at a constant current of 10 mA at room temperature for 4 h. After completion of the reaction, the product was identified by TLC. The solvent was removed under reduced pressure by an aspirator, then the pure product was obtained by flash column chromatography on silica gel (eluent: petroleum ether/ethyl acetate= 10:1).

Procedure for Gram Scale Synthesis: The synthesis of **3aa** is representative: Naphthalene (5.0 mmol, 1 equiv.), diphenylsulfonimide (7.5 mmol, 1.5 equiv.), $^{n}Bu_4NOAc$ (5.0 mmol, 1 equiv.) were placed in an oven-dried undivided three-necked bottle (100 mL). The bottle was equipped with a stir bar, a graphic rod (Φ 6mm)

anode and a platinum plate (15 mm×15 mm×0.3 mm) cathode. The bottle was flushed with nitrogen. Degased dry dichloromethane (DCM, 70 mL), degased dry acetonitrile (MeCN, 14 mL) and commercially available hexafluoroisopropanol (HFIP, 3.5 mL) were added. The reaction mixture was stirred and electrolyzed at a constant current of 10 mA at room temperature for 50 h. After completion of the reaction, the product was identified by TLC. The solvent was removed under reduced pressure by an aspirator, then the pure product was obtained by flash column chromatography on silica gel (eluent: petroleum ether/ethyl acetate= 10:1).

Procedure for Radical Trapping Experiment: Naphthalene (0.4 mmol, 1 equiv.), diphenylsulfonimide (0.6 mmol, 1.5 equiv.), $^{n}Bu_4NOAc$ (0.40 mmol, 1 equiv.), 2,6-di-tert- butyl-4-methylphenol (BHT, 2.0 equiv.) were placed in an oven-dried undivided three-necked bottle (25 mL). The bottle was equipped with a stir bar, a carbon rod (Φ 6mm) anode and a platinum plate (15 mm×15 mm×0.3 mm) cathode. The bottle was flushed with nitrogen. Degased dry dichloromethane (DCM, 10 mL), degased dry acetonitrile (MeCN, 2 mL) and commercially available hexafluoroisopropanol (HFIP, 0.5 mL) were added. The reaction mixture was stirred and electrolyzed at a constant current of 10 mA at room temperature for 4 h, the product was identified by TLC. And both of the reaction processes could be completely supressed. When BHT was used as a radical scavenger, the benzylic imidation product was obtained by flash column chromatography on silica gel (eluent: petroleum ether/ethyl acetate= 10:1) in 35% isolated yield. ^{1}H NMR spectra for **10aa:** 1H NMR (400 MHz, $CDCl_3$) δ 7.78 (dd, *J* = 8.5, 1.1 Hz, 4H), 7.58 – 7.49 (m, 2H), 7.46 – 7.31 (m, 4H), 7.19 (s, 2H), 5.21 (s, 1H), 4.90 (s, 2H), 1.35 (s, 18H). 13C NMR (101 MHz, $CDCl_3$) δ 153.58, 140.34, 135.71, 133.33, 128.62, 127.84, 126.20, 124.73, 53.10, 34.16, 30.11.

Procedure for Radical Clock Experiment: (1-Cyclopropylprop-1-en-1-yl)benzene (0.4 mmol, 1 equiv.), diphenylsulfonimide (0.6 mmol, 1.5 equiv.), $^{n}Bu_4NOAc$ (0.40 mmol, 1 equiv.), 2,2,6,6-tetramethyl-1-piperidinyloxy (TEMPO, 1.0 equiv.) were placed in an oven-dried undivided three-necked bottle (25 mL). The bottle was equipped with a stir bar, a carbon rod (Φ 6mm) anode and a platinum plate (15 mm×15 mm×0.3 mm) cathode. The bottle was flushed with nitrogen. Degased dry dichloromethane (DCM, 10 mL), degased dry acetonitrile (MeCN, 2 mL) and commercially available hexafluoroisopropanol (HFIP, 0.5 mL) were added. The reaction mixture was stirred and electrolyzed at a constant current of 10 mA at room temperature for 4 h, the product was identified by TLC. the ring-opening and radical trapping product **13aa** was obtained by flash column chromatography on silica gel (eluent: petroleum ether/ethyl acetate= 10:1) in 13% isolated yield. ^{1}H NMR spectra

for **13aa:** ^{1}H NMR (400 MHz, $CDCl_3$) δ 8.14 – 8.01 (m, 4H), 7.70 – 7.61 (m, 2H), 7.59 – 7.51 (m, 4H), 7.51 – 7.43 (m, 2H), 7.34 – 7.21 (m, 3H), 5.52 (t, *J* = 7.2 Hz, 1H), 4.89 (q, *J* = 6.8 Hz, 1H), 3.91 – 3.60 (m, 2H), 3.02 – 2.68 (m, 2H), 1.52 – 1.41 (m, 4H), 1.35 (d, *J* = 6.9 Hz, 3H), 1.20 – 1.23 (m, 5H), 1.13 (s, 3H), 1.03 (s, 6H). ^{13}C NMR (101 MHz, $CDCl_3$) δ 145.70, 141.17, 139.69, 133.69, 128.96, 128.02, 127.72, 127.37, 126.63, 124.52, 80.04, 59.74, 59.15, 48.45, 40.32, 40.20, 33.96, 33.83, 29.58, 29.55, 20.88, 20.39, 20.22, 17.00. HRMS (ESI) calcd for $C_{33}H_{43}N_2O_5S_2^+$, $[M+H]^+$, 611.2608, found: 611.2603. $C_{33}H_{42}N_2O_5S_2Na^+$, $[M+Na]^+$, 633.2427, found: 633.2420.

Procedure for Competitive Kinetic Isotope Effect Experiments： The substrate **1aa** (0.2 mmol), **[D8]-1aa** (0.2 mmol), diphenylsulfonimide (0.6 mmol, 1.5 equiv.), ^{n}Bu$_4$NOAc (0.40 mmol, 1 equiv.) were placed in an oven-dried undivided three-necked bottle (25 mL). The bottle was equipped with a stir bar, a carbon rod (Φ 6mm) anode and a platinum plate (15 mm×15 mm×0.3 mm) cathode. The bottle was flushed with nitrogen. Degased dry dichloromethane (DCM, 10 mL), degased dry acetonitrile (MeCN, 2 mL) and commercially available hexafluoroisopropanol (HFIP, 0.5 mL) were added. The reaction mixture was stirred and electrolyzed at a constant current of 10 mA at room temperature for 30min. The solvent was removed under reduced pressure by an aspirator, then the pure product was obtained by flash column chromatography on silica gel (eluent: petroleum ether/ethyl acetate= 10:1) to afford a mixture of **3aa/ [D8]-3aa** in 18% yield. ^{1}H NMR spectra for the isolated product was shown as follow, which indicate that the ratio of **3aa: [D8]-3aa** is 0.82:1. ^{1}H NMR spectra for **[D8]-3aa:** ^{1}H NMR (400 MHz, $CDCl_3$) δ 8.04 – 7.91 (m, 4H), 7.70 – 7.61 (m, 2H), 7.53 (dd, *J* = 10.7, 4.9 Hz, 1H).

Procedure for Parallel Kinetic Isotope Effect Experiments： The substrate **1aa** (0.4 mmol) or **[D8]-1aa** (0.4 mmol), diphenylsulfonimide (0.6 mmol, 1.5 equiv.), ^{n}Bu$_4$NOAc (0.40 mmol, 1 equiv.) were placed in an oven-dried undivided three-necked bottle (25 mL). The bottle was equipped with a stir bar, a carbon rod (Φ 6mm) anode and a platinum plate (15 mm×15 mm×0.3 mm) cathode. The bottle was flushed with nitrogen. Degased dry dichloromethane (DCM, 10 mL), degased dry acetonitrile (MeCN, 2 mL) and commercially available hexafluoroisopropanol (HFIP, 0.5 mL) were added. The reaction mixture was stirred and electrolyzed at a constant current of 10 mA at room temperature for 30min. The solvent was removed under reduced pressure by an aspirator, then the pure product was obtained by flash column chromatography on silica gel (eluent: petroleum ether/ethyl acetate= 10:1) to afford **2aa** in 18% yield, **[D8]-2aa** in 17% yield, respectively. Which indicate that the ratio of **3aa: [D8]-3aa** is 1.06:1.

Procedure for Cyclic Voltammetry (CV):

Cyclic voltammetry experiment was performed in a three-electrode cell connected to a schlenk line under nitrogen at room temperature. The working electrode was a glass carbon electrode, the counter electrode a platinum wire. The reference was an Ag|AgCl electrode submerged in saturated aqueous KCl solution, and separated from reaction by a salt bridge. Anhydrous Degassed acetonitrile containing 1.0 mmol $^{n}Bu_4NBF_4$ were poured into the electrochemical cell in all experiments. The scan rate is 0.05 V/s, ranging from 0 V to 3.5 V). Data was analyzed using MATLAB by subtracting a background current prior to identifying the maximum current (C_p) and determining the potential ($E_{p/2}$) at half this value ($C_{p/2}$). The obtained value was referenced to Ag|AgCl and converted to SCE by subtracting 0.03 V. In Supplementary Figure 148, we tested the cyclic voltammetry of **2aa** (0.002M), $^{n}Bu_4NOAc$(0.002M), and a mixture of **2aa** (0.002M) with 1 equivalent $^{n}Bu_4NOAc$ (0.002M) in acetonitrile containing 0.1 M $^{n}Bu_4NBF_4$. An oxidation peak of **2aa** in acetonitrile was observed at 3.40 V (vs Ag|AgCl), the half-peak potential was measured as 3.05 V (vs Ag|AgCl). An oxidation peak of $^{n}Bu_4NOAc$ (tetrabutylammonium acetate) in acetonitrile was observed at 1.92 V (vs Ag|AgCl), the half-peak potential was measured as 1.78 V (vs Ag|AgCl). An oxidation peak of a mixture of **2aa** and 1 equivalent $^{n}Bu_4NOAc$ in acetonitrile was observed at 2.38 V (vs Ag|AgCl), the half-peak potential was measured as 2.25 V (vs Ag|AgCl). In Supplementary Figure 149, we tested the cyclic Voltammetry of dibenzenesulfonimide (2aa) with different loading of tetrabutylammonium acetate ($^{n}Bu_4NOAc$). Experiments were run using a 0.002 M dibenzenesulfonimide (**2aa**) and various concentration of tetrabutylammonium acetate ($^{n}Bu_4NOAc$). In Supplementary Figure 150, we thsted the cyclic voltammetry of naphthalene (**1aa**), a mixture of dibenzenesulfonimide (**2aa**) with 1 equivalent $^{n}Bu_4NOAc$, a mixture of **1aa** with **2aa** and $^{n}Bu_4NOAc$; Methy thiophene-2-carboxylate (**2ao**), a mixture of **1ao** with **2aa** and $^{n}Bu_4NOAc$. An oxidation peak of **1aa** in acetonitrile was observed at 2.00 V (vs Ag|AgCl), the half-peak potential was measured as 1.84 V (vs Ag|AgCl). An oxidation peak of a mixture of **2aa** and 1 equivalent $^{n}Bu_4NOAc$ in acetonitrile was observed at 2.38 V (vs Ag|AgCl), the half-peak potential was measured as 2.25 V (vs Ag|AgCl). An oxidation peak of **1ao** in acetonitrile was observed at 2.67 V (vs Ag|AgCl), the half-peak potential was measured as 2.48 V (vs Ag|AgCl). The oxidation peak of a mixture of **1aa**, **2aa** and $^{n}Bu_4NOAc$ is the same as **1aa**. The oxidation peak of **1ao**, **2aa** and $^{n}Bu_4NOAc$ is the same as two individual oxidation peaks which belongs to **1ao** and the mixture of **2aa** and $^{n}Bu_4NOAc$.

Procedure for Mono-Desulfonation: To a solution of N-(naphthalen-1-yl)-N-(phenylsulfonyl)benzenesulfonamide (**3aa**) (0.1 mmol, 1 equiv) in dry MeOH (3.0 mL) was added Mg powder (1.0 mmol, 10 equiv) and the suspension was refluxed under nitrogen atmosphere for 12 h. Saturated solution of NH_4Cl (2.0 mL) was then added and the resulting mixture was extracted with diethyl ether (3 × 15 mL). The combined organic layers were dried ($MgSO_4$), filtered, and concentrated in vacuo. The residue was purified by flash chromatography on silica gel, eluting with hexane/EtOAc (v/v＝10:1) to afford 28.0 mg of the title compound as a white solid (99% yield). NMR Spectroscopy of **8aa**: ^{1}H NMR (400 MHz, CDCl3) δ 7.84 – 7.77 (m, 2H), 7.77 – 7.71 (m, 3H), 7.51 – 7.32 (m, 7H), 6.99 (s, 1H). ^{13}C NMR (101 MHz, CDCl3) δ 139.14, 134.17, 132.91, 131.16, 128.92 (two peaks overlapped), 128.39, 127.42, 127.24, 126.65, 126.27, 125.40, 123.18, 121.33.

Procedure for Double-Desulfonation： The transformation from **3aa** to **5aa** is representative: Under an argon atmosphere, to a mixture of sulfonamide **3aa** (0.1 mmol, 1.0 equiv.) and Mg powder (1.0 mmol, 10 equiv.) in THF (2 mL) were added Ti(O-*i*-Pr)$_4$ (0.2 mmol, 2.0 equiv.) and Me_3SiCl (0.3 mmol, 3.0 equiv.). The resulting mixture was stirred at 50 ^{o}C. After checking consumption of the substrate by TLC analysis, the mixture was filtered through a pad of celite. The solvent was removed under reduced pressure by an aspirator, then the corresponding amine **9aa** was obtained as by flash column chromatography on silica gel. NMR Spectroscopy of **9aa**: ^{1}H NMR (400 MHz, CDCl3) δ 7.81 (td, *J* = 5.6, 1.8 Hz, 2H), 7.51 – 7.41 (m, 2H), 7.34 – 7.26 (m, 2H), 6.78 (dd, *J* = 6.8, 1.6 Hz, 1H), 4.14 (s, 2H). ^{13}C NMR (101 MHz, CDCl3) δ 142.00, 134.33, 128.51, 126.29, 125.81, 124.83, 123.60, 120.74, 118.95, 109.65. NMR Spectroscopy of **9ab**: ^{1}H NMR (400 MHz, CDCl3) δ 8.71 (dd, *J* = 8.5, 1.0 Hz, 1H), 8.56 (d, *J* = 8.2 Hz, 1H), 7.92 (dd, *J* = 8.0, 1.3 Hz, 1H), 7.73 – 7.58 (m, 3H), 7.53 – 7.46 (m, 1H), 7.45 – 7.39 (m, 1H), 6.99 (s, 1H), 4.14 (s, 1H). ^{13}C NMR (101 MHz, CDCl3) δ 139.83, 133.16, 131.10, 126.86, 126.60, 126.28, 126.24, 126.09, 125.43, 123.37, 123.33, 122.42, 121.25, 107.37. NMR Spectroscopy of **9ad**: ^{1}H NMR (400 MHz, $CDCl_3$) δ 7.96 (d, *J* = 6.9 Hz, 1H), 7.88 (d, *J* = 7.4 Hz, 1H), 7.79 (dd, *J* = 11.8, 7.9 Hz, 2H), 7.72 (d, *J* = 7.5 Hz, 1H), 7.59 (dd, *J* = 8.2, 7.0 Hz, 1H), 7.34 (td, *J* = 7.4, 1.0 Hz, 1H), 7.28 (dd, *J* = 7.4, 1.0 Hz, 1H), 6.76 (d, *J* = 7.5 Hz, 1H), 4.45 (s, 2H). ^{13}C NMR (101 MHz, $CDCl_3$) δ 143.98, 139.77, 138.24, 136.98, 133.29, 127.46, 127.31, 126.24, 125.44, 122.03, 121.37, 120.67, 120.26, 120.09, 110.34. NMR Spectroscopy of **9af**: ^{1}H NMR (400 MHz, $CDCl_3$) δ 7.57 – 7.50 (m, 2H), 7.45 – 7.36 (m, 4H), 7.30 – 7.24 (m, 1H), 6.82 – 6.69 (m, 2H), 3.70 (s, 2H). ^{13}C NMR (101 MHz, $CDCl_3$) δ 145.78, 141.10, 131.52, 128.62, 127.97, 126.36, 126.21, 115.34.

Analytical Data of Compounds

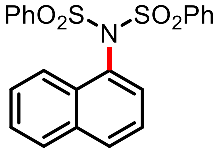

N-(naphthalen-1-yl)-N-(phenylsulfonyl)benzenesulfonamide (**3aa**)[1], 128.6 mg (yield: 76%, 0.4 mmol scale), white solid. ^{1}H NMR (400 MHz, $CDCl_3$) δ 8.01 – 7.90 (m, 5H), 7.84 (d, *J* = 8.2 Hz, 1H), 7.72 – 7.61 (m, 2H), 7.58 – 7.48 (m, 5H), 7.48 – 7.36 (m, 2H), 7.32 – 7.25 (m, 1H), 7.10 (d, *J* = 7.3 Hz, 1H); ^{13}C NMR (101 MHz, $CDCl_3$) δ 139.01, 134.58, 134.06, 132.71, 131.12, 130.91, 130.49, 128.99, 128.90, 128.07, 126.95, 126.49, 124.94, 123.85.

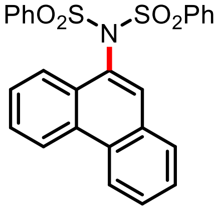

N-(phenanthren-9-yl)-N-(phenylsulfonyl)benzenesulfonamide (**3ab**)[1], 136.5 mg (yield: 72%, 0.4 mmol scale), white solid. ^{1}H NMR (400 MHz, $CDCl_3$) δ 8.65 (d, *J* = 7.6 Hz, 2H), 7.97 (d, *J* = 8.2 Hz, 4H), 7.76 – 7.63 (m, 5H), 7.62 – 7.54 (m, 2H), 7.49 (t, *J* = 7.4 Hz, 4H), 7.42 – 7.29 (m, 2H); ^{13}C NMR (101 MHz, $CDCl_3$) δ 138.87, 134.12, 132.04, 131.44, 131.06, 130.56, 130.49, 129.73, 129.22, 129.05, 128.87, 128.49, 127.23, 127.07, 126.81, 124.77, 122.70, 122.68.

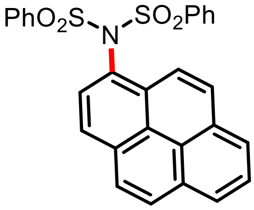

N-(phenylsulfonyl)-N-(pyren-1-yl)benzenesulfonamide (**3ac**)[1], 101.4 mg (yield: 51%, 0.4 mmol scale), white solid. ^{1}H NMR (400 MHz, $CDCl_3$) δ 8.23 (d, *J* = 7.5 Hz, 1H), 8.16 (dd, *J* = 8.1, 3.2 Hz, 2H), 8.09 (dd, *J* = 13.4, 8.6 Hz, 2H), 8.03 (t, *J* = 7.6 Hz, 1H), 7.99 – 7.93 (m, 4H), 7.87 (d, *J* = 9.3 Hz, 1H), 7.74 – 7.64 (m, 3H), 7.60 (d, *J* = 8.2 Hz, 1H), 7.52 (t, *J* = 7.9 Hz, 4H); ^{13}C NMR (101 MHz, $CDCl_3$) δ 139.20, 134.09, 132.73, 131.64, 130.78, 130.48, 129.51, 129.23, 129.00 (two peaks overlapped), 128.67, 127.40, 126.91, 126.48, 126.19, 126.07, 125.46, 124.62, 124.03, 122.70.

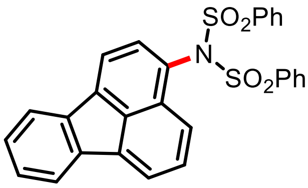

N-(fluoranthen-3-yl)-N-(phenylsulfonyl)benzenesulfonamide (**3ad**)[1], 149.5 mg (yield: 75%, 0.4 mmol scale), white solid. ^{1}H NMR (400 MHz, $CDCl_3$) δ 8.00 – 7.90 (m, 4H), 7.86 – 7.75 (m, 4H), 7.62 (t, *J* = 7.5 Hz, 2H), 7.48 (t, *J* = 7.9 Hz, 4H), 7.41 – 7.31 (m, 3H), 7.29 – 7.20 (m, 2H); ^{13}C NMR (101 MHz, $CDCl_3$) δ 139.86, 139.58, 138.90, 138.16, 136.89, 134.04, 133.37, 132.53, 130.14, 129.76, 128.90 (two peaks overlapped), 128.80, 128.43, 127.84, 123.52, 121.99, 121.65, 120.60, 119.38.

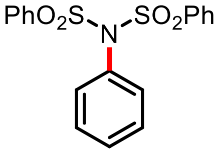

N-phenyl-N-(phenylsulfonyl)benzenesulfonamide (**3ae**)[2], 74.6 mg (yield: 50%, 0.4 mmol scale), white solid. ^{1}H NMR (400 MHz, $CDCl_3$) δ 7.94 (dd, *J* = 8.6, 1.6 Hz, 4H), 7.72 – 7.62 (m, 2H), 7.60 – 7.49 (m, 4H), 7.49 – 7.40 (m, 1H), 7.40 – 7.31 (m, 2H), 7.07 – 6.98 (m, 2H); ^{13}C NMR (101 MHz, $CDCl_3$) δ 139.33, 134.11, 133.94, 131.48, 130.29, 129.22, 128.96, 128.49.

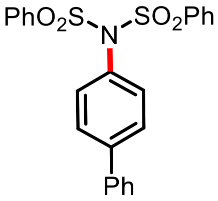

N-([1,1'-biphenyl]-4-yl)-N-(phenylsulfonyl)benzenesulfonamide (**3af**)[3], 113.4 mg (yield: 63%, 0.4 mmol scale), white solid. ^{1}H NMR (400 MHz, $CDCl_3$) δ 7.98 (dd, *J* = 8.4, 1.1 Hz, 4H), 7.72 – 7.62 (m, 2H), 7.61 – 7.49 (m, 8H), 7.48 – 7.40 (m, 2H), 7.40 – 7.30 (m, 1H), 7.16 – 7.02 (m, 2H); ^{13}C NMR (101 MHz, $CDCl_3$) δ 143.14, 139.52, 139.32, 133.96, 133.05, 131.70, 128.98, 128.84, 128.47, 127.96, 127.86, 127.16.

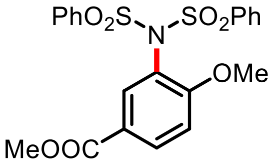

Methyl 4-methoxy-3-(N-(phenylsulfonyl)phenylsulfonamido)benzoate (**3ag**)[2], 88.5 mg (yield: 48%, 0.4 mmol scale), white solid. ^{1}H NMR (400 MHz, $CDCl_3$) δ 8.11 (dd, *J* = 8.7, 2.1 Hz, 1H), 8.02 – 7.89 (m, 4H), 7.82 (d, *J* = 2.1 Hz, 1H), 7.73 – 7.62 (m, 2H), 7.54 (t, *J* = 7.8 Hz, 4H), 6.87 (d, *J* = 8.7 Hz, 1H), 3.87 (s, 3H), 3.42 (s, 3H); ^{13}C NMR (101 MHz, $CDCl_3$) δ 165.54, 161.05, 139.64, 134.53, 133.78 (two peaks overlapped), 128.69, 128.64, 122.92, 122.65, 111.46, 55.46, 52.10.

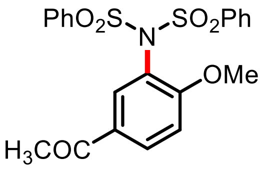

N-(5-acetyl-2-methoxyphenyl)-N-(phenylsulfonyl)benzenesulfonamide (**3ah**), 110.4 mg (yield: 62%, 0.4 mmol scale), white solid. ^{1}H NMR (400 MHz, $CDCl_3$) δ 8.04 (dd, *J* = 8.7, 2.2 Hz, 1H), 8.00 – 7.91 (m, 4H), 7.72 – 7.62 (m, 3H), 7.54 (t, *J* = 7.8 Hz, 4H), 6.91 (d, *J* = 8.7 Hz, 1H), 3.47 (s, 3H), 2.47 (s, 3H); ^{13}C NMR (101 MHz, $CDCl_3$) δ 195.33, 161.21, 139.59, 133.85, 133.59, 132.50, 130.22, 128.70, 128.67, 122.74, 111.64, 55.55, 26.22. HRMS (ESI) calcd for $C_{21}H_{19}NO_6S_2Na^+$, $[M+Na]^+$, 468.0546, found: 468.0534.

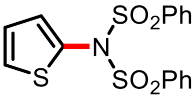

N-(phenylsulfonyl)-N-(thiophen-2-yl)benzenesulfonamide (**3ai**)[2], 107.3 mg (yield: 71%, 0.4 mmol scale), white solid. ^{1}H NMR (400 MHz, $CDCl_3$) δ 7.98 (dd, *J* = 8.6, 1.6 Hz, 4H), 7.74 – 7.62 (m, 2H), 7.60 – 7.46 (m, 4H), 7.37 (dd, *J* = 5.6, 1.4 Hz, 1H), 6.93 (dd, *J* = 5.6, 3.8 Hz, 1H), 6.74 (dd, *J* = 3.8, 1.4 Hz, 1H); ^{13}C NMR (101 MHz, $CDCl_3$) δ 138.49, 134.21, 133.74, 131.23, 129.00, 128.84, 128.60, 125.66.

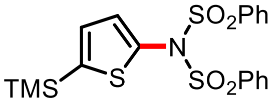

N-(phenylsulfonyl)-N-(5-(trimethylsilyl)thiophen-2-yl)benzenesulfonamide (**3aj**), 166.1 mg (yield: 92%, 0.4 mmol scale), white solid. ^{1}H NMR (400 MHz, $CDCl_3$) δ 8.08 – 7.90 (m, 4H), 7.72 – 7.62 (m, 2H), 7.61 – 7.46 (m, 4H), 7.05 (d, *J* = 3.6 Hz, 1H), 6.75 (d, *J* = 3.6 Hz, 1H), 0.30 (s, 9H); ^{13}C NMR (101 MHz, $CDCl_3$) δ 145.52, 138.59, 138.01, 134.11, 132.08, 132.04, 128.92, 128.59, -0.37. HRMS (ESI) calcd for $C_{19}H_{21}NO_4S_3SiNa^+$, $[M+Na]^+$, 474.0294, found: 474.0298.

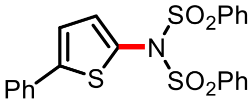

N-(phenylsulfonyl)-N-(5-phenylthiophen-2-yl)benzenesulfonamide (**3ak**)[1], 103.8 mg (yield: 57%, 0.4 mmol scale), white solid. ^{1}H NMR (400 MHz, $CDCl_3$) δ 8.12 – 7.96 (m, 4H), 7.73 – 7.63 (m, 2H), 7.61 – 7.49 (m, 6H), 7.41 – 7.34 (m, 2H), 7.33 – 7.26 (m, 1H), 7.12 (d, *J* = 4.0 Hz, 1H), 6.70 (d, *J* = 4.0 Hz, 1H); ^{13}C NMR (101 MHz, $CDCl_3$) δ 147.55, 138.58, 134.24, 133.35, 132.56, 132.30, 129.04, 128.93, 128.65, 128.42, 125.83, 121.34.

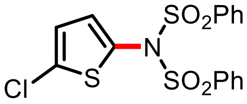

N-(5-chlorothiophen-2-yl)-N-(phenylsulfonyl)benzenesulfonamide (**3al**)[3], 153.8 mg (yield: 93%, 0.4 mmol scale), white solid. ^{1}H NMR (400 MHz, $CDCl_3$) δ 8.05 – 7.91 (m, 4H), 7.74 – 7.64 (m, 2H), 7.61 – 7.49 (m, 4H), 6.77 (d, *J* = 4.1 Hz, 1H), 6.53 (d, *J* = 4.1 Hz, 1H); ^{13}C NMR (101 MHz, $CDCl_3$) δ 138.25, 134.40, 132.98, 131.49, 131.24, 129.10, 128.57, 125.05.

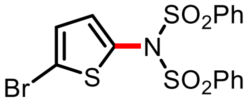

N-(5-bromothiophen-2-yl)-N-(phenylsulfonyl)benzenesulfonamide (**3am**)[2], 177.3 mg (yield: 97%, 0.4 mmol scale), white solid. ^{1}H NMR (400 MHz, $CDCl_3$) δ 8.06 – 7.87 (m, 4H), 7.69 (t, *J* = 7.5 Hz, 2H), 7.56 (t, *J* = 7.9 Hz, 4H), 6.93 (d, *J* = 4.0 Hz, 1H), 6.50 (d, *J* = 4.0 Hz, 1H); ^{13}C NMR (101 MHz, $CDCl_3$) δ 138.24, 134.39, 133.98, 132.09, 129.10, 128.67, 128.55, 115.59.

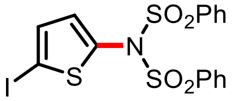

N-(5-iodothiophen-2-yl)-N-(phenylsulfonyl)benzenesulfonamide (**3an**), 145.4 mg (yield: 72%, 0.4 mmol scale), white solid. ^{1}H NMR (400 MHz, $CDCl_3$) δ 8.07 – 7.90 (m, 4H), 7.74 – 7.64 (m, 2H), 7.62 – 7.48 (m, 4H), 7.15 (d, *J* = 4.0 Hz, 1H), 6.39 (d, *J* = 4.0 Hz, 1H); ^{13}C NMR (101 MHz, $CDCl_3$) δ 138.27, 138.08, 135.52, 134.37, 132.75, 129.09, 128.56, 77.61. HRMS (ESI) calcd for $C_{16}H_{12}NO_4S_3Na^+$, $[M+Na]^+$, 527.8865, found: 527.8870.

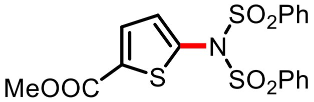

Methyl 5-(N-(phenylsulfonyl)phenylsulfonamido)thiophene-2-carboxylate (**3ao**), 101.4 mg (yield: 58%, 0.4 mmol scale), white solid. ^{1}H NMR (400 MHz, $CDCl_3$) δ 7.99 (d, *J* = 7.8 Hz, 4H), 7.80 – 7.68 (m, 2H), 7.66 – 7.55 (m, 5H), 6.73 (d, *J* = 4.0 Hz, 1H), 3.88 (s, 3H); ^{13}C NMR (101 MHz, $CDCl_3$) δ 161.69, 139.41, 138.26, 135.55, 134.51, 132.01, 131.49, 129.20, 128.68, 52.51. HRMS (ESI) calcd for $C_{18}H_{15}NO_6S_3Na^+$, $[M+Na]^+$, 459.9954, found: 459.9960.

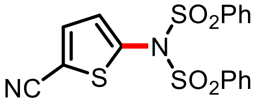

N-(5-cyanothiophen-2-yl)-N-(phenylsulfonyl)benzenesulfonamide (**3ap**)[2], 66.5 mg (yield: 41%, 0.4 mmol scale), white solid. ^{1}H NMR (400 MHz, $CDCl_3$) δ 8.02 – 7.91 (m, 4H), 7.79 – 7.70 (m, 2H), 7.64 – 7.55 (m, 4H), 7.47 (d, *J* = 4.1 Hz, 1H), 6.77 (d, *J* = 4.1 Hz, 1H); ^{13}C NMR (101 MHz, $CDCl_3$) δ 140.00, 137.84, 135.68, 134.78, 131.42, 129.33, 128.64, 112.99, 112.35.

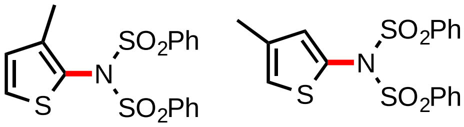

3aq **3aq'**

N-(3-methylthiophen-2-yl)-N-(phenylsulfonyl)benzenesulfonamide (**3aq**) and N-(4-methylthiophen-2-yl)-N-(phenylsulfonyl)benzenesulfonamide (**3aq'**) , 125.8 mg (yield: 80%, a mixture of regioisomers, **3aq**: **3aq'**= 5:1, 0.4 mmol scale), white solid. Major regioisomer **3aq**: ^{1}H NMR (400 MHz, $CDCl_3$) δ 8.04 – 7.92 (m, 4H), 7.75 – 7.64 (m, 2H), 7.55 (t, *J* = 7.8 Hz, 4H), 7.28 (d, *J* = 5.7 Hz, 1H), 6.79 (d, *J* = 5.7 Hz, 1H), 1.65 (s, 3H); ^{13}C

NMR (101 MHz, $CDCl_3$) δ 141.66, 138.91, 134.19, 129.02, 128.76, 128.42, 128.03, 127.23, 13.11. HRMS (ESI) calcd for $C_{17}H_{15}NO_4S_3Na^+$, $[M+Na]^+$, 416.0055, found: 416.0055.

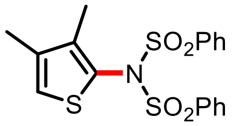

N-(3,4-dimethylthiophen-2-yl)-N-(phenylsulfonyl)benzenesulfonamide (**3ar**), 117.2 mg (yield: 72%, 0.4 mmol scale), white solid. ^1H NMR (400 MHz, $CDCl_3$) δ 8.11 – 7.88 (m, 4H), 7.67 (t, J = 7.5 Hz, 2H), 7.54 (t, J = 7.8 Hz, 4H), 6.95 (s, 1H), 2.08 (s, 3H), 1.56 (s, 3H); ^{13}C NMR (101 MHz, $CDCl_3$) δ 141.40, 138.92, 136.30, 134.12, 128.94, 128.74, 127.60, 122.73, 15.52, 12.10. HRMS (ESI) calcd for $C_{18}H_{17}NO_4S_3Na^+$, $[M+Na]^+$, 430.0212, found: 430.0211.

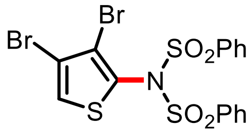

N-(3,4-dibromothiophen-2-yl)-N-(phenylsulfonyl)benzenesulfonamide (**3as**), 111.5 mg (yield: 52%, 0.4 mmol scale), white solid. ^1H NMR (400 MHz, $CDCl_3$) δ 8.13 – 7.97 (m, 4H), 7.70 (t, J = 7.5 Hz, 2H), 7.57 (t, J = 7.9 Hz, 4H), 7.48 (s, 1H); ^{13}C NMR (101 MHz, $CDCl_3$) δ 138.56, 134.59, 130.36, 129.12, 129.07, 125.94, 120.96, 113.30. HRMS (ESI) calcd for $C_{16}H_{11}Br_2NO_4S_3Na^+$, $[M+Na]^+$, 557.8109, found: 557.8106.

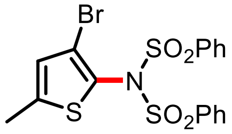

N-(3-bromo-5-methylthiophen-2-yl)-N-(phenylsulfonyl)benzenesulfonamide (**3at**), 150.7 mg (yield: 80%, 0.4 mmol scale), white solid. ^1H NMR (400 MHz, $CDCl_3$) δ 8.13 – 7.95 (m, 4H), 7.75 – 7.61 (m, 2H), 7.60 – 7.48 (m, 4H), 6.63 (d, J = 1.1 Hz, 1H), 2.46 (d, J = 1.0 Hz, 3H); ^{13}C NMR (101 MHz, $CDCl_3$) δ 143.66, 138.93, 134.33, 129.09, 129.02, 127.50, 126.42, 116.74, 16.28. HRMS (ESI) calcd for $C_{17}H_{14}BrNO_4S_3Na^+$, $[M+Na]^+$, 493.9161, found: 493.9168.

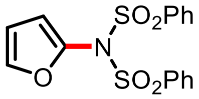

N-(furan-2-yl)-N-(phenylsulfonyl)benzenesulfonamide (**3au**), 71.4 mg (yield: 49%, 0.4 mmol scale), white solid. ^1H NMR (400 MHz, $CDCl_3$) δ 8.06 – 7.92 (m, 4H), 7.74 – 7.63 (m, 2H), 7.62 – 7.51 (m, 4H), 7.41 (dd, J = 2.0, 0.9 Hz, 1H), 6.45 (dd, J = 3.4, 2.1 Hz, 1H), 6.23 (dd, J = 3.4, 0.9 Hz, 1H); ^{13}C NMR (101 MHz, $CDCl_3$) δ 143.27, 139.05, 138.81, 134.25, 129.04, 128.51, 112.26, 111.84. HRMS (ESI) calcd for $C_{16}H_{13}NO_5S_2Na^+$, $[M+Na]^+$, 386.0127, found: 386.0128.

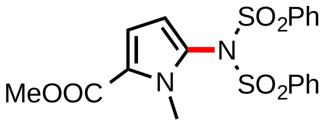

Methyl 1-methyl-5-(N-(phenylsulfonyl)phenylsulfonamido)-1H-pyrrole-2-carboxylate (**3av**), 130.4 mg (yield: 75%, 0.4 mmol scale), white solid. ^{1}H NMR (400 MHz, $CDCl_3$) δ 7.90 (dd, J = 8.4, 1.1 Hz, 4H), 7.76 – 7.62 (m, 2H), 7.61 – 7.50 (m, 4H), 6.88 (d, J = 4.3 Hz, 1H), 5.86 (d, J = 4.3 Hz, 1H), 3.82 (s, 3H), 3.45 (s, 3H). ^{13}C NMR (101 MHz, $CDCl_3$) δ 160.96, 138.33, 134.43, 129.05, 128.64, 125.93, 123.55, 115.87, 111.20, 51.34, 32.23. HRMS (ESI) calcd for $C_{19}H_{18}N_2O_6S_2Na^+$, $[M+Na]^+$, 457.0498, found: 457.0495.

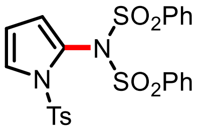

N-(phenylsulfonyl)-N-(1-tosyl-1H-pyrrol-2-yl)benzenesulfonamide (**3aw**), 115.6 mg (yield: 56%, 0.4 mmol scale), white solid. ^{1}H NMR (400 MHz, $CDCl_3$) δ 8.06 – 7.94 (m, 4H), 7.86 (d, J = 8.4 Hz, 2H), 7.68 (t, J = 7.5 Hz, 2H), 7.54 (t, J = 7.9 Hz, 4H), 7.27 (d, J = 8.9 Hz, 2H), 7.19 (dd, J = 3.6, 1.8 Hz, 1H), 6.25 (t, J = 3.7 Hz, 1H), 6.03 (dd, J = 3.6, 1.8 Hz, 1H), 2.39 (s, 3H); ^{13}C NMR (101 MHz, $CDCl_3$) δ 145.37, 138.25, 135.80, 134.25, 129.58, 129.56, 128.70, 128.42, 124.53, 122.27, 117.28, 110.71, 21.69. HRMS (ESI) calcd for $C_{23}H_{20}N_2O_6S_3Na^+$, $[M+Na]^+$, 539.0376, found: 539.0379.

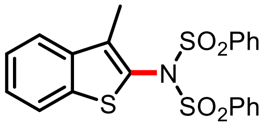

N-(3-methylbenzo[b]thiophen-2-yl)-N-(phenylsulfonyl)benzenesulfonamide (**3ax**), 97.5 mg (yield: 55%, 0.4 mmol scale), white solid. ^{1}H NMR (400 MHz, $CDCl_3$) δ 8.03 (d, J = 7.5 Hz, 4H), 7.78 – 7.73 (m, 1H), 7.70 (t, J = 7.5 Hz, 2H), 7.66 – 7.62 (m, 1H), 7.56 (t, J = 7.8 Hz, 4H), 7.45 – 7.36 (m, 2H), 1.82 (s, 3H); ^{13}C NMR (101 MHz, $CDCl_3$) δ 139.12, 138.95, 137.44, 137.28, 134.29, 129.09, 128.84, 128.30, 126.28, 124.38, 123.19, 122.49, 11.42. HRMS (ESI) calcd for $C_{21}H_{17}NO_4S_3Na^+$, $[M+Na]^+$, 466.0212, found: 466.0217.

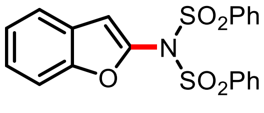

N-(Benzofuran-2-yl)-*N*-(phenylsulfonyl)benzenesulfonamide (**3ay**), 82.8mg (yield: 50%, 0.4 mmol scale), white solid. ^{1}H NMR (400 MHz, $CDCl_3$) δ 8.12 – 8.00 (m, 4H), 7.74 – 7.67 (m, 2H), 7.57 (t, J = 7.7 Hz, 5H), 7.47 (dd, J = 8.3, 0.5 Hz, 1H), 7.41 – 7.34 (m, 1H), 7.31 – 7.24 (m, 1H), 6.60 (d, J = 0.7 Hz, 1H); ^{13}C NMR (101 MHz, $CDCl_3$) δ 153.59, 141.54, 138.76, 134.40, 129.11, 128.62, 127.11, 126.26, 123.41, 121.88, 111.74, 109.00. HRMS (ESI) calcd for $C_{20}H_{15}NO_5S_2Na^+$, $[M+Na]^+$, 436.0284, found: 436.0286.

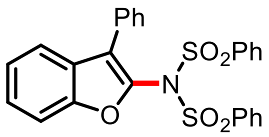

N-(3-Phenylbenzofuran-2-yl)-N-(phenylsulfonyl)benzenesulfonamide (**3az**), 180.3mg (yield: 92%, 0.4 mmol scale), white solid. ^{1}H NMR (400 MHz, $CDCl_3$) δ 7.96 – 7.83 (m, 4H), 7.66 (d, *J* = 7.9 Hz, 1H), 7.59 – 7.47 (m, 5H), 7.46 – 7.21 (m, 9H). ^{13}C NMR (101 MHz, $CDCl_3$) δ 152.79, 139.01, 137.47, 134.10, 129.45, 128.73, 128.69, 128.67, 128.49, 128.22, 127.37, 126.63, 123.48, 122.64, 121.27, 111.78. HRMS (ESI) calcd for $C_{26}H_{19}NO_5S_2Na^+$, $[M+Na]^+$, 512.0597, found: 516.0598.

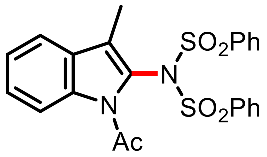

N-(1-Acetyl-3-methyl-1H-indol-2-yl)-N-(phenylsulfonyl)benzenesulfonamide (**3ba**), 99.5mg (yield: 53%, 0.4 mmol scale), white solid. ^{1}H NMR (400 MHz, $CDCl_3$) δ 8.10 – 8.02 (m, 4H), 7.99 (d, *J* = 8.5 Hz, 1H), 7.70 (t, *J* = 7.5 Hz, 2H), 7.56 (t, *J* = 7.9 Hz, 4H), 7.50 (d, *J* = 7.8 Hz, 1H), 7.46 – 7.40 (m, 1H), 7.30 (t, *J* = 7.3 Hz, 1H), 2.40 (s, 3H), 1.65 (s, 3H); ^{13}C NMR (101 MHz, $CDCl_3$) δ 168.73, 138.41, 135.07, 134.48, 129.61, 128.89, 127.87, 126.76, 123.44, 123.41, 123.07, 120.00, 115.63, 26.37, 9.13. HRMS (ESI) calcd for $C_{23}H_{20}N_2O_5S_2Na^+$, $[M+Na]^+$, 491.0706, found: 491.0709.

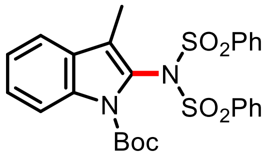

Tert-butyl 3-methyl-2-(N-(phenylsulfonyl)phenylsulfonamido)-1H-indole-1-carboxylate (**3bb**), 113.6mg (yield: 54%, 0.4 mmol scale), white solid. ^{1}H NMR (400 MHz, $CDCl_3$) δ 8.09 (d, *J* = 8.5 Hz, 1H), 8.06 – 8.00 (m, 4H), 7.65 (t, *J* = 7.4 Hz, 2H), 7.57 – 7.44 (m, 5H), 7.43 – 7.36 (m, 1H), 7.30 – 7.22 (m, 1H), 1.66 (s, 3H), 1.42 (s, 3H); ^{13}C NMR (101 MHz, $CDCl_3$) δ 148.28, 139.35, 135.13, 133.96, 129.51, 128.65, 127.58, 126.33, 123.41, 122.82, 122.58, 119.70, 116.27, 84.31, 27.87, 8.96. HRMS (ESI) calcd for $C_{26}H_{26}N_2O_6S_2Na^+$, $[M+Na]^+$, 549.1124, found: 549.1132.

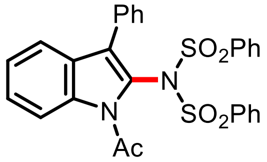

N-(1-Acetyl-3-phenyl-1H-indol-2-yl)-N-(phenylsulfonyl)benzenesulfonamide (**3bc**), 123.1 mg (yield: 58%, 0.4 mmol scale), white solid. ^{1}H NMR (400 MHz, $CDCl_3$) δ 7.91 – 7.84 (m, 4H), 7.81 (d, *J* = 8.4 Hz, 1H), 7.59 – 7.47 (m, 5H), 7.46 – 7.40 (m, 1H), 7.39 – 7.31 (m, 4H), 7.30 – 7.23 (m, 1H), 7.23 – 7.15 (m, 3H), 2.46 (s, 3H). ^{13}C NMR (101 MHz, $CDCl_3$) δ 168.97, 138.97, 134.68, 133.88, 130.38, 129.68, 129.57, 128.44, 128.39, 128.09, 127.85, 126.97,

126.70, 123.32, 123.20, 121.59, 114.60, 27.19. HRMS (ESI) calcd for $C_{28}H_{22}N_2O_5S_2Na^+$, $[M+Na]^+$, 553.0862, found: 553.0858.

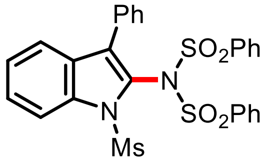

N-(1-(Methylsulfonyl)-3-phenyl-1H-indol-2-yl)-N-(phenylsulfonyl)benzenesulfonamide(**3bd**), 142.6 mg (yield: 63%, 0.4 mmol scale), white solid. ^{1}H NMR (400 MHz, $CDCl_3$) δ 8.05 (d, *J* = 8.4 Hz, 1H), 8.00 – 7.84 (m, 4H), 7.66 – 7.46 (m, 6H), 7.44 – 7.27 (m, 5H), 7.23 – 7.13 (m, 3H), 3.10 (s, 3H). ^{13}C NMR (101 MHz, $CDCl_3$) δ 138.94, 135.56, 133.85, 129.96, 129.63, 129.28, 128.52, 128.48, 128.44, 128.34, 127.92, 127.37, 124.49, 124.43, 121.88, 114.72, 39.57. HRMS (ESI) calcd for $C_{27}H_{22}N_2O_6S_3Na^+$, $[M+Na]^+$, 589.0532, found: 589.0535.

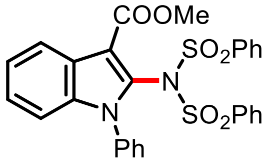

Methyl 1-phenyl-2-(N-(phenylsulfonyl)phenylsulfonamido)-1H-indole-3-carboxylate (**3be**), 124.5 mg (yield: 57%, 0.4 mmol scale), white solid. ^{1}H NMR (400 MHz, $CDCl_3$) δ 8.37 – 8.22 (m, 1H), 7.85 – 7.71 (m, 4H), 7.64 – 7.46 (m, 4H), 7.44 – 7.37 (m, 5H), 7.36 – 7.26 (m, 4H), 7.08 – 6.96 (m, 1H), 3.19 (s, 3H). ^{13}C NMR (101 MHz, $CDCl_3$) δ 163.01, 139.21, 136.99, 134.97, 133.77, 130.56, 129.47, 129.31, 129.26, 129.15, 128.52, 125.16, 125.09, 122.89, 122.25, 111.70, 108.67, 50.38. HRMS (ESI) calcd for $C_{28}H_{22}N_2O_6S_2Na^+$, $[M+Na]^+$, 569.0811, found: 569.0806.

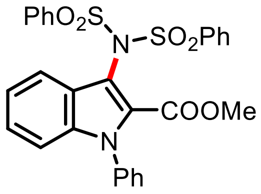

Methyl 1-phenyl-3-(N-(phenylsulfonyl)phenylsulfonamido)-1H-indole-2-carboxylate (**3bf**), 166.3 mg (yield: 76%, 0.4 mmol scale), white solid. ^{1}H NMR (400 MHz, $CDCl_3$) δ 8.11 – 7.93 (m, 4H), 7.68 – 7.58 (m, 2H), 7.56 – 7.45 (m, 7H), 7.40 – 7.32 (m, 2H), 7.31 – 7.19 (m, 2H), 7.17 – 7.06 (m, 2H), 3.16 (s, 3H). ^{13}C NMR (101 MHz, $CDCl_3$) δ 159.56, 139.90, 137.85, 137.42, 133.75, 129.14, 128.92, 128.66, 128.56, 127.82, 127.71, 126.06, 125.10, 122.44, 120.69, 115.44, 111.67, 51.21. HRMS (ESI) calcd for $C_{28}H_{22}N_2O_6S_2Na^+$, $[M+Na]^+$, 569.0811, found: 569.0808.

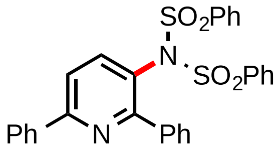

N-(2,6-Diphenylpyridin-3-yl)-N-(phenylsulfonyl)benzenesulfonamide (**3bg**), 134.7mg (yield: 64%, 0.4 mmol scale), white solid. ^{1}H NMR (400 MHz, $CDCl_3$) δ 8.08 (dd, *J* = 8.0, 1.6 Hz,

2H), 7.88 – 7.78 (m, 2H), 7.73 (dd, J = 8.4, 1.0 Hz, 4H), 7.65 (d, J = 8.4 Hz, 1H), 7.61 – 7.54 (m, 2H), 7.49 – 7.36 (m, 7H), 7.35 – 7.23 (m, 4H); ^{13}C NMR (101 MHz, $CDCl_3$) δ 159.72, 158.06, 140.14, 138.61, 138.03, 137.81, 134.06, 129.73, 129.63, 129.17, 128.72 (two peaks overlapped), 128.65, 128.09, 127.97, 127.26, 118.85. HRMS (ESI) calcd for $C_{29}H_{22}N_2O_4S_2Na^+$, $[M+Na]^+$, 549.0913, found: 549.0909.

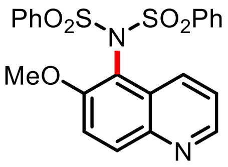

N-(2,6-Diphenylpyridin-4-yl)-N-(phenylsulfonyl)benzenesulfonamide (**3bh**), 99.8mg (yield: 55%, 0.4 mmol scale), white solid. ^{1}H NMR (400 MHz, $CDCl_3$) δ 8.78 (dd, J = 4.1, 1.5 Hz, 1H), 8.21 (d, J = 9.4 Hz, 1H), 8.00 – 7.84 (m, 5H), 7.71 – 7.58 (m, 2H), 7.50 (t, J = 7.9 Hz, 4H), 7.38 (d, J = 9.4 Hz, 1H), 7.28 – 7.23 (m, 1H), 3.41 (s, 3H); ^{13}C NMR (101 MHz, $CDCl_3$) δ 156.01, 148.63, 143.72, 139.57, 133.99, 133.83, 131.73, 129.44, 129.10, 128.54, 122.10, 115.63, 115.30, 55.52. HRMS (ESI) calcd for $C_{22}H_{18}N_2O_5S_2Na^+$, $[M+Na]^+$, 477.0549, found: 477.0561.

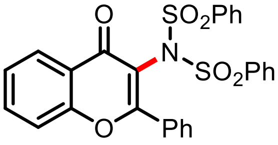

N-(4-Oxo-2-phenyl-4H-chromen-3-yl)-N-(phenylsulfonyl)benzenesulfonamide (**3bi**)[1], 120.1mg (yield: 58%, 0.4 mmol scale), white solid. ^{1}H NMR (400 MHz, $CDCl_3$) δ 8.13 (dd, J = 8.0, 1.5 Hz, 1H), 7.94 – 7.87 (m, 2H), 7.86 – 7.78 (m, 4H), 7.76 – 7.68 (m, 1H), 7.57 – 7.48 (m, 3H), 7.47 – 7.40 (m, 2H), 7.38 – 7.29 (m, 6H); ^{13}C NMR (101 MHz, $CDCl_3$) δ 174.51, 168.16, 155.45, 139.03, 134.44, 133.76, 131.36, 130.75, 129.50, 128.94, 128.30, 128.21, 126.27, 125.80, 123.36, 119.14, 118.03.

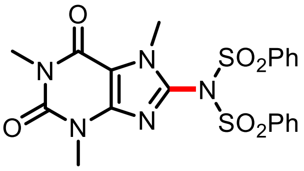

N-(Phenylsulfonyl)-N-(1,3,7-trimethyl-2,6-dioxo-2,3,4,5,6,7-hexahydro-1H-purin-8-yl)benzenesulfonamide(**3bj**)[1],155.4mg (yield: 79%, 0.4 mmol scale), white solid. ^{1}H NMR (400 MHz, $CDCl_3$) δ 7.90 (d, J = 7.5 Hz, 4H), 7.71 (t, J = 7.5 Hz, 2H), 7.53 (t, J = 7.9 Hz, 4H), 3.72 (s, 3H), 3.49 (s, 3H), 3.42 (s, 3H); ^{13}C NMR (101 MHz, CDCl3) δ 155.18, 151.30, 146.25, 137.93, 137.85, 134.83, 129.14, 129.03, 108.77, 32.32, 29.82, 28.08.

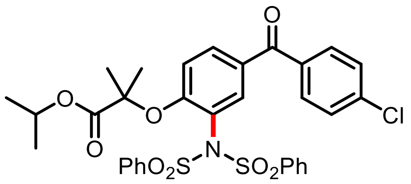

Isopropyl2-(4-(4-chlorobenzoyl)-2-(*N*-(phenylsulfonyl)phenylsulfonamido)phenoxy)-2-methylpropanoate (**3bk**)[2], 86.4mg (yield: 33%, 0.4 mmol scale), white solid. ^{1}H NMR (400 MHz, $CDCl_3$) δ 8.07 – 8.00 (m, 4H), 7.86 (dd, *J* = 8.7, 2.3 Hz, 1H), 7.73 – 7.62 (m, 4H), 7.59 – 7.50 (m, 5H), 7.49 – 7.39 (m, 2H), 6.70 (d, *J* = 8.7 Hz, 1H), 5.07 (sep, *J* = 6.3 Hz, 1H), 1.34 (s, 6H), 1.25 (d, *J* = 6.3 Hz, 6H). ^{13}C NMR (101 MHz, $CDCl_3$) δ 192.73, 172.43, 157.86, 140.17, 138.74, 135.82, 135.57, 133.72, 133.04, 131.12, 129.65, 128.89, 128.83, 128.69, 124.20, 115.78, 80.30, 69.36, 24.15, 21.60.

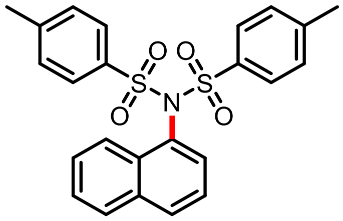

4-Methyl-N-(naphthalen-1-yl)-N-tosylbenzenesulfonamide (**3bl**)[3], 158.7mg (yield: 88%, 0.4 mmol scale), white solid. ^{1}H NMR (400 MHz, $CDCl_3$) δ 7.91 (d, *J* = 8.3 Hz, 1H), 7.86 – 7.76 (m, 5H), 7.61 (d, *J* = 8.5 Hz, 1H), 7.48 – 7.41 (m, 1H), 7.38 (t, *J* = 7.8 Hz, 1H), 7.33 – 7.26 (m, 5H), 7.08 (dd, *J* = 7.3, 0.9 Hz, 1H), 2.44 (s, 6H); ^{13}C NMR (101 MHz, $CDCl_3$) δ 145.11, 136.11, 134.54, 132.77, 131.07, 130.92, 130.41, 129.42, 128.99, 127.99, 126.83, 126.41, 124.88, 124.02, 21.66.

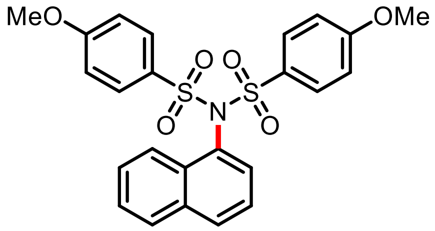

4-Methoxy-N-((4-methoxyphenyl)sulfonyl)-N-(naphthalen-1-yl)benzenesulfonamide (**3bm**), 148.8mg (yield: 77%, 0.4 mmol scale), white solid. ^{1}H NMR (400 MHz, $CDCl_3$) δ 7.90 (d, *J* = 8.3 Hz, 1H), 7.88 – 7.75 (m, 5H), 7.64 (d, *J* = 8.4 Hz, 1H), 7.48 – 7.40 (m, 1H), 7.37(t, *J* = 7.6 Hz, 1H), 7.35 – 7.28 (m, 1H), 7.08 (dd, *J* = 7.3, 0.9 Hz, 1H), 6.99 – 6.80 (m, 4H), 3.82 (s, 6H); ^{13}C NMR (101 MHz, $CDCl_3$) δ 163.85, 134.49, 132.77, 131.18 (two peaks overlapped), 130.79, 130.45, 130.29, 127.97, 126.83, 126.37, 124.87, 123.96, 113.89, 55.63. HRMS (ESI) calcd for $C_{24}H_{21}NO_6S_2Na^+$, $[M+Na]^+$, 506.0702, found: 506.0707.

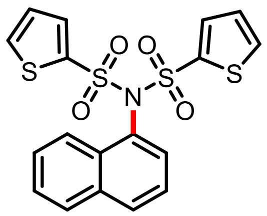

N-(naphthalen-1-yl)-N-(thiophen-2-ylsulfonyl)thiophene-2-sulfonamide (**3bn**), 74.8mg (yield: 43%, 0.4 mmol scale), white solid. ^{1}H NMR (400 MHz, $CDCl_3$) δ 7.95 (d, *J* = 8.3 Hz, 1H), 7.85 (d, *J* = 8.2 Hz, 1H), 7.79 (dd, *J* = 3.8, 1.3 Hz, 2H), 7.72 (dd, *J* = 5.0, 1.3 Hz, 2H), 7.64 (d, *J* = 8.2 Hz, 1H), 7.50 – 7.41 (m, 2H), 7.39 – 7.33 (m, 1H), 7.19 (dd, *J* = 7.4, 0.9 Hz, 1H), 7.12 (dd, *J* = 5.0, 3.9 Hz, 2H); ^{13}C NMR (101 MHz, $CDCl_3$) δ 138.69, 136.00, 134.67, 134.59, 132.77, 131.31, 130.62, 130.08, 128.14, 127.48, 127.20, 126.57, 125.02, 123.36. HRMS (ESI) calcd for $C_{18}H_{13}NO_4S_4Na^+$, $[M+Na]^+$, 457.9620, found: 457.9619.

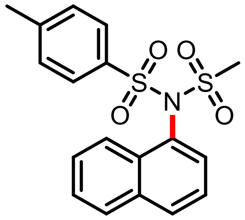

4-Methyl-N-(methylsulfonyl)-N-(naphthalen-1-yl)benzenesulfonamide (**3bo**), 123.3mg (yield: 82%, 0.4 mmol scale), white solid. ^{1}H NMR (400 MHz, $CDCl_3$) δ 7.95 (d, *J* = 8.3 Hz, 1H), 7.90 – 7.85 (m, 1H), 7.81 (d, *J* = 8.2 Hz, 1H), 7.78 – 7.72 (m, 2H), 7.60 – 7.40 (m, 3H), 7.38 – 7.15 (m, 3H), 3.61 (s, 3H), 2.45 (s, 3H); ^{13}C NMR (101 MHz, $CDCl_3$) δ 145.46, 135.13, 134.65, 132.62, 131.15, 130.48, 130.04, 129.49, 129.19, 128.32, 127.38, 126.60, 125.04, 123.37, 43.79, 21.71. HRMS (ESI) calcd for $C_{18}H_{17}NO_4S_2Na^+$, $[M+Na]^+$, 398.0491, found: 398.0495.

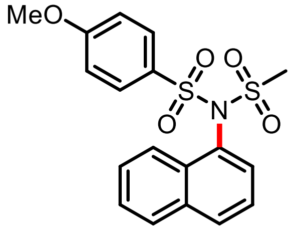

4-Methoxy-N-(methylsulfonyl)-N-(naphthalen-1-yl)benzenesulfonamide (**3bp**), 126.7mg (yield: 81%, 0.4 mmol scale), white solid. ^{1}H NMR (400 MHz, $CDCl_3$) δ 7.92 (d, *J* = 8.3 Hz, 1H), 7.88 – 7.73 (m, 4H), 7.54 – 7.36 (m, 3H), 7.24 (dd, *J* = 7.6, 1.1 Hz, 1H), 7.00 – 6.79 (m, 2H), 3.82 (s, 3H), 3.58 (s, 3H); ^{13}C NMR (101 MHz, $CDCl_3$) δ 164.09, 134.56, 132.58, 131.42, 131.03, 130.48, 129.97, 129.30, 128.26, 127.32, 126.54, 125.01, 123.31, 113.96, 55.65, 43.70. HRMS (ESI) calcd for $C_{18}H_{17}NO_5S_2Na^+$, $[M+Na]^+$, 414.0440, found: 414.0444.

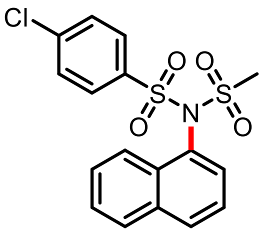

4-Chloro-N-(methylsulfonyl)-N-(naphthalen-1-yl)benzenesulfonamide (**3bq**), 129.6mg (yield: 82%, 0.4 mmol scale), white solid. ^{1}H NMR (400 MHz, $CDCl_3$) δ 7.94 (d, *J* = 8.3 Hz, 1H), 7.89 – 7.83 (m, 1H), 7.82 – 7.72 (m, 3H), 7.57 – 7.32 (m, 5H), 7.28 – 7.20 (m, 1H), 3.57 (s, 3H); ^{13}C NMR (101 MHz, $CDCl_3$) δ 140.93, 136.50, 134.60, 132.38, 131.30, 130.53, 130.14, 129.98, 129.15, 128.38, 127.50, 126.69, 125.06, 123.09, 43.75. HRMS (ESI) calcd for $C_{17}H_{14}ClNO_4S_2Na^+$, $[M+Na]^+$, 417.9945, found: 417.9951.

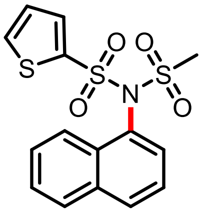

N-(methylsulfonyl)-N-(naphthalen-1-yl)thiophene-2-sulfonamide (**3br**), 108.7mg (yield: 74%, 0.4 mmol scale), white solid. ^{1}H NMR (400 MHz, $CDCl_3$) δ 7.95 (d, *J* = 8.3 Hz, 1H), 7.90 – 7.77 (m, 2H), 7.72 (dd, *J* = 3.9, 1.4 Hz, 1H), 7.68 (dd, *J* = 5.0, 1.3 Hz, 1H), 7.54 – 7.39 (m, 3H), 7.29 (dd, *J* = 7.3, 1.0 Hz, 1H), 7.08 (dd, *J* = 5.0, 3.9 Hz, 1H), 3.61 (s, 3H); ^{13}C NMR (101 MHz, $CDCl_3$) δ 137.61, 136.20, 134.91, 134.60, 132.56, 131.30, 130.08, 129.88, 128.37, 127.52, 126.63, 125.07, 122.93, 43.89. HRMS (ESI) calcd for $C_{15}H_{13}NO_4S_3Na^+$, $[M+Na]^+$, 389.9899, found: 389.9901.

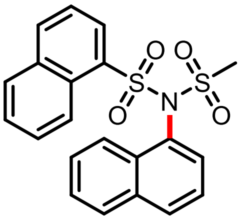

N-(methylsulfonyl)-N-(naphthalen-1-yl)naphthalene-1-sulfonamide (**3bs**), 108.5mg (yield: 66%, 0.4 mmol scale), white solid. ^{1}H NMR (400 MHz, $CDCl_3$) δ 8.41 (dd, *J* = 7.5, 0.8 Hz, 1H), 8.14 (d, *J* = 8.7 Hz, 1H), 8.05 (d, *J* = 8.2 Hz, 1H), 7.93 (d, *J* = 8.2 Hz, 1H), 7.80 (dd, *J* = 12.8, 8.2 Hz, 2H), 7.68 (d, *J* = 8.5 Hz, 1H), 7.57 – 7.40 (m, 4H), 7.37 (t, *J* = 7.5 Hz, 1H), 7.28 – 7.13 (m, 2H), 3.55 (s, 3H); ^{13}C NMR (101 MHz, $CDCl_3$) δ 135.96, 134.38, 133.90, 133.85, 132.88, 132.67, 131.26, 130.70, 130.00, 128.63, 128.43, 128.07, 126.99, 126.80, 126.45, 125.05, 124.92, 123.99, 123.61, 42.86. HRMS (ESI) calcd for $C_{21}H_{17}NO_4S_2Na^+$, $[M+Na]^+$, 434.0491, found: 434.0493.

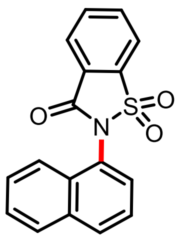

2-(Naphthalen-1-yl)benzo[d]isothiazol-3(2H)-one 1,1-dioxide (**3bt**), 105.3mg (yield: 85%, 0.4 mmol scale), white solid. ^{1}H NMR (400 MHz, $CDCl_3$) δ 8.23 – 8.15 (m, 1H), 8.09 – 8.00 (m, 2H), 7.98 – 7.81 (m, 4H), 7.71 (dd, *J* = 7.3, 1.1 Hz, 1H), 7.65 – 7.59 (m, 1H), 7.58 – 7.51 (m, 2H); ^{13}C NMR (101 MHz, $CDCl_3$) δ 158.73, 138.09, 135.16, 134.64, 134.47, 131.46, 131.40, 129.31, 128.47, 127.71, 126.99, 126.87, 125.73, 125.54, 124.60, 122.70, 121.37. HRMS (ESI) calcd for $C_{17}H_{11}NO_3SNa^+$, $[M+Na]^+$, 332.0352, found: 332.0352.

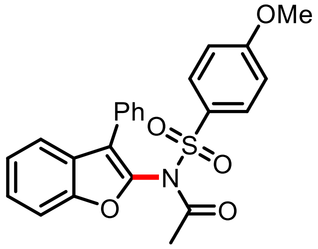

N-((4-methoxyphenyl)sulfonyl)-N-(3-phenylbenzofuran-2-yl)acetamide (**3bu**), 47.2 mg (yield: 28%, 0.4 mmol scale), white solid. ^{1}H NMR (400 MHz, $CDCl_3$) δ 8.03 – 7.94 (m, 2H), 7.76 (ddd, *J* = 7.9, 1.3, 0.7 Hz, 1H), 7.71 – 7.63 (m, 4H), 7.61 – 7.55 (m, 1H), 7.55 – 7.42 (m, 2H), 7.40 – 7.33 (m, 1H), 7.01 – 6.92 (m, 2H), 3.90 (s, 3H), 1.88 (s, 3H). ^{13}C NMR (101 MHz, $CDCl_3$) δ 169.45, 164.07, 152.64, 139.85, 131.90, 129.62, 129.57, 129.28, 128.58, 128.43, 127.31, 126.48, 123.70, 121.28, 120.89, 113.87, 111.82, 55.71, 23.64. HRMS (ESI) calcd for $C_{23}H_{19}NO_5SNa^+$, $[M+Na]^+$, 444.0876, found: 444.0871.

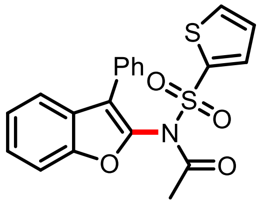

N-(3-phenylbenzofuran-2-yl)-N-(thiophen-2-ylsulfonyl)acetamide (**3bv**), 58.8 mg (yield: 37%, 0.4 mmol scale), white solid. ^{1}H NMR (400 MHz, $CDCl_3$) δ 7.85 (dd, *J* = 3.9, 1.4 Hz, 1H), 7.79 – 7.67 (m, 2H), 7.66 – 7.59 (m, 2H), 7.56 (d, *J* = 8.3 Hz, 1H), 7.52 – 7.39 (m, 4H), 7.38 – 7.32 (m, 1H), 7.09 (dd, *J* = 5.0, 3.8 Hz, 1H), 1.94 (s, 3H). ^{13}C NMR (101 MHz, $CDCl_3$) δ 169.51, 152.65, 139.50, 138.10, 136.48, 135.03, 129.44, 129.23, 128.62, 128.32, 127.15, 127.13, 126.58, 123.74, 121.27, 120.90, 111.83, 23.64. HRMS (ESI) calcd for $C_{20}H_{15}NO_4S_2Na^+$, $[M+Na]^+$, 420.0335, found: 420.0332.

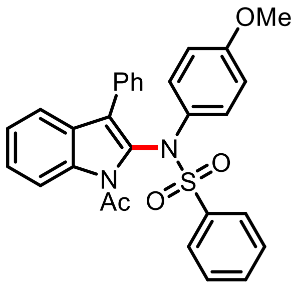

N-(1-acetyl-3-phenyl-1H-indol-2-yl)-N-(4-methoxyphenyl)benzenesulfonamide (**5aa**), 164.8 mg (yield: 83%, 0.4 mmol scale), white solid. ^{1}H NMR (400 MHz, Chloroform-*d*) δ 8.23 (dt, *J* = 8.4, 0.9 Hz, 1H), 7.52 – 7.45 (m, 3H), 7.45 – 7.35 (m, 2H), 7.30 – 7.09 (m, 8H), 7.08 – 7.02 (m, 2H), 6.84 – 6.71 (m, 2H), 3.76 (s, 3H), 2.87 (s, 3H). ^{13}C NMR (101 MHz, $CDCl_3$) δ 170.23, 156.91, 139.46, 135.57, 134.69, 132.82, 131.22, 129.57, 128.74, 128.50, 128.30, 127.85, 127.70, 127.66, 126.28, 123.40, 123.13, 122.40, 120.47, 115.90, 114.36, 55.39, 26.86. HRMS (ESI) calcd for $C_{29}H_{24}N_2O_4SNa^+$, $[M+Na]^+$, 519.1349, found: 519.1345.

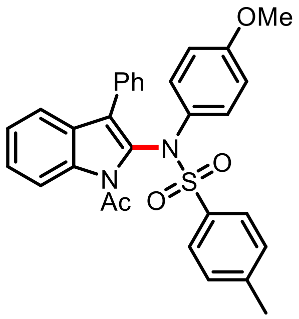

N-(1-acetyl-3-phenyl-1H-indol-2-yl)-N-(4-methoxyphenyl)-4-methylbenzenesulfonamide (**5ab**), 164.8 mg (yield: 83%, 0.4 mmol scale), white solid. ^{1}H NMR (400 MHz, $CDCl_3$) δ 8.28 (dt, *J* = 8.5, 0.9 Hz, 1H), 7.52 – 7.39 (m, 2H), 7.38 – 7.32 (m, 2H), 7.29 – 7.17 (m, 4H), 7.15 – 7.08 (m, 2H), 7.08 – 7.01 (m, 2H), 6.94 (d, *J* = 8.1 Hz, 2H), 6.84 – 6.74 (m, 2H), 3.77 (s, 3H), 2.86 (s, 3H), 2.31 (s, 3H). ^{13}C NMR (101 MHz, $CDCl_3$) δ 170.31, 156.69, 143.71, 136.34, 135.80, 134.81, 131.30, 129.58, 129.13, 128.65, 128.24, 127.67(two peaks overlapped), 127.49, 126.30, 123.42, 122.59, 122.32, 120.43, 116.09, 114.38, 55.39, 26.79, 21.46. HRMS (ESI) calcd for $C_{30}H_{26}N_2O_4SNa^+$, $[M+Na]^+$, 533.1505, found: 533.1500.

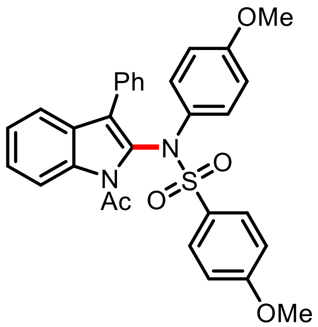

N-(1-acetyl-3-phenyl-1H-indol-2-yl)-4-methoxy-N-(4-methoxyphenyl)benzenesulfonamide (**5ac**), 199.8 mg (yield: 95%, 0.4 mmol scale), white solid. ^{1}H NMR (400 MHz, $CDCl_3$) δ 8.30 (d, *J* = 8.6 Hz, 1H), 7.54 – 7.34 (m, 4H), 7.30 – 7.17 (m, 4H), 7.17 – 7.10 (m, 2H), 7.10 – 7.03 (m, 2H), 6.84 – 6.76 (m, 2H), 6.65 – 6.51 (m, 2H), 3.78 (s, 3H), 3.76 (s, 3H), 2.86 (s,

3H). ^{13}C NMR (101 MHz, $CDCl_3$) δ 170.33, 162.86, 156.57, 135.86, 134.85, 131.39, 130.75, 129.87, 129.56, 128.66, 128.31, 127.65, 126.30, 123.42, 122.29, 122.24, 120.39, 116.17, 114.40, 113.66, 55.47, 55.38, 26.74. HRMS (ESI) calcd for $C_{30}H_{26}N_2O_5SNa^+$, $[M+Na]^+$, 549.1455, found: 549.1450.

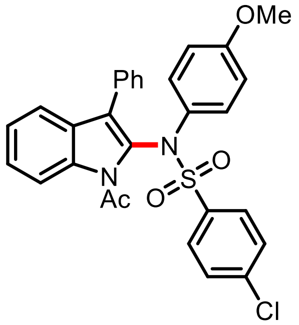

N-(1-acetyl-3-phenyl-1H-indol-2-yl)-4-chloro-N-(4-methoxyphenyl)benzenesulfonamide (**5ad**), 127.2 mg (yield: 60%, 0.4 mmol scale), white solid. ^{1}H NMR (400 MHz, $CDCl_3$) δ 8.22 (dt, *J* = 8.5, 0.9 Hz, 1H), 7.51 – 7.41 (m, 2H), 7.41 – 7.35 (m, 2H), 7.31 – 7.20 (m, 4H), 7.19 – 7.13 (m, 2H), 7.12 – 7.03 (m, 4H), 6.84 – 6.76 (m, 2H), 3.77 (s, 3H), 2.85 (s, 3H). ^{13}C NMR (101 MHz, $CDCl_3$) δ 170.05, 157.01, 139.37, 137.74, 135.34, 134.69, 131.09, 129.51, 128.93, 128.69, 128.39 (two peaks overlapped), 127.80, 127.56, 126.41, 123.50, 123.00, 122.51, 120.53, 115.93, 114.48, 55.39, 26.87. HRMS (ESI) calcd for $C_{29}H_{23}ClN_2O_4SNa^+$, $[M+Na]^+$, 553.0959, found: 553.0955.

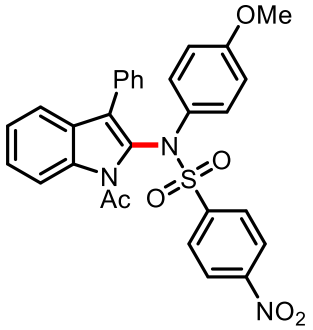

N-(1-acetyl-3-phenyl-1H-indol-2-yl)-N-(4-methoxyphenyl)-4-nitrobenzenesulfonamide (**5ae**), 112.5 mg (yield: 52%, 0.4 mmol scale), white solid. ^{1}H NMR (400 MHz, $CDCl_3$) δ 8.10 (dt, *J* = 8.4, 0.9 Hz, 1H), 8.03 – 7.93 (m, 2H), 7.70 – 7.57 (m, 2H), 7.54 – 7.39 (m, 2H), 7.34 – 7.10 (m, 8H), 6.86 – 6.73 (m, 2H), 3.78 (s, 3H), 2.86 (s, 3H). ^{13}C NMR (101 MHz, $CDCl_3$) δ 169.77, 157.68, 149.71, 144.98, 134.57, 134.47, 131.02, 129.67, 128.85, 128.48, 128.36, 128.13, 127.59, 126.54, 124.23, 123.64, 123.46, 123.01, 120.76, 115.60, 114.60, 55.43, 27.12. RMS (ESI) calcd for $C_{29}H_{23}N_3O_6SNa^+$, $[M+Na]^+$, 564.1200, found: 564.1199.

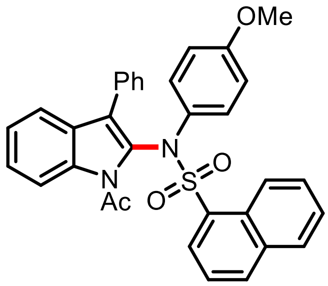

N-(1-acetyl-3-phenyl-1H-indol-2-yl)-N-(4-methoxyphenyl)naphthalene-1-sulfonamide (**5af**), 209.6 mg (yield: 96%, 0.4 mmol scale), white solid. ^{1}H NMR (400 MHz, $CDCl_3$) δ 8.25 (d, *J* = 8.7 Hz, 1H), 8.15 (d, *J* = 8.5 Hz, 1H), 7.94 – 7.83 (m, 2H), 7.80 (dt, *J* = 8.2, 0.9 Hz, 1H), 7.51 – 7.38 (m, 2H), 7.35 – 7.13 (m, 6H), 7.13 – 7.03 (m, 1H), 7.00 – 6.89 (m, 2H), 6.80 – 6.66 (m, 4H), 3.75 (s, 3H), 2.81 (s, 3H). ^{13}C NMR (101 MHz, $CDCl_3$) δ 170.23, 157.21, 136.07, 136.03, 134.31, 134.13, 130.82, 129.54, 128.94, 128.77, 128.74, 128.50, 127.91, 127.84, 127.80, 127.59, 126.67, 126.14, 125.22, 124.66, 123.87, 123.22, 122.97, 120.50, 115.36, 114.06 , 55.39, 27.00. RMS (ESI) calcd for $C_{33}H_{26}N_2O_4SNa^+$, $[M+Na]^+$, 569.1505, found: 569.1500.

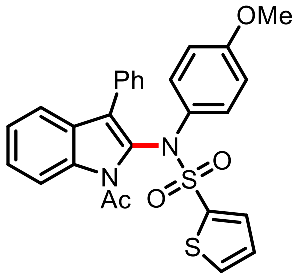

N-(1-acetyl-3-phenyl-1H-indol-2-yl)-N-(4-methoxyphenyl)thiophene-2-sulfonamide (**5ag**), 180.7 mg (yield: 90%, 0.4 mmol scale), white solid. ^{1}H NMR (400 MHz, $CDCl_3$) δ 8.27 (dt, *J* = 8.3, 0.9 Hz, 1H), 7.50 – 7.40 (m, 2H), 7.36 (dd, *J* = 5.0, 1.4 Hz, 1H), 7.33 – 7.28 (m, 2H), 7.28 – 7.23 (m, 1H), 7.24 – 7.19 (m, 1H), 7.18 – 7.12 (m, 2H), 7.11 – 7.04 (m, 3H), 6.88 – 6.78 (m, 2H), 6.73 (dd, *J* = 5.0, 3.8 Hz, 1H), 3.78 (s, 3H), 2.83 (s, 3H). ^{13}C NMR (101 MHz, $CDCl_3$) δ 170.10, 156.90, 139.25, 135.20, 134.78, 133.79, 132.69, 131.17, 129.56, 128.33, 128.16, 127.82, 127.75, 126.98, 126.40, 123.50, 122.77, 122.72, 120.47, 116.10, 114.42, 55.41, 26.77. RMS (ESI) calcd for $C_{27}H_{22}N_2O_4SNa^+$, $[M+Na]^+$, 525.0913, found: 525.0908.

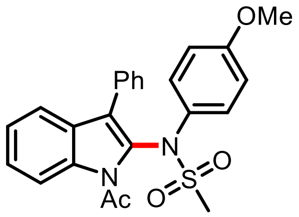

N-(1-acetyl-3-phenyl-1H-indol-2-yl)-N-(4-methoxyphenyl)methanesulfonamide (**5ah**), 144.1 mg (yield: 83%, 0.4 mmol scale), white solid. ^{1}H NMR (400 MHz, $CDCl_3$) δ 8.33 (dt, *J* = 8.4, 0.9 Hz, 1H), 7.51 – 7.42 (m, 2H), 7.43 – 7.33 (m, 5H), 7.29 (td, *J* = 7.5, 0.9 Hz, 1H), 7.24 – 7.17 (m, 2H), 6.91 – 6.77 (m, 2H), 3.79 (s, 3H), 2.75 (s, 3H), 2.55 (s, 3H). ^{13}C NMR (101

MHz, $CDCl_3$) δ 169.92, 156.71, 135.03, 134.81, 131.42, 129.80, 129.72, 128.78, 128.52, 128.41, 128.30, 127.77, 126.53, 123.75, 123.11, 121.34, 120.20, 116.56, 114.81, 55.44, 39.87, 26.62. RMS (ESI) calcd for $C_{24}H_{22}N_2O_4SNa^+$, $[M+Na]^+$, 457.1192, found: 457.1187.

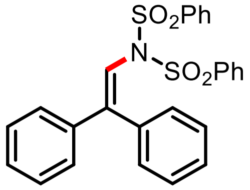

N-(2,2-diphenylvinyl)-N-(phenylsulfonyl)benzenesulfonamide (**7aa**), 133.3mg (yield: 70%, 0.4 mmol scale), white solid. ^1H NMR (400 MHz, $CDCl_3$) δ 7.69 (dd, J = 7.4, 1.0 Hz, 4H), 7.61 – 7.50 (m, 2H), 7.44 – 7.13 (m, 14H), 6.13 (s, 1H); ^{13}C NMR (101 MHz, $CDCl_3$) δ 152.19, 139.70, 138.44, 136.64, 133.78, 129.79, 129.05, 128.72, 128.62, 128.55, 128.31, 128.20, 128.10, 116.13. HRMS (ESI) calcd for $C_{26}H_{21}NO_4S_2Na^+$, $[M+Na]^+$, 498.0804, found: 498.0812.

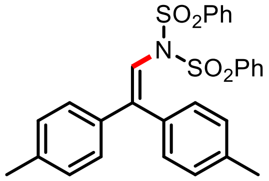

N-(2,2-di-p-tolylvinyl)-N-(phenylsulfonyl)benzenesulfonamide (**7ab**), 144.9mg (yield: 72%, 0.4 mmol scale), white solid. ^1H NMR (400 MHz, $CDCl_3$) δ 7.77 – 7.63 (m, 4H), 7.60 – 7.52 (m, 2H), 7.42 – 7.33 (m, 4H), 7.19 – 7.07 (m, 6H), 6.98 (d, J = 7.9 Hz, 2H), 6.05 (s, 1H), 2.46 – 4.26 (m, 6H). ^{13}C NMR (101 MHz, $CDCl_3$) δ 151.98, 139.10, 138.60, 137.95, 137.07, 133.84, 133.58, 129.74, 128.96, 128.76, 128.61, 128.57, 115.12, 21.20, 21.19. HRMS (ESI) calcd for $C_{28}H_{25}NO_4S_2Na^+$, $[M+Na]^+$, 526.1117, found: 526.1119.

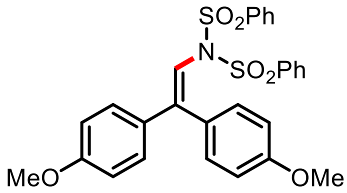

N-(2,2-bis(4-methoxyphenyl)vinyl)-N-(phenylsulfonyl)benzenesulfonamide (**7ac**), 124.2mg (yield: 58%, 0.4 mmol scale), white solid. ^1H NMR (400 MHz, $CDCl_3$) δ 7.81 – 7.65 (m, 4H), 7. 62 – 7.51 (m, 2H), 7.47 – 7.31 (m, 4H), 7.24 – 7.14 (m, 4H), 6.91 – 6.78 (m, 2H), 6.77 – 6.64 (m, 2H), 5.98 (s, 1H), 3.89 – 3.72 (m, 6H). ^{13}C NMR (101 MHz, $CDCl_3$) δ 160.36, 159.55, 151.24, 138.71, 133.64, 132.47, 131.26, 130.11, 129.11, 128.65, 128.56, 114.04, 113.63, 113.44, 55.29, 55.17. HRMS (ESI) calcd for $C_{28}H_{25}NO_6S_2Na^+$, $[M+Na]^+$, 558.1016, found: 558.1018.

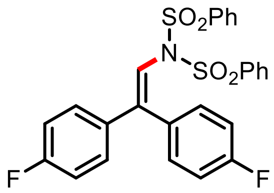

N-(2,2-bis(4-fluorophenyl)vinyl)-N-(phenylsulfonyl)benzenesulfonamide (**7ad**), 130.4mg (yield: 64%, 0.4 mmol scale), white solid. ^{1}H NMR (400 MHz, $CDCl_3$) δ 7.80 – 7.70 (m, 4H), 7.67 – 7.54 (m, 2H), 7.49 – 7.38 (m, 4H), 7.26 – 7.16 (m, 4H), 7.06 – 6.97 (m, 2H), 6.91 – 6.81 (m, 2H), 6.08 (s, 1H). ^{13}C NMR (101 MHz, $CDCl_3$) δ 164.25 (d, *J* = 69.1 Hz), 161.77 (d, *J* = 68.1 Hz), 149.92, 138.51, 135.61 (d, *J* = 3.3 Hz), 133.92, 132.33 (d, *J* = 3.4 Hz), 131.72 (d, *J* = 8.2 Hz), 130.47 (d, *J* = 8.3 Hz), 128.82, 128.47, 116.54, 115.48 (d, *J* = 21.7 Hz), 115.19 (d, *J* = 21.5 Hz). ^{19}F NMR (377 MHz, $CDCl_3$) δ -111.68, -112.79. HRMS (ESI) calcd for $C_{26}H_{19}F_2NO_4S_2Na^+$, $[M+Na]^+$, 534.0616, found: 534.0617.

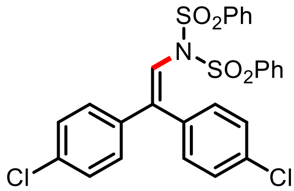

N-(2,2-bis(4-chlorophenyl)vinyl)-N-(phenylsulfonyl)benzenesulfonamide (**7ae**), 126.4mg (yield: 58%, 0.4 mmol scale), white solid. ^{1}H NMR (400 MHz, $CDCl_3$) δ 7.82 – 7.68 (m, 4H), 7.67 – 7.56 (m, 2H), 7.48 – 7.39 (m, 4H), 7.34 – 7.27 (m, 2H), 7.21 – 7.09 (m, 6H), 6.11 (s, 1H). ^{13}C NMR (101 MHz, $CDCl_3$) δ 149.60, 138.42, 137.66, 135.45, 134.69, 134.53, 133.94, 131.15, 129.92, 128.86, 128.71, 128.49, 128.47, 117.33. HRMS (ESI) calcd for $C_{26}H_{19}Cl_2NO_4S_2Na^+$, $[M+Na]^+$, 566.0025, found: 566.0027.

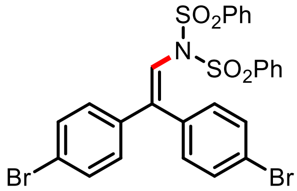

N-(2,2-bis(4-bromophenyl)vinyl)-N-(phenylsulfonyl)benzenesulfonamide (**7af**), 103.8mg (yield: 41%, 0.4 mmol scale), white solid. ^{1}H NMR (400 MHz, $CDCl_3$) δ7.80 – 7.69 (m, 4H), 7.67 – 7.59 (m, 2H), 7.50 – 7.40 (m, 6H), 7.33 – 7.27 (m, 2H), 7.14 – 7.06 (m, 4H), 6.12 (s, 1H). ^{13}C NMR (101 MHz, $CDCl_3$) δ 149.69, 138.41, 138.05, 134.94, 133.95, 131.69, 131.47, 131.44, 130.20, 128.89, 128.48, 123.77, 123.12, 117.34. HRMS (ESI) calcd for $C_{26}H_{19}Br_2NO_4S_2Na^+$, $[M+Na]^+$, 653.9014, found: 653.9017.

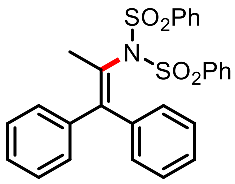

N-(1,1-diphenylprop-1-en-2-yl)-N-(phenylsulfonyl)benzenesulfonamide (**7ag**), 134.5mg (yield: 68%, 0.4 mmol scale), white solid. ^{1}H NMR (400 MHz, $CDCl_3$) δ 7.96 – 7.76 (m, 4H), 7.58 – 7.49 (m, 2H), 7.41 – 7.24 (m, 7H), 7.19 – 7.08 (m, 4H), 7.07 – 6.95 (m, 3H), 2.06 (s, 3H). ^{13}C NMR (101 MHz, $CDCl_3$) δ 148.43, 141.58, 139.87, 139.00, 133.58, 129.01, 128.85, 128.82, 128.68, 128.37, 127.64, 127.62, 127.54, 127.13, 21.93. HRMS (ESI) calcd for $C_{27}H_{23}NO_4S_2Na^+$, $[M+Na]^+$, 512.0961, found: 512.0970.

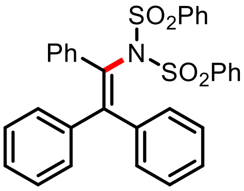

N-(phenylsulfonyl)-N-(1,2,2-triphenylvinyl)benzenesulfonamide (**7ah**), 141.1mg (yield: 64%, 0.4 mmol scale), white solid. ^{1}H NMR (400 MHz, $CDCl_3$) δ 7.69 (dd, J = 6.5, 2.9 Hz, 2H), 7.43 – 7.31 (m, 6H), 7.27 (dd, J = 5.1, 3.3 Hz, 2H), 7.17 – 6.96 (m, 13H), 6.90 (t, J = 7.6 Hz, 2H); ^{13}C NMR (101 MHz, $CDCl_3$) δ 147.15, 142.23, 140.27, 139.54, 136.76, 132.83, 131.84, 131.22, 130.88, 130.32, 128.90, 128.34, 128.16, 127.87, 127.71, 127.53, 127.22. HRMS (ESI) calcd for $C_{32}H_{25}NO_4S_2Na^+$, $[M+Na]^+$, 574.1117, found: 574.1130.

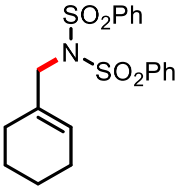

N-(cyclohex-1-en-1-ylmethyl)-N-(phenylsulfonyl)benzenesulfonamide (**7ai**), 46.9 mg (yield: 30%, 0.4 mmol scale), white solid. ^{1}H NMR (400 MHz, $CDCl_3$) δ 8.15 – 7.98 (m, 4H), 7.69 – 7.59 (m, 2H), 7.58 – 7.48 (m, 4H), 5.70 (t, J = 3.7, 1H), 4.38 (s, 2H), 1.98 – 1.82 (m, 2H), 1.80 – 1.66 (m, 2H), 1.42 – 1.29 (m, 2H), 1.29 – 1.18 (m, 2H). ^{13}C NMR (101 MHz, $CDCl_3$) δ 140.46, 133.52, 130.80, 128.81, 128.76, 128.14, 55.90, 25.28, 25.08, 21.84, 21.64. RMS (ESI) calcd for $C_{19}H_{21}NO_4S_2Na^+$, $[M+Na]^+$, 414.0804, found: 414.0799.

Supplementary References

1. Kawakami, T.; Murakami, K.; Itami, K., Catalytic C–H Imidation of Aromatic Cores of Functional Molecules: Ligand-Accelerated Cu Catalysis and Application to Materials- and Biology-Oriented Aromatics. *J. Am. Chem. Soc.*, **137**, 2460-2463 (2015).
2. Boursalian, G. B.; Ngai, M. Y.; Hojczyk, K. N.; Ritter, T., Pd-Catalyzed Aryl C–H Imidation with Arene as the Limiting Reagent. *J. Am. Chem. Soc.*, **135**, 13278–13281 (2013).
3. Ito, E.; Fukushima, T.; Kawakami, T. Murakami, K.; Itami, K., Catalytic Dehydrogenative C–H Imidation of Arenes Enabled by Photo-generated Hole Donation to Sulfonimide. *Chem*, **2**, 383-392 (2017).
